# Rapid Initiation of Injection Naltrexone for Opioid Use Disorder: A Stepped-Wedge Cluster Randomized Clinical Trial

**DOI:** 10.1001/jamanetworkopen.2024.9744

**Published:** 2024-05-08

**Authors:** Matisyahu Shulman, Miranda G. Greiner, Hiwot M. Tafessu, Onumara Opara, Kaitlyn Ohrtman, Kenzie Potter, Kathryn Hefner, Eve Jelstrom, Richard N. Rosenthal, Kevin Wenzel, Marc Fishman, John Rotrosen, Udi E. Ghitza, Edward V. Nunes, Adam Bisaga

**Affiliations:** 1 Department of Psychiatry, New York State Psychiatric Institute and Columbia University Irving Medical Center, New York, New York; 2 The Emmes Company LLC, Rockville, Maryland; 3 Department of Psychiatry, Stony Brook University, Stony Brook, New York; 4 Department of Psychiatry, Johns Hopkins University School of Medicine and Maryland Treatment Centers, Baltimore, Maryland; 5 Department of Psychiatry, New York University Grossman School of Medicine, New York, New York; 6 National Institute on Drug Abuse, Bethesda, Maryland

## Abstract

**Importance:**

Injectable extended-release (XR)-naltrexone is an effective treatment option for opioid use disorder (OUD), but the need to withdraw patients from opioid treatment prior to initiation is a barrier to implementation.

**Objective:**

To compare the effectiveness of the standard procedure (SP) with the rapid procedure (RP) for XR-naltrexone initiation.

**Design, Setting, and Participants:**

The Surmounting Withdrawal to Initiate Fast Treatment with Naltrexone study was an optimized stepped-wedge cluster randomized trial conducted at 6 community-based inpatient addiction treatment units. Units using the SP were randomly assigned at 14-week intervals to implement the RP. Participants admitted with OUD received the procedure the unit was delivering at the time of their admission. Participant recruitment took place between March 16, 2021, and July 18, 2022. The last visit was September 21, 2022.

**Interventions:**

Standard procedure, based on the XR-naltrexone package insert (approximately 5-day buprenorphine taper followed by a 7- to 10-day opioid-free period and RP, defined as 1 day of buprenorphine at minimum necessary dose, 1 opioid-free day, and ascending low doses of oral naltrexone and adjunctive medications (eg, clonidine, clonazepam, antiemetics) for opioid withdrawal.

**Main Outcomes and Measures:**

Receipt of XR-naltrexone injection prior to inpatient discharge (primary outcome). Secondary outcomes included opioid withdrawal scores and targeted safety events and serious adverse events. All analyses were intention-to-treat.

**Results:**

A total of 415 participants with OUD were enrolled (mean [SD] age, 33.6 [8.48] years; 205 [49.4%] identified sex as male); 54 [13.0%] individuals identified as Black, 91 [21.9%] as Hispanic, 290 [69.9%] as White, and 22 [5.3%] as multiracial. Rates of successful initiation of XR-naltrexone among the RP group (141 of 225 [62.7%]) were noninferior to those of the SP group (68 of 190 [35.8%]) (odds ratio [OR], 3.60; 95% CI, 2.12-6.10). Withdrawal did not differ significantly between conditions (proportion of days with a moderate or greater maximum Clinical Opiate Withdrawal Scale score (>12) for RP vs SP: OR, 1.25; 95% CI, 0.62-2.50). Targeted safety events (RP: 12 [5.3%]; SP: 4 [2.1%]) and serious adverse events (RP: 15 [6.7%]; SP: 3 [1.6%]) were infrequent but occurred more often with RP than SP.

**Conclusions and Relevance:**

In this trial, the RP of XR-naltrexone initiation was noninferior to the standard approach and saved time, although it required more intensive medical management and safety monitoring. The results of this trial suggest that rapid initiation could make XR-naltrexone a more viable treatment for patients with OUD.

**Trial Registration:**

ClinicalTrials.gov Identifier: NCT04762537

## Key Points

**Question:**

Can the opioid antagonist injectable extended-release (XR)-naltrexone be started in patients with active opioid use disorder (OUD) with a rapid (5-7 days) procedure?

**Findings:**

In a 6-site stepped wedge cluster randomized clinical trial that included 415 individuals with OUD, more patients successfully initiated XR-naltrexone using the rapid procedure (62.7%) compared with a standard (12-14 days) procedure (35.8%). The rapid procedure was shown to be both noninferior and superior, but had a higher number of safety events and serious adverse events and required more staff attention.

**Meaning:**

The findings of this trial suggest that a rapid approach to withdrawal management should be considered for patients with OUD attempting to start treatment with XR-naltrexone, but more intensive staffing needs may be a barrier to implementation.

## Teaser

This stepped-wedge cluster trial evaluates the effectiveness of a rapid 5- to 7-day initiation of extended-release naltrexone compared with the standard 12- to 14-day procedure among individuals with opioid use disorder.

## Introduction

The opioid epidemic continues to contribute to substantial morbidity and mortality,^1^ with more than 80 000 opioid overdose deaths in the US in 2022.^1,2^ Efficacious, Food and Drug Administration–approved medications for opioid use disorder (MOUD) that promote abstinence and protect against overdose include the full opioid agonist methadone, the partial agonist buprenorphine, and the antagonist naltrexone in an injected extended-release (XR) formulation (XR-naltrexone).^3,4,5^ Extended-release naltrexone may be particularly relevant for marginalized populations that may have less-favorable attitudes toward agonist-based treatments.^6^

A substantial barrier to use of XR-naltrexone is the need to withdraw patients from opioid treatment to avoid precipitated withdrawal.^7^ The XR-naltrexone (Vivitrol; Alkermes Inc) prescribing information recommends a 7- to 10-day opioid-free period before administering XR-naltrexone.^8^ Assuming a typical 3- to 5-day buprenorphine taper for withdrawal from opioid treatment, this commonly used protocol adds up to 10 to 15 days before administering XR-naltrexone. During this extended period, patients are vulnerable to residual opioid withdrawal, cravings, and dropping out of treatment with the subsequent risk of overdose and death.

Procedures have been developed to shorten XR-naltrexone initiation by limiting the use of opioid agonists and using nonopioid medications proactively to manage withdrawal symptoms, together with gradually titrating small doses of oral naltrexone.^9,10,11,12^ In an outpatient single-site trial, this rapid initiation method was superior to the standard approach on the proportion of patients successfully initiating XR-naltrexone.^13^

Improving the rate of XR-naltrexone initiation using the rapid procedure (RP) in community-based treatment settings could be an important step to ensure more patients with OUD have the option of initiating XR-naltrexone, if this is their preferred medication treatment. The NIDA Clinical Trials Network CTN-0097 Surmounting Withdrawal to Initiate Fast Treatment with Naltrexone (SWIFT) trial was conducted to test the effectiveness of the rapid XR-naltrexone initiation procedure compared with usual care at community-based inpatient addiction treatment units.

## Methods

**Figure 1.**
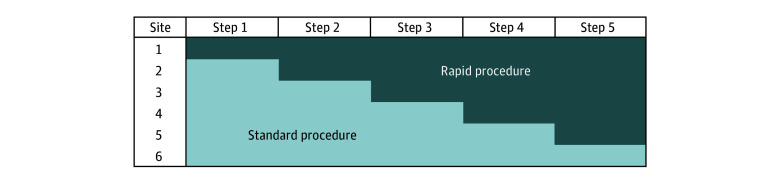
Stepped-Wedge Design in Which Treatment Sites Switched From Standard Procedure for Initiation of Extended-Release (XR)-Naltrexone to the Rapid Procedure Over Successive 14-Week Steps

The SWIFT trial was a 70-week, stepped-wedge, cluster-randomized design trial^14^ conducted at 6 geographically diverse, community inpatient addiction treatment programs in the US. Inpatient sites (American Society of Addiction Medicine level 3.7 or higher) were selected from across the National Institute of Drug Abuse (NIDA) clinical trials network and had a sufficient volume of inpatient admissions with OUD. At the start of the trial and at 14-week intervals, 1 site at a time was randomly assigned to switch from their standard procedure (SP), which followed the Vivitrol prescribing information^8^ to initiate XR-naltrexone, to the RP; the sixth site continued their SP throughout the trial (Figure 1). Randomization was conducted centrally through the Emmes NIDA Data and Statistics Center. SWIFT was approved at all sites by Biomedical Research Alliance of New York, and informed consent was obtained from all participants; financial compensation was provided. Methodologic details of the study were published previously^15^ and are available in the Trial Protocol in Supplement 1. The reported findings follow the Consolidated Standards of Reporting Trials (CONSORT) reporting guideline for randomized studies.

Participants received the initiation procedure (ie, SP or RP) that their program was assigned to at the time of their admission. After the inpatient stay, participants were referred for outpatient addiction treatment according to each site’s SPs and were followed up for 8 outpatient weeks; the second and third XR-naltrexone injections were offered at weeks 4 and 8 after the first injection.

### Participants

Participants were recruited sequentially from patients with OUD seeking inpatient treatment at study sites. Clinical staff met with patients and reviewed MOUD options using a shared decision-making tool (eAppendix 2 in Supplement 2). All participating sites were well versed in the use of agonist-based treatment and encouraged all forms of MOUD. Study participation was only offered to individuals who expressed interest in XR-naltrexone treatment. Participants completed study consent up to 4 days from admission. Eligibility criteria were broad, allowing individuals with stable comorbid psychiatric, medical, and other substance use disorders to enroll.

### XR-Naltrexone Initiation Procedures

The SP consisted of a 3- to 5-day buprenorphine taper followed by a 7- to 10-day opioid-free period before receipt of XR-naltrexone injection (eTable 1 in Supplement 2). Adjunctive medication regimens varied across sites (eTable 2 in Supplement 2).

The RP required approximately 5 to 7 days (eTable 1 in Supplement 2). This included a single day of buprenorphine administration at the minimal dose needed to control withdrawal symptoms. Beginning 2 days after buprenorphine initiation, participants received a 3- to 4-day titration of oral naltrexone, from 0.5 to 6 mg, in 2 divided doses. During this titration, the first daily oral naltrexone dose was given if the participants’ Clinical Opiate Withdrawal Scale (COWS) score was less than 6. The COWS has a score range of 0 to 48, with higher scores indicating more severe withdrawal. The second dose was given 3 to 4 hours later. This dose was skipped if there was a 3-point or more increase in the COWS score after the initial dose to avoid worsening withdrawal. On the third day of naltrexone titration, XR-naltrexone, 380 mg, was administered if the COWS score did not increase after a 6-mg oral naltrexone dose. The naltrexone for the trial was procured by each site from local compounding pharmacies to reflect clinical practice implementation. Nonopioid medications for withdrawal were administered throughout the RP as standing doses. These included clonidine, 0.1 to 0.2 mg, every 4 hours (withheld for low blood pressure or heart rate), clonazepam, 1 mg, every 6 hours (held for oversedation), daily antinausea medication (prochlorperazine, 10 mg, or ondansetron, 8 mg), a sedative agent at bedtime (eg, zolpidem, 5-10 mg; trazodone, 50-100 mg), and nicotine replacement therapy (patch and gum) if needed. Further medications were given as needed. Electrolyte-rich beverages were encouraged, with at least 1 to 2 cups given every 4 hours. Given the potential adverse effects with clonidine and clonazepam (eg, orthostatic hypotension, dizziness, and gait instability), sites were advised to provide education and closely monitor patients for fall risk. Individuals who left the unit before XR-naltrexone initiation were strongly encouraged to begin another form of MOUD by clinical staff. A detailed order set was provided to clinicians (eAppendix 1 in Supplement 2).

Sites were trained on the RP through an implementation facilitation process beginning 8 weeks before the scheduled switch to RP implementation. Expert clinicians on the lead research team served as external facilitators and a local implementation team was formed at each site, consisting of administrators and clinicians who served as champions and local trainers. This team met with the lead research team weekly for 1-hour sessions to work out logistics and for didactic trainings provided by lead team clinicians. After RP implementation, the teams met as needed (at a minimum, every week for the first 4 weeks of implementation). Once sites were comfortable in implementing the RP, site coaching shifted to collaborative meetings that included all sites assigned to the RP. Sites assigned to the SP met as a group with the lead research team every 1 to 3 weeks to review clinical issues.

### Study Assessments

Race and ethnicity data were collected from patient self-report from the NIDA demographic questionnaire to provide information about the participants included in a study and to inform potential generalizability of the results. During the inpatient phase, assessments included daily monitoring of opioid withdrawal and cravings, medications administered, and weekly monitoring of depression and anxiety symptoms. Outpatient phase assessments occurred weekly (weeks 1-4) or every other week (weeks 4-8) and included monitoring of self-reported MOUD use, substance use, opioid cravings and withdrawal, and urine point of care urine drug screen samples (eTable 3 in Supplement 2). In addition to serious adverse events (SAEs), targeted safety events (TSEs) potentially related to detoxification were defined a priori based on previous studies^5,13,16,17^ and systematically collected. These included falls, acute medical complications, and acute changes in mental status or psychiatric symptoms.

### Statistical Analysis

#### Primary and Secondary Outcomes

The primary outcome was dichotomous receipt of the first injection of XR-naltrexone during inpatient status. Secondary outcomes included opioid withdrawal severity, opioid craving, safety events, receipt of second and third XR-naltrexone injections, and opioid use as measured by self-reported days of opioid use and urine drug screen samples. All analyses were performed using SAS, version 9.4 (SAS Institute Inc), and were intention-to-treat, and prespecified in the trial protocol (Supplement 1) and in the trial statistical analysis plan (Supplement 3), apart from the Fisher exact tests for baseline differences, the Fisher exact tests on SAEs and overdose, and the χ^2^ test for follow-up injections, which were added post hoc.

#### Primary Outcome Analysis

The primary outcome analysis was prespecified to test noninferiority, because the rapid approach has a clear pragmatic advantage for patients and was therefore considered preferable even if the 2 approaches were similar in effectiveness. The statistical analysis plan prespecified that, if noninferiority is established (lower bound of the 95% CI of the odds ratio [OR], >0.67), we would establish superiority if the 95% CI is above 1.^18,19^ The analysis used a generalized linear mixed-effects model,^14^ with a logistic link and assuming observations are equally correlated within cluster (site) by treatment group, regardless of time period. The log odds of a participant receiving the first XR-naltrexone injection was modeled as a function of the procedure received (RP vs SP), time (study month or step) to control for secular trends, and a random effect for site to control for nesting of participants within the site. Observations were assumed to be equally correlated within the site (cluster), regardless of time period. Supplemental analyses related to the primary outcome tested for a fixed effect of treatment by time interaction, which, if significant, would indicate differential impact of treatment across time. Sensitivity analyses of the primary outcome were performed to account for different correlation structures.^14,20^ Subgroup analyses were performed to examine intervention effect within subgroups defined by age, sex, and race and ethnicity.

#### Secondary Outcome Analysis

Withdrawal ratings during the initiation phase were analyzed using a mixed-effects model adjusting for initiation procedure, step, site, and inpatient day as fixed effects and participant as a random effect. The Subjective Opiate Withdrawal Scale score was modeled as a continuous measure, while the COWS score was modeled as a binary measure for the presence of at least 1 moderate to severe daily COWS score (maximum score ≥12). Proportions of SAEs, overdose, and TSEs by initiation procedure were compared using the Fisher exact test.

#### Power and Sample Size

The target sample size of 450 participants (15 participants per step ×6 sites ×5 steps) was chosen to provide robust power (88%) to detect noninferiority of RP to SP, assuming a true probability of successful XR-naltrexone initiation of 70% on RP vs 55% on SP and a noninferiority margin of 10% (eg, observed successful XR-naltrexone initiation of 45% on RP vs 55% on SP), corresponding to the lower bound of the 2-sided 95% CI for the OR for RP vs SP above 0.67. The intraclass correlation (ie, the correlation between 2 participants within the same site) was assumed to be 0.14, based on prior data.^21^ The primary outcome and the supportive analysis for the primary outcome used a 1-sided test with 2.5% type I error rate. A similar error rate was used for the test of superiority.

## Results

**Figure 2.**
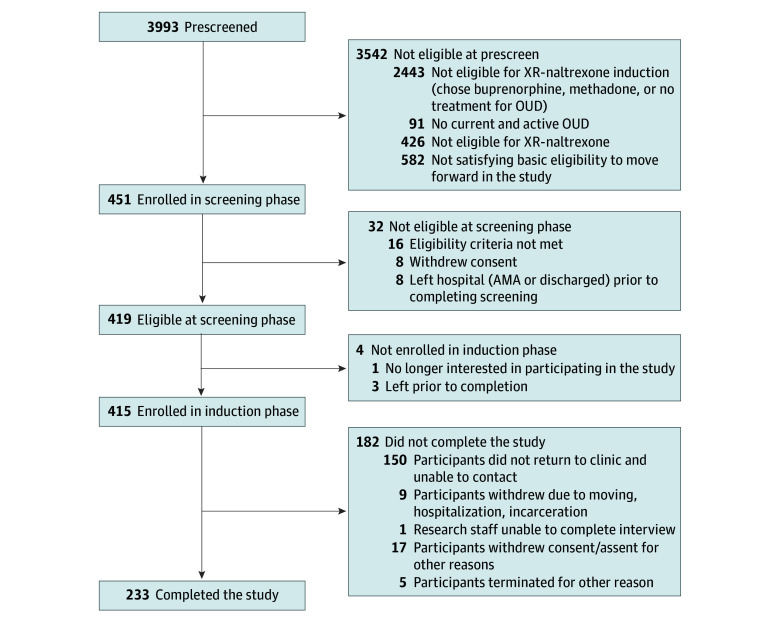
Patient Flowchart AMA indicates against medical advice; OUD, opioid use disorder; XR, extended-release.

Participant recruitment was from March 16, 2021, to July 18, 2022. The last visit was September 21, 2022. At the 6 sites, 3993 patients were admitted for management of OUD. Of those, 2443 did not attempt XR-naltrexone initiation for reasons including 848 preferred buprenorphine, 1085 preferred methadone treatment, and 280 preferred withdrawal management followed by no MOUD (eTable 4 in Supplement 2). A total of 415 participants (10.4%) were attempting to initiate XR-naltrexone and agreed to participate in the trial, with 225 enrolled in sites assigned to the RP and 190 in sites assigned to the SP (Figure 2); 37.3% of participants did not initiate XR-naltrexone. All participants were included in the primary outcome assessment of induction during the inpatient phase; 56% of the participants completed the final outpatient week 8 study assessment.

The recruited participants had a mean (SD) age of 33.6 (8.48) years and consisted of 210 (50.6%) individuals identifying as female sex; male sex, 205 (49.4%); male gender, 187 (45.1%); female gender, 182 (43.9%); and transgender/nonbinary, 2 (0.5%); 5 (1.2%) identified as American Indian or Alaska Native; Asian, 6 (1.4%); Black, 54 (13.0%); Hispanic, 91 (21.9%); White, 290 (69.9%); and multiracial, 22 (5.3%). Demographic and clinical characteristics and comparisons using Fisher exact tests on the differences between arms of the study population are presented in Table 1.

**Table 1.** Summary and Comparison of Baseline Characteristics by Initiation Procedure

Characteristic	No. (%)			*P* value^a^
	Initiation procedure		Total (N = 415)	
	Standard (n = 190)	Rapid (n = 225)		
Age, mean (SD), y	35.1 (9.09)	32.2 (7.69)	33.6 (8.48)	<.001
Sex				
Male	113 (59.5)	92 (40.9)	205 (49.4)	<.001
Female	77 (40.5)	133 (59.1)	210 (50.6)	
Gender				
Male gender	101 (53.2)	86 (38.2)	187 (45.1)	<.001^b^
Female gender	64 (33.7)	118 (52.4)	182 (43.9)	
Transgender/nonbinary	2 (1.1)	0	2 (0.5)	
Heterosexual orientation or straight	146 (76.8)	173 (76.9)	319 (76.9)	.52
Hispanic or Latino ethnicity	38 (20.0)	53 (23.6)	91 (21.9)	.46
Race				
American Indian or Alaska Native	4 (2.1)	1 (0.4)	5 (1.2)	.01^b^
Asian	4 (2.1)	2 (0.9)	6 (1.4)	
Black or African American	28 (14.7)	26 (11.6)	54 (13.0)	
Native Hawaiian or Pacific Islander	1 (0.5)	1 (0.4)	2 (0.5)	
White	115 (60.5)	175 (77.8)	290 (69.9)	
Multiracial	16 (8.4)	6 (2.7)	22 (5.3)	
Education completed				
Less than high school	39 (20.5)	49 (21.8)	88 (21.2)	.56
High school graduate/GED	82 (43.2)	103 (45.8)	185 (44.6)	
Greater than high school	67 (35.3)	67 (29.8)	134 (32.3)	
Unemployed/looking for work	121 (63.7)	140 (62.2)	261 (62.9)	.76
Married/living with partner	31 (16.3)	44 (19.6)	75 (18.1)	.34
Spent the night before admission in an apartment or house	122 (64.2)	152 (67.6)	274 (66.0)	.17
Under criminal justice supervision	20 (10.5)	38 (16.9)	58 (14.0)	.06
Has health insurance	122 (64.2)	85 (37.8)	207 (49.9)	<.001
Substance use history				
Fentanyl-positive at baseline toxicology screening	123 (64.7)	140 (62.2)	263 (63.4)	.60
TLFB substance use at baseline (at least once in the past 30 d)				
Heavy alcohol use (females: ≥3; males: ≥4 drinks/d)	36 (18.9)	39 (17.3)	75 (18.1)	.74
Heroin/fentanyl	141 (74.2)	155 (68.9)	296 (71.3)	.29
Opioid analgesics	45 (23.7)	47 (20.9)	92 (22.2)	.56
Buprenorphine	25 (13.2)	16 (7.1)	41 (9.9)	.05
Methadone	11 (5.8)	3 (1.3)	14 (3.4)	.01
No opioids (heroin/fentanyl, opioid analgesics, buprenorphine, methadone)^c^	2 (1.1)	8 (3.6)	10 (2.4)	.11^d^
Methamphetamine	60 (31.6)	73 (32.4)	133 (32.0)	.73
Other amphetamine	9 (4.7)	10 (4.4)	19 (4.6)	.92
Cocaine	70 (36.8)	61 (27.1)	131 (31.6)	.04
Benzodiazepines	28 (14.7)	64 (28.4)	92 (22.2)	<.001
Cannabis	95 (50.0)	114 (50.7)	209 (50.4)	.71
Ecstasy (MDMA)	18 (9.5)	6 (2.7)	24 (5.8)	.004
Route of last heroin/fentanyl use from TLFB				
Oral	3 (1.6)	1 (0.4)	4 (1.0)	0.03^b^
Nasal	54 (28.4)	66 (29.3)	120 (28.9)	
Smoking	25 (13.2)	12 (5.3)	37 (8.9)	
Injection	56 (29.5)	73 (32.4)	129 (31.1)	
History of lifetime opioid overdose^e^	89 (46.8)	111 (49.3)	200 (48.2)	
No. of lifetime overdoses, mean (SD)	4.5 (6.92)	4.1 (4.59)	4.3 (5.73)	.64
History of taking medication to treat opioid use disorder (any medication)	101 (53.2)	117 (52.0)	218 (52.5)	.81
History of XR-naltrexone monthly injection	26 (13.7)	25 (11.1)	51 (12.3)	.43
History of opioid detoxification attempts	149 (78.4)	166 (73.8)	315 (75.9)	.27
No. of attempts, mean (SD)	4.6 (8.89)	3.7 (6.75)	4.1 (7.80)	.29
Substance use disorder diagnosis				
Alcohol use disorder	67 (35.3)	62 (27.6)	129 (31.1)	.09
Amphetamine use disorder	65 (34.2)	85 (37.8)	150 (36.1)	.45
Cannabis use disorder	75 (39.5)	90 (40.0)	165 (39.8)	.91
Cocaine use disorder	80 (42.1)	67 (29.8)	147 (35.4)	.008
Sedative use disorder	41 (21.6)	65 (28.9)	106 (25.5)	.09
Medical and psychiatric history				
Hepatitis C	36 (18.9)	34 (15.1)	70 (16.9)	.30
Anxiety or panic disorder	101 (53.2)	165 (73.3)	266 (64.1)	<.001
Attention deficit/hyperactivity disorder	43 (22.6)	47 (20.9)	90 (21.7)	.67
Bipolar disorder	32 (16.8)	82 (36.4)	114 (27.5)	<.001
Major depressive disorder	73 (38.4)	84 (37.3)	157 (37.8)	.82
Schizophrenia/other psychotic disorder	10 (5.3)	8 (3.6)	18 (4.3)	.39
Suicidal ideation	22 (11.6)	47 (20.9)	69 (16.6)	.01
Suicidal behavior	19 (10.0)	38 (16.9)	57 (13.7)	.04
Baseline screening for mental health symptoms				
ASRS-5 score ≥14	53 (27.9)	83 (36.9)	136 (32.8)	.05
PCL-5 score ≥31)	76 (40.0)	97 (43.1)	173 (41.7)	.52
Generalized Anxiety Disorder-7 score ≥8)	103 (54.2)	145 (64.4)	248 (59.8)	.03
Patient Health Questionnaire-9 score ≥10	101 (53.2)	141 (62.7)	242 (58.3)	.05
Severity of opioid use disorder				
Mild	1 (0.5)	1 (0.5)	3 (0.5)	.73^e^^,^^f^
Moderate	1 (0.5)	0	1 (0.2)	
Severe	187 (98.9)	223 (99.6)	410 (99.3)	
Severity of alcohol use disorder				
Mild	22 (35.5)	16 (23.9)	38 (29.5)	.35^e^
Moderate	6 (9.7)	7 (10.5)	13 (10.1)	
Severe	34 (54.8)	44 (65.7)	78 (60.5)	
Severity of amphetamine use disorder				
Mild	20 (23.5)	14 (21.5)	34 (22.7)	.73^f^
Moderate	4 (4.7)	5 (7.7)	9 (6.0)	
Severe	61 (71.8)	46 (70.7)	107 (71.3)	
Severity of cannabis use disorder				
Mild	42 (46.7)	29 (38.7)	71 (43.0)	.20^e^
Moderate	13 (14.4)	19 (25.3)	32 (19.4)	
Severe	35 (38.9)	27 (36.0)	62 (37.6)	
Severity of cocaine use disorder				
Mild	22 (32.8)	19 (23.8)	41 (27.9)	.29^e^
Moderate	5 (7.5)	11 (13.8)	16 (10.9)	
Severe	40 (59.7)	50 (62.5)	90 (61.2)	
Severity of sedative use disorder				
Mild	19 (29.2)	10 (24.3)	29 (27.4)	.70^e^
Moderate	9 (13.9)	8 (19.5)	17 (16.0)	
Severe	37 (56.9)	23 (56.1)	60 (56.6)	

Abbreviations: ADHD, attention deficit/hyperactivity disorder; ASRS-5, Adult ADHD Self-Report Screening Scale for *Diagnostic and Statistical Manual of Mental Disorders, Fifth Edition*; GED, general educational development; MDMA, 3,4-methylenedioxymethamphetamine; PCL-5, PTSD Checklist for *Diagnostic and Statistical Manual of Mental Disorders, Fifth Edition*; PTSD, posttraumatic stress disorder; TLFB, timeline follow-back; XR, extended release.

^a^ Continuous variables were compared using *t* test between the 2 treatment groups; categorical variables were compared using χ^2^ test between the 2 treatment groups. *P* value was calculated excluding the missing values due to missing, not sure, don’t know, and refused to answer.

^b^ Pearson exact χ^2^ test was used when 20% of cells have expected cell counts less than 5.

^c^ All participants met criteria for opioid use disorder, but some denied recent use on TLFB.

^d^ Fisher exact test was used when 20% of cells have expected cell counts less than 5.

^e^ One participant in the standard procedure group had a history of lifetime opioid overdose but did not provide the number of lifetime overdoses.

^f^ For comparing severity of substance use disorder, *P* values were calculated by removing the participants who were classified as none.

### Primary Outcome

In the RP, 141 of 225 individuals (62.7%) received XR-naltrexone compared with 68 of 190 (35.8%) in the SP (OR, 3.60; 95% CI, 2.12-6.10). The noninferiority test determined that, since the lower bound of the OR exceeded 0.67, noninferiority was demonstrated. Secondarily, it was determined that, since the lower bound of the OR exceeded 1.0, it was consistent with superiority (*P* < .001). The fixed effect of step was not significant (*F*_4,24_ = 1.112; *P* = .37), indicating no significant impact of the step on the primary outcome. Subgroup analyses of sex, age, and race and ethnicity showed no statistically significant interaction terms, implying the effects of initiation procedure did not differ across subgroups. The mean (SD) times to injection from admission were 7.0 (1.42) days (range, 4-12 days) with the RP and 14.5 (3.57) days (range, 7-23 days) with the SP.

For the 206 participants who did not receive a first dose of XR-naltrexone, the most common reasons were chose to leave the inpatient unit early (RP: 52 [61.9%]; SP: 86 [70.5%]), decided to begin buprenorphine or methadone (RP: 18 [21.4%]; SP: 25 [20.5%]), and withdrawal symptoms too uncomfortable (RP: 12 [14.3%]; SP: 6 [4.9%).

### Secondary Outcomes

In the RP, 31.7% (69 of 225) individuals received the second injection and 21.3% (48 of 225) received the third injection. In the SP, 23.7% (45 of 190) received the second injection and 16.8% (32 of 190) received the third injection. Receipt of the second (*P* = .11) or third (*P* = .25) injection in those who were initiated on XR-naltrexone did not differ significantly between the groups.

**Figure 3.**
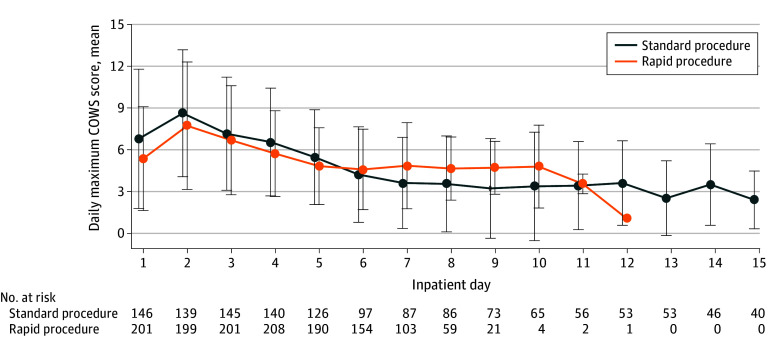
Mean (SD) Daily Maximum Clinical Opiate Withdrawal Scale (COWS) Score During the First 15 Days of the Standard and Rapid Procedures

The mean (SD) daily maximum COWS scores over the first 15 days of the trial are plotted in Figure 3. The proportion of days with a moderate or greater maximum COWS score (≥12) did not differ significantly between the SP and RP groups after controlling for site and day of admission (OR, 1.25; 95% CI, 0.62-2.50; *P* = .54). Day from admission was associated with a lower frequency of elevated COWS scores (OR, 0.74; 95% CI, 0.69-0.79; *P* < .001). The mean maximum Subjective Opiate Withdrawal Scale score also did not differ significantly after controlling for site and day of admission (mean difference, 1.79; 95% CI, −2.67 to 6.25; *P* = .43).

### Safety Outcomes

Safety events occurred at low frequencies overall (Table 2) but were more frequent in the RP than the SP group, particularly for TSEs; during the initiation/inpatient period, this was related to more falls and medical complications, while during the 2-month outpatient follow-up period, this was related to more falls, medical complications, and psychiatric or mental status problems. The frequency of TSEs was not significantly different between groups during the initiation period (RP: 12 [5.3%]; SP: 4 [2.1%]; *P* = .12; Fisher exact test), but was significantly greater in the RP group during the outpatient period (RP: 15 [6.7%]; SP: 3 [1.6%]; *P* = .01; Fisher exact test). During the 8-week follow-up period, 3 participants (1.3%) in the RP group and 6 (3.2%) in the SP group reported nonfatal overdose.

**Table 2.** SAEs and TSEs During the Inpatient Initiation and Outpatient Postinitiation Periods for RP and SP

Event	No. (%)			Fisher *P* value
	Initiation procedure		Total (N = 415)	
	Standard (n = 190)	Rapid (n = 225)		
SAEs				
No. of participants with at least 1 SAE during initiation phase (inpatient)	2 (1.1)	3 (1.3)		
Overdose	0	1	1	>.99
Suicidal ideation/attempt	0	1	1	
Medical complications (decreased level of consciousness, infectious ileitis, seizures)	2	1	3	
Other	0	0	0	
No. of participants with at least 1 SAE during postinitiation phase (2-mo outpatient follow up)	4 (2.1)	10 (4.4)	14 (3.4)	
Overdose	1	2	3	.28
Suicidal ideation/attempt	0	2	2	
Medical complications^a^	3	3	6	
Other	0	3	3	
TSEs				
No. of participants with TSEs during initiation phase (inpatient)	4 (2.1)	12 (5.3)	16 (3.9)	
Fall event	0	4	4	.12
Acute change in mental status	1^b^	0	1	
Acute medical complication likely exacerbated by the stress of withdrawal	3; Seizures during withdrawal (n = 1)^b^; precipitated withdrawal (n = 2)	8; Vomiting (n = 5); precipitated withdrawal (n = 2); wheezing/shortness of breath (n = 1)	11	
Acute psychiatric symptoms	0	0	0	
No. of participants with ≥1 TSE during postinitiation phase (2-mo outpatient follow-up)^c^	3 (1.6)	15 (6.7)	18 (4.3)	
Fall event	1	3	4	.01
Acute change in mental status	0	1	1	
Acute medical complication likely exacerbated by the stress of withdrawal	2; Precipitated withdrawal (n = 1), kidney infection (n = 1)	6; Nausea/vomiting (n = 1), anxiety/panic attack (n = 1), hypertension (n = 2), hypotension (n = 1), asthma (n = 1)	8	
Acute psychiatric symptoms	0	8; Suicide attempt (n = 1),^b^ suicidal ideations (n = 3), agitation (n = 1), anxiety (n = 1), stimulant psychosis (n = 1), drug-induced hallucinations (n = 1)	8	
No. of participants with a nonfatal overdose postinitiation phase^d^	6 (3.2)	3 (1.3)	9 (2.2)	.31

Abbreviations: SAE, serious adverse event; TSE, targeted safety event.

^a^ Hypokalemia, chronic obstructive pulmonary disease, sepsis, COVID-19, pneumonia, and neck abscess.

^b^ TSE also classified as SAE.

^c^ Some participants experienced 2 or more of the listed complications/symptoms.

^d^ Based on response to the Overdose Questionnaire.

## Discussion

This stepped-wedge, site-level randomized clinical trial conducted across 6 community inpatient addiction treatment programs showed that an RP for initiation of XR-naltrexone was noninferior to an SP and led to a substantially greater number of patients receiving a first injection (62.7% vs 35.8%). The trial also highlighted the difficulty in starting XR-naltrexone, with 37.3% of participants not initiating XR-naltrexone treatment even with the more effective RP. Nevertheless, our study supports previous findings^7^ that a small but sizable number of individuals prefer treatment with an opioid antagonist when offered all 3 medication options and demonstrates a faster, more successful pathway to XR-naltrexone initiation that should be considered.

The RP uses aggressive oral hydration and additional nonopioid medications via standing doses. This appears to control withdrawal symptoms, as opioid withdrawal symptoms were low on average and did not differ significantly between the RP and SP (Figure 3). Safety events related to withdrawal procedures were infrequent overall but were more common with the RP (5.3% vs 2.1%) (Table 2). The significantly greater incidence of TSEs, in particular psychiatric events, after induction, may relate to the RP placing more stress on the cardiovascular and nervous systems, although greater baseline psychiatric severity may have played a role. The RP therefore requires more intense monitoring and clinical expertise, and this may not be feasible in some settings.

Although not the primary focus of the study, the results highlight the problem of adherence in community-based outpatient follow-up (less than half of patients who initiated treatment received 2 subsequent monthly injections). This is consistent with observational studies reporting that XR-naltrexone has low rates of retention and poorer outcomes compared with buprenorphine or methadone,^22,23^ although controlled studies have not shown a substantial difference in retention.^21,24^ In addition, only 56% of participants completed the final study assessment and the trial was not powered to detect long-term differences in treatment outcomes (ie, relapse, overdose, and improvement in quality of life). More research is needed on how to prevent dropout from XR-naltrexone after it has been initiated, with more aggressive research follow-up to improve trial retention.

### Limitations

SWIFT was conceptualized as a hybrid effectiveness-implementation trial in which trial procedures were implemented by the regular clinical staff members of the participating community-based inpatient treatment programs. However, site-based research teams developed working relationships with the clinical staff and contributed in varying degrees to patient care, limiting generalizability to non–research-involved programs. In addition, although all 6 sites made arrangements with local compounding pharmacies to purchase low-dose naltrexone at low cost, low-dose naltrexone is not on the formulary at most hospitals and therefore represents a barrier for widespread implementation. In addition, clinical teams were intensively coached by expert lead team clinicians, which may not be feasible on a large scale. More research is needed on sustainability, feasibility, and health economic aspects of this intervention. Substantially fewer days used with the RP is cost saving, but the resources needed for more intensive monitoring may outweigh these savings.

## Conclusions

This cluster trial demonstrated that an RP for initiating XR-naltrexone consisting of minimal buprenorphine, use of higher doses of nonopioid medications for opioid withdrawal, and titration of low doses of oral naltrexone is feasible, relatively safe, and noninferior, compared with the SP recommended in the XR-naltrexone prescribing information. The RP requires close medical monitoring. Due to its shorter duration, the RP may be cost-effective and a better fit within constraints on the duration of inpatient stays imposed by third-party payers.

It is noteworthy that only 10.4% of all patients entering treatment chose to attempt XR-naltrexone initiation and even in the RP 37.3% of participants did not initiate XR-naltrexone. In future studies, the implementation strategies used in the present trial could be broadened to encompass initiation of buprenorphine and methadone, addressing the larger goal of maximizing the proportion of patients with OUD admitted to inpatient or residential treatment who emerge stabilized on MOUD.

## Notes

Supplement 1.
Trial Protocol

Supplement 2. **eTable 1.** Medication Regimen by Induction Procedure **eTable 2.** Summary of Adjunctive Medications by Induction Procedure **eTable 3.** Study Assessments for Participants (RP and SP) **eTable 4.** Reasons for Not Attempting XR-NTX Induction **eAppendix 1.** Rapid Procedure Example Order Set **eAppendix 2.** Shared Decision-Making Tool for Medications for Opioid Use Disorder

Supplement 3.
Statistical Analysis Plan

Supplement 4.
Data Sharing Statement
