# Supplementary material for: Rapid Initiation of Injection Naltrexone for Opioid Use Disorder: A Stepped-Wedge Cluster Randomized Clinical Trial
Source: JAMA Netw Open. 2024 May 8;7(5):e249744. doi: 10.1001/jamanetworkopen.2024.9744 (PMC11079685; doi:10.1001/jamanetworkopen.2024.9744)
Supplement: Supplement 1. — Trial Protocol [file jamanetwopen-e249744-s001.pdf]

NIDA CTN Protocol 0097

Surmounting Withdrawal to Initiate Fast Treatment with Naltrexone:

Improving the Real-World Effectiveness of Injection Naltrexone for Opioid Use Disorder

(SWIFT)

Lead Investigator: Adam Bisaga, MD

Funded by: National Institute on Drug Abuse (NIDA)

Version 3.0

October 13, 2021

CONFIDENTIAL

**Lead Investigator (LI):**	**Adam Bisaga, MD**<br>Greater New York Node<br>NYS Psychiatric Institute<br>Columbia University, Department of Psychiatry
**Co-LI:**	**Edward V. Nunes, MD**<br>Greater New York Node<br>NYS Psychiatric Institute<br>Columbia University, Department of Psychiatry
	**John Rotrosen, MD**<br>Greater New York Node<br>New York University, School of Medicine
**CCTN Scientific Officer:**	**Udi Ghitza, PhD**<br>National Institute on Drug Abuse
**Data and Statistics Center (DSC):**	**Paul Van Veldhuisen, PhD**<br>DSC Principal Investigator<br>Emmes
**Clinical Coordinating Center (CCC):**	**Eve Jelstrom, CRNA, MBA**<br>CCC Principal Investigator<br>Emmes

CONFIDENTIALITY STATEMENT

This document is confidential communication. Acceptance of this document constitutes agreement by the recipient that no unpublished information contained herein will be published or disclosed without prior approval of the Lead Investigator or other participating study leadership and as consistent with the NIDA terms of award.

TABLE OF CONTENTS

1.0 LIST OF ABBREVIATIONS ... 8

2.0 STUDY SYNOPSIS ... 11

2.1 STUDY OBJECTIVES ... 11
2.2 STUDY DESIGN AND OUTCOMES ... 11
2.3 SAMPLE SIZE AND STUDY POPULATION ... 12
2.4 TREATMENT/ASSESSMENT/INTERVENTION AND DURATION ... 13
2.5 SAFETY REPORTING ... 13
2.6 ANALYSES ... 13

3.0 STUDY SCHEMA ... 14

3.1 SCHEMATIC OF STUDY DESIGN ... 14
3.1.1 Table 1. Schematic of study design with six study sites and five steps, each lasting 14 weeks ... 14
3.1.2 Figure 1. Schematic of the study phases and participant flow ... 14
3.2 KEY RESEARCH SITE ROLES ... 15

4.0 INTRODUCTION ... 16

4.1 BACKGROUND AND SIGNIFICANCE TO THE FIELD ... 16
4.1.1 Effectiveness of XR-NTX to Prevent Relapse Following Detoxification in OUD ... 16
4.1.2 Addressing the Naltrexone Induction Hurdle ... 16
4.1.3 Changing Practice for Inpatient OUD Treatment ... 18
4.1.4 Study Design ... 18
4.1.5 Duration of XR-NTX Treatment ... 20
4.1.6 Evaluation of Implementation Parameters ... 21
4.1.7 Implementation Facilitation Manual Development Process ... 21
4.2 INNOVATION ... 22
4.3 SUITABILITY, FEASIBILITY, AND SUSTAINABILITY FOR THE CTN SITES ... 23
4.4 PUBLIC HEALTH IMPACT SIGNIFICANCE TO THE FIELD ... 23

5.0 OBJECTIVES ... 24

5.1 PRIMARY OBJECTIVE ... 24
5.2 SECONDARY OBJECTIVE(S) ... 24
5.3 EXPLORATORY OBJECTIVE(S) ... 24
5.4 IMPLEMENTATION OBJECTIVES ... 25

6.0 STUDY DESIGN ... 26

6.1 OVERVIEW OF STUDY DESIGN ... 26
6.1.0 Overview ... 26
6.1.1 Patient-Participants ... 26
6.1.2 Random Site Selection and Treatment Assignment ... 26
6.1.3 Training in SP and RP procedures ... 27
6.2 IMPLEMENTATION FACILITATION ... 29
6.2.0 Initial Elements Included in Implementation Facilitation ... 30
6.2.1 Iterative Development of the Implementation Facilitation Package ... 31
6.3 DURATION OF STUDY AND VISIT SCHEDULE ... 32
6.4 STUDY TIMELINE ... 33
6.4.0 Table 2: Study Timeline ... 33

7.0 OUTCOME MEASURES 34

7.1 PRIMARY OUTCOME MEASURE 34
7.2 SECONDARY OUTCOME MEASURE(S) 34
7.2.1 Table 3: Secondary Clinical Outcomes, Outcome Measures and Hypotheses 34
7.3 IMPLEMENTATION PROCESS AND OUTCOME MEASURES 35
7.3.1 Identifying Facilitators and Barriers to Implementation Success 35
7.3.2 Table 4: Implementation Measures Organized by the Consolidated Framework for Implementation Research Constructs 35
7.3.3 Implementation Related Outcome Measures 37
7.3.4 Table 5: Implementation Outcome Domain 37
7.3.5 Integration Process for Iterative Improvement of Implementation Facilitation 37

8.0 STUDY POPULATION 39

8.1 PARTICIPANT INCLUSION CRITERIA 39
8.2 PARTICIPANT EXCLUSION CRITERIA 39
8.3 SPECIAL POPULATIONS TO CONSIDER 40
8.4 STRATEGIES FOR RECRUITMENT AND RETENTION 40

9.0 SITE SELECTION 41

9.1 NUMBER OF SITES 41
9.2 SITE ELIGIBILITY 41
9.3 RATIONALE FOR SITE SELECTION 42

10.0 STUDY PROCEDURES 43

10.1 PRE-SCREENING, CONSENT AND SCREENING (PHASE 1) 43
10.1.1 Informed Consent Procedures for Participants 43
10.1.2 HIPAA Authorization and Medical Record Release Forms 44
10.1.3 Screening/Baseline Visit 44
10.2 TREATMENT INITIATION 44
10.3 RANDOMIZATION 44
10.4 INPATIENT TREATMENT/INTERVENTION (PHASE 2) 45
10.4.1 Standard Induction Procedure (SP) 45
10.4.2 Table 6A. Standard Induction Procedures 45
10.4.3 Rapid Induction Procedures (RP) 45
10.4.4 Table 6B. Rapid Induction Procedure Guidelines 46
10.5 POST-INDUCTION TREATMENT (PHASE 3) 47
10.5.1 Psychosocial Counseling 47
10.5.2 Medical Management 47
10.5.3 Rapid Procedure: Adjunctive Medication Taper 48
10.5.4 XR-NTX Injections 48
10.5.5 Handling of XR-NTX Induction Procedure Intolerance 48
10.5.6 Handling of Missed XR-NTX Doses, Lapses, and Relapses 49
10.5.7 XR-NTX Discontinuation Post-Induction 49
10.5.8 Ancillary Medications 49
10.5.9 Criminal Justice Involvement 50
10.6 END OF STUDY 50
10.7 DISPOSITION AND REFERRAL (PHASE 4) 50
10.8 STUDY INTERVENTION ADHERENCE 50
10.9 PREMATURE WITHDRAWAL OF PARTICIPANTS 50
10.10 STUDY TERMINATION 51
10.11 BLINDING 51
10.12 PARTICIPANT REIMBURSEMENT 52

10.13 RETENTION PLAN 52

11.0 MEDICATION PACKAGING/HANDLING/STORAGE / ACCOUNTABILITY 53

11.1 STUDY MEDICATION SOURCES 53
11.2 STUDY MEDICATION MANAGEMENT 53
11.3 MEDICATION ACCOUNTABILITY RECORDS 53
11.4 DISPENSING OF STUDY MEDICATION 53
11.5 STUDY MEDICATION STORAGE 53
11.6 USED/UNUSED MEDICATION 54
11.7 LOST MEDICATION 54
11.8 MEDICATION PACKAGING 54

12.0 STUDY ASSESSMENTS 55

12.1 GENERAL 55
12.1.1 Locator Form 55
12.1.2 Prisoner Status Assessment 55
12.1.3 PhenX Tier 1 and Demographics 55
12.1.4 Motivation Scale 55
12.1.5 Patient-Reported Outcomes Measurement Information System (PROMIS) 56
12.1.6 End of Medication Treatment Form 56
12.1.7 Study Completion Form 56
12.1.8 Treatment Satisfaction Survey 56
12.2 MEASURES OF PRIMARY AND SECONDARY OUTCOMES 57
12.2.1 End of Induction Form 57
12.2.2 The Subjective Opioid Withdrawal Scale (SOWS) 57
12.2.3 Clinical Opiate Withdrawal Scale (COWS) 57
12.2.4 Visual Analogue Scales (VAS) 57
12.2.5 Timeline Followback (TLFB) 57
12.2.6 Urine Drug Screen (UDS) 58
12.2.7 Patient Health Questionnaire (PHQ-9) 58
12.2.8 Low Dose Naltrexone Titration 58
12.2.9 XR-NTX Administration 58
12.3 SAFETY AND MEDICAL 58
12.3.1 Medical and Psychiatric History 58
12.3.2 PTSD Checklist for DSM-5 (PCL-5) 59
12.3.3 Adult ADHD Self-Report Screening Scale for DSM-5 (ASRS-5) 59
12.3.4 Overdose Questionnaire 59
12.3.5 Concomitant Medications 59
12.3.6 Death 59
12.3.7 HIV and Hepatitis Self-Report Assessments 59
12.3.8 DSM-5 Criteria 59
12.3.9 General Anxiety Disorder-7 59
12.3.10 PHQ-Panic Disorder 60
12.3.11 Pregnancy, Birth Control, and Breastfeeding Assessment 60
12.3.12 Mental Health Assessment 60
12.3.13 Adverse Events (AEs) and Serious Adverse Events (SAEs) 60
12.4 IMPLEMENTATION RELATED OUTCOMES 61
12.4.1 Quantitative Data Collection and Analysis 61
12.5 QUALITATIVE DATA COLLECTION AND ANALYSIS 62
12.5.1 Verbal Consent for Structured Participant Interviews 62
12.5.2 Conduct of Structured Interviews 63
12.6 IMPLEMENTATION PROCESS OUTCOMES MEASURES 63
12.6.1 Fidelity to Clinical Procedures 63

12.6.2 Coaching Progress Notes ... 64
12.6.3 Site Needs Assessment and Site Mapping Progress Notes ... 64
12.6.4 Internal Implementation Team Meeting Notes ... 64
12.6.5 Fidelity to Implementation Procedures Checklist ... 65
12.6.6 Treatment Utilization Measures ... 65
12.7 OTHER ASSESSMENTS ... 65
12.7.1 Fagerström Test for Nicotine Dependence (FTND) ... 65
12.7.2 NIDA Cannabis Assessment ... 65
12.7.3 Daily Medication Administration Log ... 65
12.7.4 Non-Study Medical and Other Services (NMS) ... 65
12.7.5 Oral Fluid Collection for Genetic Sample and Family Origin ... 66
12.7.6 Table 7: Study Assessments ... 67
12.7.7 Table 7a: Implementation Assessments ... 70

13.0 GENETIC SAMPLING ... 71

14.0 TRAINING REQUIREMENTS ... 72

14.1 OVERALL ... 72

15.0 CONCOMITANT THERAPY/INTERVENTION ... 73

15.1 GENERAL ... 73
15.2 MEDICATIONS PROHIBITED/ALLOWED DURING TRIAL ... 73

16.0 STATISTICAL DESIGN AND ANALYSES ... 74

16.1 GENERAL DESIGN ... 74
16.1.1 Study Hypothesis ... 74
16.1.2 Primary and Secondary Outcomes (Endpoints) ... 74
16.1.3 Study Design ... 74
16.1.4 Recruitment ... 74
16.1.5 Randomization and Factors for Stratification ... 74
16.2 RATIONALE FOR SAMPLE SIZE AND STATISTICAL POWER ... 75
16.2.1 Introduction ... 75
16.2.2 Table 8: Design Pattern Matrix ... 75
16.2.3 Values of Parameters Underlying Power Simulations ... 76
16.2.4 Power Curve ... 78
16.2.5 Power in Unforeseen Circumstances ... 79
16.2.6 Conclusion ... 82
16.3 STATISTICAL METHODS FOR PRIMARY AND SECONDARY OUTCOMES ... 82
16.3.1 Primary Outcome Analysis ... 82
16.3.2 Secondary Outcome Analysis: ... 83
16.4 IMPLEMENTATION OUTCOME ANALYSIS ... 83
16.5 SIGNIFICANCE TESTING ... 83
16.6 INTERIM ANALYSIS ... 83
16.7 EXPLORATORY ANALYSIS ... 83
16.8 PREDICTION MODELS ... 84
16.9 MISSING DATA AND DROPOUTS ... 84
16.10 DEMOGRAPHIC AND BASELINE CHARACTERISTICS ... 84
16.11 SAFETY ANALYSIS ... 84

17.0 REGULATORY COMPLIANCE, REPORTING AND MONITORING ... 86

17.1 STATEMENT OF COMPLIANCE ... 86
17.2 INSTITUTIONAL REVIEW BOARD APPROVAL ... 86
17.3 INFORMED CONSENT ... 86

17.4 Quality Assurance Monitoring 88
17.5 Participant and Data Confidentiality 88
17.5.1 Certificate of Confidentiality 89
17.5.2 Health Insurance Portability and Accountability Act (HIPAA) 89
17.6 Investigator Assurances 89
17.6.1 Financial Disclosure/Conflict of Interest 89
17.7 Clinical Monitoring 89
17.8 Inclusion of Women and Minorities 90
17.9 Prisoner Certification 90
17.10 Regulatory Files 90
17.11 Records Retention and Requirements 91
17.12 Reporting to Sponsor 91
17.13 Audits 91
17.14 Study Documentation 91
17.15 Protocol Deviations 92
17.16 Safety Monitoring 92
17.16.1 Data and Safety Monitoring Board (DSMB) 93
17.16.2 Safety Monitor/Medical Monitor 93
17.16.3 Safety Events 93
17.16.4 Serious Adverse Events (SAEs) 94
17.16.5 Known Potential Toxicities of Study Medication/Intervention 94

18.0 DATA MANAGEMENT 95

18.1 Design and Development 95
18.2 Site Responsibilities 95
18.3 Data Center Responsibilities 95
18.4 Data Collection 95
18.5 Data Acquisition and Entry 95
18.6 Data Editing 96
18.7 Data Transfer/Lock 96
18.8 Data Training 96
18.9 Data Quality Assurance 96

19.0 PUBLIC ACCESS AND DATA SHARING PLAN 97

20.0 PROTOCOL SIGNATURE PAGE 98

21.0 REFERENCES 99

22.0 APPENDIX A: ADVERSE EVENT REPORTING AND PROCEDURES 103

22.1 Definition of Adverse Events and Serious Adverse Events 103
22.2 Definition of Expectedness 103
22.3 Pregnancy 104
22.4 Medical and Psychiatric History 104
22.5 Site's Role in Eliciting and Reporting Safety Events 104
22.6 Site's Role in Assessing Severity and Causality of Adverse Events 105
22.7 Guidelines for Assessing Severity 105
22.8 Guidelines for Determining Causality 105
22.9 Site's Role in Monitoring Safety Events 105
22.10 Sponsor's Role in Safety Management Procedures of AEs/SAEs 105
22.11 Regulatory Reporting for a non-IND study 106
22.11.1 Reporting to the Data and Safety Monitoring Board 106
22.12 Participant Withdrawal 106
22.13 Adverse Event Reporting (Chart) 107

23.0 APPENDIX B: DATA AND SAFETY MONITORING PLAN 108

1.0 LIST OF ABBREVIATIONS

Abbreviation	Definition
AE	Adverse Event
BMI	Body Mass Index
BP	Blood Pressure
BUP	Buprenorphine
BUP-NX	Buprenorphine-Naloxone (Suboxone®)
CAP	College of American Pathologists
CCC	Clinical Coordinating Center
CCTN	Center for the Clinical Trials Network
CFIR	Consolidated Framework for Implementation
CFR	Code of Federal Regulations
CLIA	Clinical Laboratory Improvement Amendment of 1988
CNS	Central Nervous System
CoC	Certificate of Confidentiality
COWS	Clinical Opiate Withdrawal Scale
CRF	Case Report Form
CTN	Clinical Trials Network
CTP	Community Treatment Program
DEA	Drug Enforcement Agency
DSC	Data and Statistics Center
DSM-5	Diagnostic and Statistical Manual of Mental Disorders Fifth Edition
DSMB	Data and Safety Monitoring Board
eCRF	Electronic Case Report Form
EDC	Electronic Data Capture
EMR	Electronic Medical Record
EOS	End of Study
EMT	End of Medication Treatment
ERC	Ethics Review Committee
FDA	Food and Drug Administration
FTND	Fagerström Test for Nicotine Dependence
FWA	Federalwide Assurance
GAD-7	Generalized Anxiety Disorder Screener
GCP	Good Clinical Practice
HCV	Hepatitis C Virus
HHS	Department of Health and Human Services
HIPAA	Health Insurance Portability and Accountability Act
HIV	Human Immunodeficiency Virus

Abbreviation	Definition
HR	Heart Rate
HSP	Human Subjects Protection
ICC	Intraclass Correlation Coefficient
IF	Implementation Facilitation
IND	Investigational New Drug
IRB	Institutional Review Board
IM	Intramuscular
ITT	Intent-to-Treat
IV	Intravenous
LI	Lead Investigator
MD	Medical Doctor
MDMA	Methylenedioxymethamphetamine (Ecstasy)
MDD	Maximum Daily Dose
MedDRA	The Medical Dictionary for Regulatory Activities
Mg	Milligrams
mL	Milliliters
MM	Medical Management
MOP	Manual of Operations
MOUD	Medications for Opioid Use Disorder
NDA	New Drug Application
NIAAA	National Institute on Alcohol Abuse and Alcoholism
NIDA	National Institute on Drug Abuse
NIH	National Institutes of Health
NMS	Non-Study Medical and Other Services
NP	Nurse Practitioner
NTX	Naltrexone
NX	Naloxone
OUD	Opioid Use Disorder
OHRP	Office for Human Research Protections
ORCA	Organizational Readiness to Change Assessment
PO	per os (by mouth)
PA	Physician Assistant
PCL-5	PTSD Checklist for DSM-5
PHQ-9	Patient Health Questionnaire -9
PI	Principal Investigator
PLG	Polylactide-co-glycolide
QA	Quality Assurance
QALYs	Quality-Adjusted Life Year Estimates
RAP-C	Research Advisory Panel of California

Abbreviation	Definition
RP	Rapid Procedure
RRTC	Regional Research and Training Center
SAE	Serious Adverse Event
SAMHSA	Substance Abuse and Mental Health Services Administration
SC	Subcutaneous
SOWS	Subjective Opiate Withdrawal Scale
SP	Standard Procedure
SW	Stepped-Wedge
TAU	Treatment as Usual
TLFB	Timeline Followback
TSE	Targeted Safety Event
UDS	Urine Drug Screen
VA	Veterans Administration
VAS	Visual Analog Scale
XR-NTX	Extended-Release Naltrexone (Vivitrol®)

2.0 STUDY SYNOPSIS

2.1 Study Objectives

The overarching objective of the SWIFT trial (CTN-0097) is to foster widespread adoption of a regimen for rapid initiation of treatment with extended-release injection naltrexone (XR-NTX) at inpatient or residential Community Treatment Programs (CTPs). If widely adopted, such a regimen would have a substantial public health impact by expanding medication treatment options offered to patients with OUD to include XR-NTX.

In multi-site, randomized (subject level) trials, XR-NTX, once initiated, has been found to have similar effectiveness to sublingual buprenorphine on clinical outcomes of retention in treatment and abstinence from opioids. However, initiation of naltrexone often involves a significant (up to 2 weeks) delay, which is a clinical hurdle that impedes the widespread adoption of XR-NTX as a treatment option. Initiation of naltrexone in patients actively using opioids requires that a patient be detoxified first, and the official prescribing information for XR-NTX recommends an additional 7- to 10-day waiting period after last dose of opioid before administering XR-NTX. This standard initiation regimen, involving a brief period of agonist, usually buprenorphine, taper followed by a 7- to 10-day waiting period, takes approximately two weeks. During this time, patients are vulnerable to drop out and relapse; further, this waiting period is problematic in the face of funding restrictions on the duration of inpatient stays. In a single-site randomized trial, a Rapid naltrexone induction method utilizing minimal buprenorphine, non-opioid medications to treat withdrawal symptoms, and upward titration of oral naltrexone starting with small doses, XR-NTX initiation was accomplished in 5 to 7 days and was found superior to the standard 14-day approach on the proportion of patients initiating XR-NTX.

The primary goal of this hybrid effectiveness-implementation study is to determine whether the Rapid method of initiating treatment with XR-NTX is non-inferior to a standard method on the primary effectiveness outcome of successful initiation of XR-NTX (receiving the first injection while inpatient) when implemented at community-based inpatient or residential programs. Secondary objectives include comparing rapid versus standard method of XR-NTX initiation on: time from admission to first dose of XR-NTX while inpatient and time to dropout, craving, withdrawal severity, retention, abstinence, and safety measures, as measured during the inpatient initiation process and the first two months post XR-NTX induction. Other exploratory outcomes include predictors of initiation success, economic analyses, engagement with medical visits and receipt of MOUD after discharge from the inpatient unit. The implementation goal is to operationalize an implementation facilitation strategy that will be used to train clinical sites on the XR-NTX initiation method, to capture fidelity to the rapid induction process, and to study barriers and facilitators to implementation and refine the implementation facilitation strategy accordingly.

2.2 Study Design and Outcomes

The proposed study is an open-label, multi-center, stepped-wedge cluster randomized trial. It will be conducted in 6 CTN-affiliated CTPs that are able to provide inpatient or residential detoxification services and have the capacity for an ongoing outpatient treatment with XR-NTX.

As part of the stepped-wedge design proposed for this trial, one site, randomly chosen, will start implementing the Rapid Procedures and will remain in this arm for the rest of the study.

The next 4 sites randomly chosen will first implement the control procedure (Standard, 13-day procedure; SP), to establish the within-site comparison condition, and then at selected staggered time-points (steps) will switch to implementing only the experimental (Rapid, 5-7-day procedure; RP), and the 6th site (after 5 sites have been randomized to RP) will remain in the SP arm throughout the whole duration of the study. Implementation of RP at study sites will be staggered by 14 weeks and the order in which sites will cross-over from SP to RP will be randomly chosen.

The primary effectiveness objective of the study is to determine whether the novel RP of initiating treatment with XR-NTX is non-inferior to the SP induction method as implemented in community treatment programs. The primary outcome measure is a dichotomous measure of treatment success which is receipt of the first injection of XR-NTX.

Secondary objectives are to: (1) compare RP vs. SP across a range of secondary efficacy domains and clinical safety measures, (2) compare retention rates to subsequent second and third monthly XR-NTX injections.

Exploratory objectives include: (1) evaluation of demographic and clinical predictors of successful induction and moderators of differential effectiveness, (2) evaluation of whether the primary opioid of dependence (prescription painkillers vs heroin vs fentanyl) will interact with treatment, (3) evaluation whether fentanyl positive toxicology at screening and during treatment is predictive of relapse, (4) collection of a limited dataset to permit analyses of economic costs and benefits of the two treatment regimens, (5) compare time to induction failure in SP vs. RP (6) explore engagement with medical visits and therapy in SP vs. RP (7) compare use of MOUD as measured by patient self-report by treatment arm (SP vs. RP), and (9) compare percentage of participants inducted onto XR-NTX (during induction and post-induction phase) in RP vs. SP (8) explore percentage of induction failure participants receive XR-NTX during the course of the study.

The implementation goal is to operationalize an implementation facilitation strategy that will be used to train sites in the naltrexone initiation methods, evaluate implementation parameters including fidelity and reach, identify facilitators and barriers to implementation of RP, and refine the implementation facilitation strategy in an iterative process over the course of the trial. The ultimate goal is to emerge from the trial with an implementation facilitation strategy that can support widespread adoption of XR-NTX initiation across inpatients and residential treatment facilities.

2.3 Sample Size and Study Population

The projected sample size is approximately 450 participants, recruited across all study sites (approximately 75 at each of the six sites) over a study period of 70 weeks, at an enrollment rate of approximately 1-2 new participants per week per site. Study participants are treatment-seeking adults of all genders, at least 18 years of age or older, heroin- and/or prescription opioid-dependent volunteers, without chronic pain requiring opioid therapy, who are willing to accept naltrexone-based therapy and have no contraindications to XR-NTX.

2.4 Treatment/Assessment/Intervention and Duration

Two XR-NTX induction regimens will be evaluated: a Standard Procedure - SP typically a five-day buprenorphine taper followed by an 8-day washout period (naloxone challenge (e.g., 0.8-1.2 mg IM) and XR-NTX injection (380 mg IM) occurs 13 or 14 days from the first dose of buprenorphine and a Rapid Procedure - RP typically one day of buprenorphine (about 6-8mg), one day of washout, and 4-day oral naltrexone titration and XR-NTX injection occurs 6 or 7 days from the first buprenorphine dose. Both regimens have flexibility built into them according to clinical judgment, and sites will receive technical assistance and supervision according to the implementation-facilitation package developed for the study. All participants who complete the inpatient induction procedure (SP or RP) will receive the first XR-NTX injection while inpatient. Subsequently, all participants will enter the post-induction phase where they will be offered two additional XR-NTX injections, every 4 weeks, in combination with medical management clinical support. Participants will receive psychosocial counseling according to usual treatment at their participating treatment program. Study assessments will be collected at baseline, daily during the inpatient induction phase, and at weeks 1, 2, 3, 4, 6 and 8 during the post-induction phase.

2.5 Safety Reporting

At each medical management visit the study clinician will assess participants for AEs and SAEs. AE and SAE reporting will be in accordance with applicable regulatory requirements. As the safety profiles of the study medications are well established, and this trial is not powered to detect new, unknown safety signals, there will be no unsolicited AE collection during this study and only protocol-defined AEs (targeted safety events) and all SAEs will be collected.

2.6 Analyses

The primary outcome analysis will be performed using a generalized linear mixed effects model with a logistic link, a standard method for the analysis of stepped-wedge designs.

Secondary outcomes will be modeled using mixed effects regression methods.

Implementation outcomes will be modelled as continuous outcomes using mixed effects linear regression models. Additional implementation outcome measures including acceptability and barriers will be measured using qualitative methods.

3.0 STUDY SCHEMA

3.1 Schematic of Study Design

3.1.1 Table 1. Schematic of study design with six study sites and five steps, each lasting 14 weeks

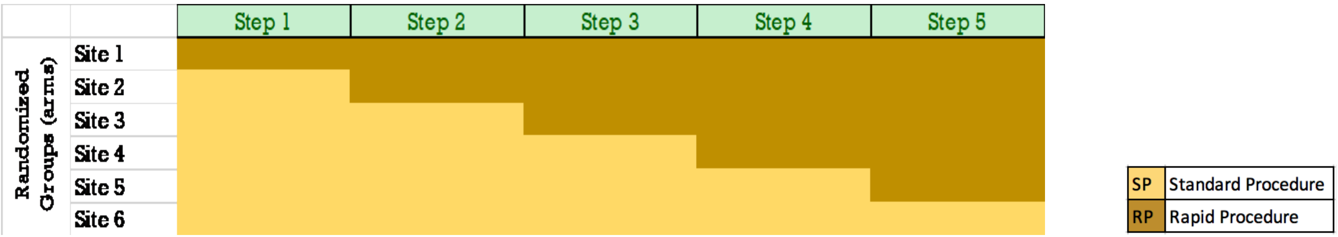

3.1.2 Figure 1. Schematic of the study phases and participant flow

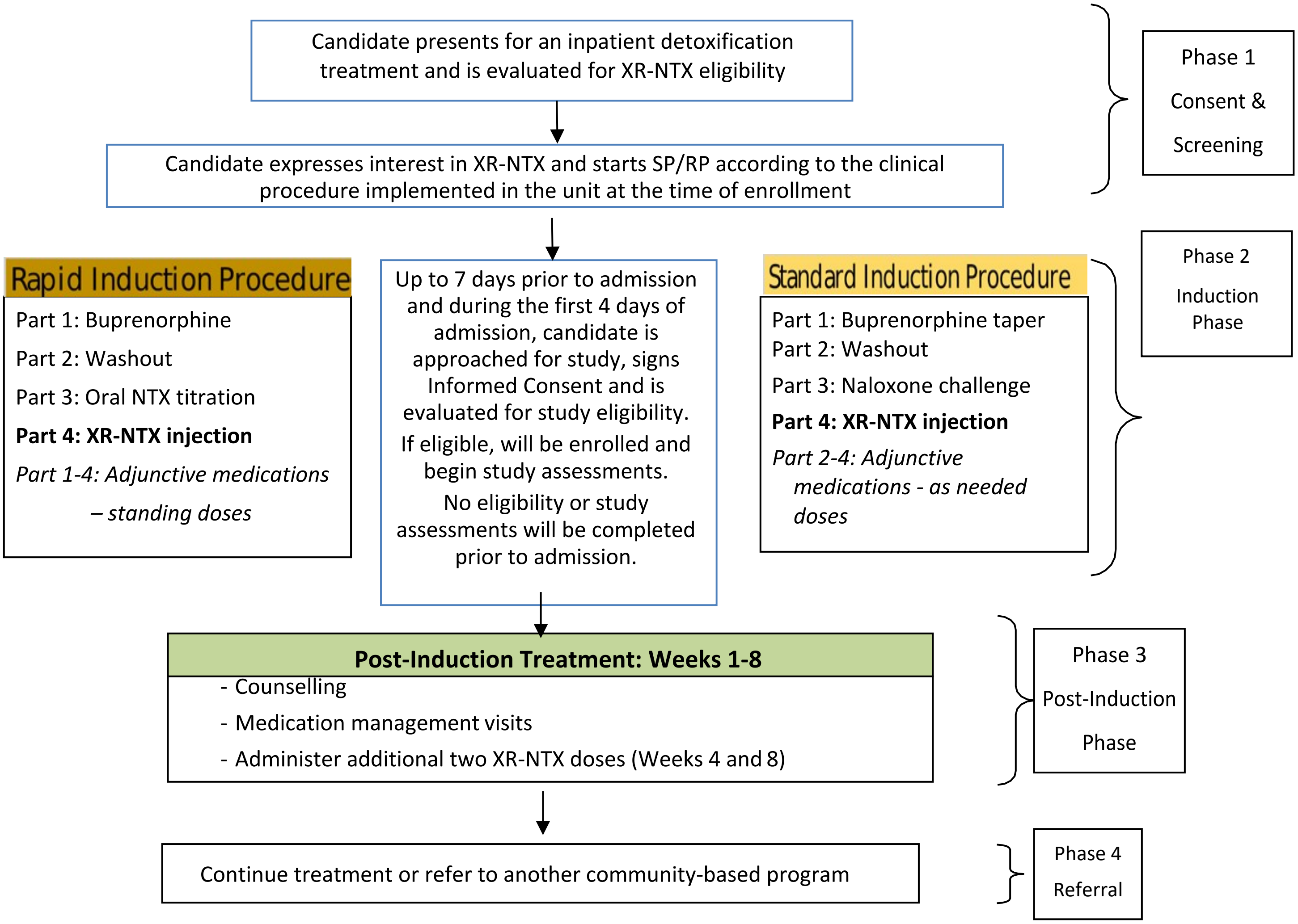

3.2 Key Research Site Roles

Each of the six research sites will require the following professionals:

- Site Principal Investigator
- Study Physician (NP; PA; MD; DO)
- Site Medical Clinician (NP; PA; MD; DO)
- Site Medical Personnel (RN; LPN; LVN)
- Research Coordinator
- Research Assistant
- Pharmacy professional (pharmacist or a pharmacy technician) if needed

4.0 INTRODUCTION

4.1 Background and Significance to the Field

4.1.1 Effectiveness of XR-NTX to Prevent Relapse Following Detoxification in OUD

Two community-based effectiveness trials in the U.S. (CTN-0051) (Lee *et al.*, 2018) and Norway (Tanum *et al.*, 2017) have shown that long-acting injection naltrexone (XR-NTX; Vivitrol®) is a viable alternative to buprenorphine as maintenance treatment for opioid use disorder (OUD). As an antagonist, with a distinct mechanism of action compared to partial agonist (buprenorphine) or full agonist (methadone), XR-NTX has the potential to attract more patients with OUD onto effective medication treatment nationwide. Many patients who otherwise would not have been open to treatment with agonist medications may find this antagonist-based treatment acceptable.

Ideally, patients and their clinicians would choose one of the three medication options (methadone, buprenorphine, or injection naltrexone) through a shared decision-making process. Among the medication options, extended-release injection naltrexone (XR-NTX) is currently the least utilized in the field as a whole. Recent large trials, including CTN-0051 (X:BOT), have shown that monthly XR-NTX is effective (Krupitsky *et al.*, 2011, Lee *et al.*, 2016), it is more effective than oral naltrexone (Sullivan *et al.*, 2019), and it is similar in effectiveness to buprenorphine maintenance (Lee *et al.*, 2018, Tanum *et al.*, 2017). In addition, XR-NTX may be particularly well suited for patients who are seeking relapse-prevention treatment that does not include an opioid agonist or those that have not responded well to buprenorphine or methadone.

However, XR-NTX remains under-utilized. An important barrier to the implementation of XR-NTX is the “induction hurdle,” namely the problem that among patients presenting for treatment with active opioid use, initiation of naltrexone requires a detoxification and transition from agonist to antagonist. The Official Prescribing Information for XR-NTX recommends a two-stage approach to naltrexone initiation, namely, an opioid detoxification followed by a 7 to 10-day delay from last agonist dose before starting naltrexone, to ensure that any opioids are off the receptors and physiological dependence is eliminated to minimize the risk of precipitated withdrawal. However, this involves patients tolerating acute and protracted withdrawal symptoms and a substantial delay to initiation of naltrexone, carrying the risk of dropout and relapse. This was the approach implemented in CTN-0051 (Lee *et al.*, 2018) where substantial rates of failure to initiate naltrexone were observed across sites—up to 47% failure across the 8 sites, with the highest failure rate among sites that used methadone taper as a detoxification strategy.

4.1.2 Addressing the Naltrexone Induction Hurdle

Several approaches exist for addressing the induction hurdle including methods decreasing the time to the injection by using minimal agonist treatment. Shorter methods minimize the drop-out rate during the wait period and increase the induction success rate. These “rapid methods” use only 1-3 days of buprenorphine at the outset followed by a rapid titration of oral naltrexone, starting at very low doses, with standing doses of adjunctive medications targeting emerging opioid withdrawal symptoms. The use of low doses of opioid antagonist to accelerate detoxification was

first proposed in 1970's with naloxone (Kurland and McCabe, 1976), later used to facilitate initiation of treatment with naltrexone (Resnick *et al.*, 1977). In this method, an antagonist, usually naltrexone, is administered without waiting for opioids to washout. The method starts with low doses of naltrexone, which do not precipitate withdrawal but prepare the system to tolerate a large dose of naltrexone; in this case, the first XR-NTX injection (Umbricht *et al.*, 1999). Because of the relatively low-level of withdrawal, rapid methods do not require sedation or anesthesia, techniques which are often used in so called "ultra-rapid" (1-day) methods (Gowing *et al.*, 2010).

The Rapid induction method can be as short as 5 days from the day of admission to the day of XR-NTX injection, but it can be extended by 1-2 days depending on individual patient tolerability of the naltrexone titration. Using this slow titration approach minimizes the severity of opioid withdrawal which is comparable to residual withdrawal present during buprenorphine taper (Sullivan *et al.*, 2017). The variations of this method include: duration of buprenorphine treatment as well as timing, starting doses, and duration of oral naltrexone treatment (Bisaga *et al.*, 2018b, Mannelli *et al.*, 2014, Sullivan *et al.*, 2017). Non-opioid adjunctive medications targeting symptoms of withdrawal are given as standing doses to reduce emergent symptoms of opioid withdrawal (Sigmon *et al.*, 2012).

Risks that apply to both the Standard and Rapid initiation regimens include opioid withdrawal symptoms during the detoxification period, drop out from treatment prior to initiating XR-NTX and drop out from treatment once XR-NTX has been initiated. Opioid withdrawal symptoms may be unpleasant but are generally not serious, while drop out from treatment increases risk for relapse leading to overdose, disability and death. Risks of the Rapid method include the possibility of more severe precipitated withdrawal with the use of low dose oral naltrexone or after XR-NTX induction as well as side effects from standing doses of adjunctive medications. Although we expect the RP to be non-inferior to the Standard method in terms of retention and receipt of XR-NTX, there is the possibility that increased withdrawal symptoms will lead to lower rates of retention.

In a single-site randomized controlled trial conducted at an outpatient research clinic, this rapid induction method was found superior to the standard approach of buprenorphine taper followed by 7-day delay to first naltrexone dose, on proportion of patients successfully initiating XR-NTX (Sullivan *et al.*, 2017). A recent study evaluated an 8-day version of the rapid XR-NTX induction protocol (Bisaga *et al.*, 2018b) in outpatient community-based treatment programs. The overall rate of success was approximately 44% and there was no difference between participants that received buprenorphine and low-dose naltrexone in addition to adjunctive medications and those that received only adjunctive medications. The reason for not showing additional benefit of buprenorphine taper or low-dose NTX may include an extended duration of treatment which eliminates the benefits of rapid naltrexone titration (i.e., participants that received oral naltrexone may be able to receive XR-NTX earlier than on day 8). Clinical experience suggests that low dose buprenorphine at the outset is important for controlling the most severe opioid withdrawal symptoms over the first several days in patients with more severe physiological dependence and tolerance levels. The low dose naltrexone titration has a practical advantage in that it establishes with relative certainty that the patient's system is ready to tolerate naltrexone and avoids the need for naloxone challenge. In summary, this study (Bisaga *et al.*, 2018b) showed that: 1) the rapid

method was feasible; 2) there was a higher failure rate for heroin users (suggesting that heroin users, who likely have greater physiological dependence and tolerance, may do better initially in inpatient settings where withdrawal symptoms can be managed more directly with non-opioid medication); and 3) experienced sites (sites with experience initiating naltrexone) enrolled more participants and had better success rate than inexperienced sites – suggesting a key need addressed by this CTN study, namely, 4) that a training and implementation package is needed to train and coach treatment programs to learn how to successfully initiate patients onto naltrexone.

4.1.3 Changing Practice for Inpatient OUD Treatment

Short-term inpatient units (so-called “detoxification units”) or short-term residential treatment programs are an important portal of entry for patients with OUD into the treatment system, offering patients an opportunity for stabilization and planning a long-term outpatient treatment regimen post-discharge. Historically, the standard of care on these types of units has been detoxification from opioids, often using a brief (typically 5-day) taper with methadone, or buprenorphine. However, clinical experience and mounting evidence from observational studies and clinical trials (Lee *et al.*, 2018, Nunes *et al.*, 2017) have highlighted the high rate of relapse to OUD among patients discharged from inpatient treatment without an effective medication for OUD having been initiated. Observational studies have shown an elevated risk of relapse, overdose and death after discharge from controlled settings in which patients have been detoxified, including prison or residential treatment (Binswanger *et al.*, 2013, Gossop *et al.*, 2002, Naderi-Heiden *et al.*, 2012, Pierce *et al.*, 2016, Strang *et al.*, 2003, Winter *et al.*, 2015). Thus, a powerful argument can be made that the standard of care on these units should change from detoxification to initiation on one of the effective maintenance medications for OUD (Bisaga *et al.*, 2018a). The implementation facilitation package for naltrexone initiation that will be developed and tested in this study should fill an important need in providing a blueprint for inpatient programs to follow in adopting injection naltrexone as part of their standard practice and treatment options.

4.1.4 Study Design

The CTN trial will be a hybrid Type I implementation-effectiveness design inpatient trial that will compare the effectiveness of Standard Procedure (SP) (per official prescribing information) versus Rapid Procedure (RP) to initiate XR-NTX, seeking to replicate the findings of the prior single-site outpatient trial (Sullivan *et al.*, 2017) across a sample of community- based inpatient detoxification or short-term residential treatment units. At the same time, we will develop and test the implementation package that will be used to train sites in the RP. We seek to determine whether the RP is non-inferior to the SP in community-based treatment settings, to understand factors influencing its implementation, and to emerge from the study with an implementation package that can support the widespread adoption of RP on inpatient units. There is a large population of opioid-dependent patients who enter detoxification programs to which the findings of the trial should generalize. We propose to conduct this study first on an inpatient basis in order to address this important population of patients. We have observed lower injection naltrexone initiation success rates among heroin users when initiation is attempted on an outpatient basis (Sullivan *et al.*, 2017), presumably due to the higher level of tolerance / dependence and correspondingly more withdrawal symptoms. This may be particularly true now that fentanyl and

other high potency synthetic analogs are commonly present in the heroin supply. The inpatient setting is likely better suited to aggressively treating withdrawal symptoms tailored to patients' needs.

4.1.4.1 Stepped-Wedge Design

We chose to implement stepped-wedge design after consideration of its relative strengths and limitations compared to patient-level randomized and program-level randomized parallel group designs (Handley *et al.*, 2011). While patient-level randomization is the preferred design for efficacy trials, and often applied for effectiveness as well, the evidence for efficacy of the RP vs. SP is already established (Sullivan *et al.*, 2017). Further, the SP and RP procedures being compared are service delivery interventions, involving quite different approaches to naltrexone initiation at the level of the treatment team and program. A patient-level randomized trial would have been logistically difficult to carry out at community-based treatment programs, asking programs and their teams to implement both procedures at the same time for different patients. A program (site)-level randomized, parallel group design was considered, but such designs typically require large numbers of sites, and only half of sites would get to implement the new RP. The program (site)-level randomized stepped-wedge design is appropriate for evaluation of service delivery interventions, where there is already evidence of effectiveness, and where there is an implementation aim in addition to an effectiveness aim, namely development and refinement of an implementation facilitation package for RP. The proposed stepped-wedge design allows study of implementation at each of the participating sites (except one site out of 6 sites), while furnishing a test of effectiveness compared to the SP naltrexone initiation approach.

As compared to the parallel-group (patient-level randomization), the stepped-wedge design used in the proposed study has the following advantages:

1. is more feasible to implement at sites with limited research infrastructure and support; each program will implement one regimen at a time, which is more feasible and has greater external validity (for other CTPs);
2. requires fewer resources to start, as staggered implementation of the experimental regimen can reduce training staff effort/burden by training only 1-2 sites at a time and focusing on training a program's clinical team on one naltrexone initiation method at a time;
3. increased enrollment, as participants can be enrolled and receive treatment more flexibly with no delay due to the need for participant-level randomization which creates fewer barriers for the participants;
4. fewer study implementation barriers, as there is no risk of contamination of the study arms. In an open label trial with similar medications but at different doses and different treatment durations, staff may be confused as to which study arm a participant belongs; further, participants, who will be housed together, will discuss/compare their treatment experience and may be more likely to withdraw from the study;
5. allows for both testing the comparative effectiveness of the RP vs. SP, as well as for developing, improving, and evaluating the implementation facilitation package with

continuous quality improvement with input from stakeholders. This is important given that a training intervention is needed for sustainability and disseminability; and

6. offering only one regimen at a time will be more naturalistic to real-world clinical practice, improving generalizability and dissemination value.

We have also considered a cross-over study design, with half sites starting with SP arm and half sites starting with RP arm, with a cross-over to the other arm half-way through the trial. This design offers a greater power to detect hypothesized effect size however it has significant limitations including: 1) difficulty with implementing regimen in practice as half the sites will be asked to switch from the Standard Procedure to the Rapid Procedure and then back to the Standard Procedure with a possibility of a carry-over effect (sites may not want to change back to a longer, more expensive, and less effective regimen, and 2) fewer opportunities (only two steps) to develop and improve the Training Package using the iterative process.

4.1.4.2 Adjunctive Medications

In the proposed trial we will use clonidine as the primary adjunctive medication which is one of several presynaptic alpha-2 adrenergic agonist which are used clinically to alleviate opioid withdrawal (Gowing *et al.*, 2016). We considered an alternative choice of lofexidine. Although lofexidine, unlike clonidine, is FDA approved for treatment of opioid withdrawal we decided that a regimen that includes clonidine will have greater chance of dissemination since it is inexpensive, and is already in wide use, unlike lofexidine. Moreover, the package insert for lofexidine includes the risk of QT prolongation which is not the case for clonidine. In previous trials lofexidine has been used previously to facilitate rapid induction onto naltrexone (Buntwal *et al.*, 2000, Gerra *et al.*, 2001). In comparison to clonidine the data was mixed, with some trials finding lofexidine to be better tolerated (Carnwath and Hardman, 1998, Gerra *et al.*, 2001) while another trial did not (Kahn *et al.*, 1997). Considering the mixed trial data regarding the superiority of lofexidine over clonidine and significantly higher cost and implementation hurdles facing lofexidine we chose to use clonidine.

Documenting effectiveness of clonidine will serve as a proof of concept for other alpha-2 adrenergic agonist such as tizanidine (Rudolf *et al.*, 2018) or guanfacine (San *et al.*, 1990). We believe that future studies, especially those conducted in an outpatient setting, might consider whether lofexidine is better tolerated than clonidine.

4.1.5 Duration of XR-NTX Treatment

The primary study goal is the success of XR-NTX induction, i.e., receiving the first injection. However, we will continue treatment by offering two additional XR-NTX injections. First, the acceptance of the second and third injections is an important outcome and may be reflective of the tolerability of the first injection and the induction procedure overall. The first three months of XR-NTX treatments is the time when greatest dropout is anticipated (Sullivan *et al.*, 2019) and therefore providing treatment with three consecutive injections is ethically favorable. The post-induction portion of the study will last 8 weeks, until the third XR-NTX injection is given, at which point participants will complete final study assessments and will transition to continuing treatment in the community.

4.1.6 Evaluation of Implementation Parameters

One of the main challenges in the field of medication-based treatment of OUD is the gap between evidence-based approaches developed and tested in research settings and real-world practice.

Therefore, research on new methods to initiate treatment with XR-NTX should include an evaluation of factors that may impact successful implementation of research findings into routine practice. A secondary goal of the proposed study, therefore, will be to understand potential facilitators and barriers to implementation of rapid initiation of XR-NTX, knowledge that will be important in subsequent efforts to achieve widespread implementation across community-based treatment settings. In the proposed trial, we will focus on the inpatient setting as this is the most frequently used approach in addressing opioid use disorder in the community. Using the Consolidated Framework for Implementation Research (CFIR; (Damschroder and Hagedorn, 2011)) to guide measurement selection, we will use mixed methods to assess the domains of intervention characteristics (e.g., complexity, relative advantage), inner setting (e.g., readiness, clinical leadership), individual characteristics (e.g., provider beliefs, self-efficacy), and implementation process (e.g., fidelity and feasibility).

4.1.7 Implementation Facilitation Manual Development Process

Implementation Facilitation (IF) is defined as a "process of 'helping individuals and teams to understand what they need to change and how they need to change it in order to apply evidence to practice" (VA Mental Health Queri, 2016). IF is an effective approach that includes a "deliberate process of interactive problem solving and support that occurs in the context of a recognized need for improvement and supportive interpersonal relationship" (Stetler *et al.*, 2011). A recent systematic review and meta-analysis found that IF has a positive impact on guideline adoption in primary care. A central aspect of IF includes the active role of the facilitator(s) working in partnership with relevant stakeholders, and other implementation intervention components. In this case, we will leverage experience gained in implementation of novel designs in previous work to develop an implementation plan that interfaces with these stakeholders through formal training and a "formative evaluation" to identify the specific and dynamic needs of stakeholders and the context (i.e., detoxification units) for implementation of evidence-based practices that may be specific to the site. This "diagnostic" formative evaluation informs the initial tailoring and refinement of the IF, which includes the "bundle" of services tailored to meet site-specific needs.

Tailored implementation strategies have been found to be more effective than non-tailored interventions in changing practice (Baker *et al.*, 2010) and have been applied to changing primary care and mental health treatment delivery (Johnson *et al.*, 2014, Kirchner *et al.*, 2014) IF has been used by large healthcare organizations including the Veterans Health Administration and is endorsed by the Agency for Health Care Research and Quality as a way to assist practices in becoming Primary Care Medical Homes.

In this study, formative evaluation of the IF process will be iterative and will occur following each stepped wedge, as well as at the trial's conclusion. Prior to the first step, a subset of the research team will develop an initial prototype IF process, based on current research and practice knowledge of implementation strategies and processes. The prototype will include approximately 4 hours of staff education and training, academic detailing with each clinical team weekly (or more

frequently based on site needs), and structured clinical materials. Following each step of the study, relevant stakeholders including site clinicians, individuals from the research team with expertise in implementation, and clinical experts will meet to identify and address barriers to the implementation, the need to modify or adapt particular strategies, and ways to modify the IF manual for parsimony and efficiency.

Given our multifaceted implementation approach, a structured framework and process will be used to capture adaptations or modifications to the implementation strategies throughout the IF process. We will combine the Framework for Reporting Adaptations and Modifications to Evidence-Based Implementation Strategies (FRAME-IS) (Miller *et al.,* 2021) with additional tracking (informed by the Expert Recommendations for Implementing Change (ERIC) (Perry *et al.,* 2019, Powell *et al*., 2015, Waltz *et al.,* 2015) and Proctor *et al.*'s guidance on specifying implementation strategies (Proctor *et al.,* 2013). Each modification or adaptation will be described along with the reason(s) for the change (informed by the CFIR framework). The iterative nature of these adaptations and use of FRAME-IS provides the opportunity to simultaneously examine the implementation components and feasibility of using this tool for a dynamic implementation facilitation approach. In this way, at the conclusion of the study, a high-quality IF manual will be completed that may be used for dissemination of the brief induction procedure or further implementation testing. In addition, this will add to the implementation science literature by providing a reproducible process for evaluating implementation facilitation using the ERIC taxonomy for strategies; although specific to the RP intervention, certain processes and implementation strategies are generalizable to implementation of evidence-based interventions.

4.2 Innovation

This would be the first community-based effectiveness-implementation trial of a method of rapid initiation of XR-NTX for treatment of OUD. This has the potential to move the field in a new direction of adopting rapid initiation of XR-NTX as standard practice in short-term inpatient units. Clinicians currently hesitate to consider XR-NTX in part because of lack of experience and concerns about the feasibility and the length of time needed to initiate XR-NTX. Clinicians are concerned about following the recommendation of the Official Prescribing Information, which calls for a waiting period of 7 to 10 days after last dose of opioid before administering XR-NTX (Vivitrol). Administrators and third-party payors often do not support longer inpatient stays and clinicians are concerned about the risk of dropout, relapse and overdose during the wait period. The proposed trial seeks to move the field in a new direction by showing that XR-NTX can be safely and effectively administered within the typical 5 to 7-day window of acute inpatient ("detoxification") stays for opioid use disorder.

The proposed trial would be among the first hybrid effectiveness-implementation trials (Curran *et al.*, 2012) to be conducted by the CTN, where implementation factors at the levels of patients, clinicians, and treatment programs are studied in conjunction within a randomized effectiveness trial. A key goal of effectiveness trials conducted in CTN is to ultimately promote widespread dissemination and adoption of effective interventions; thus, studying the implementation processes should provide critical information to assist in more rapid uptake.

4.3 Suitability, Feasibility, and Sustainability for the CTN Sites

We believe that a large number of inpatient treatment programs will be interested in participating in a trial testing a regimen for rapid XR-NTX treatment initiation. During the protocol development process for CTN-0051, over 30 CTN-affiliated inpatient treatment programs applied to participate in the trial, among which only 8 could be selected. This is consistent with the large number of such inpatient or residential treatment programs throughout the U.S. health system that are interested in implementing evidence-based medications for OUD, and the extent to which these programs are confronting how best to care for the many patients with OUD that they admit. The CTN should be able to easily supply a sample of appropriate inpatient treatment programs for the proposed trial and provide a vehicle for rapidly disseminating the findings across the network and field.

4.4 Public Health Impact Significance to the Field

The epidemic of OUD and opioid overdose deaths is a major public health challenge. Short-term inpatient addiction treatment units are a common portal of entry into treatment for patients with OUD. Such programs represent an important opportunity for initiating patients with OUD onto effective medication treatment. The current proposal seeks to facilitate the larger goal of making initiation of medications for OUD standard practice for inpatient treatment programs, by addressing the naltrexone “induction hurdle,” which is a significant barrier to the implementation of XR-NTX. Improving success of XR-NTX treatment initiation has important potential public impact via reducing barriers to the use of XR-NTX, thus potentially expanding access to this effective alternative across the health care system.

5.0 OBJECTIVES

5.1 Primary Objective

The primary goal of the study is to determine whether the RP method of initiating treatment with XR-NTX is non-inferior to the SP method recommended in the XR-NTX Prescribing Information. The primary outcome measure is a dichotomous measure of treatment initiation success defined as receipt of the first injection of XR-NTX while inpatient. Treatment initiation failure, defined as failure to receive first injection of XR-NTX while inpatient, can be subdivided for descriptive purposes into 1) decision by patient and their clinicians to abort the naltrexone initiation effort and change to initiation of buprenorphine or methadone maintenance; 2) dropout or discharge from the inpatient unit prior to receiving XR-NTX. We hypothesize that the RP will be non-inferior to SP in terms of proportion of successful initiations of XR-NTX.

5.2 Secondary Objective(s)

A key secondary objective is to summarize the below outcome to confirm expected characteristics of the RP compared to SP:

1. Time to receipt of first injection of XR-NTX from day of admission for participants that receive first injection of XR-NTX while inpatient.

Additional key secondary objectives are to compare the below outcomes of RP versus SP for all enrolled participants (ITT population).

2. Craving for opioids measured by Visual Analog Scales (VAS).
3. Opioid withdrawal symptoms as measured by the Subjective Opioid Withdrawal Scale (SOWS) and the Clinical Opiate Withdrawal Scale (COWS).
4. Safety, as measured by overdose questionnaire, targeted safety events and serious adverse events.

Other secondary objectives are to compare the below outcomes of RP vs. SP for participants who receive the first injection while inpatient.

5. Retention in the trial to receive the second and the third XR-NTX injections.
6. Craving for opioids measured by Visual Analog Scales (VAS).
7. Opioid withdrawal symptoms following XR-NTX injection as measured by the Subjective Opioid Withdrawal Scale (SOWS)
8. Safety, as measured by overdose questionnaire, targeted safety events and serious adverse events.
9. Opioid abstinence, as measured by the Timeline Followback (TLFB) (self-report days using opioids) and proportion of opioid-positive urine tests.

5.3 Exploratory Objective(s)

1. Explore baseline demographic and clinical features (e.g., the primary opioid of dependence (heroin/fentanyl vs. prescription opioid)) as: a) predictors of induction

success, secondary outcomes and retention during the trial (main effect of predictors), and b) as moderators of differential treatment effect (moderator by treatment interaction).

2. Compare duration of inpatient treatment and the associated costs from the time when detoxification is initiated to the time that XR-NTX is administered to permit analyses of economic costs and benefits of the two treatments.
3. Compare RP versus SP for all enrolled participants in terms of time from day of admission to XR-NTX initiation failure (day of discontinuation of detoxification period that resulted in failure to receive 1st XR-NTX) and reasons for failure.
4. Compare RP versus SP for all enrolled participants in terms of other depressive, anxiety, and subacute withdrawal symptoms as measured by the Patient Health Questionnaire-9 (PHQ-9) and General Anxiety Disorder-7 (GAD-7).
5. Compare RP versus SP use of alcohol and other drugs of abuse (e.g., cocaine, other stimulants, cannabis, benzodiazepines), by self-report and urine drug screens for all enrolled participants.
6. Explore engagement with medical visits and therapy (based on Medical Management Log, Psychosocial Log, XR-NTX Administration Form, TLFB).
7. Compare RP versus SP for all enrolled participants in terms of use of MOUD as measured by patient self-report on Timeline Followback (TLFB).
8. Investigate the percentage of induction failure participants that receive XR-NTX during the course of the study overall and for each treatment arm as measured by patient self-report on Timeline Followback (TLFB).
9. Compare RP versus SP for percentage of participants inducted on XR-NTX (during both induction and post-induction phases of the trial).

5.4 Implementation Objectives

The objective of the implementation component of the study is to understand facilitators and barriers to implementation of Rapid initiation of naltrexone and to iteratively develop an implementation facilitation manual which can be used to disseminate XR-NTX initiation methods across the treatment system. Measurement will be grounded in the CFIR (Damschroder and Hagedorn, 2011) and will assess intervention characteristics (provider survey), inner setting factors (e.g., readiness, clinical leadership structure, resources; survey and environmental scan), individual provider characteristics (provider survey), and implementation process through fidelity measures. Following each stepped wedge, this information will be integrated into the IF manual (by adding new information and/or making modifications to existing information) to improve the procedures. The ultimate goal will be to produce a high quality IF manual at the conclusion of trial which could facilitate widespread implementation of XR-NTX induction across community-based treatment settings, as well as be tested within its own right in a larger implementation-focused trial.

6.0 STUDY DESIGN

6.1 Overview of Study Design

6.1.1 Overview

This is a six-center, stepped-wedge, cluster randomized trial comparing effectiveness and safety of Rapid (5-7-day) versus Standard (13-day) XR-NTX induction procedure (RP vs. SP). Sites will be addiction treatment programs offering inpatient/residential "detoxification" and follow-up outpatient treatment (site characteristics are described in Section 9.0). Patient-participants will be admitted to these sites for treatment of opioid use disorder, eligible for and seeking treatment with XR-NTX, and consenting to research assessments during their course of XR-NTX treatment. Upon enrollment in the study, participants will receive the naltrexone initiation regimen being offered at the site at the time of admission, either SP or RP depending on the random assignment as per study design (Figure 1). The post-induction phase will begin the day after receiving XR-NTX injection. During the 8-week post-induction phase, participants will receive medication management visits per the site's standard clinical practice, plus an additional two XR-NTX injections, one at four weeks and one at eight weeks after the first XR-NTX injection, along with other psychosocial treatment as per the standard of care provided by each site. The primary effectiveness outcome measure is induction success defined as the administration of the first XR-NTX injection. The primary implementation outcomes derive from the process evaluation of implementation using the CFIR framework and evaluation methods.

6.1.2 Patient-Participants

Patients with OUD planning admission or admitted to inpatient/short-term residential treatment sites will participate in a shared decision-making process regarding choice of maintenance medication (methadone, buprenorphine, XR-NTX). All those determined to be clinically appropriate by their admitting clinicians for XR-NTX and choosing XR-NTX will be offered participation in the study and will undergo informed consent procedures before completing any study-related activities.

6.1.3 Random Site Selection and Treatment Assignment

The study will proceed in five steps, with each step lasting 14 weeks, for a total of 70 weeks of the study (Table 1). At the beginning of each step a randomly selected site will begin implementing RP and will continue to offer it for the remainder of the trial, according to the optimized stepped-wedge study design (Thompson *et al.*, 2017). At the outset of the trial (Step 1), one randomly selected site will initiate RP while the remaining five sites will continue providing XR-NTX initiation using the SP. At the beginning of Step 2 another randomly selected site will stop implementing SP and will begin implementing the RP. The same process will take place at the beginning of Steps 3, 4, and 5. The remaining site, one that has not been selected to transition from SP to RP during the five steps will be offering SP for the 70 weeks duration of the trial. After the transition to RP, this will be the only regimen available for patients interested in participating in the research study (i.e., SP will no longer be offered as a part of the study). However, standard clinical care may be provided for individuals admitted to the study sites/units but not interested in the research

study.

6.1.4 Training in SP and RP procedures

At the beginning of the trial all sites will receive training in the study procedures. In addition, the five sites chosen to offer SP will receive an overview training and review of practices in the SP, while the site selected to start with RP in Step 1 will receive an overview training in the RP. This didactic training will occur before the study start date. At the start date participant enrollment begins, the units chosen to begin by offering SP will receive academic detailing in SP, while the unit assigned to begin RP will receive the further targeted RP implementation facilitation intervention (training and academic detailing) in an 8-week pre-implementation or preparation phase before the start of the RP implementation step. Upon transition to RP implementation (or start of the step), RP academic detailing and coaching support will be held weekly or more frequently based on the site's needs. After individual site-level coaching support sessions, all sites randomized to RP will participate in collaborative coaching sessions that occur weekly or more frequently based on sites' needs.

Study Schema

The flow of a patient-participant through the study, at either SP or RP sites, will proceed in four phases (see Figure 1).

6.1.4.1 Phase 1 & 2 Inpatient Induction (Recruitment and Induction Phase)

The clinical procedures for all individuals interested in XR-NTX will be determined by the assignment of the unit to either the Rapid or Standard Procedures and the clinical judgement of the unit staff. These will begin immediately upon admission and will be independent of study participation. Recruitment for research participation will occur independent of the clinical procedures any time from seven days before admission until the fourth day of admission.

Research Recruitment

The research recruitment phase begins with informed consent up to one week before or up until the fourth day of inpatient admission to the study site. During the inpatient admission intake/evaluation or the first four days of an inpatient admission, clinical staff, as part of standard clinical procedures, will conduct a medical and psychiatric workup, and conduct a shared – decision-making process regarding choice of maintenance medication to be initiated during the inpatient stay. During this process research staff will provide information about the study to potential participants, describing XR-NTX as an option for relapse-prevention to opioid dependence following detoxification, and the study plan to monitor the progress of patients undergoing initiation and initial months of treatment with XR-NTX using materials prepared for the study. For scheduled admissions, this information may be provided in advance of the admission. Once a patient decides (with their clinicians) to pursue XR-NTX treatment, then the patient can be offered informed consent to participate in the study.

After the participant provides written informed consent, the participant will be evaluated for study eligibility. Eligible participants will continue with study assessments. Participants may be stabilized

on buprenorphine before study enrollment but those who received methadone at treatment outset (e.g., in the ER or upon arriving at the unit) are not eligible for enrollment.

Clinical Induction Procedures

Following the initial buprenorphine treatment, the units will provide participants with treatment guided by the regimen for the respective study arm as implemented by clinical staff at the detoxification program (Tables 6A and 6B).

Standard Procedures: The plan is for 13-14 days from first dose of buprenorphine in the Standard Procedures (SP) for induction, detailed in Table 6A; this includes a 5-day buprenorphine taper followed by an 8-day washout before being eligible for the XR-NTX injection (as determined by the naloxone challenge, naltrexone titration, or toxicology). In case that participant does not meet criteria of eligibility by Day 14, the eligibility determination can be repeated on subsequent days. Since the SP regimen will be the standard of care for XR-NTX initiation during the SP phase of the participation of a Site, there will be flexibility based on clinical judgment.

Rapid Procedures: The plan is for 5-7 days from first dose of buprenorphine in the Rapid Procedures (RP) for induction, detailed in Table 6B; this includes 1 day of buprenorphine followed by 1-day washout and a 4-day period of treatment with low-doses of oral naltrexone. Adjunctive medication will be given throughout the RP. Again, since the RP regimen will be the standard of care for XR-NTX initiation during the RP phase of the participation of a Site, there will be flexibility based on clinical judgment. For example, it is possible that some participants will not be able to tolerate naltrexone 6 mg on Day 6 and in those participants, XR-NTX may be administered on Day 7 or thereafter.

After administration of XR-NTX participants will continue treatment per standard clinical practice. The day after the first XR-NTX injection is received or the day after the decision to discontinue XR-NTX induction is considered the Post-Induction Day 1. Participants who receive the first XR-NTX injection will be classified as meeting criteria for “XR-NTX induction success.” Participants who discontinue the induction phase before receiving the XR-NTX injection will be classified as “XR-NTX induction failure.” These participants will continue in the study but will only follow up at Week 4 and Week 8.

6.1.4.2 Phase 3 (Post-Induction Phase): Follow-Up Treatment

Following inpatient XR-NTX induction phase, participants will continue with the post-induction treatment for 8 weeks (post-induction Week 1 through Week 8; Days 1 – 55). Most participants will be treated on an outpatient basis during this phase, but some may be in extended residential or therapeutic community programs. Participants that receive first XR-NTX are expected to receive a second injection of XR-NTX towards the end of Week 4 and a third injection towards the end of Week 8 in combination with weekly Medical Management visits. The study will supply the three XR-NTX injections (initial and at Weeks 4 and 8). Study assessment visits occur weekly during this phase, with an End of Study (EOS) visit occurring at Week 8. For participants who request discontinuation of treatment, relapse, and/or become lost to follow up, a +14 day window is permissible to complete the EMT visit.

Patients who fail to initiate XR-NTX while inpatient will also be followed and evaluated at Week 4 and Week 8 to determine what treatment they are receiving (for example, whether they initiated XR-NTX after leaving the unit or if they initiated and remained on another medication (buprenorphine or methadone maintenance)) and whether or not they have relapsed to opioid use.

6.1.4.3 Phase 4 (Referral Phase) Referral to continuing treatment

During the last month of study, as part of standard clinical care, arrangements will be made by the clinical team for participants to either continue treatment at the same program or be referred to another program in the community for follow-up care, which should ideally include continuation of XR-NTX. The third XR-NTX injection will be administered as a part of the study in order to objectively assess the retention in treatment 8-weeks after the induction and to assure the continuation of treatment with XR-NTX during the post-study transition period. However, participants will not be followed with study assessments after third injection as at this point, they will be engaged with and followed in the community-based clinical care where they will be assessed medically following standard procedures.

6.1.4.4 Management of participants who fail to initiate XR-NTX

Participants will be made aware, as part of standard clinical care during initial clinical evaluation at the site, and also at the time of study consent, that other treatment options, particularly buprenorphine, are available at any time if the patient is not tolerating the XR-NTX induction, or otherwise changes their mind and wants buprenorphine instead, or initiation and referral to methadone maintenance if locally available. Effort will be made to avoid having patients leave the inpatient-residential unit without initiating an effective medication for OUD. Patients who fail to initiate XR-NTX will be followed and evaluated at Week 4 and Week 8.

6.2 Implementation Facilitation

A secondary goal of the current study is to develop an Implementation Facilitation (IF) strategy to facilitate the increased adoption and implementation of Rapid Induction onto XR-NTX in inpatient detoxification and residential facilities. We will use IF which, as mentioned above, provides a “deliberate process of interactive problem solving and support that occurs in the context of a recognized need for improvement and supportive interpersonal relationship.” Through this organized and iterative process, the needed steps will be taken to support inpatient unit staff and to promote uptake of XR-NTX treatment initiation among enrolled participants.

The Rapid Procedure involves systematic implementation facilitation support. The study team will act as external facilitators throughout the implementation process for RP and will reduce support as sites take lead in implementation. Before initiating the trial, we will develop a complete IF package for the Rapid Induction Procedure based on our prior experience training clinical teams for clinical trials and through the SAMHSA-funded Providers’ Clinical Support System (PCSS).

Before each stepped wedge of the study, an 8-week pre-implementation or preparation phase will occur for each site randomized to RP. Note that during the pre-implementation phase the randomized site will continue to offer SP until the actual transition date to the next step. Pre-implementation activities include identification of a local implementation team and champions,

Site Mapping or an implementation needs assessment, adaptations or modifications to implementation strategies based on site's needs, intensive staff training, and review of the RP IF package and clinical tools.

During the implementation phase of RP, the study team will meet weekly, or more frequently if needed, with site's clinical staff and other key stakeholders to provide coaching support and monitoring during the initial implementation of the intervention. The study team or lead implementation team will meet routinely to review the implementation process and identify modifiable barriers or facilitators to facilitate RP implementation. The study team will reduce coaching support as each site takes lead in implementing RP and at that point the site will shift into the maintenance phase. During the maintenance phase, all sites randomized to RP will meet routinely in a collaborative coaching session and the study team will reduce support further and act in a monitoring role.

After each stepped wedge, the study team with expertise in implementation will meet to review identified barriers and facilitators to implementing the RP intervention and modify or adapt the RP IF package accordingly. Barriers and facilitators mapped across the CFIR framework will be captured using various qualitative data including structured notes from Site Mapping meetings and coaching sessions, internal implementation team meeting notes, and staff structured interviews at the end of each stepped wedge. Review of other implementation-level data at the end of the study will inform final adaptations or modifications to the RP IF package. This IF package will form the basis of technical assistance and support widespread dissemination of rapid XR-NTX initiation.

The Standard Procedure follows treatment as usual for induction onto XR-NTX and it is expected that site-level processes are already established for implementing SP (preconditions to be selected for study participation). Given these preconditions, SP sites do not require systematic implementation facilitation support. A brief overview and training of SP (primarily based on the XR-NTX prescribing information) will be provided at the start of the trial. Throughout the trial, the study team will meet with sites implementing SP for monitoring and engaging treatment teams in study participation.

6.2.1 Initial Elements Included in Implementation Facilitation

The following elements will be included in the initial implementation of the Rapid Procedure and facilitation package;

**Pre-Implementation/Planning Phase**	
Identifying a local Implementation Team and Champion	A local implementation team and champion will be identified at each site to help guide and motivate change in the local context. This team may include executive leadership, key clinical staff, site PI, or other relevant stakeholders essential to the success of implementing the Rapid Procedure.
Site Mapping	We will meet with the identified local implementation team weekly (for approximately 4 to 5 weeks depending on each site's needs) and conduct an implementation needs assessment that identifies relevant staff and procedures on the detoxification unit, facilitates site-level buy-in and engagement, maps inner organizational processes, external factors influencing the success of

	implementation, and identifies potential barriers and facilitators to implementing the intervention.
Facilitate Buy-in and Engagement	Site Mapping meetings will begin process of facilitating buy-in and we will encourage engagement of staff (not involved in these meetings) by internal facilitators and local champions.
Training Needs Assessment	We will identify specific training needs at each site and customize delivery of training and procedural support.
General Staff Training and Orientation	We will provide a brief intensive training program for unit staff outlining the clinical procedures in the Rapid Induction Procedures, commonly encountered clinical issues, and provide guidance on communicating with patients throughout the procedure.
Customization of Clinical Tools	Clinical tools developed by the study team will be reviewed with the site staff and customized accordingly to their needs. Clinical tools and handouts include a structured progress note, clinical algorithms for the procedure, flow charts, checklists, order sets, shared decision-making handout on medications for opioid use disorder, etc.
Logistics Planning and Procedural Support	Any necessary modifications to internal procedures will be addressed prior to implementation of RP when feasible. All providers and staff will receive a manual describing in detail the steps involved in the RP with detailed guidelines on prescribing and monitoring. Clinical tools will be reviewed and customized as per local staff needs.
**Implementation Phase**	
Coaching and Procedural Support Sessions	We will act as external facilitators to implementation of the RP intervention and meet with the site weekly, or more frequently if needed, to provide coaching and procedural support. Site staff will include key clinical staff and other relevant stakeholders (i.e., members of local implementation team).
Expert clinician consultation as needed	Expert clinicians on the study team will be readily available and on call to clinical staff during the initial implementation phase for acute clinical concerns or questions.
Individual-level feedback from local champion(s) and stakeholders	We will interview individual staff from each site at the end of the 14-week stepped wedge to gather feedback about their experience implementing the procedure, other key issues not identified in group meetings, and any other barriers or facilitators to implementation and the maintenance of the RP.
**Maintenance Phase**	
Collaborative Coaching Sessions	All sites randomized to RP will meet routinely (weekly or less frequently depending on sites' needs) for collaborative coaching sessions. The study team will attend these sessions for monitoring, and will be available if additional coaching is needed but collaboration and coaching between site staff will be encouraged with a longer term goal of these sessions continuing after study completion.
Individual Site-Level Coaching Support as needed	Individual site coaching sessions with the study team will be available as needed.

6.2.2 Iterative Development of the Implementation Facilitation Package

After each step of the study and RP implementation, a subset of the study team with implementation expertise will meet to review barriers and facilitators mapped across the Consolidated Framework for Implementation Research (CFIR). The CFIR identifies elements for determining successful implementation of an evidence-based practice into clinical care, including:

1. Characteristics of the **intervention**,
2. Individual **recipients** of the facilitation, including people affected by and who influence implementation of the innovation,
3. Qualities of the inner and outer (i.e., social, policy, regulatory and political infrastructures) **context** in which the evidence is being introduced and enacted upon, and
4. **Facilitation** (i.e., the implementation intervention), the active process of promoting implementation by assessing and responding to the recipients and associated context.

The CFIR domains contain various constructs that are determinants or measures related to implementation of the intervention. These measures will be assessed after each stepped wedge across various qualitative data including structured notes from Site Mapping meetings and coaching sessions, internal implementation team meeting notes, and staff structured interviews at the end of each stepped wedge. Staff will include medical prescribers, nursing staff, rehabilitation clinicians, social workers, and unit administrators. Identified barriers and facilitators (mapped across the CFIR framework) will inform modifications and adaptations to implementation strategies and the IF package. Modifications and adaptations to implementation strategies will be categorized using the FRAME-IS model, the ERIC taxonomy for naming of implementation strategies, and Proctor and colleagues' guidance on specifying implementation strategies (REF).

Review of other implementation-level data at the end of the study will inform final adaptations or modifications to the RP IF package. These data include the qualitative listed above as well as patient satisfaction survey and quantitative data (Organizational Readiness to Change Assessment (ORCA) and readiness/preparedness rulers administered to individual staff, fidelity to the implementation facilitation components (fidelity to implementation assessments), fidelity to the intervention (critical action checklists and daily medication administration log), and proportion of participants successfully inducted onto XR-NTX. This strategic approach will facilitate refinement and production of a high-quality IF manual for widespread dissemination.

6.3 Duration of Study and Visit Schedule

Each participant is engaged in the overall study for approximately 13 weeks as follows:

- Up to 11 days: consent, screening (up to 7 days prior to inpatient admission; up to 4 days from admission, including admission day).
- Up to 30-day induction phase: starting with day of admission up to day of 1st XR-NTX (or day of detox failure): Daily research assessment.
- 8 weeks (56 days) of post-induction treatment. Weekly research assessment.
- Post-study transition to community-based treatment.

6.4 Study Timeline

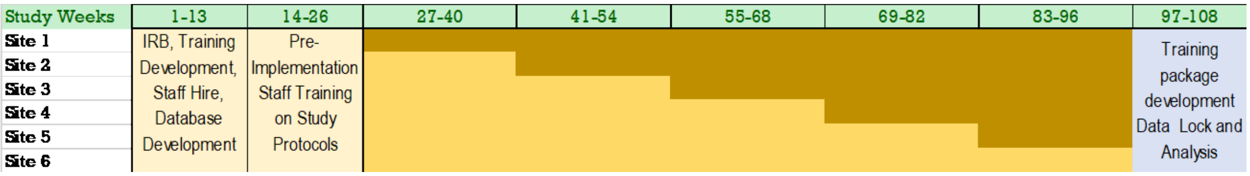

6.4.1 Table 2: Study Timeline

The first step of trial preparation includes obtaining IRB approval of the protocol for all study sites; this is expected to last up to 6 weeks.

After receiving CCTN approval of the full/final protocol, approximately 13 weeks of trial preparation activities will elapse prior to commencing enrollment of participants. This will include developing the data collection forms, developing the manual of operating procedures (MOP), developing the study and SP training plan and implementation guidelines for the RP, hiring staff and conducting all staff training, sites' securing CLIA Certificates of Waiver (as needed), completing Site Initiation Visits and action items required for endorsement, and endorsing sites.

If feasible, the initial study initiation may be implemented in a single wave; Recruitment is expected to take approximately 70 weeks, with follow-up continuing for approximately 10 weeks post completion of the recruitment phase. Following completion of the induction phase at week 96 (when data collection for the primary outcome has been completed), approximately three months of data cleaning will elapse. Data lock will take place at approximately Week 100, or at minimum 2 months following last participant's last visit; at the same time work will continue on developing a training package.

Therefore, data lock is projected to occur at approximately 25 months after IRB approval of the initial protocol. The preliminary analyses and a draft of the final implementation package will be developed within four months of the data lock.

7.0 OUTCOME MEASURES

7.1 Primary Outcome Measure

The primary goal of the study is to show RP is non-inferior to SP XR-NTX induction method.

The primary outcome measure is the proportion of patients who receive the first XR-NTX injection (dichotomous: did or did not receive first dose of XR-NTX).

We hypothesize that a Rapid Induction Procedure will be non-inferior to a Standard Induction Procedure in terms of proportion of patients with successful inductions while inpatient.

7.2 Secondary Outcome Measure(s)

7.2.1 Table 3: Secondary Clinical Outcomes, Outcome Measures and Hypotheses

Outcome	Outcome Measure	Hypothesis
Time from admission to first XR-NTX injection	Days to first XR-NTX injection	Participants in the RP will receive their first injection significantly faster than those in the SP. Significance/Rationale: The RP decreases the time to the first injection (which has a potential to decrease costs and staff burden).
Opioid Craving (VAS) over time	Mean for opioid craving measured by VAS daily during days leading to the first XR-NTX injection, and during post-induction Weeks 1-8	Participants in RP and SP will have comparable intensity of craving during 1) the inpatient treatment period, and 2) during the first week after the first XR-NTX injection.<br>Significance/Rationale: Earlier trial showed comparable craving severity in both procedures (Sullivan *et al.*, 2017).
Opioid withdrawal (SOWS and COWS) over time	Mean for opioid withdrawal measured by SOWS and COWS score, daily (starting with day of admission) during days leading to the first XR-NTX injection and during post-induction Weeks 1-8	Participants in RP and SP will have comparable severity of opioid withdrawal during: 1) the inpatient treatment period, and 2) during the first four weeks after the first XR-NTX injection.<br>Significance/Rationale: Earlier trial showed comparable withdrawal severity in both procedures (Sullivan *et al.*, 2017).
Receive second and third injections (binary: did or did not receive second and third dose of XR-NTX)	Proportion of participants that receive second and third injection of XR-NTX (at 4 weeks and 8 weeks, from first injection)	Participants in RP and SP will have comparable rates of treatment retention.<br>Significance/Rationale: Because there is no difference in tolerability of the first and second XR-NTX injections in both study arms, we do not expect differential treatment dropout.
Targeted safety events, overdoses and SAEs, related to study medications	Frequency of targeted safety events, overdose episodes and SAEs by relationship to study medication during the induction period and during eight weeks of post-induction	RP and SP will produce equivalent rates of targeted safety events and SAEs during the induction and during the first eight weeks of treatment with XR-NTX.<br>Significance/Rationale: Careful documentation of SAEs, targeted safety events, and overdose

Outcome	Outcome Measure	Hypothesis
	treatment	episodes, would be considered essential safety data, and important component of a comparative effectiveness trial.
Use of opioids over time during the 8-week of post-induction treatment while on study medication (Weekly TLFB, confirmed by urine drug screens when available)	Percent of participants positive for opioids using weekly TLFB during eight weeks of post-induction, and urine drug screens at week 4 and 8.	RP and SP will produce comparable levels of opioid use.<br><br>Significance/Rationale: XR-NTX produces complete blockade of opioid effects, so that during treatment with monthly injections, opioid use can be expected to be minimal and no different between study arms.

7.3 Implementation Process and Outcome Measures

7.3.1 Identifying Facilitators and Barriers to Implementation Success

Both quantitative and qualitative measures will be collected on factors hypothesized to be related to implementation success. Measures of implementation process capturing elements organized by the domains in the Consolidated Framework for Implementation Research (CFIR; (Damschroder and Hagedorn, 2011)) were used to guide measurement selection. This framework identifies the domains of intervention characteristics (e.g., complexity, relative advantage), inner setting (e.g., readiness, clinical leadership), individual characteristics (e.g., provider beliefs, self-efficacy), and implementation process (e.g., fidelity and feasibility) as necessary to understand the factors contributing to and barriers preventing implementation success. Table 4 summarizes these domains and the associated measures applicable to this trial.

7.3.2 Table 4: Implementation Measures Organized by the Consolidated Framework for Implementation Research Constructs

Construct	Measure	Data Sources (Timeline)
Intervention Characteristics	Intervention Source<br><br>Evidence Strength & Quality<br><br>Relative Advantage<br><br>Adaptability<br><br>Complexity<br><br>Design Quality and Packaging<br><br>Cost	ORCA (Before and after each wedge intervention)<br><br>Staff Structured Interviews (After each wedge intervention and study conclusion)<br><br>Coaching Session Progress Notes<br><br>Internal Implementation Team Meeting Notes<br><br>NMS Assessments and Staff Structured Interviews
Outer Setting	Patient Needs & Resources<br>Peer Pressure<br><br>External Policy & Incentives	Site Mapping Needs Assessment and Progress Notes (Pre-implementation phase before each wedge intervention)<br><br>Coaching Session Progress Notes<br><br>Staff Structured Interviews (After each wedge

Construct	Measure	Data Sources (Timeline)
		intervention and study conclusion)
Inner Setting	Structural Characteristics<br>Culture<br><br>Implementation Climate (Tension for Change, Compatibility, Relative Priority)<br><br>Learning Climate<br><br>Readiness for Implementation (Leadership Engagement, Available Resources)	Site Mapping Needs Assessment and Progress Notes (Pre-implementation phase before each wedge intervention)<br><br>Coaching Session Progress Notes<br><br>Internal Implementation Team Meeting Notes<br><br>ORCA (Before and after each wedge intervention)<br><br>Readiness/preparedness rulers (Before and after each wedge intervention)<br><br>Staff Structured Interviews (After each wedge intervention and study conclusion)
Individuals	Knowledge and Beliefs about the Intervention<br><br>Self-efficacy<br><br>Individual Stage of Change<br><br>Individual Identification with Organization<br><br>Other Personal Attributes	ORCA (Before and after each wedge intervention)<br><br>Readiness/preparedness rulers (Before and after each wedge intervention)<br><br>Staff Structured Interviews (After each wedge intervention and study conclusion)
Process	Planning<br><br>Engaging<br><br>Executing<br><br>Reflecting & Evaluating	Critical Action Checklists (Throughout trial, weekly)<br><br>Implementation Process Fidelity Checklist (Throughout trial, monthly)<br><br>Daily Medication Administration Log (Throughout trial, daily)<br><br>Coaching Session Progress Notes (Throughout trial, weekly or more frequently based on site needs)<br><br>Internal Implementation Team Meeting Notes (Throughout trial as needed)<br><br>Staff Structured Interviews (After each wedge intervention and study conclusion)<br><br>Patient End of Induction Survey (At end of detox admission)<br><br>Patient Treatment Satisfaction Survey (At trial completion)<br><br>Organization Level Clinical Implementation Data Form (Before and after each wedge intervention)

7.3.3 Implementation Related Outcome Measures

In addition to the above measures considering factors impacting successful implementation, implementation-related outcome measures will also be collected. These will be organized according to the RE-AIM framework, designed to enhance the public health impact of research evidence through coherent organization of outcomes related to implementation (Glasgow *et al.*, 1999).

This framework includes the domains of: **Reach** to the intended target population, **Effectiveness**, **Adoption** by target staff and institutions, **Implementation** consistency (including fidelity to the Intervention), and **Maintenance** of the intervention over time by the target providers. The following

table describes study related measures organized according to this framework:

7.3.4 Table 5: Implementation Outcome Domain

Implementation Outcome Domain	Specific Study Measures
Reach	Clinic level data on percent of individuals with OUD screened for trial participation, percentage of individuals with OUD who receive XR-NTX during inpatient detox or residential rehab.
Effectiveness	Secondary clinical outcomes related to effectiveness will be gathered as described above (Table 3).
Adoption	An implementation checklist will capture all planned implementation processes, including the number of providers participating in each implementation activity.
Implementation	Fidelity to suggested clinical guidelines for trial participants will be gathered for each patient throughout the detoxification and rehabilitation stay via a critical action checklist.
Maintenance	Qualitative data (structured progress notes and staff interviews with key personnel) will capture barriers and facilitators that inform modifications and adaptations to implementation strategies to support long term adoption of the study procedures.

7.3.5 Integration Process for Iterative Improvement of Implementation Facilitation

All implementation measures will be analyzed by categorizing individual facilitators and barriers within the framework of the CFIR model. These measures will be assessed after each stepped wedge across various qualitative data including structured notes from Site Mapping meetings and coaching sessions, internal implementation team meeting notes, and staff structured interviews at the end of each stepped wedge.

Structured progress notes will contain sections for identified barriers and facilitators captured through site meetings and internal implementation team discussions. In addition, any modifications or adaptations made throughout the step wedge will be documented using these structured progress notes. Structured interviews with clinical staff and participants will be digitally

recorded, transcribed, and coded based on the CFIR framework using Atlas.ti software (i.e., intervention characteristics, patient/provider characteristics, internal setting factors, and external setting factors). Qualitative data analysis will involve conceptual mapping of identified barriers and facilitators across the CFIR framework), and will inform modifications and adaptations to implementation strategies and the IF package. Modifications and adaptations to implementation strategies will be categorized using the FRAME-IS model, the ERIC taxonomy for naming of implementation strategies, and Proctor *et al.*'s guidance on specifying implementation strategies. This systematic approach will be used to make improvements on implementation procedures and contribute to an improved implementation package at the trial conclusion

8.0 STUDY POPULATION

Study participants are treatment-seeking heroin- and/or prescription opioid-dependent volunteers, without chronic pain requiring opioid therapy, who are willing to undergo opioid withdrawal and accept relapse prevention treatment with XR-NTX. The target recruitment is approximately 75 participants per site or approximately 450 participants in total across 6 sites, with approximately 225 participants treated using SP and approximately 225 participants treated using the RP.

8.1 Participant Inclusion Criteria

Study participants must meet all of the following inclusion criteria in order to be eligible to participate in the study:

1. 18 years of age or older.
2. Meets current DSM-5 criteria for opioid use disorder.
3. Seeking treatment for opioid use disorder, willing to accept treatment with XR-NTX and, in the judgment of the treating physician, is a good candidate for naltrexone- based treatment.
4. Willing and able to provide written informed consent.
5. Able to speak English sufficiently to understand the study procedures and provide written informed consent to participate in the study.
6. If female of childbearing potential, willing to practice an effective method of birth control for the duration of participation in the study.

8.2 Participant Exclusion Criteria

All individuals meeting any of the following exclusion criteria will be excluded from study participation:

1. Serious medical, psychiatric or substance use disorder that, in the opinion of the study physician, would make a detoxification and naltrexone initiation, or maintenance treatment with XR-NTX, hazardous (relative contra-indications) or requires a different level of care. Examples include:
   a) Disabling or terminal medical illness (e.g., uncompensated heart failure, severe acute hepatitis, cirrhosis or end-stage liver disease) as assessed by medical history and/or review of systems.
   b) Severe, untreated or inadequately treated mental disorder (e.g., active psychosis, uncontrolled manic-depressive illness) as assessed by history and/or clinical interview.
   c) Current severe alcohol, benzodiazepine, or other depressant or sedative hypnotic use likely to require a complicated medical detoxification (routine alcohol and sedative detoxifications may be included).
   d) Suicidal or homicidal ideation that requires immediate attention.

2. Known allergy or sensitivity to buprenorphine, naloxone, naltrexone, polylactide-co-glycolide, carboxymethylcellulose, or other components of the Vivitrol® diluent.
3. Maintenance treatment with methadone within 14 days of consent.
4. Maintenance treatment with buprenorphine unless the patient is determined to have a poor treatment response (in the form of buprenorphine non-adherence with or without the use of illicit opioids), warranting change to XR-NTX treatment.
5. Presence of pain of sufficient severity as to require ongoing pain management with opioids.
6. Circumstances (legal, personal, occupational) that would threaten the feasibility of XR-NTX treatment or make another treatment (e.g., buprenorphine or methadone) a better choice.
7. Are currently in jail, prison or other overnight facility as required by court of law or have pending legal action that could prevent participation in study activities.
8. If female, currently pregnant or breastfeeding, or planning on conception.
9. Body habitus that, in the judgment of the study physician, precludes safe intramuscular injection of XR-NTX (e.g., BMI>40, excess fat tissue over the buttocks, emaciation).
10. Admitted to the inpatient detoxification or residential rehabilitation unit more than 4 calendar days prior to enrollment into to SP or RP.

8.3 Special Populations to Consider

This study is likely to enroll persons involved in the criminal justice system, many of whom are expected to be in treatment at the clinical sites participating in this study. The study will exclude persons incarcerated/detained in a correctional facility, but will not exclude parolees, probationers, or persons in sentencing diversion or drug court programs who are enrolled at participating sites. Some of these subjects may be classified as prisoners per 45 CFR 46 Subpart C.

8.4 Strategies for Recruitment and Retention

Recruitment efforts will vary per site and may broadly include site staff education and distribution of study materials, community or participant-level outreach and advertisements, and the encouragement of word-of-mouth referrals among site patient populations. During (or prior to) the first four calendar days of an inpatient admission, clinical and/or research staff will evaluate all admitted patients for XR-NTX and provide information about the study to all patient-participants that express interest in treatment with XR-NTX.

9.0 SITE SELECTION

9.1 Number of Sites

The study is conducted at six sites that will be recruited from the NIDA National Drug Abuse Treatment Clinical Trials Network (CTN). Each of the sites will be expected to enroll approximately 1-2 eligible participant(s) per week over the 70 weeks recruitment period for the total N=450 enrolled.

9.2 Site Eligibility

To be included in the study, the selected sites must have the following characteristics:

1. Provide opioid detoxification services (inpatient/residential) where participants can be maintained opioid-free.
2. Able to provide treatment in hospital or residential treatment for at least the 14 days needed to carry out the SP initiation regimen.
3. Have the capacity to provide ongoing treatment with XR-NTX on an outpatient basis for at least 2 months after the induction and administer subsequent XR-NTX injections.
4. Have the capacity to provide outpatient care including medical management and counselling (at least one group and/or individual counseling session per week).
5. Have the capacity to provide psychiatric evaluation and either offer treatment for co-occurring psychiatric disorders or provide linkage to psychiatric care.
6. Currently offering XR-NTX induction using buprenorphine taper and a wait period for individuals entering treatment with active substance use and requesting XR-NTX.
7. Have no experience initiating patients onto XR-NTX using a RP.
8. Agree to implement the suggested RP as the induction method for patients attempting to transition from active opioid use to XR-NTX using the implementation regimen included in the study.
9. Have an adequate flow of patients entering treatment as to provide a sufficient population of potential participants to achieve study enrollment goals. We predict that with 3-5 patients admitted each week for "detoxification," at least 1 participant per week should be interested in XR-NTX induction and be eligible to enroll into the study (other will initiate agonist maintenance).
10. Have leadership desiring to adopt XR-NTX initiation (SP and RP) as routine clinical practice for patients requesting detoxification.
11. Have the ability to provide deidentified information on individuals who were admitted to the unit during the study period but were not enrolled in the study.
12. Can provide space and resources for up to 6 study-supported staff.
13. With support from the study budget, able to enroll participants who do not have resources or insurance coverage to pay directly or through a third party for treatment.

9.3 Rationale for Site Selection

The volume of patients admitted to the unit must be adequate to ensure a steady stream of eligible patients for recruitment. A diverse, representative sample of patients and unit characteristics is desirable to enhance the external validity of the study. Geographical diversity of settings is desirable and research experience and successful participation in prior studies is essential as it provides evidence of the feasibility of study implementation.

Clinical experience using the standard method of XR-NTX induction with buprenorphine taper followed by a waiting period before XR-NTX induction is the standard of care in many treatment programs. Including only programs with experience with this method will allow for an appropriate comparison between the RP and SP methods of induction without the need for significant implementation or training to provide the SP. At the same time, we will not include sites experienced with the RP. Reduction of site variance in this research study will improve the power in statistical analysis to detect an effect for the primary outcome.

The research staff will preferably have experience working in the addiction treatment setting and therefore can be quickly integrated into the team.

10.0 STUDY PROCEDURES

Schematic of study phases is presented in Figure 1.

10.1 Pre-screening, Consent and Screening (Phase 1)

Participants will be recruited from the population of individuals presenting for treatment of opioid use disorder at the participating study sites.

Pre-screening will be completed before participants are approached for informed consent. All patients admitted to the unit with an OUD diagnosis should be pre-screened for the study and entered on the Participant Pre-Screening Log as well as on the pre-screening enrollment form in Advantage eClinical. The following will be assessed at pre-screening: current and active OUD, demographics, eligibility to receive XR-NTX and willingness to participate in the study.

All participants who are interested may begin the informed consent procedures up to 7 calendar days prior to the inpatient admission or during the first four calendar days of admission. All participants must sign the informed consent and complete screening procedures prior to the fourth calendar day of admission to be eligible to enroll in the Rapid Procedure or Standard Procedure. The timing of consent and screening procedures is flexible, and allows sites to customize recruitment, consent, and screening procedures to accommodate local conditions. Obtaining consent and screening/baseline data early in the admission will allow a full accounting of persons interested in study enrollment. For individuals consenting after admission, data will be collected about medication administration starting from admission using medication flowsheets and EMR abstraction (performed and confirmed by study staff).

10.1.1 Informed Consent Procedures for Participants

Candidates for the study are given a current IRB-approved copy of the Informed Consent Form to read. Appropriately qualified and trained study personnel explain all aspects of the study in lay language and answer all the study candidate’s questions. Study procedures and the potential risks and benefits of participating in the trial will be explained. Staff will be available to answer questions about the consent form while participants are reviewing it.

Prior to signing the consent form, the participant must pass a brief consent quiz to illustrate comprehension of the study activities - understanding of the project, the purpose and procedures involved, and the voluntary nature of his/her participation. Those who cannot successfully answer quiz items have the study re-explained by research staff with a focus on aspects they did not understand. Those who demonstrate understanding of the study and voluntarily agree to participate are asked to sign the Informed Consent Form. After passing the quiz and signing the consent form, participants will be offered a copy of the forms to keep for their records. The informed consent process and quiz will take approximately 30-45 and 5 minutes to complete, respectively.

Given the multi-site nature of the trial, it is possible that ancillary studies, or long term follow up studies, will be proposed before or after the study begins recruitment. For this reason, during the informed consent process, we also will seek permission to contact the participant in the future

about other study opportunities.

Participants are not administered any study assessments or procedures prior to signing informed consent. The informed consent includes a HIPAA Authorization to permit study staff to access the participant's medical records at the study site relating to the inpatient admission; the purpose of this is to capture study related clinical information that is collected during routine treatment (daily vitals, daily COWS, medication administration log, etc.).

10.1.2 HIPAA Authorization and Medical Record Release Forms

Study sites may be required by their institutions to obtain authorization from participants for use of protected health information (PHI). Sites will be responsible for communicating with the IRB of Record or any local Privacy Boards and obtaining the appropriate approvals or waivers to be in regulatory compliance.

10.1.3 Screening/Baseline Visit

Screening and baseline assessments are detailed in Section 12 and capture participant demographics; medical, psychiatric, drug use, and treatment history; quality of life; and current health status. Urine testing will also be performed. Following obtainment of consent and the final confirmation of eligibility, participants are considered enrolled into SP or RP and begin completing baseline assessments.

10.2 Treatment Initiation

To maximize generalizability, this study is designed to permit consent of participants before and during the first four calendar days of inpatient treatment (Day 1 is day of admission). Patients judged clinically to be good candidates for detoxification and XR-NTX induction will, upon admission to the unit, begin treatment with non- opioid medications (mainly clonidine) and buprenorphine will be administered to address withdrawal symptoms as they emerge. Sites will agree to this regimen as their standard practice for the first four calendar days of inpatient-residential treatment. During the first four calendar days of admission, patients will have the opportunity to consent to the study, and concurrent treatment will follow the SP or RP, depending on which procedure the site is offering at that time per the study design.

10.3 Randomization

Randomization will be performed at the site level. Six sites that have the required characteristics to be included in this study will be randomized to the order in which they begin to implement the RP. Patient-participants will be treated with the SP or RP according to which procedure is being used at the site at the time the patient is admitted.

The randomization of each site will follow the stepped-wedge design (Table 1). At the selected time points as described in the Study Timeline section , one randomly selected site will make a "step" and will cross-over from the standard to the Rapid XR-NTX induction. Note that the first randomized site will be in the Rapid Procedure arm for the entire duration of the study whereas the 6th site (after 5 sites have been randomized to the Rapid Procedure) will be in the Standard Procedure arm for the entire duration of the study and will never switch to the Rapid Procedure.

The DSC statistician will conduct the randomization and inform the sites eight weeks before the selected site is scheduled to transition.

10.4 Inpatient Treatment/Intervention (Phase 2)

Participants are inducted onto their assigned XR-NTX induction regimen: Standard (Table 6A) or Rapid (Table 6B). These regimens are guidelines to be applied by the clinical teams with coaching from the lead team and may be modified by the site clinician based on the clinical status of each patient.

10.4.1 Standard Induction Procedure (SP)

SP includes stabilization on buprenorphine (6-8 mg) on Day 1 followed by a taper over the subsequent 4 days (Table 6A), however this schedule may be modified by the site clinician to suit the clinical status of each patient. Depending on the level of physical dependence, time from the last dose of an opioid, type of an opioid (prescription opioid, heroin, or fentanyl), and the severity of withdrawal, participants may be required to wait additional time and receive symptomatic treatment until it is safe for them to start taking buprenorphine.

After the completion of buprenorphine taper, participants will enter a washout period of at least 8 days. Adjunctive (non-opioid) medications will be administered during the washout period, as needed, to alleviate significant withdrawal symptoms, with choice of medications and doses as routinely used at the site. On the last day of the taper, participants will be evaluated for eligibility to receive XR-NTX injection, as it is routinely used at the site. This may include a naloxone challenge (e.g., 0.8-1.2 mg IM), a naltrexone titration (e.g., 25 mg PO), and/or a urine toxicology negative for buprenorphine and other opioids (as in CTN-0051). Once found eligible, an XR-NTX injection will be given.

10.4.2 Table 6A. Standard Induction Procedures

Day 1	Participants will receive buprenorphine 6 mg. If continue to experience significant withdrawal (COWS >6) they can receive additional 2-4 mg.
Day 2	Buprenorphine 6 mg in AM
Day 3	Buprenorphine 4 mg in AM
Day 4	Buprenorphine 2 mg in AM
Day 5	Buprenorphine 1 mg in AM
Days 6-13	Adjunctive medications as needed (i.e., clonidine and clonazepam)
Day 13 or 14	XR-NTX eligibility determination followed by the XR-NTX injection

10.4.3 Rapid Induction Procedures (RP)

RP includes 1 day of buprenorphine 6-8 mg, followed by a day of washout and 4 days of oral naltrexone titration, however this schedule may be modified by the site clinician to suit the clinical status of each patient. Implementation facilitation will train sites to provide detoxification based on the following procedures:

Conditions for initiating buprenorphine and dosing schedule in RP on Day 1 are similar to the Day 1 in the SP guidelines. Naltrexone should be administered starting with Day 3, according to the schedule outlined in Table 6B. On Day 3 naltrexone will be administered starting with 0.5 mg dose, 1 hour after pretreatment with adjunctive medication. COWS will be administered 2 hours after naltrexone and, if there is no increase in withdrawal (change in COWS score is less than 3) the next dose scheduled for this day (e.g., another 0.5 mg on Day 3) will be administered. The same procedure will be repeated on Days 4-6. Table 6B provides a guided timeline for RP, however, induction procedures should be modified to suit the clinical status of each patient and determine eligibility to administer XR-NTX.

10.4.4 Table 6B. Rapid Induction Procedure Guidelines

Day 1	Participants will receive buprenorphine 6 mg. If continue to experience significant withdrawal (COWS >6) they can receive additional 2-4 mg. Adjunctive medications will be initiated if necessary, to alleviate residual withdrawal persisting after administration of buprenorphine 10 mg.
Day 2	Standing doses of clonidine and clonazepam, with additional adjunctive medications as needed*.
Day 3	Standing doses of clonidine and clonazepam, with additional adjunctive medications as needed*.<br>Following the first morning dose of adjunctive medication, participants will receive naltrexone 0.5 mg. If there is no significant increase in withdrawal over the subsequent 120 minutes (COWS increase < 3) they will receive additional naltrexone 0.5 mg.
Day 4	Standing doses of clonidine and clonazepam, with additional adjunctive medications as needed*.<br>Following the first morning dose of adjunctive medication naltrexone 1 mg. If there is no significant increase in withdrawal over the subsequent 120 minutes (COWS increase < 3) they will receive additional naltrexone 1 mg.
Day 5	Standing doses of clonidine and clonazepam, with additional adjunctive medications as needed*.<br>Following the first morning dose of adjunctive medication they will receive naltrexone 3 mg. If there is no significant increase in withdrawal over the subsequent 120 minutes (COWS increase < 3) they will receive additional naltrexone 3 mg.
Day 6	Standing doses of clonidine and clonazepam, with additional adjunctive medications as needed*.<br>Following the first morning dose of adjunctive medication they will receive naltrexone 6 mg. If there is no significant increase in withdrawal over the subsequent 4 hours (COWS increase < 3) participant will receive an injection of XR-NTX 380 mg IM. For those who were not able to tolerate naltrexone 6mg, please refer to Day 7 procedure.
Day 7	Standing doses of clonidine and clonazepam, with additional adjunctive medications as needed*.<br>Participants that were not able to tolerate naltrexone 6 mg during Day 6 will have procedures from Day 6 repeated.
****Adjunctive medication to be administered during days 1-7***	• clonidine 0.2 mg every 4 hours (lower or withhold the dose if SBP<90 or HR<50), MDD=1.2 mg/d<br>• clonazepam 1 mg every 6 hours (withhold the dose if the patient is difficult to

	arouse), MDD=4 mg/d<br>• trazodone 100 mg at night *as needed* for insomnia<br>• prochloperazine 10 mg every 8 hours *as needed* for nausea<br>• zolpidem 10 mg at night *as needed* for insomnia<br>• ibuprofen 600 mg po every 8 hours *as needed* for muscle pain

The XR-NTX injection is administered on Day 6 after naltrexone dose of 6 mg is received and tolerated over the 4 hours post-dose monitoring period (change in COWS score is less than 3).

In the RP Order Set, adjunctive medication will be written to be administered as standing doses and withheld only in case of intolerance, excessive sedation or patient's request. Adjunctive medications will include clonazepam, up to 4 mg/day, and clonidine, up to 1.2 mg/day.

Staff on units providing rapid induction will be trained to offer psychoeducational counseling, conducted by clinical staff, on a daily basis. The goals of these counseling sessions are as follows: (1) To educate and support the participant as they transition to XR-NTX to improve tolerability of the induction; (2) Provide education on opioid withdrawal signs and symptoms and discuss pharmacological and nonpharmacological strategies for the management of opioid withdrawal;(3) Provide education regarding any medication side effects with a goal to increase adherence and correct use of medications, and (4) Increase participant's motivation to adhere to the regimen and to complete the transition to XR-NTX.

10.5 Post-Induction Treatment (Phase 3)

Following the administration of the first XR-NTX dose or induction failure, participants will be encouraged to continue follow-up treatment per standard clinical practice. The sites should provide outpatient care as clinically appropriate and are recommended to provide participants with at least weekly clinic visits during the first month of treatment then biweekly visits during the second month. Participants will also complete research ratings, including self-report of withdrawal, mood, and drug use as noted in Section 12. The participant provides a urine sample at each injection visit for an onsite toxicology testing.

10.5.1 Psychosocial Counseling

Psychosocial counseling consists of outpatient counseling provided at the CTP. Selected sites agree to provide outpatient care that consists of at least one group and/or individual counseling session. Data is collected from the clinic record or from the participant on counseling sessions attended. Participation in counseling sessions is voluntary. Failure to attend counseling sessions will not be considered a reason to be excluded or withdrawn from the trial.

10.5.2 Medical Management

During the 8-week course of post-induction treatment, participants receive two additional injections of XR-NTX (Vivitrol®) 380 mg, at 4 and 8 weeks after the first injection. All sites will be provided with training in providing Medical Management (MM) clinical support to be provided by the same study clinicians (e.g., MD, DO, PA, NP, RN/LPN; working within the scope of their local licensure, clinical privileges and/or scope of practice) in unblinded fashion.

Briefly, MM sessions focus on establishing and maintaining patient-clinician rapport and partnership, education surrounding opioid addiction and treatment, establishing and maintaining a plan for XR-NTX adherence, advice and encouragement to maintain abstinence, monitoring medication side effects and dose adjustments, and support for ancillary treatment, including psychosocial counseling, 12-step involvement, and further community-based treatment. MM also provides guidelines for assessment and management of relapse in both arms.

In keeping with a pragmatic, community-based comparative effectiveness trial, MM is broadly guided by provider training prior to study start and regular MM provider calls. Suggested guidance for providing post-induction follow up can be found in Module 6 – Treatment & Follow-Up of the MOP, but is not subject to rigorous quality assurance procedures (i.e., audiotaping, manualization and QA audits). Suggested MM frequency is weekly until week 4 then biweekly until week 8. XR-NTX is injected at the MM visit at Week 4 and at Week 8.

10.5.3 Rapid Procedure: Adjunctive Medication Taper

On the day of discharge, following the XR-NTX injection, participants in RP arm will receive a prescription for tapering doses of adjunctive medications for 7 days. The study guidance for this taper is Day 1-2: clonidine 0.1 mg 4xday, clonazepam 0.5 mg 2xday; Day 3-4: clonidine 0.1 mg 3xday, clonazepam 0.25 mg 3xday; Day 5-6: clonidine 0.1 mg 2xday, clonazepam 0.25 mg 2xday; Day 7-8: clonidine 0.1 mg 1xday, clonazepam 0.25 mg 1xday). Participants will also receive zolpidem 10 mg at night *as needed* for insomnia during the first week. Flexibility and clinical judgment should be used in providing this taper based on the needs of the individual patient.

10.5.4 XR-NTX Injections

XR-NTX (4cc, ~380mg of naltrexone base) is administered approximately every four weeks, in the form of Vivitrol®. XR-NTX injections may take place more often than 4 weeks apart if there is clinical concern about non-adherence (participant is inconsistent in attending scheduled visits) or if clinical observation is that opioid craving and/or use re-emerge during the 4th week after the last injection; however, time between injections must always be at least 21 days. XR-NTX is administered by a deep intramuscular injection to the buttocks (alternating sides) according to the injection preparation and administration procedures specified in the Vivitrol® product package insert. These procedures are designed to minimize the risk of injection site reactions. XR-NTX is provided by the study at no cost to the participant.

10.5.5 Handling of XR-NTX Induction Procedure Intolerance

Participants who are unable to tolerate the study-specific procedures will have the option to remain in the study with modification to the regimen based on the clinical judgment of the treatment providers. For example, a buprenorphine taper duration in a SP can be extended or a naltrexone titration in RP can be held. If adjustment is not sufficient, induction is not achieved within 30 days of admission or at any point when participants are no longer willing to continue with XR-NTX induction, participants will be considered an induction failure and should be managed as clinically appropriate by the unit with encouragement of continuation of medication for opioid use disorder – i.e., stabilized on buprenorphine or methadone and referred for ongoing outpatient

medication maintenance treatment. The goal is to minimize the chance that participants leave the hospital without medication to prevent relapse. Participants who fail to induct XR-NTX while inpatient will remain in the study and be asked to return only for week 4 and week 8 study visits. Induction failures will still be allowed to receive study supplied XR-NTX until study completion at week 8.

10.5.6 Handling of Missed XR-NTX Doses, Lapses, and Relapses

Use of illicit opioids may be of a concern in patients receiving XR-NTX. Because of the long duration of action of XR-NTX (full blockade out to 5 weeks after the last injection), a grace period of 7-21 days can be expected during which the injection can be rescheduled, provided the participant has not relapsed and become re-dependent. Relapse is defined as daily use of illicit opioid for 7 or more days that starts after 35 or more days after the XR-NTX injection. If the participant misses a scheduled injection and does not otherwise meet the relapse criteria, XR-NTX should be administered as soon as possible, following a negative naloxone challenge (if clinically indicated). The participant is eligible to receive the 2nd and 3rd XR-NTX injection from study supply up to 21 days from the target Week 8 visit (i.e. up to 77 days from 1st XR-NTX).

10.5.7 XR-NTX Discontinuation Post-Induction

XR-NTX may be discontinued in the event of intolerable side effects or safety concerns preventing further medication treatment (e.g., pregnancy), relapse where restarting XR-NTX is not possible, or the end of the active treatment phase. Participants discontinuing medication prior to Week 8 continue within the same study assessment schedule. All participants who end treatment early (prior to Week 8) are encouraged to complete an End of Medication Treatment visit. In all cases of treatment discontinuation, the research and clinical team will make an effort to arrange for continued community treatment, as appropriate and available, including further XR-NTX and Buprenorphine-Naloxone (Suboxone®) (BUP-NX), or methadone maintenance and intensive outpatient psychosocial aftercare. For participants who do not wish to continue with XR-NTX, or for whom community resources to continue with XR-NTX are not available, the study will make alternative arrangements to make sure there is no gap in providing medication-based treatment (e.g., buprenorphine or methadone) as clinically appropriate to minimize the risk of relapse and related adverse outcomes such as an overdose.

10.5.8 Ancillary Medications

Participants who experience protracted withdrawal symptoms, sleeplessness, and/or depressive symptoms may be treated with ancillary medications. Depression is common in opioid-dependent patients and may adversely affect prognosis of naltrexone treatment. Participants who show depressive symptoms may be treated with antidepressants and/or referred for psychiatric evaluation and treatment. In general, psychiatric or medical problems emerging during the study treatment period are handled by the CTP according to their usual practices for treatment and referral.

10.5.9 Criminal Justice Involvement

This study includes subjects who may be classified as prisoners per 45 CFR 46 Subpart C. If a subject becomes incarcerated during the study, treatment and follow-up procedures may be continued in accordance with local IRB approvals. Procedures must be compliant with 45 CFR 46 Subpart C. Data may be collected either in person, by phone, in writing, and/or by electronic means, provided that data collection follows the procedures approved by OHRP, the IRB of record, and any local regulatory authorities. Details of the nature of the research will not be shared with staff at the jail/prison, and visits, whether in person or by phone, will only be conducted if the participant's confidentiality can be maintained and no audio-taping occurs.

10.6 End of Study

Participants will have their last study visit at the end of post-induction Week 8. An effort will be made to assess all participants at Week 8 post-induction or induction failure. P55 assessments will be completed at that time.

Aggressive outreach procedures will be implemented to locate and assess all study participants to minimize missing data.

10.7 Disposition and Referral (Phase 4)

During the last month of study, as part of standard clinical care, arrangements will be made by the clinical team for participants to either continue treatment at the same program or be referred to another program in the community for follow-up care, which should ideally include continuation of XR-NTX. The third XR-NTX injection will be administered as a part of the study in order to objectively assess the retention in treatment 8-weeks after the induction and to assure the continuation of treatment with XR-NTX during the post-study transition period. However, participants will not be followed with study assessments after third injection as at this point, they will be engaged with and followed in the community-based clinical care where they will be assessed medically following standard procedures. The disposition plan may include continuing treatment with XR-NTX or another medication in the study clinic or in a different clinic in the community. Participant may also be referred to another CTN study where long-term outcome of treatment with XR-NTX is evaluated (e.g., CTN-0100).

10.8 Study Intervention Adherence

As part of the implementation assessment noted below adherence to study procedures on the inpatient units will be monitored via review of nursing flowsheets and order sets. Once participants begin the post-induction phase, participant adherence (i.e. retention in treatment during post-induction phase) to XR-NTX injections will be tracked as part of a secondary outcome measure.

10.9 Premature Withdrawal of Participants

All participants will be followed for the duration of the study unless they withdraw consent, die, or the investigator or sponsor decides to discontinue their enrollment for any reason. The participant may be discontinued from the study if the participant, the investigator, or the sponsor determines that it is not in the best interest for the participant to continue in the study. Individuals who fail to

achieve induction will continue to be followed as part of the study for all secondary outcomes until discharge and will be assessed at Week 4 and Week 8 for the end of study visit. Reasons for study discontinuation may include, but are not limited to:

- Withdrawal by participant
- Lack of efficacy
- Relapse
- Participant becoming a threat to self or others in the opinion of the investigator
- Lost to follow-up
- Non-adherence to study medications or procedures in the opinion of the investigator
- DSMB early termination of the study for safety or effectiveness reasons
- Lack of funding

In the event that a participant chooses to withdraw from the study, the investigator will make an effort to ascertain the reason for withdrawal and be encouraged to continue with medication assisted treatment. Enrolled participants will be asked to return to the clinic for an Early Medication Termination visit. If the participant fails or refuses to return to the clinic an attempt must be made to contact the subject by phone or mail, and attempts must be documented. Participants who withdraw from the trial will be offered treatment referrals as clinically appropriate. The reason for study discontinuation will be documented on the appropriate case report form.

10.10 Study Termination

The sponsor may close the investigational sites or terminate the study at any time. The Investigator may initiate site closure at any time, provided there is reasonable cause and sufficient notice is given in advance of the intended termination. Reasons for the early closure of an investigational site by the Sponsor or Investigator, or termination of a study by the sponsor, may include but are not limited to:

- failure of the Investigator to comply with the protocol
- safety concerns
- ethical issues
- inadequate recruitment of participants by the Investigator.

Study termination will be promptly reported to IRB, DSMB and NIDA.

10.11 Blinding

This study is a pragmatic, open-label clinical trial. Treatment assignment and active medication assignment is known to all staff and all participants.

10.12 Participant Reimbursement

Study participants who cannot access treatment or specific study medications at participating programs due to reimbursement restrictions will be provided with pharmacotherapy, medication, and medical management at no cost. However, the study will not cover the cost of detoxification or residential treatment stay.

In addition, participants are reimbursed with cash, or vouchers of equivalent value, to offset costs of time and travel and to provide modest incentives for attending study visits. This study does not employ contingency management targeting abstinence or specifically targeting medication adherence (in other words, reimbursement is earned for presenting to scheduled visits and completing assessments and medication management visits if applicable).

Participants will be reimbursed for participation in this study. Reimbursement will be in accordance with the IRB of record's policies and procedures, and subject to IRB approval. If a participant is found to be incarcerated at time of follow-up, the participant is reimbursed the agreed upon amount as approved by the IRB of record and/or collaborating prison facilities.

10.13 Retention Plan

Retention in treatment during the induction phase and receipt of XR-NTX is a central focus of the intervention used in the present study and the primary outcome of the randomized study. All participants, regardless of induction success or failure status, will be strongly encouraged to remain in treatment even if they choose to leave the study.

11.0 MEDICATION PACKAGING/HANDLING/STORAGE / ACCOUNTABILITY

11.1 Study Medication Sources

Study will provide XR-NTX (Vivitrol®) to all sites to avoid the issue of inability to fund treatment, which is an issue independent of the study objectives (how to induct onto XR-NTX). It is expected that all units will be able to provide all other medications, including low-dose oral naltrexone and buprenorphine.

11.2 Study Medication Management

Each research site is required to observe local, state, and federal regulations regarding receipt, custody, dispensing, and disposition of all study medications. Each site will maintain an adequate supply of unexpired study medications on site. Medications will be supplied to sites by the NIDA contractor.

11.3 Medication Accountability Records

Appropriately qualified and trained study personnel maintain accurate and current accounting of all study medication by utilizing drug accountability records which are made available for review by study monitors and other appropriate research personnel.

Accurate drug accountability records:

- Demonstrate that the study medication was dispensed according to the protocol.
- Document receipt of the study medication, date, lot #, expiration date, quantity and dosage.
- Account for unopened, un-dispensed, unused, returned, wasted or broken medication.
- Dosing logs should record participant ID #, date dispensed, drug name, lot # and amount dispensed.
- Indicate who dispensed or handled the study medication.
- Temperature logs should show a daily record of medication storage temperature.

11.4 Dispensing of Study Medication

All study medications shall be prepared and dispensed by a pharmacist or licensed medical practitioner appropriately trained and authorized to dispense study medications per local regulations.

11.5 Study Medication Storage

Study medication should be stored in compliance with federal, state, and local laws and institutional policy. Study medication is stored in a secured location under the conditions specified by the package insert(s).

11.6 Used/Unused Medication

Study medication returned by a participant may not be re-issued for use. Unused study medication is returned and logged into a perpetual inventory of study medication returned. Damaged, returned, expired, or unused study medication is accounted for by the NIDA contract monitor and may be sent to the central distributor or a reverse distributor for destruction. Expired XR-NTX may be destroyed on-site per local institutional policies following a complete accounting by the NIDA contract monitor.

11.7 Lost Medication

At the discretion of the site study treatment team, very limited replacement of study medications is permitted as clinically indicated.

11.8 Medication Packaging

All study medications shall be prepared and dispensed by a pharmacist or licensed medical practitioner appropriately trained and authorized to dispense study medications per local regulations.

XR-NTX is supplied in single use kits. Each kit will contain one 380 mg vial of Vivitrol® microspheres and one vial containing 4 mL (to deliver 3.4 mL) diluent for the suspension of Vivitrol®, one 5-mL prepackaged syringe, one 1-inch 20-gauge needle, two 1.5-inch 20-gauge needles and two 2-inch 20-gauge needles with needle protection devices. Lot number is included on the kit labels as supplied by the manufacturer.

12.0 STUDY ASSESSMENTS

Study assessments are intended to capture the outcomes of interest as efficiently as possible, minimizing the time and expense of research visits. Table 7 and 7a is a schedule of procedures and assessments.

12.1 General

12.1.1 Locator Form

A locator form is used to obtain information to assist in contacting participants during treatment and at follow-up. This form collects the participant's current address, email address, and phone numbers. In order to facilitate locating participants if direct contact efforts are unsuccessful, addresses and phone numbers of family/friends who may know how to reach the participant are collected, as well as information such as social security number, driver's license number and other information to aid in searches of public records. This information is collected at screening and is updated at least every month during the active treatment phase. No information from this form is used in data analyses.

12.1.2 Prisoner Status Assessment

The Prisoner Status Assessment will collect information on the participant's incarceration status. Candidates will complete a Prisoner Status Assessment to determine that they do not meet the OHRP definition of prisoner. If they do meet the definition, they will not be able to enter the study and considered a screen fail. They will be referred to clinical staff on site for their treatment needs. If the potential participant does not meet the definition of prisoner, they will be invited to learn more about the study and complete the informed consent process.

12.1.3 PhenX Tier 1 and Demographics

The Substance Abuse and Addiction Collection of the PhenX Toolkit (www.phenxtoolkit.org) includes measures that are being adopted across NIDA-funded research. The Core Tier 1 collection includes measures for demographics (age, ethnicity, gender, race, educational attainment, employment status, marital status), quality of life, and HIV risk and status; substance use measures include age of onset, past 30-day quantity and frequency, lifetime use for alcohol, tobacco and other substances. Substance use and other data that will be captured via other forms (e.g., Timeline Followback) will not be re-assessed via Core Tier 1 PhenX measures. However, PhenX assessments will be used to supplement this data in order to maintain compliance with NIDA recommendations, (e.g., Quality of Life (QLP) and Tobacco Use History (TUH)). Core Tier 1 assessments are completed at Baseline only.

12.1.4 Motivation Scale

A Likert scale asking about motivation to complete detoxification will be administered at baseline. This will be used to assess patient reported level of motivation as a mediator of successful treatment.

12.1.5 Patient-Reported Outcomes Measurement Information System (PROMIS)

Health-Related Quality of Life (HRQoL) is measured using the Patient-Reported Outcomes Measurement Information System (PROMIS) (Cella *et al*., 2010; HealthMeasures). PROMIS was developed using item response theory (IRT), with support from the NIH. Because of its IRT foundation, PROMIS is able to improve upon common deficiencies of existing, widely-used, HRQoL instruments capable of generating a single health utility index value for the calculation of Quality-Adjusted Life Year estimates (QALYs), such as the Euro-QoL, Health Utilities Index (HUI), SF-6D, and Quality of Well-being Index, including: floor and ceiling effects among participants who are especially ill or healthy, respectively, and imprecise questions that blend concepts. The PROMIS-Preference (PROPr) scoring system uses the respondent's scores for each of the following PROMIS domains to calculate a health utility index value that represents the general US population's preference for the respondent's current health state: Cognitive Function–Abilities, Depression, Anxiety, Fatigue, Pain Interference, Pain Intensity, Physical Function, Sleep Disturbance, and Ability to Participate in Social Roles and Activities (Hanmer *et al*., 2015). PROMIS has 5 levels for each domain: no problems, slight problems, moderate problems, severe problems, and extreme problems. The health-utility value produced by PROPr can range from -0.022 to 1, where 0 represents death, 1 represents perfect health, and values below 0 represent states perceived to be worse than death. Construct validity for PROPr has been demonstrated using the EQ-5D-5L, the HUI, and two large datasets from the general US population (Hanmer *et al*., 2018). The health-utility value is then used to calculate QALYs. PROMIS is completed at the time points specified in Table 7.

12.1.6 End of Medication Treatment Form

If a participant withdraws from XR-NTX treatment before Week 8, the reason for discontinuation of treatment should be documented on the EMT assessment form.

12.1.7 Study Completion Form

This form tracks the participant's status in the study. It is completed at the Week 8 visit, after the third XR-NTX injection is administered. This form is used in data analyses to address variables such as treatment retention and completion. This form also provides a location for the site PI attestation of review of all study data. At the conclusion of study treatment, participants are discharged to treatment in the community. This form documents the treatment plan identified for the participant at the timepoints specified in Table 7 if an individual leaves the study early.

12.1.8 Treatment Satisfaction Survey

Satisfaction with treatment is recorded on the Treatment Satisfaction Survey completed at the timepoints specified in Table 7.

12.1.8 COVID-19 Impact

This form was developed for the NIDA CTN and documents the effect that COVID-19 has had on the participant's life in the following domains: A) Personal exposure and illness related to COVID-19; B) Mental health symptoms and functioning and health care accessibility (Hefner *et al*., 2021).

12.2 Measures of Primary and Secondary Outcomes

12.2.1 End of Induction Form

This form tracks the participant's status with regards to the completed inpatient XR-NTX induction and receipt of the first XR-NTX dose (primary outcome measure). It will be completed by all enrolled patients in both arms of the trial at the end of the inpatient induction phase.

12.2.2 The Subjective Opioid Withdrawal Scale (SOWS)

The SOWS is a 16-item questionnaire designed to measure the severity of opioid withdrawal symptoms. The participant rates the intensity of symptoms using a 5-point scale; with 0 representing "not at all" and 4 representing "extremely". SOWS is administered at the time points specified in Table 7. Assessments captured during inpatient admission but prior to study enrollment will be retrospectively abstracted from the participant's chart.

12.2.3 Clinical Opiate Withdrawal Scale (COWS)

The COWS is a questionnaire designed to measure 11 common opioid withdrawal signs and symptoms (Wesson and Ling, 2003). The summed score provides information about the severity of opioid withdrawal and the level of physical dependence on opioids. The COWS will be administered by a clinician at the time points specified in Table 7. Assessments captured during inpatient admission but prior to study enrollment will be retrospectively abstracted from the participant's chart.

12.2.4 Visual Analogue Scales (VAS)

The craving for opioids will be measured using a horizontal VAS, that ranges from 0 (no craving) to 100 (most intense craving possible). Participants will be instructed to indicate the overall intensity of craving experienced. These scales are completed for opioid craving at the time points specified in Table 7.

A separate VAS will be used to document responses to opioid use, in the event that participants self-reports use of opioids during the study and will range from 0 (no opioid effect) to 100 (most intense opioid effect possible).

12.2.5 Timeline Followback (TLFB)

The Timeline Followback (Robinson *et al.*, 2014, Sobell and Sobell, 1992) procedure is used to elicit the participant's self-reported use of substances before and throughout the entire study period. The study TLFB also captures participant self-reported use of MOUD. At baseline, this form is used to assess substance use reported by the participant for 31 days, ranging from 30days prior to inpatient admission and including any use on admission day. After baseline, the TLFB is administered at every study visit to document the participant's self-reported use of substances and MOUD for each day since the previous TLFB assessment. The TLFB is administered at the time points specified in Table 7. Note that TLFB assessments during post-induction phase should capture any XR-NTX study injection given to participants who leave the unit and return to receive their first XR-NTX injection. If available in the EMR, research staff should capture any MOUD

received by participants that do not complete follow up assessments during the post-induction phase.

12.2.6 Urine Drug Screen (UDS)

Urine samples for drug toxicology testing are collected at the time points specified in Table 7. Urine toxicology tests administered during inpatient admission but prior to study enrollment will be retrospectively abstracted from the participant's chart. In most cases the UDS should be completed before assessing self-reported drug use or administering/dispensing medications. All urine specimens are collected using FDA-approved one-step temperature-controlled urine drug test cups following all of the manufacturer's recommended procedures. The UDS tests for the presence of the following drugs: opioids (morphine, oxycodone, methadone, buprenorphine, fentanyl), benzodiazepines, barbiturates, cocaine, amphetamine, methamphetamine, phencyclidine (PCP), ecstasy (MDMA), and marijuana. In the event urine specimen tampering is suspected, either based on the observation of specimen collection or identified by the adulterant tests, study staff will request a second urine sample and may observe the urine collection process according to clinic standard operating procedures. A further validity check will be performed using a commercially available adulterant test strip.

12.2.7 Patient Health Questionnaire (PHQ-9)

The PHQ-9 is a 9-item screening, diagnosing, monitoring, and measurement tool of depression severity with comparable diagnostic validity across multiple studies to longer screening tools (Kroenke *et al*., 2001). This assessment is completed at the time points specified in Table 7.

12.2.8 Low Dose Naltrexone Titration

The Low Dose Naltrexone Titration form records whether or not a low dose naltrexone titration occurred at each study day. If a titration did not occur for a visit, this form records the reason.

12.2.9 XR-NTX Administration

The XR-NTX Administration form is used to collect information about the participant's prior XR-NTX injection and the current XR-NTX injection performed at the follow-up study visit. Induction success participant's injections are documented on the EOI (1st injection) and the INN and M97 (2nd and 3rd injections). Induction failure injections are documented only on the M97 (all 3 injections).

12.3 Safety and Medical

The study physician must review and approve all safety and eligibility assessments in order to confirm participant eligibility prior to enrollment. However, the baseline medical and psychiatric evaluation (including history, physical and mental status exams) and laboratory tests are part of standard admission procedures at sites and may be conducted prior to study consent.

12.3.1 Medical and Psychiatric History

The site medical clinician will obtain a medical and psychiatric history from the participant covering past and present health conditions to help determine eligibility and to provide baseline information.

This will be collected during screening. This will include screening for suicidal thoughts and risk factors as well as severe psychiatric illness that are exclusionary for study participation.

12.3.2 PTSD Checklist for DSM-5 (PCL-5)

This is a 20-item self-report measure that assess symptoms of PTSD at baseline. The PCL-5 assesses trauma exposure (Criterion A), allows for a total symptom severity score (0-80), DSM-5 symptom cluster severity scores (Clusters B, C, D, and E), provisional PTSD diagnosis, and a preliminary diagnostic cut-point (i.e., 33). (Weathers et al., 2013b).

12.3.3 Adult ADHD Self-Report Screening Scale for DSM-5 (ASRS-5)

This 6-item screening scale (Ustun et al., 2017) is used to assess Attention Deficit-Hyperactivity Disorder based on DSM-5 criteria at baseline.

12.3.4 Overdose Questionnaire

An 8-item overdose questionnaire has been developed and used in research at McLean Hospital (unpublished questionnaire). The questionnaire examines participants' lifetime and recent history of opioid overdose, and the degree to which it is related to suicidal ideation vs. accident (responses indicating suicidal ideation trigger an evaluation and completion of the MHA). All overdoses that occur during an individual's participation in the study and meet SAE criteria will also be reported as an SAE.

12.3.5 Concomitant Medications

The Concomitant Medications form captures any medications that have been taken in the past week, other than those taken for treatment of opioid use disorder.

12.3.6 Death

The Death Form is used to report any participant deaths that occurred during the duration of the study.

12.3.7 HIV and Hepatitis Self-Report Assessments

At baseline, self-report forms will be collected regarding the participant's HIV, hepatitis C virus (HCV) and hepatitis B status. These forms will not be used to determine eligibility and will only be collected from participants who enroll into the study.

12.3.8 DSM-5 Criteria

The site medical clinician, or designee, conducting initial evaluation at screening will complete the DSM-5 checklist that includes all 11 DSM-5 criteria to determine a current diagnosis for all substances (opioids, cannabis, alcohol, stimulants, etc.) use categories, with opioid use disorder diagnosis being used to determine eligibility.

12.3.9 General Anxiety Disorder-7

The GAD-7 is a 7-item screen used to assess anxiety. It has been validated as a screening tool providing diagnostic and severity information in the general and substance use disorder

populations. This assessment is completed at the time points specified in Table 7.

12.3.10 PHQ-Panic Disorder

The PHQ-PD is a two-part screening battery including a single screening question with follow up questions. It has been found to have moderate sensitivity and good specificity for panic disorder in high risk populations. It will be administered at baseline.

12.3.11 Pregnancy, Birth Control, and Breastfeeding Assessment

Urine pregnancy test (for biologically female participants) will be performed to help determine eligibility at screening. Receipt and review of pregnancy test result is necessary before confirming eligibility, finalizing enrollment, and starting study medication for biological females of childbearing potential. Pregnancy tests administered during inpatient admission but prior to study enrollment will be retrospectively abstracted from the participant's chart.

This form documents that pregnancy tests were administered, pregnancy test results, and self-reported birth control and breastfeeding status. The pregnancy and birth control assessment, including on-site urine pregnancy tests, is conducted at screening and may be repeated prior to enrollment and/or induction. Birth control assessment and urine pregnancy tests are performed before each XR-NTX injection.

12.3.12 Mental Health Assessment

This assessment must be completed if the participant endorses suicidality on item 9 of the PHQ-9. The completion of the Mental Health Follow-Up Assessment form requires notification of the site clinician and evaluation of the participant for suicide/homicide risk according to the site's specific SOP. This evaluation will take place prior to the participant's leaving the study site.

12.3.13 Adverse Events (AEs) and Serious Adverse Events (SAEs)

At each medical management visit the study clinician assess for AEs and SAEs by asking the study participant, "How have you been feeling since your last visit?" AEs and SAEs may also be spontaneously reported to study staff at any visit following consent. A trained clinician will examine the XR-NTX injection site and participants will be asked to immediately report any injection site reactions to study staff for evaluation. Medication injection site reactions should be documented on the Medication Injection Site Abnormality Log and will not be duplicate-reported as an adverse event unless meeting the SAE definition.

AEs and SAEs suggesting medical or psychiatric deterioration will be brought to the attention of a study clinician for further evaluation and management. Medical management visits will emphasize overdose risk and risk-management; reported overdoses will be captured on the overdose questionnaire and if they meet SAE criteria will also be reported as SAEs on AE/SAE form sets. All volunteered, elicited, and observed SAEs will be recorded on the AE/SAE form set. AE and SAE reporting is according to the reporting definitions and procedures outlined in the protocol and in accordance with applicable regulatory requirements.

For the purpose of this study, AEs included in the following list of targeted safety events should be recorded on the Targeted Safety Events form and reported in the data system:

- Fall event (related to medical/psychiatric condition such as dizziness, confusion with head injury)
- Acute change in mental status (i.e. disorientation, amnesia, cerebrovascular accident, coma)
- Acute medical complication likely exacerbated by the stress of withdrawal (i.e. hypertensive crisis, hypotensive event with medical sequelae such as fall and/or requiring urgent fluid resuscitation, severe chest pain, acute respiratory decompensation, asthma attack, diabetic ketoacidosis, severe hypoglycemia, severe electrolyte abnormalities (hyper-/hyponatremia, hyper-/hypokalemia), precipitated withdrawal
- Acute psychiatric symptoms (i.e., psychosis, hypomania, severe agitation, violence)

Targeted safety events will also be captured on study specific forms (for example, COWS, SOWS, PHQ-9, Medication Injection Site Abnormality Log and Overdose Questionnaire).

Spontaneous reporting of withdrawal symptoms (including any withdrawal precipitated by the study medication) by the participant will be captured on scheduled specific structured questionnaires. Targeted safety events will not be duplicate-reported as an adverse event unless meeting the SAE definition.

12.4 Implementation Related Outcomes

12.4.1 Quantitative Data Collection and Analysis

12.4.1.1 Unit Organizational Readiness

The Organizational Readiness to Change Assessment (ORCA) (Helfrich *et al.*, 2009) instrument evaluates factors impacting implementation. The ORCA has been applied to the evaluation of interventions intended to promote evidence-based practices, including addiction treatment, and predicts implementation efforts. We will use a modified version of the ORCA with wording to reflect XR-NTX initiation readiness. This 5-minute survey asks the respondent to rate local factors related to beliefs regarding the evidence behind the intervention, internal and external organizational level factors as well as facilitation process related factors on a 5-point Likert scale from strongly disagree to strongly agree. Facilitation questions will be omitted from the baseline assessment since this part of the intervention will not have taken place.

12.4.1.2 Medical Provider Readiness and Preparedness

We will use change rulers, among appropriate providers, to assess readiness and preparedness on XR-NTX initiation (LaBrie *et al.*, 2005). Stage of change assessments have been validated and have been used in the field of addiction and mental health to assess readiness to adopt evidence-based treatments (Aarons, 2004, Amodei and Lamb, 2004, Haug *et al.*, 2008).

The change rulers will independently assess, on a 0-10 scale each provider's:

1. Readiness to provide the intervention
2. Preparedness to provide the intervention

The readiness rulers will be completed at the beginning of the study and before and after each stepped-wedge intervention.

12.5 Qualitative Data Collection and Analysis

At each of the six study sites, we will conduct interviews with a purposeful sample of key stakeholders at distinct stages of our project: at each site after the brief XR-NTX implementation intervention and at the study conclusion. However, this information is part of an iterative process, and therefore additional structured interviews, one-on-one phone interviews and email correspondence will take place as needed. Purposeful sampling is a well-established method in qualitative studies and is designed to identify study participants who have direct experience with or knowledge of the phenomenon of interest, in this case OUDs and XR-NTX initiation (Palinkas *et al.*, 2015). We will interview a variety of participants including detox/rehab patients, nurses, social workers, physicians, NPs, PAs, pharmacists and nursing directors at each detoxification/ residential rehab site and office-based XR-NTX providers and representatives from outpatient treatment programs to allow for evaluation of processes from multiple perspectives (triangulation). Interviews will be conducted with study participants chosen at random and representation from each of the stakeholder categories. The interview guides will be informed by the CFIR framework and include questions designed to establish rapport and elicit open-ended responses. For example, participants will be asked open ended questions regarding their attitudes towards the overall experience of implementing XR-NTX. Probes will be used to understand specific details of those experiences and allow for clarification of ideas. We will design and pilot test these guides with study participants and key informants from the participating NIDA CTN nodes and refine as indicated.

12.5.1 Verbal Consent for Structured Participant Interviews

Key stakeholders comprised of detoxification/residential rehab staff and participants undergoing induction procedures will each be provided with an IRB-approved verbal consent including all significant elements of the study. We will work with the detox and rehab units to provide staff assurances that their participation in the research will in no way affect their employment status either positively or negatively. Interviews will be voluntary, and information collected for research purposes will not become part of staff participants' personnel records. The verbal consent for the detoxification/residential rehab staff participants will outline these assurances. For the recording of the interviews, all participants will be made aware during the verbal consent process that the sessions will be digitally recorded and, for induction participants, that the nature of these sessions will involve participants speaking about information regarding their health status and opioid use. All structured interview participants will also be made aware that they will complete a brief demographic survey.

12.5.2 Conduct of Structured Interviews

Trained and qualified personnel will conduct structured interviews with each detoxification/ residential rehab site. The structured interviews will be recorded by the lead team and professionally transcribed for qualitative analysis. Participants will also complete a brief demographic survey. Using directed content analysis, we will analyze the data with a multi-disciplinary group with experience in qualitative methods, which will include Addiction Medicine/Psychiatry physicians. Consistent with prior studies, we will plan to generate a summary of the findings to share with participants via email and conference calls for their feedback. We will use data reduction strategies to sharpen, sort, focus, discard and organize data in order to draw inferences regarding the implementation interventions in place at each detoxification/residential rehab site. These data will directly inform the implementation facilitation package and be used to evaluate the facilitation process and outcomes. This will be an iterative process. We will develop a template summary of data at each detoxification/residential rehab organized by evidence, context and facilitation-related factors. At the completion of all detoxification/residential rehab sites implementation periods, we will create a matrix across all sites and respondents to understand the major issues regarding implementation across sites that can be disseminated to the wider addiction treatment community.

12.6 Implementation Process Outcomes Measures

12.6.1 Fidelity to Clinical Procedures

12.6.1.1 Critical Action Checklist and Medical Procedures Data Capture Form

Study personnel will assess fidelity to clinical procedures and guidelines (adherence) for each participant using a critical action checklist throughout the detoxification and rehab stay. The checklist will include confirmation of: urine toxicology obtained, urine pregnancy test completed for females; patient-participant meets criteria for DSM-5 moderate-severe opioid use disorder; urine positive for opioid; daily formal assessment of Clinical Opioid Withdrawal Scale (COWS); buprenorphine and comfort medication orders written and administered according to the recommended study procedures, including standing and as needed additional administrations; detox/rehab-initiated XR-NTX provided; XR-NTX education and induction instructions provided; education regarding XR-NTX and continued treatment is provided; comfort medication prescriptions provided upon discharge for potential protracted opioid withdrawal symptoms and appropriate tapered dosing plans for clonidine and benzodiazepines; and an appointment for ongoing XR-NTX is provided.

In addition to the critical action checklist, all medical assessments (vital signs, COWS, lab tests etc.) and administered medications will be captured using a medical procedures data capture form. In cases where programs are showing poor fidelity to study related procedures, this will be raised with the local team during coaching calls to explore barriers and create an action plan to improve fidelity going forward.

12.6.1.2 Organization Level Clinical Implementation Data Collection Form

In addition, the following organization level implementation related clinical and clinical process outcomes will be captured: The proportion of eligible patients screened for XR-NTX, proportion of patients who begin XR-NTX on the detox or residential units, proportion of those who are successfully linked to office-based XR-NTX providers and/or OTPs. EHRs will be queried for each month to determine potential ICD (International Classification of Diseases) 10 codes identifying patients that were potentially eligible for XR-NTX.

12.6.2 Coaching Progress Notes

Clinical staff and other key stakeholders from each site will participate in routine meetings to discuss study and clinical issues that arise during the RP implementation phase. Individual site-level coaching sessions will occur once weekly at a minimum during the initial RP implementation phase with flexibility for additional support sessions as per each site's needs (total number of coaching sessions delivered to each site will be captured). After individual site-level coaching support sessions, all sites randomized to RP will participate in collaborative coaching sessions beginning weekly and the frequency moving forward will be determined by the sites' needs. A structured progress note will be developed (informed by the CFIR framework) and completed by the study team that will include barriers/facilitators to implementing the RP intervention, pertinent action items, and any modifications or adaptations to implementation strategies or clinical processes. This information will be integrated with other implementation data to guide iterative improvements in the implementation facilitation package.

12.6.3 Site Needs Assessment and Site Mapping Progress Notes

Study personnel at each site will complete an implementation needs assessment during the pre-implementation phase that will provide preliminary information to the study team about organizational processes, clinical procedures and workflow, and other factors potentially influencing the uptake and success of implementing the Rapid Procedure. The study team will meet with the site to review the Site Needs Assessment and gather additional details through a process of Site Mapping. Weekly meetings will be held in the pre-implementation phase to review a structured Site Mapping guide that facilitates discussions around inner structure and organizational processes related to the implementation of the Rapid Procedure. A structured progress note will be used to capture meeting minutes, identified barriers and facilitators, and potential modifications or adaptations needed on-site and to implementation strategies in order to facilitate future implementation of the intervention.

12.6.4 Internal Implementation Team Meeting Notes

A subset of the research team with expertise in implementation will meet routinely to review the implementation facilitation process, identified barriers and facilitators, and evaluate if modifications or adaptations to the implementation strategies are needed in order for the site to successfully implement the Rapid Procedure. A structured note will be used to capture meeting minutes, pertinent barriers and facilitators, and final decisions on any modifications or adaptations to the implementation strategies.

12.6.5 Fidelity to Implementation Procedures Checklist

Study personnel at each site will track implementation activities using an implementation activity checklist. This will include fidelity to suggested training programs and other implementation procedures. The checklist will be completed after the initial training activities at the start of the study, after each wedge implementation and each month to capture follow up implementation support (weekly clinical case conferences etc.). To capture engagement with implementation processes, the number of providers attending the initial educational session and follow up coaching session will be tracked.

12.6.6 Treatment Utilization Measures

12.6.6.1 Medical Management Log

A Medical Management attendance log will be completed at the end of the induction phase and at every post-induction visit to document attendance or non- attendance at each Medical Management session during the active treatment phase.

12.6.6.2 Psychosocial and Medical Treatment Participation Log

At each visit, participants will complete a psychosocial and medical treatment utilization log (“psychosocial log”) to report on their participation in any medical and/or psychosocial treatment since the last visit.

12.7 Other Assessments

12.7.1 Fagerström Test for Nicotine Dependence (FTND)

The Fagerström Test for Nicotine Dependence (FTND) is used for assessing nicotine dependence and is administered at the time points specified in Table 7 (Fagerstrom, 1978).

12.7.2 NIDA Cannabis Assessment

The NIDA cannabis assessment is a recently developed measure of medical and recreational cannabis use, and perceived benefit and/or harm associated with its use over the previous 12 months. It will be assessed at the time points specified in Table 7.

12.7.3 Daily Medication Administration Log

Data on detoxification, including number of days on the unit and medications received since admission, will be collected. The Daily Medication Administration Log will be completed as described in Table 7.

12.7.4 Non-Study Medical and Other Services (NMS)

Medical services that are not part of the treatment intervention will be recorded on the NMS form. The NMS form captures services received outside the study and CTP, including therapy visits, physician visits, subsequent residential or hospital detoxification, hospital visits and emergency room visits, medication use, and participation in mutual-support group meetings through participant self-report. The assessment also captures health insurance status, housing status, employment, criminal activities, and contact with the criminal justice system. Homelessness is of

particular interest and concern with this population. Thus, a question to determine whether the participant is currently homeless or living in a shelter is asked at baseline in conjunction with the PhenX Quality of Life assessment and during each monthly visit where follow-up XR-NTX injections are administered. The NMS will be completed as described in Table 7.

12.7.5 Oral Fluid Collection for Genetic Sample and Family Origin

NIDA CCTN has requested that oral fluid samples for genetic analyses be collected for all pharmacotherapy trials. Enrolled participants who provide consent for genetic analyses will have an oral fluid sample collected for genetic analyses and will complete a Family Origin form. The family origin form is designed to be interviewer administered. It collects information about the participant and her/his biological family members' race/ethnicity, place of birth, and ancestry. If a participant does not know the information requested, the participant may answer "unknown." This sample will ideally be collected during the baseline visit. If this is not possible, this sample can be collected at any other future visit. The oral fluid samples will be coded, thus only the local investigators will know the true identity of the participant providing the sample. This sample will be sent to a common repository.

12.7.6 Table 7: Study Assessments

	Consent	Screening	Baseline	Inpatient Induction Phase		Post-Induction Phase						End of Medication Treatment
**Visit #**				**Rapid Procedure**	**Standard Procedure**	**1**	**2**	**3**	**4**	**5**	**6**	**EMT**
**Week**						**1**	**2**	**3**	**4**	**6**	**8**	
**Time**				**IND-RP**	**IND-SP**	**P6**	**P13**	**P20**	**P27**	**P41**	**P55**	**Varies**
Discussion of XR-NTX for relapse prevention to opioid dependence	X											
Informed Consent	X											
Medical Release Form	X											
**GENERAL**												
Inclusion/Exclusion Checklist		X										
Locator form		X				X	X	X	X	X		X
Prisoner Status Assessment		X						X				
Demographics	X		X									
PhenX Tier 1 Assessments [1]			X									
Fagerstrom			X									
Cannabis Use Assessment			X									
Motivation Scale			X									
End of Induction Form				X	X							
End of Medication Treatment Form												X
Treatment Satisfaction Survey											X	
Study Completion Form				^	^	^	^	^	^	^	X	
COVID Impact Assessment			X									
**SAFETY & MEDICAL**												
Medical & Psychiatric History		X										
DSM-5 criteria		X										
ASRS-5			X									
PHQ-Panic Disorder			X									
PCL-5			X									

	Consent	Screening	Baseline	Inpatient Induction Phase		Post-Induction Phase						End of Medication Treatment
**Visit #**				**Rapid Procedure**	**Standard Procedure**	**1**	**2**	**3**	**4**	**5**	**6**	**EMT**
**Week**						**1**	**2**	**3**	**4**	**6**	**8**	
**Time**				**IND-RP**	**IND-SP**	**P6**	**P13**	**P20**	**P27**	**P41**	**P55**	**Varies**
General Anxiety Disorder-7			X	X[2]	X[2]	X	X	X	X	X	X	
HIV and Hepatitis Assessments			X									
Urine Pregnancy and Birth Control Assessment		X		X[3]	X[3]				X[3]		X[3]	
Daily Medication Administration Log			X	X	X							
Concomitant Medications		X				X	X	X	X	X	X	X
AEs & SAEs	X	X	X	X	X	X	X	X	X	X	X	X
Opioid Overdose Questionnaire			X			X	X	X	X	X	X	
Medication Injection Site Abnormality Log				^	^	^	^	^	^	^	^	^
Death Form		^	^	^	^	^	^	^	^	^	^	^
Saliva Sample (Genetic Sample)			X									
Family Origin Assessment			X									
**TREATMENT SCHEDULE**												
Induction Buprenorphine				X	X							
Naltrexone Titration				X								
XR-NTX Administration				X	X				X		X	

	Consent	Screening	Baseline	Inpatient Induction Phase		Post-Induction Phase						End of Medication Treatment
**Visit #**				**Rapid Procedure**	**Standard Procedure**	**1**	**2**	**3**	**4**	**5**	**6**	**EMT**
**Week**						**1**	**2**	**3**	**4**	**6**	**8**	
**Time**				**IND-RP**	**IND-SP**	**P6**	**P13**	**P20**	**P27**	**P41**	**P55**	**Varies**
**ADHERENCE**												
Medical Management Log				X	X	X	X	X	X	X	X	X
Psychosocial Log				X	X	X	X	X	X	X	X	
**OUTCOME**												
Timeline Followback – Substance Use			X			X	X	X	X	X	X	
Timeline Followback – MOUD			X			X	X	X	X	X	X	
Urine Drug Screen		X		X^3	X^3				X^3		X^3	
PROMIS			X						X		X	
VAS Opioid Craving			X	X^4	X^4	X	X	X	X	X	X	
VAS Opioid Effect						^	^	^	^	^	^	
Patient Health Questionnaire-9			X	X^2	X^2	X	X	X	X	X	X	
Mental Health Assessment			^			^	^	^	^	^	^	
Subjective Opioid Withdrawal Scale			X	X^4	X^4	X	X	X	X	X	X	
Clinical Opioid Withdrawal Scale			X	X^5	X^5							
Non-Medical and Other Services			X						X		X	

X^1 = Includes ASU, TUH, QLP assessments
X^2 = repeat weekly during inpatient induction phase
X^3 = before each XR-NTX injection
X^4= repeat each inpatient study day
X^5= repeat daily, as many times as clinically appropriate
^ = As Needed

12.7.7 Table 7a: Implementation Assessments

	Pre-Implementation Training (8 weeks prior to Step Start)	Beginning of Site Implementation (Step Start)	Weekly during Site Implementation	Monthly during Site Implementation	Post Site Implementation (End of Step)	Study Conclusion
**Critical Action Checklist**			X[1,2]			
**Medical Procedures Form**			X[1,2]			
**Coaching Progress Notes**			X[3]			
**Staff Structured Interviews**					X	X
**Site Needs Assessment**	X					
**Site Mapping Progress Notes**	X					
**Internal Implementation Team Notes**	X		X[3]		X	X
**ORCA**	X				X	
**Readiness and Preparedness Rulers**	X				X	
**Organization Level Clinical Implementation Data Collection Form**		X				
**Fidelity to Implementation Procedures Checklist**		X			X	

1 = Patient level assessment
2 = During inpatient induction phase only
3 = May occur more frequently depending on site needs

13.0 GENETIC SAMPLING

The NIDA CCTN has requested that blood or saliva samples for genetic analysis be obtained for all new CTN pharmacotherapy trials. All participants will be asked to provide a saliva sample for genetic testing. As part of the study ICF, participants will be asked to consent to providing a genetics sample. This will consist of having a single saliva cheek swab. Saliva will be collected during the baseline visit but may occur thereafter. The saliva sample will be sent to Infinity BiologiX, LLC (previously called the Rutgers University Cell and DNA Repository) for storage and future analysis. The saliva samples will be coded and only the local investigators will know the identity of the participant providing the sample.

The Family Origin CRF will be collected from participants at baseline.

14.0 TRAINING REQUIREMENTS

14.1 Overall

A Training Plan will be developed that will be independent from the implementation facilitation procedures for clinical team. The Plan will incorporate study-specific training for research procedures and assessments, mechanisms for competency assessment as well as a detailed description of training, supervision, and fidelity monitoring procedures. The Investigative Team is responsible for the development of a comprehensive Training Plan, instructional materials, and delivery of the training, with the team comprised of the Lead Node, CCC, DSC, as well as other participating nodes and subject matter experts, as applicable.

The CTN-0097 study staff will be trained as specified in the study Training Plan. Training will include Human Subjects Protection (HSP) and Good Clinical Practice (GCP) as well as protocol-specific training on assessments, medication management, study interventions, safety and safety event reporting, study visits and procedures, data management, quality assurance, laboratory procedures, etc. The Lead Node is primarily responsible for development and delivery of study-specific training related to the study intervention(s) and procedures. The CCC is responsible for the development and delivery of non-intervention training, including regulatory and laboratory procedures, safety and safety event reporting, quality assurance and monitoring, etc. The DSC is responsible for training related to data management (DM), the electronic data capture system, and good DM practices. Other parties will contribute as needed based on the subject matter and material to be covered. The various sub-teams will collaborate to deliver quality instructional material designed to prepare research staff to fully perform study procedures based on the assigned research roles and responsibilities.

In addition to general and study-specific training, the Training Plan will include a description of the delivery methods to be used for each training module (e.g., via self-study, online, webcast, or teleconference). Study staff is required to complete institutionally required training per their research site, Institutional Review Board(s), and authorities with regulatory oversight. Tracking of training completion for individual staff as prescribed for assigned study role(s) will be documented, endorsed by the site Principal Investigator and the Lead Node, and audited by the CCC. As changes occur in the prescribed training, the Training Plan and training documentation tracking forms will be amended to reflect these adjustments.

15.0 CONCOMITANT THERAPY/INTERVENTION

15.1 General

Prescribed psychoactive medications, including anxiolytics and antidepressants, will be allowed for the duration of the study per the judgement of the local clinical team. At the screening visit, participants will be asked about the medications they taken one week prior to inpatient admission, including prescription and nonprescription medications, vitamins, and supplements. At subsequent post-induction visits, participants will be asked about medication use over the last week.

15.2 Medications Prohibited/Allowed During Trial

Opioid agonist that are not prescribed as part of the trial (e.g., buprenorphine) will not be allowed for the duration of the trial. Though, participants will not be withdrawn if they use illicit opioids including non-prescribed buprenorphine or methadone.

16.0 STATISTICAL DESIGN AND ANALYSES

16.1 General Design

16.1.1 Study Hypothesis

We hypothesize that the Rapid method will be non-inferior to the Standard method in terms of proportion of participants that receive the first XR-NTX injection. The primary goal of this hybrid effectiveness-implementation study is to determine whether the Rapid Procedure of initiating treatment with XR-NTX is non-inferior to a Standard Procedure on the primary effectiveness outcome of successful initiation of XR-NTX (receiving the first injection) when implemented at community- based inpatient or residential programs.

16.1.2 Primary and Secondary Outcomes (Endpoints)

Primary Endpoint: The primary outcome measure is a dichotomous indicator of whether each participant receives the first XR-NTX injection while inpatient or not at the end of the induction period, which is approximately Day 6 in the Rapid Induction Procedure and Day 13 in the Standard Induction Procedure.

Secondary Endpoints: Secondary endpoints include time to first injection or time to failure to receive first injection, receiving 2nd and 3rd monthly injections of naltrexone, opioid withdrawal symptoms, selected opioid and other drug use, adherence to any type of MOUD and adverse events during the induction period and in the month after XR-NTX initiation as discussed in Section 7.2. A set of additional implementation outcome measures, including acceptability and barriers, are detailed in Section 7.3. A preliminary economic analysis that compares cost of both interventions will also be included.

16.1.3 Study Design

The therapeutic strategies defined above are evaluated and compared in a multi-center, optimized stepped-wedge cluster randomized trial.

16.1.4 Recruitment

Patients with OUD planning admission or admitted to inpatient/short-term residential treatment sites will participate in a shared decision-making process regarding choice of maintenance medication (methadone, buprenorphine, XR-NTX). All those choosing XR-NTX and meeting clinical criteria for appropriateness for XR-NTX will be offered participation in the study and asked to provide informed consent. Further recruitment will vary based on individual site level factors (see Section 8.4 for further recruitment details). Note that the step length corresponding to the stepped-wedge design will remain fixed and will not be extended if recruitment rates are found to be slower than anticipated.

16.1.5 Randomization and Factors for Stratification

For this stepped-wedge cluster randomized trial, the randomization will be performed at site level. A total of six sites will be enrolled, five sites to initiate treatment using the Standard Procedure

first, and one site to initiate the Rapid Procedure (randomly chosen). The Rapid regimen will be introduced 1 site at a time, with one site chosen randomly every 14 weeks. The 6th site (the site remaining after the other 5 sites have been chosen to transition to RP) will only offer SP for the entire duration of the study).

16.2 Rationale for Sample Size and Statistical Power

16.2.1 Introduction

CTN-0097 SWIFT is a six-center, optimized stepped-wedge cluster randomized trial comparing effectiveness and safety of Rapid (5-7-day) versus the Standard (14-day) XR-NTX induction procedure (SP vs. RP). In this optimized stepped-wedge design (Thompson *et al.*, 2017), one randomly selected site will initiate treatment using a RP regimen, while 5 sites will initiate treatment using SP regimen first. After 14 weeks, one of the 5 sites will be randomly selected to stop offering SP and will begin offering a RP to participants interested in the study. After another 14 weeks, another randomly selected site will switch from SP to RP and 14 weeks later another randomly selected site will switch from SP to RP, and finally after another 14 weeks another randomly selected site will switch from SP to RP. The remaining (6th) site will continue offering SP for the reminder of the study and will never switch to RP. This design is represented schematically as the Design Pattern matrix of Table 8. This optimized stepped-wedge design provides more power to detect a treatment effect than a standard stepped-wedge design (Thompson *et al.*, 2017).

16.2.2 Table 8: Design Pattern Matrix

Table 8: Design pattern matrix giving schematic representation of the optimized stepped- wedge design compared with a standard stepped-wedge design feasible for CTN0097

Rows are sites, columns are blocks of time (steps), and cells contain 0 for Standard Induction Procedure or 1 for Rapid Induction Procedure.

Optimized Stepped-Wedge Design

		Time Block			
		1	2	3	4
Site	1	1	1	1	1
	2	0	1	1	1
	3	0	0	1	1
	4	0	0	0	1
	5	0	0	0	0
	6	0	0	0	0

Standard Stepped-Wedge Design

		Time Block			
		1	2	3	4
Site	1	0	1	1	1
	2	0	1	1	1
	3	0	0	1	1
	4	0	0	1	1
	5	0	0	0	1
	6	0	0	0	1

The primary outcome measure is the proportion of patients who receive the first XR-NTX injection at the end of the induction period, which is approximately Day 6 in the Rapid Induction Procedure

and Day 13 in the in the Standard Induction Procedure (binary: did or did not receive first injection of XR-NTX). It is hypothesized that the Rapid Induction Procedure will be non-inferior to the Standard Induction Procedure.

16.2.3 Values of Parameters Underlying Power Simulations

Probability of Success in the SP arm: We base the assumed success probability in the SP initiation regimen on the mean success rate in the NTX arm of CTN-0051, which is 55% for sites 02011, 02017, and 02052 pooled together. These are sites that are similar to the types of sites that would be eligible for this study. These sites were chosen because they used the standard induction method (agonist taper followed by the wait). Other sites, with much higher success rates, (75%-95%) were excluded because these were already using a version of the Rapid method (no agonist taper, using only alpha-2 agonist and other non-opioid medications) or were highly experienced with XR-NTX induction.

Probability of Success in the RP arm: We assume a difference in the proportion of successes of 15% based on the results of our prior controlled study, which compared Standard and Rapid XR-NTX procedures and found a 23.4% difference (56.1% vs 32.7%) (Sullivan *et al.*, 2017). We are assuming a smaller true treatment difference (15%) between SP and RP arm, which we believe to be more realistic in the inpatient/short-term residential setting. As the proposed trial will be conducted on the inpatient unit, reducing the risk of illicit opioid use, and because sites will use regimen-driven, aggressive dosing of adjunctive medications, we believe that overall success will be 55% in the Standard Procedure arm (as in study CTN051) with the 15% difference for the Rapid Procedure arm leading to an assumed probability of success of 70% in the RP arm.

Margin of non-inferiority: To show non-inferiority of RP to SP, we assume a 10% margin of non-inferiority, which corresponds to an odds ratio of 0.67 (proportion of success in RP = 0.45, proportion of successes in SP = 0.55). To show RP is non-inferior to SP, we need to show that the lower bound of the two-sided 95% CI for the odds ratio for RP and SP is higher than 0.67.

Under the above assumptions, the null and alternative hypotheses can be stated as follows: In terms of proportions (p):

Null (inferiority of RP to SP): p(RP) - p(SP) <=-10%

Alternative (non-inferiority of RP to SP under which power was calculated): p(RP) - p(SP) >15%

In terms of odds ratios (OR):

Null (inferiority of RP to SP): OR [odds(RP)/odds(SP)] <= 0.67

Alternative (non-inferiority of RP to SP under which power was calculated): OR [odds(RP)/odds(SP)] > 1.91

Intraclass Correlation Coefficient: We base the ICC estimate on a logistic regression of induction success/failure in CTN-0051 on treatment arm, with a random site effect whose variance is allowed to depend on arm. The adjusted analysis model for this study may have extra covariates (chosen

before data analysis), but we do not include them in the power analysis. We assume that extra covariates will improve analysis accuracy, implying that our current power estimates may be conservative. The estimated site standard deviation in the NTX arm from the regression model using data from CTN-0051 is 0.86, leading to an estimated ICC in the Standard induction arm of 0.14 under the generalized mixed model. As mentioned above, this ICC may be thought of as the induction status correlation between two individuals chosen randomly without replacement from the NTX arm of CTN-0051, given that they come from the same site.

In summary, the assumptions used for the power calculations for this non-inferiority optimized stepped-wedge cluster randomized trial are as follows:

- The projected number of clusters is 6 (sites), that will enroll participants across 5 periods of time, each period 14 weeks long
- The projected total number of participants enrolled is 450, with 15 participants enrolled per cluster per time period (equal allocation to sites per time period)
- The outcome of interest is a binomial outcome (the patient received or did not receive the first XR-NTX injection)
- The probability of success in the Standard induction arm is 0.55
- The probability of success in the Rapid induction arm is 0.70 (i.e. an effect size of 0.15)
- The margin of non-inferiority is 10%, which corresponds to an odds ratio of 0.67 (based on proportion of success in RP = 0.45 and proportion of successes in SP = 0.55). Refer to Figure 5 for the relationship between non-inferiority margin as a difference in proportions versus non-inferiority margin as an odds ratio.
- Observations are equally correlated within cluster, regardless of time or induction method with a site standard deviation of 0.86. The corresponding ICC in the Standard induction arm based on a logistic regression with random effects is 0.14.

In our power calculations, we vary the above assumptions to evaluate the sensitivity of the power calculations to different parameter values such as different ICCs, effect sizes, or number of sites.

For the power calculations, correlated binary data were simulated using the Parzen algorithm [*Parzen, M., 2009*] and power analyses were performed using the logistic model below.

Logistic model used for power calculations:

$$llllllllll(pp_{iiiiii}) = JJ_{iiii} * \delta\delta + \theta\theta_{ii} + \alpha\alpha_{ii}$$

$$\alpha\alpha_{ii} \sim NN(0, \tau\tau^2)$$

where:

- j indexes the site
- s indexes the 4-month block of time
- i indexes the individual within site j at time s.
- $\theta\theta_{ii}$ is the fixed effect of time

- $\alpha\alpha_{ii}$ is the random site effect
- pp_{iiiiii} is the probability of success for individual i within site j at time s.
- JJ_{iiii} is the treatment indicator for site j at time s
- $\delta\delta$ is the treatment effect

For all simulations, we used 10,000 iterations.

A SAS snippet capable of estimating the above model follows: proc glimmix data = simul

method = quad;
class site trt time;
model success = trt time / dist = binary link = logit solution oddsratio;

random intercept / subject = site group = trt;
run;

16.2.4 Power Curve

Two-tailed power to show non-inferiority of RP to SP at alpha level 0.05 is shown in Figure 2 as a function of ICC. For the sake of clarity, the vertical power scale runs from 0.6 to 1 instead of the conventional 0 to 1. Horizontal and vertical reference lines denote power = 80% and ICC = 0.14, respectively. Figure 2 depicts two power curves (explained below), but the uppermost curve (black line) shows power for the optimized stepped-wedge design. For the optimized stepped-wedge design, the expected power exceeds 88% for all possible values of the ICC. As the ICC increases, power first declines, then increases. This reflects that the source of power in a stepped-wedge design shifts from across-site comparisons to within-site comparisons as ICC increases. Note that the power for a similar standard stepped-wedge design was 3% to 8% lower compared to the optimized stepped-wedge design, across different ICC values.

16.2.4.1 Figure 2: Two Power Curves as Functions of ICC

Figure 2: Two power curves as functions of ICC corresponding to the optimized and standard stepped-wedge (SW) design to show non-inferiority of RP to SP, assuming a 15% treatment effect (d = 0.15). The non-inferiority margin is 10%, operationalized here as an odds ratio of 0.67. In other words, we reject the null of inferiority of RP to SP if the lower 95% confidence limit of the treatment success odds ratio [odds(RP)/odds(SP)] exceeds 0.67.

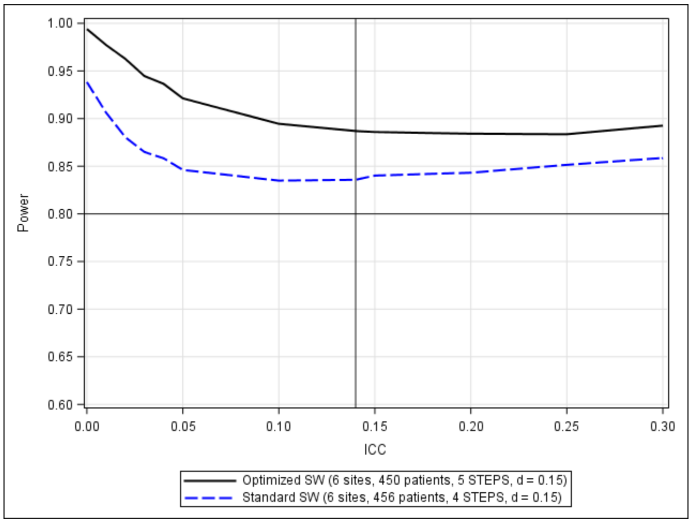

16.2.4.2 Table 9: Power for ICC to show non-inferiority of RP to SP

Table 9: *Power for ICC = 0.14 corresponding to the optimized and standard stepped- wedge (SW) design to show non-inferiority of RP to SP, assuming a 15% treatment effect (d = 0.15) and a margin of 10%*

Non-inferiority SW Design	Sites	Steps	SP Success	RP Success	Effect Size	ICC	Power	Participants per Site per Time Period	Total Sample Size
Optimized	6	5	0.55	0.70	0.15	0.14	0.887	15	**450**
Standard	6	4	0.55	0.70	0.15	0.14	0.836	19	456
SP = Standard Procedure; RP = Rapid Procedure; SW – Stepped – wedge; ICC – intra class correlation coefficient.									

16.2.5 Power in Unforeseen Circumstances

As remarked above, for the parameters chosen, we expect power to be at least 88%, irrespective of the true ICC. However, there might be unforeseen circumstances, such as sites failing to enroll or rates of enrollment to be lower than anticipated. Figure 3 explores the impact upon power if a site drops or if enrollment is 10% lower than expected. Figure 3 suggests that for this study design, losing site 3 costs more than losing site 1 or 5, or having a 10% less enrollment, in terms of power loss. However, there is still reasonable power (79% or more) to show non-inferiority of RP to SP regardless of which site drops or if enrollment is 10% lower, and irrespective of the true ICC. For ICC = 0.14, the power to show RP non-inferior to SP is 80% or more regardless of which site drops or if the enrollment is 10% lower.

In addition, we explored loss of power when the true treatment effect is smaller than anticipated. Figure 4 suggests that even for a smaller treatment effect of 13% we still have 80% power or more to show RP is non-inferior to SP, irrespective of the ICC values; for a treatment effect of 12% we have 78% power or more, irrespective of the ICC values; however, for a treatment effect

smaller than 11% we will not have enough power to show non-inferiority of RP to SP, for larger values of ICC. If the treatment effect is 11% or 10%, the power drops to 75% and 71%, respectively, for ICC=0.14 (or even lower for higher ICC values).

16.2.5.1 Figure 3: Power curves as functions of ICC when a site drops or when enrollment rates are lower than anticipated

Figure 3: Power curves as functions of ICC corresponding to the optimized stepped-wedge (SW) design to show non-inferiority of RP to SP, assuming a 15% treatment effect (d = 0.15) and a 10% margin when a site drops or when enrollment rates are lower than anticipated.

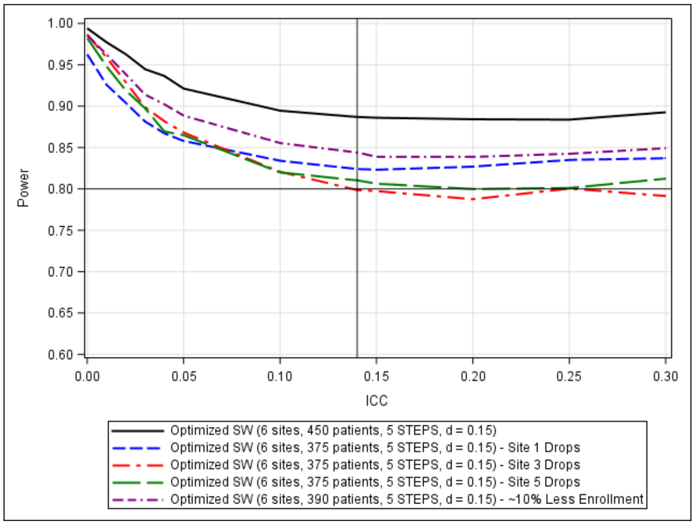

16.2.5.2 Table 10: Power for ICC when a site drops or when enrollment rates are lower than anticipated

Table 10: Power for ICC = 0.14 for the optimized stepped-wedge (SW) design to show non-inferiority of RP to SP, assuming a 15% treatment effect (d = 0.15) and a 10% margin when a site drops or when enrollment rates are lower than anticipated.

Non-inferiority Optimized SW Design	Sites	Steps	Participants per Site per Step	Total Sample Size	ICC	SP Success	RP Success	Effect Size	Power
No Drop-out	6	5	15	450	0.14	0.55	0.7	0.15	0.887
Site 1 Drops	5	5	15	375	0.14	0.55	0.7	0.15	0.824
Site 3 Drops	5	5	15	375	0.14	0.55	0.7	0.15	0.799
Site 5 Drops	5	5	15	375	0.14	0.55	0.7	0.15	0.810
~10% Less Enrollment	6	5	13	390	0.14	0.55	0.7	0.15	0.844

SP = Standard Procedure; RP = Rapid Procedure; SW – Stepped – wedge; ICC – intra class correlation coefficient.

16.2.5.3 Figure 4: Power curves as functions of ICC to show non-inferiority of RP to SP with a 10% margin, assuming different treatment effects

Figure 4: Power curves as functions of ICC corresponding to the optimized stepped-wedge (SW) design to show non-inferiority of RP to SP with a 10% margin, assuming different treatment effects between 15% and 10%

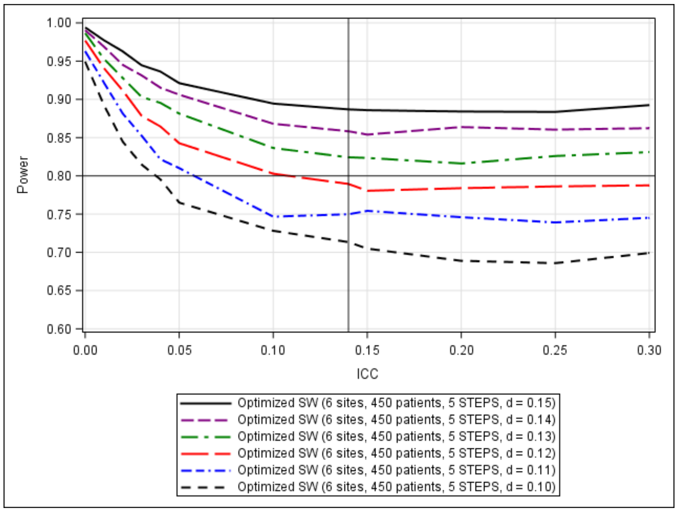

16.2.5.4 Table 11: Power for ICC to show non-inferiority of RP to SP with a 10% margin

***Table 11**: Power for ICC = 0.14 corresponding to the optimized stepped-wedge (SW) design to show non-inferiority of RP to SP with a 10% margin, assuming different treatment effects between 15% and 10%*

Non-inferiority Optimized SW Design	Sites	Steps	Participants per Site per Step	Total Sample Size	ICC	SP Success	RP Success	Effect Size	Power
Optimized SW	6	5	15	450	0.14	0.55	0.70	0.15	0.887
Optimized SW	6	5	15	450	0.14	0.55	0.69	0.14	0.858
Optimized SW	6	5	15	450	0.14	0.55	0.68	0.13	0.824
Optimized SW	6	5	15	450	0.14	0.55	0.67	0.12	0.789
Optimized SW	6	5	15	450	0.14	0.55	0.66	0.11	0.750
Optimized SW	6	5	15	450	0.14	0.55	0.65	0.10	0.713
SP = Standard Procedure; RP = Rapid Procedure; SW – Stepped – wedge; ICC – intra class correlation coefficient.									

16.2.5.5 Figure 5: Non-inferiority margin as a difference in proportions versus non-inferiority margin as an odds ratio

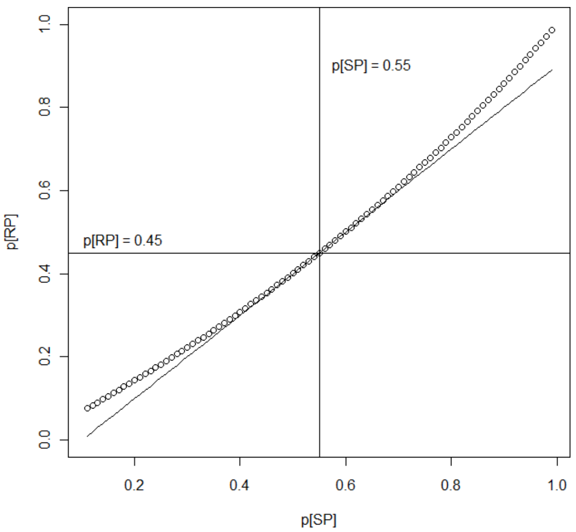

In Figure 5, the vertical and horizontal reference lines represent the proportion of successes in the Rapid Procedure (p[RP] = 0.45) and proportion of successes in the Standard Procedure (p[SP] = 0.55) used to calculate the margin of non-inferiority in terms of odds ratios [OR=0.67]. The straight line represents the margin of non-inferiority as a difference in proportions i.e., the line p[RP]-p[SP] = -10%. The curved line represents the margin of non-inferiority as an odds ratio i.e. odds[RP]/odds[SP] = 0.67. The graph suggests that the margin of non-inferiority as a difference in proportions versus odds ratio, in general, corresponds to similar probabilities of success except for extreme values of p[SP] and p[RP] (i.e. less than 0.3 or greater than 0.7).

16.2.6 Conclusion

The optimized stepped-wedge design, with 6 sites (clusters) and 5 periods of time (steps), enrolling a total of 450 participants (15 participants per cluster per time period), assuming 0.55 probability of success in the SP and 0.70 in the RP and a non-inferiority margin of 10%, will provide 88% or more power to show non-inferiority of RP to SP. For the sample size, and probabilities of success hypothesized for CTN0097, power to show RP is non-inferior to SP is at least 88%, irrespective of the value of the ICC. In addition, the study is adequately powered to account for simulated site drop-out, lower rates of enrollment or for slightly smaller true treatment effects.

16.3 Statistical Methods for Primary and Secondary Outcomes

16.3.1 Primary Outcome Analysis

As is standard for the analysis of stepped-wedge designs (Barker *et al.*, 2016) the primary outcome analysis for non-inferiority will be performed using a generalized linear mixed effects model with a logistic link, similar to the model used for the power calculations. The log odds of a participant receiving the first XR-NTX injection (yes/no) will be modeled as a function of which treatment they received (RP vs SP), which study month it is (to control for secular trends), and a random effect for site to control for nesting of participants within site. We will reject the null of inferiority of RP to

SP if the lower 95% confidence limit of the odds ratio of success [odds(RP)/odds(SP)] exceeds 0.67. Supplemental analyses related to the primary objective will test for a treatment*study month interaction, which, if significant, would indicate differential impact of treatment after a longer period of experience. Sensitivity analyses of the primary outcome may be performed to account for different correlation structures.

16.3.2 Secondary Outcome Analysis:

Secondary outcomes will include receiving 2nd and 3rd monthly injections of naltrexone, and opioid withdrawal symptoms and adverse events during and in the month after XR-NTX initiation. These outcomes will be modeled using similar mixed effects logistics regression methods as above. Note that the analysis of secondary outcomes before first XR-NTX injection will be performed using all enrolled participants using an intent-to-treat population (ITT) whereas the analyses of secondary outcomes after receipt of first XR-NTX injection will be performed for all enrolled participants as well as for a subset of the ITT population i.e., all enrolled participants who successfully received the scheduled XR-NTX injections (inducted population).

16.4 Implementation Outcome Analysis

Implementation outcomes, such as patients' ratings of acceptability and satisfaction with treatment, and clinicians' ratings of knowledge and attitudes toward XR-NTX and XR-NTX induction methods before and after sites' protocol participation, will be modelled as continuous outcomes using mixed effects linear regression models, again with random intercepts for site. Additional implementation outcome measures including acceptability and barriers measured qualitatively are detailed in Section 7.3.

16.5 Significance Testing

The primary analysis focuses on estimation of the treatment difference and uses and α=5% criterion for significance. There are several secondary outcomes; however, multiple comparisons will not be adjusted for since these are not part of the study's primary objective.

16.6 Interim Analysis

Interim analysis for futility is not planned, as a secondary aim is to place confidence limits on the difference in XR-NTX initiation success rates between Standard vs. Rapid methods, and greater precision of this estimate is desirable, regardless of whether the null hypothesis can be rejected. Also, a stepped-wedge requires all steps to be completed to estimate the treatment effect. The Investigators and DSMB will monitor SAEs.

16.7 Exploratory Analysis

Participant-level factors will be examined with moderator analysis to identify factors predicting success vs. failure to initiate XR-NTX. Site-level factors as measured with the implementation measures will also be explored as moderators of successful initiation. A nested prediction model will be created with participant-level factors nested within sites, and site level factors as gathered from implementation assessments (like staff knowledge and attitudes).

16.8 Prediction Models

Several factors are predicted to be associated with successful XR-NTX induction including severity of opioid use disorder based on route of use (IV vs. non-IV) with IV users having a more severe disorder, type of opioid (fentanyl vs. heroin vs. prescription opioid) with fentanyl users being most severe and prescription opioid users least severe, as well as psychiatric and substance use disorder comorbidity.

We will explore prediction in terms of patient level factors that predict success vs. failure to initiate XR-NTX. We are also interested in site level factors, measured with the implementation measures. Though with 6 sites power for that would be limited. It could be a nested prediction model with patients nested within sites, and site level factors (like staff knowledge and attitudes) in the model.

16.9 Missing Data and Dropouts

The primary outcome, whether or not a participant receives the XR-NTX while inpatient, is not expected to have missing data. Other outcome variables (opioid and other substance use over time, craving, mood, etc.) will have missing data due to missed visits and dropout from treatment and from study participation. The generalized linear model, or mixed effects model framework that will be used for analyses, works with what data are gathered, and assumes missing data are missing at random. For selected secondary outcome analyses, sensitivity analyses will be considered to examine the stability of estimated treatment effects in the face of departures from the assumption of missing at random.

16.10 Demographic and Baseline Characteristics

Baseline demographic and clinical variables will be summarized for participants enrolled in the active medication phase of the trial. Descriptive summaries of the distribution of continuous baseline variables will be presented with percentiles (median, 25th and 75th percentiles), and with mean and standard deviation. Categorical variables will be summarized in terms of frequencies and percentages.

Demographic variables will include: gender, age, race, ethnicity, educational level, employment status, marital status.

Baseline clinical characteristics will include: opioid dependence severity, severity of other substance use, severity of mood/anxiety symptoms, severity of opioid withdrawal symptoms, current/past co-occurring psychiatric disorders, current medical disorders, history of legal problems, currently under legal supervision (parole, probation, or mandated).

16.11 Safety Analysis

SAEs will be summarized by body system and preferred term using MedDRA codes (per The Medical Dictionary for Regulatory Activities). Targeted Safety Events (TSEs) will be coded if feasible. For a list of TSEs refer to Section 12.3.10. TSEs and SAEs will be presented as: (1) the number and proportion of participants experiencing at least one incidence of each event overall; and (2) the total number of each event overall in tabular form. Listings of SAEs will be sorted by

system organ class (SOC), and preferred term (PT). Detail in these listings will include severity, relationship to study medication(s), and action taken, as available.

17.0 REGULATORY COMPLIANCE, REPORTING AND MONITORING

17.1 Statement of Compliance

This trial will be conducted in compliance with the current version of the protocol, in full conformity with the ethical principles outlined in the Declaration of Helsinki, the Protection of Human Subjects described in the International Council for Harmonisation Good Clinical Practice (GCP) guidelines, applicable United States (US) Code of Federal Regulations (CFR), and NIDA Terms and Conditions of Award, and all other applicable state, local, and federal regulatory requirements. The Principal Investigator will assure that no deviation from, or changes to the protocol will take place without prior agreement from the Sponsor and documented approval from the Institutional Review Board (IRB), except where necessary to eliminate an immediate hazard(s) to the trial participants. An Operations Manual will be provided as a reference guide and study quality assurance tool.

17.2 Institutional Review Board Approval

Prior to initiating the study, participating site investigators will obtain written approval from the Ethics Review Committee (ERC) or Institutional Review Board (IRB) to conduct the study at their respective site, which will include approval of the study protocol. If changes to the study protocol become necessary, protocol amendments will be submitted in writing by the investigators for IRB approval prior to implementation. In addition, IRBs will approve all consent forms, recruitment materials, and any materials given to the participant, and any changes made to these documents throughout study implementation. Approval of both the protocol and the consent form(s) must be obtained before any participant is consented. For changes to the consent form, a decision will be made regarding whether previously consented participants need to be re-consented. IRB continuing review will be performed annually, or at a greater frequency contingent upon the complexity and risk of the study. Each site principal investigator is responsible for maintaining copies of all current IRB approval notices, IRB-approved consent documents, and approval for all protocol modifications. These materials must be received by the investigator prior to the initiation of research activities at the site and must be available at any time for audit. Unanticipated problems involving risk to study participants will be promptly reported to and reviewed by the IRB of record, according to its usual procedures.

This study will utilize a single IRB as the IRB of record for the protocol and will provide study oversight in accordance with 45 CFR 46. Participating institutions will agree to rely on the single IRB of Record and will enter into reliance/authorization agreements for Protocol CTN-0097. The single IRB will follow written procedures for reporting its findings and actions to appropriate officials at each participating institution.

17.3 Informed Consent

The informed consent process is a means of providing study information to each prospective participant and allows for an informed decision about participation in the study. Informed consent continues throughout the individual’s study participation. The informed consent form(s) will include all of the required elements of informed consent and may contain additional relevant consent elements and NIDA CCTN specific additional elements. Each study site must have the study

informed consent(s) approved by the IRB of record. Prior to initial submission to the IRB and with each subsequent consent revision, the consent form(s) must be sent to the Clinical Coordinating Center (CCC) and the Lead Node (LN) to confirm that each consent form contains the required elements of informed consent as delineated in 21 CFR 50.25(a) and CFR 46.116(b), as well as pertinent additional elements detailed in 21 CFR 50.25(b) and 45 CFR 46.116(c) and any applicable CCTN requirements. Every study participant is required to sign a valid, IRB-approved current version of the study informed consent form prior to the initiation of any study related procedures. The site must maintain the original signed informed consent for every participant in a locked, secure location that is in compliance with all applicable IRB and institutional policies and that is accessible to the study monitors. Every study participant should be given a copy of the signed consent form.

During the informed consent process, research staff will explain the study to the potential participant and provide the potential participant with a copy of the consent form to read and keep for reference. All participants will receive a verbal explanation in terms suited to their comprehension of the purposes, procedures, and potential risks of the study and their rights as research participants. Extensive discussion of risks and possible benefits will be provided to the participants. Participants will have the opportunity to carefully review the written consent form and ask questions prior to signing. The participants should have the opportunity to discuss the study with their family and close friends or think about it prior to agreeing to participate. If the participant is interested in participating in the study, a qualified staff member will review each section of the IRB-approved informed consent form in detail and answer any questions the participant may pose. The participant, or participant's legally authorized representative, will consent by signing and dating the consent document. The person obtaining consent and a witness, if required by the IRB of record, will also sign and date the consent document. It is strongly recommended that another research staff member review the consent after it is signed to ensure that the consent is properly executed and complete. Staff members delegated by the PI to obtain informed consent must be listed on the Delegation of Responsibility and Staff Signature Log and must be approved by the IRB, if required. All persons obtaining consent must have completed appropriate GCP and Human Subjects Protection training, as mandated by NIDA standard operating procedures.

The informed consent form must be updated or revised whenever important new safety information is available, or whenever the protocol is amended in a way that may affect participants' participation in the trial. The rights and welfare of the participants will be protected by emphasizing to them that the quality of their medical care will not be adversely affected if they decline to participate in this study. The participant will be informed that their participation is voluntary, and they may withdraw from the study at any time, for any reason, without penalty. Individuals who refuse to participate or who withdraw from the study will be treated without prejudice. Study sites will be responsible for maintaining signed consent forms as source documents for quality assurance review and regulatory compliance.

The study does not preempt any applicable federal, state, or local laws which require additional information to be disclosed in order for informed consent to be legally effective. It is in conformance with 42 CFR 2.52, which allows for research-related provisions with regard to the disclosure of substance use disorder patient identifying information in the absence of the informed consent

process and HIPAA authorization.

17.4 Quality Assurance Monitoring

In accordance with federal regulations, the study sponsor is responsible for ensuring proper monitoring of an investigation and ensuring that the investigation is conducted in accordance with the protocol. Qualified monitors will oversee aspects of site conformity to make certain the site staff is operating within the confines of the protocol, and in accordance with GCP. This includes but is not limited to protocol compliance, documentation auditing, monitoring of drug disposition, and ensuring the informed consent process is being correctly followed and documented. Non-conformity with protocol and federal regulations will be reported as a protocol deviation and submitted to the study sponsor and study IRB of record, as applicable, for further review.

17.5 Participant and Data Confidentiality

Participant confidentiality and privacy are strictly held in trust by the participating investigators, their staff, the safety and oversight monitor(s), and the sponsor(s) and funding agency, and will be maintained in accordance with all applicable federal regulations and/or state/Commonwealth law and regulations. This confidentiality is extended to the data being collected as part of this study. Data that could be used to identify a specific study participant will be held in strict confidence within the research team. No personally-identifiable information from the study will be released to any unauthorized third party without prior written approval of the sponsor/funding agency and the participant.

All research activities will be conducted in as private a setting as possible.

The study monitor, other authorized representatives of the sponsor or funding agency, representatives of the Institutional Review Board (IRB), regulatory agencies or representatives from companies or organizations supplying the product, may inspect all documents and records required to be maintained by the investigator, including but not limited to, medical records (office, clinic, or hospital) and pharmacy records for the participants in this study. The clinical study site will permit access to such records.

Participant records will be held confidential by the use of study codes for identifying participants on CRFs, secure storage of any documents that have participant identifiers, and secure computing procedures for entering and transferring electronic data. The study participant's contact information will be securely stored at each clinical site and on the electronic data capture system for internal use during the study. At the end of the study, all records will continue to be kept in a secure location for as long a period as denoted in Section 17.12 Records Retention and Requirements.

By signing the protocol signature page, the investigator affirms that information furnished to the investigator by NIDA will be maintained in confidence and such information will be divulged to the IRB/Privacy Board, Ethical Review Committee, or similar expert committee; affiliated institution; and employees only under an appropriate understanding of confidentiality with such board or committee, affiliated institution and employees.

17.5.1 Certificate of Confidentiality

To further protect the privacy of study participants, the Secretary, Health and Human Services (HHS), has issued a Certificate of Confidentiality (CoC) to all researchers engaged in biomedical, behavioral, clinical or other human subjects research funded wholly or in part by the federal government. Recipients of NIH funding for human subjects research are required to protect identifiable research information from forced disclosure per the terms of the NIH Policy (see https://humansubjects.nih.gov/coc/index). This protects participants from disclosure of sensitive information (e.g., drug use). It is the NIH policy that investigators and others who have access to research records will not disclose identifying information except when the participant consents or in certain instances when federal, state, or local law or regulation requires disclosure. NIH expects investigators to inform research participants of the protections and the limits to protections provided by a Certificate issued by this Policy.

17.5.2 Health Insurance Portability and Accountability Act (HIPAA)

Study sites may be required by their institutions to obtain authorization from participants for use of protected health information (PHI). Sites will be responsible for communicating with the IRBs or Privacy Boards of record and obtaining the appropriate approvals or waivers to be in regulatory compliance. Releases of participant identifying information that are permitted by the HIPAA regulations, but which are prohibited by other applicable federal regulations and/or state/Commonwealth law and regulation, are prohibited.

17.6 Investigator Assurances

Each site must have on file an active Federalwide Assurance (FWA) with the HHS Office for Human Research Protection setting forth the commitment of the organization to establish appropriate policies and procedures for the protection of human research subjects in alignment with 45 CFR 46, Subpart A, with documentation sent to NIDA or its designee. Research covered by these regulations cannot proceed in any manner prior to NIDA receipt of certification that the research has been reviewed and approved by the IRB provided for in the assurance (45 CFR 46.103). Prior to initiating the study, the Principal Investigator at each study site will sign a protocol signature page and investigator agreement, providing assurances that the study will be performed according to the standards stipulated therein.

17.6.1 Financial Disclosure/Conflict of Interest

All investigators will comply with the requirements of 42 CFR Part 50, Subpart F to ensure that the design, conduct, and reporting of the research will not be biased by any conflicting financial interest. Everyone with decision-making responsibilities regarding the protocol will confirm to the sponsor annually that they have met their institutional financial disclosure requirements.

17.7 Clinical Monitoring

Investigators will host periodic visits by NIDA contract monitors who will examine whether study procedures are conducted appropriately, and that study data are generated, documented and reported in compliance with the protocol, GCP, and applicable regulations. These monitors will audit, at mutually agreed upon times, regulatory documents, case report forms (CRFs), informed

consent forms and corresponding source documents for each participant. Monitors will have the opportunity and ability to review any study-associated document or file.

NIDA-contracted monitors will assess whether submitted data are accurate and in agreement with source documentation and will also review regulatory/essential documents such as correspondence with the IRB. Areas of particular concern will be participant informed consent forms, protocol adherence, reported safety events and corresponding assessments, and Principal Investigator oversight and involvement in the trial. Reports will be prepared following the visit and forwarded to the site principal investigator, the Lead Investigator and NIDA CCTN.

Qualified node personnel (Node QA monitors) or other designated party(ies) will provide site management for each site during the trial. Node QA staff or other designated party(ies) will audit source documentation, including informed consent forms and HIPAA forms. This will take place as specified by the local protocol team, node PI or lead team and will occur as often as needed to help prevent, detect, and correct problems at the study sites. Node QA personnel will verify that study procedures are properly followed and that site personnel are trained and able to conduct the protocol appropriately. If the node personnel's review of study documentation indicates that additional training of site study personnel is needed, node QA personnel will undertake or arrange for that training. Details of the contract, node QA and data monitoring are found in the study QA monitoring plan.

17.8 Inclusion of Women and Minorities

The study sites should aim and take steps to enroll a diverse study population. If difficulty is encountered in recruiting an adequate number of women and/or minorities, the difficulties involved in recruitment will be discussed in national conference calls and/or face-to-face meetings, encouraging such strategies as linkages with medical sites and/or treatment programs that serve large numbers of women and/or minorities, advertising in newspapers or radio stations with a high female/minority readership/listening audience, etc.

17.9 Prisoner Certification

As per 45 CFR 46 Subpart C, there are additional protections pertaining to prisoners as study participants. A prisoner is defined as any individual involuntarily confined or detained in a penal institution. The term is intended to encompass individuals sentenced to such an institution under a criminal or civil statute, individuals detained in other facilities by virtue of statutes or commitment procedures which provide alternatives to criminal prosecution or incarceration in a penal institution, and individuals detained pending arraignment, trial, or sentencing. In order to meet these additional protections, the study team will obtain certification from the Office for Human Research Protections (OHRP) to enroll prisoners (see Section 8.3) and follow-up with participants who become prisoners during the course of the study, as necessary.

17.10 Regulatory Files

The regulatory files should contain all required regulatory documents, study-specific documents, and all important communications. Regulatory files will be checked at each participating site for regulatory document compliance prior to study initiation, throughout the study, as well as at study

closure.

17.11 Records Retention and Requirements

Research records for all study participants (e.g., case report forms, source documents, signed consent forms, audio and video recordings, and regulatory files) will be maintained by the investigator in a secure location for a minimum of 3 years after the study is completed and closed. These records are also to be maintained in compliance with IRB, state and federal requirements, whichever is longest. The Sponsor and Lead Investigator must be notified in writing and acknowledgment from these parties must be received by the site prior to the destruction or relocation of research records.

17.12 Reporting to Sponsor

The site principal investigator agrees to submit accurate, complete, legible and timely reports to the Sponsor, as required. These include, but are not limited to, reports of any changes that significantly affect the conduct or outcome of the trial or increase risk to study participants. Safety reporting will occur as previously described. At the completion of the trial, the Lead Investigator will provide a final report to the Sponsor.

17.13 Audits

The Sponsor has an obligation to ensure that this trial is conducted according to good clinical research practice guidelines and may perform quality assurance audits for protocol compliance. The Lead Investigator and authorized staff from the Greater New York Node; the National Institute on Drug Abuse Clinical Trials Network (NIDA CTN, the study sponsor); NIDA's contracted agents, monitors or auditors; and other agencies such as the Department of Health and Human Services (HHS), the Office for Human Research Protection (OHRP) and the Institutional Review Board of record may inspect research records for verification of data, compliance with federal guidelines on human participant research, and to assess participant safety.

17.14 Study Documentation

Each participating site will maintain appropriate study documentation (including medical and research records) for this trial, in compliance with ICH E6 R2 and regulatory and institutional requirements for the protection of confidentiality of participants. Study documentation includes all case report forms, workbooks, source documents, monitoring logs and appointment schedules, sponsor-investigator correspondence, and signed protocol and amendments, Ethics Review Committee or Institutional Review Board correspondence and approved consent form and signed participant consent forms. As part of participating in a NIDA-sponsored study, each site will permit authorized representatives from NIDA and regulatory agencies to examine (and when permitted by law, to copy) clinical records for the purposes of quality assurance reviews, audits, and evaluation of the study safety, progress, and data validity.

Source documents include <u>all</u> recordings of observations or notations of clinical activities and all reports and records necessary for the evaluation and reconstruction of the clinical research study. Whenever possible, the original recording of an observation should be retained as the source

document; however, a photocopy is acceptable provided that it is a clear, legible, and exact duplication of the original document.

17.15 Protocol Deviations

This protocol defines a protocol deviation as any noncompliance with the clinical trial protocol. The noncompliance may be either on the part of the participant, the investigator, or the study site staff. As a result of deviations, corrective actions will be developed by the site and implemented promptly.

These practices are consistent with ICH GCP:

- Section 4.5 Compliance with Protocol, subsections 4.5.1, 4.5.2, and 4.5.3
- Section 5.1 Quality Assurance and Quality Control, subsection 5.1.1
- Section 5.20 Noncompliance, subsections 5.20.1, and 5.20.2.

Any departure from procedures and requirements outlined in the protocol will be classified as either a major or minor protocol deviation. Variations in clinical procedures are not specifically mandated by the protocol and are part of the implementation outcome and therefore will not be considered protocol deviations. The difference between a major and minor protocol deviation has to do with the seriousness of the event and the corrective action required. A minor protocol deviation is considered an action (or inaction) that by itself is not likely to affect the scientific soundness of the investigation or seriously affect the safety, rights, or welfare of a study participant. Major protocol deviations are departures that may compromise the participant safety, participant rights, inclusion/exclusion criteria or the integrity of study data and could be cause for corrective actions if not rectified or prevented from re-occurrence. Sites will be responsible for developing corrective action plans for both major and minor deviations as appropriate. Those corrective action plans may be reviewed/approved by the Lead Node and the CCC with overall approval by the IRB of record as needed. All protocol deviations will be monitored at each site for (1) significance, (2) frequency, and (3) impact on the study objectives, to ensure that site performance does not compromise the integrity of the trial.

All protocol deviations will be recorded in the Electronic Data Capture (EDC) system via the Protocol Deviation CRF. The CCC, DSC and the Lead Investigator must be contacted immediately if an unqualified or ineligible participant is randomized into the study.

Additionally, each site is responsible for reviewing the IRB of record's definition of a protocol deviation or violation and understanding which events need to be reported. Sites must recognize that the CTN and IRB definition of a reportable event may differ and act accordingly in following all reporting requirements for both entities.

17.16 Safety Monitoring

The Lead Investigator (LI) may appoint a Site Medical Clinician (MD, DO, NP, or PA) for this study, who will review or provide consultation for each Targeted Safety Event (TSE) and Serious Adverse Event (SAE) as needed. These reviews will include an assessment of the possible

relatedness of the event to the study intervention or other study procedures. The Site Medical Clinician will also provide advice for decisions to exclude, refer, or withdraw participants as required. In addition, NIDA will assign a Safety Monitor/Medical Monitor to this protocol to independently review the safety data and present it to the DSMB for periodic review. The Safety Monitor/Medical Monitor will determine which safety events require expedited reporting to NIDA and the DSMB. This will include events that are serious, related and unexpected. The study staff will be trained to monitor for and report TSEs and SAEs.

Each of the sites has established practices for managing medical and psychiatric emergencies, and the study staff will continue to utilize these procedures. Treatment providers at each site will be responsible for monitoring participants for possible clinical deterioration or other problems, and for implementing appropriate courses of action.

17.16.1 Data and Safety Monitoring Board (DSMB)

An independent CTN DSMB will examine accumulating data to assure protection of participants' safety while the study's scientific goals are being met. The CTN DSMB is responsible for conducting periodic reviews of accumulating safety and efficacy data. It will determine whether there is support for continuation of the trial, or evidence that study procedures should be changed, or if the trial should be halted, for reasons relating to the safety of the study participants, the efficacy of the treatment under study, or inadequate trial performance (e.g., poor recruitment).

17.16.2 Safety Monitor/Medical Monitor

The CCC Safety Monitor/Medical Monitor is responsible for reviewing all targeted safety events (TSEs) and serious adverse events (SAEs) reported. All SAEs will be reviewed within one business day of being reported in Advantage eClinical. The Safety Monitor/Medical Monitor will also indicate concurrence or not with the details of the report provided by the site. Where further information is needed the Safety Monitor/Medical Monitor will discuss the event with the site. Reviews of SAEs will be conducted in the Advantage eClinical data system and will be a part of the safety database. All TSEs are reviewed on a regular basis to observe trends or unusual events.

The CCC Safety Monitor/Medical Monitor will in turn report events to the sponsor if the event meets the definition of an expedited event. Reports will be generated and presented for Data Safety Monitoring Board (DSMB) meetings.

17.16.3 Safety Events

Standard definitions for adverse events and serious adverse events, their identification, characterization regarding severity and relationship to therapy and processing are described in Appendix A.

For the purpose of this study, events included in the following list of targeted safety events should be recorded on the Targeted Safety Events form and reported in the data system:

- Fall event (related to medical/psychiatric condition such as dizziness, confusion with head injury)
- Acute change in mental status (i.e. disorientation, amnesia, cerebrovascular accident, coma)
- Acute medical complication likely exacerbated by the stress of withdrawal (i.e. hypertensive crisis, hypotensive event with medical sequelae such as fall and/or requiring urgent fluid resuscitation, severe chest pain, acute respiratory decompensation, asthma attack, diabetic ketoacidosis, severe hypoglycemia, severe electrolyte abnormalities (hyper-/hyponatremia, hyper-/hypokalemia), precipitated withdrawal
- Acute psychiatric symptoms (i.e. psychosis, hypomania, severe agitation, violence)

Targeted safety events will also be captured on the appropriate, related study specific forms (for example, COWS, SOWS, PHQ-9, Medication Injection Site Abnormality Log and Overdose Questionnaire).

Spontaneous reporting of withdrawal symptoms (including any withdrawal precipitated by the study medication) by the participant will be captured on scheduled specific structured questionnaires.

Targeted safety events will not be duplicate-reported as an adverse event unless meeting the SAE definition.

17.16.4 Serious Adverse Events (SAEs)

All safety events will be evaluated to determine if they meet the criteria for an SAE (see Appendix A). For the purpose of this study, the following events will not be reported as an SAE. Detox admissions will be recorded on study specific forms in the data system. These events will be reported to the single IRB (and any local IRB(s), if applicable) per IRB guidelines:

- Admissions for detoxification
- Admission for labor and delivery
- Admission for elective or pre-planned surgery

17.16.5 Known Potential Toxicities of Study Medication/Intervention

All medications used in the proposed study are FDA-approved and widely used clinically.

18.0 DATA MANAGEMENT

18.1 Design and Development

This protocol will utilize a centralized Data and Statistics Center (DSC). The DSC will be responsible for development of the electronic case report forms (eCRFs), development and validation of the clinical study database, ensuring data integrity, and training site and participating node staff on applicable good clinical data management procedures. Advantage eClinical, a web-based distributed data entry system, will be implemented to collect eCRFs. This system will be developed to ensure that guidelines and regulations surrounding the use of computerized systems used in clinical trials are upheld. In addition, quantitative implementation assessments will be collected in Qualtrics^XM, a commercial off-the-shelf data collection system. Of note, the DSC will not be responsible for providing support for data collection and analysis of qualitative interviews or coaching progress notes. The remainder of this section provides an overview of the data management plan associated with this protocol.

18.2 Site Responsibilities

The data management responsibilities of each individual site will be specified by the DSC and outlined in the Advantage eClinical User's Guide.

18.3 Data Center Responsibilities

The DSC will 1) develop a data management plan and will conduct data system development and data management activities in accordance with that plan, 2) provide final guided source documents and eCRFs for the collection of all data required by the study, 3) develop data dictionaries for each eCRF that will comprehensively define each data element, 4) conduct ongoing data monitoring and quality assurance activities on study data from all participating sites, 5) monitor any preliminary analysis data cleaning activities as needed, and 6) rigorously monitor final study data cleaning for all data collected in eClinical.

18.4 Data Collection

Data will be collected at the study sites on source documents and entered by the site into eCRFs in Advantage eClinical or Qualtrics^XM, or will be collected via direct entry into the eCRF. In the event that Advantage eClinical or Qualtrics^XM are not available, the DSC will provide the sites with a final set of guided source documents and completion instructions. Data entry into Advantage eClinical should be completed according to the instructions provided and project specific training. The investigator is responsible for maintaining accurate, complete and up-to-date records, and for ensuring the completion of the eCRFs for each research participant.

18.5 Data Acquisition and Entry

Completed forms and electronic data will be entered into the Advantage eClinical system in accordance with the Advantage eClinical User's Guide. Only authorized individuals who have adequately completed required training shall have access to eCRFs.

18.6 Data Editing

Completed data will be entered into Advantage eClinical. If incomplete or inaccurate data are found, a query will be generated to the appropriate site for their response. Sites will resolve data inconsistencies and errors and enter all corrections and changes into Advantage eClinical.

18.7 Data Transfer/Lock

Study participant research data, which is for purposes of statistical analysis and scientific reporting, will be transmitted to and stored at the DSC. This will not include the participant's contact or identifying information. Rather, individual participants and their research data will be identified by a unique study identification number. The study data entry and study management systems used by clinical sites and by DSC staff will be secured and password protected.

Data will be transmitted by the DSC to the NIDA central data repository as requested by NIDA. The DSC will conduct final data quality assurance checks and "lock" the study database from further modification. The final analysis dataset will be returned to NIDA, as requested, for storage and archive.

18.8 Data Training

The training plan for site staff includes provisions for training on assessments, eCRF completion guidelines, data management procedures, and the use of Advantage eClinical.

18.9 Data Quality Assurance

To address the issue of data entry quality, the DSC will follow a standard data monitoring plan. An acceptable quality level prior to study lock or closeout will be established as a part of the data management plan. Data quality summaries will be made available during the course of the protocol.

19.0 PUBLIC ACCESS AND DATA SHARING PLAN

This study will comply with the NIH Data Sharing Policy and Implementation Guidance (https://grants.nih.gov/grants/policy/data_sharing/data_sharing_guidance.htm) and (for HEAL-funded studies) the HEAL Public Access and Data Sharing Policy (https://heal.nih.gov/about/public-access-data). Investigators will also register and report results of the trial in ClinicalTrials.gov, consistent with the requirements of the Policy on the Dissemination of NIH-Funded Clinical Trial Information and the Clinical Trials Registration (https://grants.nih.gov/policy/clinical-trials/reporting/understanding/nih-policy.htm).

Primary data for this study will be available to the public in the NIDA data repository, per NIDA CTN policy. For more details on data sharing please visit https://datashare.nida.nih.gov/.

The primary outcome(s) publication will be included along with study underlying primary data in the data share repository, and it will also be deposited in PubMed Central http://www.pubmedcentral.nih.gov/ per NIH Policy (http://publicaccess.nih.gov/).

The planning, preparation, and submission of publications will follow the policies of the Publications Committee of the CTN. Considerations for ensuring confidentiality of any shared data are described in Section 17.6**.**

20.0 PROTOCOL SIGNATURE PAGE

SPONSOR'S REPRESENTATIVE (CCTN SCIENTIFIC OFFICER OR DESIGNEE)

Printed Name	Signature	Date

ACKNOWLEDGEMENT BY INVESTIGATOR:

- I am in receipt of version 3.0 of the protocol and agree to conduct this clinical study in accordance with the design and provisions specified therein.
- I agree to follow the protocol as written except in cases where necessary to protect the safety, rights, or welfare of a participant, an alteration is required, and the sponsor and IRB have been notified prior to the action.
- I will ensure that the requirements relating to obtaining informed consent and institutional review board (IRB) review and approval in 45 CFR 46 are met.
- I agree to personally conduct or supervise this investigation at this site and to ensure that all site staff assisting in the conduct of this study are adequately and appropriately trained to implement this version of the protocol and that they are qualified to meet the responsibilities to which they have been assigned.
- I agree to comply with all the applicable federal, state, and local regulations regarding the obligations of clinical investigators as required by the Department of Health and Human Services (DHHS), the state, and the IRB.

SITE'S PRINCIPAL INVESTIGATOR

Printed Name	Signature	Date

Clinical Site Name

Node Affiliation

21.0 REFERENCES

Aarons, G. A. (2004). Mental health provider attitudes toward adoption of evidence-based practice: the Evidence-Based Practice Attitude Scale (EBPAS). *Ment Health Serv Res* **6**, 61-74.

Amodei, N. & Lamb, R. J. (2004). Convergent and concurrent validity of the Contemplation Ladder and URICA scales. *Drug Alcohol Depend* **73**, 301-6.

Baker, R., Camosso-Stefinovic, J., Gillies, C., Shaw, E. J., Cheater, F., Flottorp, S. & Robertson, N. (2010). Tailored interventions to overcome identified barriers to change: effects on professional practice and health care outcomes. *Cochrane Database Syst Rev*, CD005470. **Binswanger, I. A., Blatchford, P. J., Mueller, S. R. & Stern, M. F.** (2013). Mortality after prison release: opioid overdose and other causes of death, risk factors, and time trends from 1999 to 2009. *Ann Intern Med* **159**, 592-600.

Bisaga, A., Mannelli, P., Sullivan, M. A., Vosburg, S. K., Compton, P., Woody, G. E. & Kosten, T. R. (2018a). Antagonists in the medical management of opioid use disorders: Historical and existing treatment strategies. *Am J Addict* **27**, 177-187.

Bisaga, A., Mannelli, P., Yu, M., Nangia, N., Graham, C. E., Tompkins, D. A., Kosten, T. R., Akerman, S. C., Silverman, B. L. & Sullivan, M. A. (2018b). Outpatient transition to extended- release injectable naltrexone for patients with opioid use disorder: A phase 3 randomized trial. *Drug Alcohol Depend* **187**, 171-178.

Brahen, L. S., Capone, T. J. & Capone, D. M. (1988). Naltrexone: lack of effect on hepatic enzymes. *J. Clin. Pharmacol.* **28**, 64-70.

Buntwal, N., Bearn, J., Gossop, M. & Strang, J. (2000). Naltrexone and lofexidine combination treatment compared with conventional lofexidine treatment for in-patient opiate detoxification. *Drug Alcohol Depend* **59**, 183-8.

Carnwath, T. & Hardman, J. (1998). Randomised double-blind comparison of lofexidine and clonidine in the out-patient treatment of opiate withdrawal. *Drug Alcohol Depend* **50**, 251-4.

Curran, G. M., Bauer, M., Mittman, B., Pyne, J. M. & Stetler, C. (2012). Effectiveness- implementation hybrid designs: combining elements of clinical effectiveness and implementation research to enhance public health impact. *Med Care* **50**, 217-26.

Damschroder, L. J. & Hagedorn, H. J. (2011). A guiding framework and approach for implementation research in substance use disorders treatment. *Psychol Addict Behav* **25**, 194- 205.

Delgadillo, J., Payne, S., Gilbody, S., Godfrey, C., Gore, S., Jessop, D. and Dale, V., 2012. Brief case finding tools for anxiety disorders: Validation of GAD-7 and GAD-2 in addictions treatment. *Drug and alcohol dependence*, *125*(1-2), pp.37-42.

Fagerstrom, K. O. (1978). Measuring degree of physical dependence to tobacco smoking with reference to individualization of treatment. *Addict. Behav.* **3**, 235-41.

Gerra, G., Zaimovic, A., Giusti, F., Di Gennaro, C., Zambelli, U., Gardini, S. & Delsignore, R. (2001). Lofexidine versus clonidine in rapid opiate detoxification. *J Subst Abuse Treat* **21**, 11-7. **Glasgow, R. E., Vogt, T. M. & Boles, S. M.** (1999). Evaluating the public health impact of health promotion interventions: the RE-AIM framework. *Am J Public Health* **89**, 1322-7.

Gossop, M., Stewart, D., Treacy, S. & Marsden, J. (2002). A prospective study of mortality among drug misusers during a 4-year period after seeking treatment. *Addiction* **97**, 39-47.

Gowing, L., Ali, R. & White, J. M. (2010). Opioid antagonists under heavy sedation or anaesthesia for opioid withdrawal. *Cochrane Database Syst Rev*, CD002022.

Gowing, L., Farrell, M., Ali, R. & White, J. M. (2016). Alpha(2)-adrenergic agonists for the management of opioid withdrawal. *Cochrane Database Syst Rev*, CD002024.

Handley, M. A., Schillinger, D. & Shiboski, S. (2011). Quasi-experimental designs in practice- based research settings: design and implementation considerations. *J Am Board Fam Med* **24**, 589-96.

Haug, N. A., Shopshire, M., Tajima, B., Gruber, V. & Guydish, J. (2008). Adoption of evidence-based practices among substance abuse treatment providers. *J Drug Educ* **38**, 181-92.

Hefner K, Case A, Matthews A. & Moran L. (2021). Development of a COVID-19 Impact Assessment for the National Drug Abuse Treatment Clinical Trials Network (NDAT CTN). Society for Clinical Trials 42nd Annual Meeting, Virtual.

Helfrich, C. D., Li, Y. F., Sharp, N. D. & Sales, A. E. (2009). Organizational readiness to change assessment (ORCA): development of an instrument based on the Promoting Action on Research in Health Services (PARIHS) framework. *Implement Sci* **4**, 38.

Johnson, K. E., Coleman, K., Phillips, K. E., Austin, B. T., Daniel, D. M., Ridpath, J., Schaefer, J. & Wagner, E. H. (2014). Development of a facilitation curriculum to support primary care transformation: the "coach medical home" curriculum. *Med Care* **52**, S26-32.

Kahn, A., Mumford, J. P., Rogers, G. A. & Beckford, H. (1997). Double-blind study of lofexidine and clonidine in the detoxification of opiate addicts in hospital. *Drug Alcohol Depend* **44**, 57-61.

Kirchner, J. E., Ritchie, M. J., Pitcock, J. A., Parker, L. E., Curran, G. M. & Fortney, J. C. (2014). Outcomes of a partnered facilitation strategy to implement primary care-mental health. *J Gen Intern Med* **29 Suppl 4**, 904-12.

Kroenke K., Spitzer R.L., Williams J.B. The PHQ-9: validity of a brief depression severity measure. J Gen Intern Med. 2001 Sep;16(9):606-13.

Krupitsky, E., Nunes, E. V., Ling, W., Illeperuma, A., Gastfriend, D. R. & Silverman, B. L. (2011). Injectable extended-release naltrexone for opioid dependence: a double-blind, placebo-controlled, multicentre randomised trial. *Lancet* **377**, 1506-13.

Kurland, A. A. & McCabe, L. (1976). Rapid detoxification of the narcotic addict with naloxone hydrochloride. A preliminary report. *J Clin Pharmacol* **16**, 66-74.

LaBrie, J. W., Quinlan, T., Schiffman, J. E. & Earleywine, M. E. (2005). Performance of alcohol and safer sex change rulers compared with readiness to change questionnaires. *Psychol Addict Behav* **19**, 112-5.

Lee, J. D., Friedmann, P. D., Kinlock, T. W., Nunes, E. V., Boney, T. Y., Hoskinson, R. A., Jr., Wilson, D., McDonald, R., Rotrosen, J., Gourevitch, M. N., Gordon, M., Fishman, M., Chen, D. T., Bonnie, R. J., Cornish, J. W., Murphy, S. M. & O'Brien, C. P. (2016). Extended- Release Naltrexone to Prevent Opioid Relapse in Criminal Justice Offenders. *N Engl J Med* **374**, 1232-42.

Lee, J. D., Nunes, E. V., Jr., Novo, P., Bachrach, K., Bailey, G. L., Bhatt, S., Farkas, S., Fishman, M., Gauthier, P., Hodgkins, C. C., King, J., Lindblad, R., Liu, D., Matthews, A. G., May, J., Peavy, K. M., Ross, S., Salazar, D., Schkolnik, P., Shmueli-Blumberg, D., Stablein, D., Subramaniam, G. & Rotrosen, J. (2018). Comparative effectiveness of extended-release naltrexone versus buprenorphine-naloxone for opioid relapse prevention (X:BOT): a multicentre, open-label, randomised controlled trial. *Lancet* **391**, 309-318.

Löwe, B., Decker, O., Müller, S., Brähler, E., Schellberg, D., Herzog, W. and Herzberg, P.Y., 2008. Validation and standardization of the Generalized Anxiety Disorder Screener (GAD-7) in the general population. *Medical care*, pp.266-274.

Mannelli, P., Wu, L. T., Peindl, K. S., Swartz, M. S. & Woody, G. E. (2014). Extended release naltrexone injection is performed in the majority of opioid dependent patients receiving outpatient induction: a very low dose naltrexone and buprenorphine open label trial. *Drug Alcohol Depend* **138**, 83-8.

Miller, C.J., Barnett, M.L., Braumann, A.A., Gutner, C.A., & Wiltsey-Stirman, S. (2021). The FRAME-IS: a framework for documenting modifications to adaptations to implementation strategies in healthcare. *Implement Sci* 16, 36.

Naderi-Heiden, A., Gleiss, A., Backer, C., Bieber, D., Nassan-Agha, H., Kasper, S. & Frey, R. (2012). Mortality and employment after in-patient opiate detoxification. Eur Psychiatry 27, 294- 300.

Nunes, E. V., Gordon, M., Friedmann, P. D., Fishman, M. J., Lee, J. D., Chen, D. T., Hu, M. C., Boney, T. Y., Wilson, D. & O'Brien, C. P. (2017). Relapse to opioid use disorder after inpatient treatment: Protective effect of injection naltrexone. *J Subst Abuse Treat*.

Palinkas, L. A., Horwitz, S. M., Green, C. A., Wisdom, J. P., Duan, N. & Hoagwood, K. (2015). Purposeful Sampling for Qualitative Data Collection and Analysis in Mixed Method Implementation Research. *Adm Policy Ment Health* **42**, 533-44.

Perry, C., Damschroder, L., Hemler, J., Woodson, T., Ono, S., & Cohen, D. (2019). Specifying and comparing implementation strategies across seven large implementation interventions: a practical application of theory. *Implement Sci* 21;14(1):32.

Pierce, M., Bird, S. M., Hickman, M., Marsden, J., Dunn, G., Jones, A. & Millar, T. (2016). Impact of treatment for opioid dependence on fatal drug-related poisoning: a national cohort study in England. *Addiction* **111**, 298-308.

Powell, B., Waltz, T., Chinman, M., Damschroder, L., Smith, J., Matthieu M., Proctor, E., & Kirchner, J. (2015). A refined compilation of implementation strategies: results from the Expert Recommendations for Implementing Change (ERIC) project. *Implement Sci* 12;10(1):21.

Proctor, E., Powell, B., & McMillen, J. (2013). Implementation strategies: recommendations for specifying and reporting. *Implement Sci* 1;8(1):139.

Resnick, R. B., Kestenbaum, R. S., Washton, A. & Poole, D. (1977). Naloxone-precipitated withdrawal: a method for rapid induction onto naltrexone. *Clin. Pharmacol. Ther.* **21**, 409-13. **Robinson, S. M., Sobell, L. C., Sobell, M. B. & Leo, G. I.** (2014). Reliability of the Timeline Followback for cocaine, cannabis, and cigarette use. *Psychol Addict Behav* **28**, 154-62.

Rudolf, G., Walsh, J., Plawman, A., Gianutsos, P., Alto, W., Mancl, L. & Rudolf, V. (2018). A novel non-opioid protocol for medically supervised opioid withdrawal and transition to antagonist treatment. Am J Drug Alcohol Abuse 44, 302-309.

San, L., Cami, J., Peri, J. M., Mata, R. & Porta, M. (1990). Efficacy of clonidine, guanfacine and methadone in the rapid detoxification of heroin addicts: a controlled clinical trial. *Br J Addict* **85**, 141-7.

Sigmon, S. C., Bisaga, A., Nunes, E. V., O'Connor, P. G., Kosten, T. & Woody, G. (2012). Opioid detoxification and naltrexone induction strategies: recommendations for clinical practice. *Am J Drug Alcohol Abuse* **38**, 187-99.

Sobell, L. C. & Sobell, M. B. (1992). Timeline Follow-back: A technique for assessing self- reported ethanol consumption. In *Measuring Alcohol Consumption: Psychosocial and Biological Methods* (ed. J. P. Allen and R. Z. Litten), pp. 41-72. Humana Press: Totowa, NJ.

Stetler, C. B., Damschroder, L. J., Helfrich, C. D. & Hagedorn, H. J. (2011). A Guide for applying a revised version of the PARIHS framework for implementation. *Implement Sci* **6**, 99.

Strang, J., McCambridge, J., Best, D., Beswick, T., Bearn, J., Rees, S. & Gossop, M. (2003). Loss of tolerance and overdose mortality after inpatient opiate detoxification: follow up study.*BMJ* **326**, 959-60.

Sullivan, M., Bisaga, A., Pavlicova, M., Choi, C. J., Mishlen, K., Carpenter, K. M., Levin, F. R., Dakwar, E., Mariani, J. J. & Nunes, E. V. (2017). Long-Acting Injectable Naltrexone Induction: A Randomized Trial of Outpatient Opioid Detoxification With Naltrexone Versus Buprenorphine. *Am J Psychiatry* **174**, 459-467.

Sullivan, M. A., Bisaga, A., Pavlicova, M., Carpenter, K. M., Choi, C. J., Mishlen, K., Levin, F. R., Mariani, J. J. & Nunes, E. V. (2019). A Randomized Trial Comparing Extended-Release Injectable Suspension and Oral Naltrexone, Both Combined With Behavioral Therapy, for the Treatment of Opioid Use Disorder. *Am J Psychiatry* **176**, 129-137.

Tanum, L., Solli, K. K., Latif, Z. E., Benth, J. S., Opheim, A., Sharma-Haase, K., Krajci, P. & Kunoe, N. (2017). Effectiveness of Injectable Extended-Release Naltrexone vs Daily Buprenorphine-Naloxone for Opioid Dependence: A Randomized Clinical Noninferiority Trial. *JAMA Psychiatry* **74**, 1197-1205.

Thompson, J. A., Fielding, K., Hargreaves, J. & Copas, A. (2017). The optimal design of stepped wedge trials with equal allocation to sequences and a comparison to other trial designs. *Clin Trials* **14**, 639-647.

Umbricht, A., Montoya, I. D., Hoover, D. R., Demuth, K. L., Chiang, C. T. & Preston, K. L. (1999). Naltrexone shortened opioid detoxification with buprenorphine. *Drug Alcohol Depend* **56**, 181-90.

Van de Glind, G., van den Brink, W., Koeter, M.W., Carpentier, P.J., van Emmerik-van Oortmerssen, K., Kaye, S., Skutle, A., Bu, E.T.H., Franck, J., Konstenius, M. and Moggi, F., 2013. Validity of the Adult ADHD Self-Report Scale (ASRS) as a screener for adult ADHD in treatment seeking substance use disorder patients. *Drug and alcohol dependence*, *132*(3), pp.587-596.

Waltz, T., Powell, B., Matthieu, M., Damschroder, L., Chinman, M., Smith, J., Proctor, E., & Kirchner J. (2015). Use of concept mapping to characterize relationships among implementation strategies and assess their feasibility and importance: results from the Expert Recommendations for Implementing Change (ERIC) study. *Implement Sci* 7;10(1):109.

Weathers FW, Litz BT, Keane TM, Palmieri PA, Marx BP, Schnurr PP. (2013). The PTSD Checklist for *DSM-5* (PCL-5). Scale available from the National Center for PTSD at www.ptsd.va.gov.

Wesson, D. R. & Ling, W. (2003). The Clinical Opiate Withdrawal Scale (COWS). *J Psychoactive Drugs* **35**, 253-9.

Winter, R. J., Stoove, M., Degenhardt, L., Hellard, M. E., Spelman, T., Jenkinson, R., McCarthy, D. R. & Kinner, S. A. (2015). Incidence and predictors of non-fatal drug overdose after release from prison among people who inject drugs in Queensland, Australia. *Drug Alcohol Depend* **153**, 43-9.

Wittkampf, K.A., Baas, K.D., van Weert, H.C., Lucassen, P. and Schene, A.H., 2011. The psychometric properties of the panic disorder module of the Patient Health Questionnaire (PHQ-PD) in high-risk groups in primary care. *Journal of affective disorders*, *130*(1-2), pp.260-267.

22.0 APPENDIX A: ADVERSE EVENT REPORTING AND PROCEDURES

Each participating site's Principal Investigator is responsible for study oversight, including ensuring human research subject protection by designating appropriately qualified and trained study personnel to assess, report, and monitor adverse events.

22.1 Definition of Adverse Events and Serious Adverse Events

An **adverse event** (AE) is any untoward medical occurrence in humans, whether or not considered study medication/intervention related which occurs during the conduct of a clinical trial. Any change from baseline in clinical status, ECGs, lab results, x-rays, physical examinations, etc., that is considered clinically significant by the site medical clinician are considered AEs.

Suspected adverse reaction is any adverse event for which there is a reasonable possibility that the study medication/intervention caused the adverse event. A reasonable possibility implies that there is evidence that the study medication/intervention caused the event.

Adverse reaction is any adverse event caused by the study medication/intervention.

An **adverse event, suspected adverse reaction,** or **adverse reaction** is considered "**serious**" (i.e., a serious adverse event, serious suspected adverse reaction or serious adverse reaction) if, in the view of either the site medical clinician or sponsor, it:

1) Results in death: A death occurring during the study or which comes to the attention of the study staff during the protocol-defined follow-up period, whether or not considered caused by the study medication/intervention, must be reported.
2) Is life-threatening: Life-threatening means that the study participant was, in the opinion of the medical clinician or sponsor, at immediate risk of death from the reaction as it occurred and required immediate intervention.
3) Requires inpatient hospitalization or prolongation of existing hospitalization.
4) Results in persistent or significant incapacity or substantial disruption of the ability to conduct normal life functions.
5) Is a congenital abnormality or birth defect.
6) Is an important medical event that may not result in one of the above outcomes but may jeopardize the health of the study participant or require medical or surgical intervention to prevent one of the outcomes listed in the above definition of serious event.

22.2 Definition of Expectedness

Any adverse event is considered "unexpected" if it is not listed in the investigator brochure or the package insert or is not listed at the specificity or severity that has been observed. If neither is available, then the protocol and consent are used to determine an unexpected adverse event.

22.3 Pregnancy

Pregnancy will be followed within the context of this study. Any pregnancies that occur while a participant is enrolled in the study will be captured on a pregnancy CRF and not separately reported as an AE or SAE. Pregnant women should be engaged by their site medical provider in a shared decision-making process to determine whether they remain on medication for OUD and which medication to use. The pregnancy followed until an outcome is known.

22.4 Medical and Psychiatric History

A thorough medical and psychiatric history during the screening phase should record any chronic, acute, or intermittent preexisting or current illnesses, diseases, symptoms, or laboratory signs of the participant, to avoid reporting pre-existing conditions as new events and to assist in the assessment of worsening in intensity or severity of these conditions that would indicate a TSE or SAE.

Stable chronic conditions, such as arthritis, which are present prior to clinical trial entry and do not worsen are not considered TSEs.

22.5 Site's Role in Eliciting and Reporting Safety Events

Appropriately qualified and trained personnel will elicit participant reporting of TSEs and SAEs at each study visit designated to collect these events. TSEs (medical and/or psychiatric) assessment will initiate with participant consent and follow-up of ongoing events will continue through resolution or 30 days post last study visit. Study personnel will obtain as much information as possible about the reported TSE/SAE to complete the TSE/SAE forms and will consult with the Safety Monitor/Medical Monitor as warranted.

Standard reporting, within 7 days of the site becoming aware of the event, is required for reportable TSEs. Expedited reporting (within 24 hours of their occurrence and/or site's knowledge of the event) is required for reportable SAEs (including death and life-threatening events). Sites are responsible for reporting SAEs to the IRB of record, per the IRB or record's guidelines.

Sites are required to enter reportable TSEs and SAEs in the Advantage eClinical system. The TSE form is used to capture reportable TSEs. The AE/SAE form set is used to capture SAEs (as defined in the protocol). In this protocol, only SAEs are being reported on the AE/SAE form set. Additional information may need to be gathered to evaluate SAEs and to complete the appropriate CRFs and the summary. This process may include obtaining hospital discharge reports, medical records, autopsy records or any other type records or information necessary to provide a complete and clear picture of the serious event and events preceding and following the event. If the SAE is not resolved or stable at the time of the initial report or if new information becomes available after the initial report, follow-up information must be submitted as soon as possible.

Any serious adverse reactions will be followed until resolution or stabilization for as long as possible beyond the end of the study. If the study team is unable to follow up with individuals with reportable safety events, every effort will be made to connect these individuals with the appropriate community-based treatment.

22.6 Site's Role in Assessing Severity and Causality of Adverse Events

Appropriately qualified and trained study personnel will conduct an initial assessment of seriousness, severity, and causality when eliciting participant reporting of adverse events. A study medical clinician will review reportable safety events for seriousness, severity, and causality on at least a weekly basis.

22.7 Guidelines for Assessing Severity

The severity of an adverse event refers to the intensity of the event.

Grade 1	Mild	Transient or mild discomfort (typically <48 hours), no or minimal medical intervention/therapy required, hospitalization not necessary (non-prescription or single-use prescription therapy may be employed to relieve symptoms, e.g., aspirin for simple headache, acetaminophen for post-surgical pain)
Grade 2	Moderate	Mild to moderate limitation in activity, some assistance may be needed; no or minimal intervention/therapy required, hospitalization possible but unlikely.
Grade 3	Severe	Marked limitation in activity, some assistance usually required; medical intervention/ therapy required, hospitalization possible.

22.8 Guidelines for Determining Causality

The site medical clinician will use the following question when assessing causality of an adverse event to study medication/intervention where an affirmative answer designates the event as a suspected adverse reaction:

Is there a reasonable possibility that the study medication/intervention caused the event?

22.9 Site's Role in Monitoring Safety Events

Local quality assurance monitors will review study sites and respective study data on a regular basis and will promptly advise sites to report any previously unreported safety issues and ensure that the reportable safety-related events are being followed to resolution and reported appropriately. Staff education, re-training or appropriate corrective action plan will be implemented at the participating site when unreported or unidentified reportable TSEs or serious events are discovered, to ensure future identification and timely reporting by the site.

22.10 Sponsor's Role in Safety Management Procedures of AEs/SAEs

A NIDA-assigned Safety Monitor/Medical Monitor is responsible for reviewing all serious adverse event reports. All reported SAEs will generate an e-mail notification to the Safety Monitor/Medical

Monitor, Lead Investigator, and designees. All SAEs will be reviewed by the Safety Monitor/Medical Monitor in Advantage eClinical and, if needed, additional information will be requested. The Safety Monitor/Medical Monitor will also report events to the sponsor and the DSMB as required. The DSMB will receive summary reports of all targeted safety events and SAEs annually, at a minimum. The DSMB or the NIDA assigned Safety Monitor/Medical Monitor may also request additional and updated information. Details regarding specific targeted safety events or SAEs, their treatment and resolution, will be summarized by the Safety Monitor/Medical Monitor in writing for review by the sponsor and DSMB. Subsequent review by the Safety Monitor/Medical Monitor, DSMB, FDA and ethics review committee or IRB, the sponsor, or relevant local regulatory authorities may also suspend further trial treatment at a site. The study sponsor, DSMB and FDA retain the authority to suspend additional enrollment and treatments for the entire study as applicable.

22.11 Regulatory Reporting for a non-IND study

If an SAE meets the expedited reporting criteria (serious and unexpected suspected adverse reactions), the Safety Monitor/Medical Monitor on behalf of the sponsor will provide an SAE narrative summary.

22.11.1 Reporting to the Data and Safety Monitoring Board

The DSMB will receive listing of TSEs and summary reports of all SAEs at a frequency requested by the DSMB, but at least annually. Furthermore, the DSMB will be informed of expedited reports of SAEs.

22.12 Participant Withdrawal

The site medical clinician must apply his/her clinical judgment to determine whether or not an adverse event is of sufficient severity to require that the participant be withdrawn from further study medication administration/study intervention. The site medical clinician should consult with the site.

Principal Investigator, the Lead Investigator and/or Safety Monitor/Medical Monitor as needed. If necessary, a site medical clinician may suspend any trial treatments and institute the necessary medical therapy to protect a participant from any immediate danger. A participant may also voluntarily withdraw from treatment due to what he/she perceives as an intolerable adverse event or for any other reason. If voluntary withdrawal is requested, the participant will be asked to complete an end-of-medication visit to assure safety and to document end-of-medication outcomes and will be given recommendations for medical care and/or referrals to treatment, as necessary.

22.13 Adverse Event Reporting (Chart)

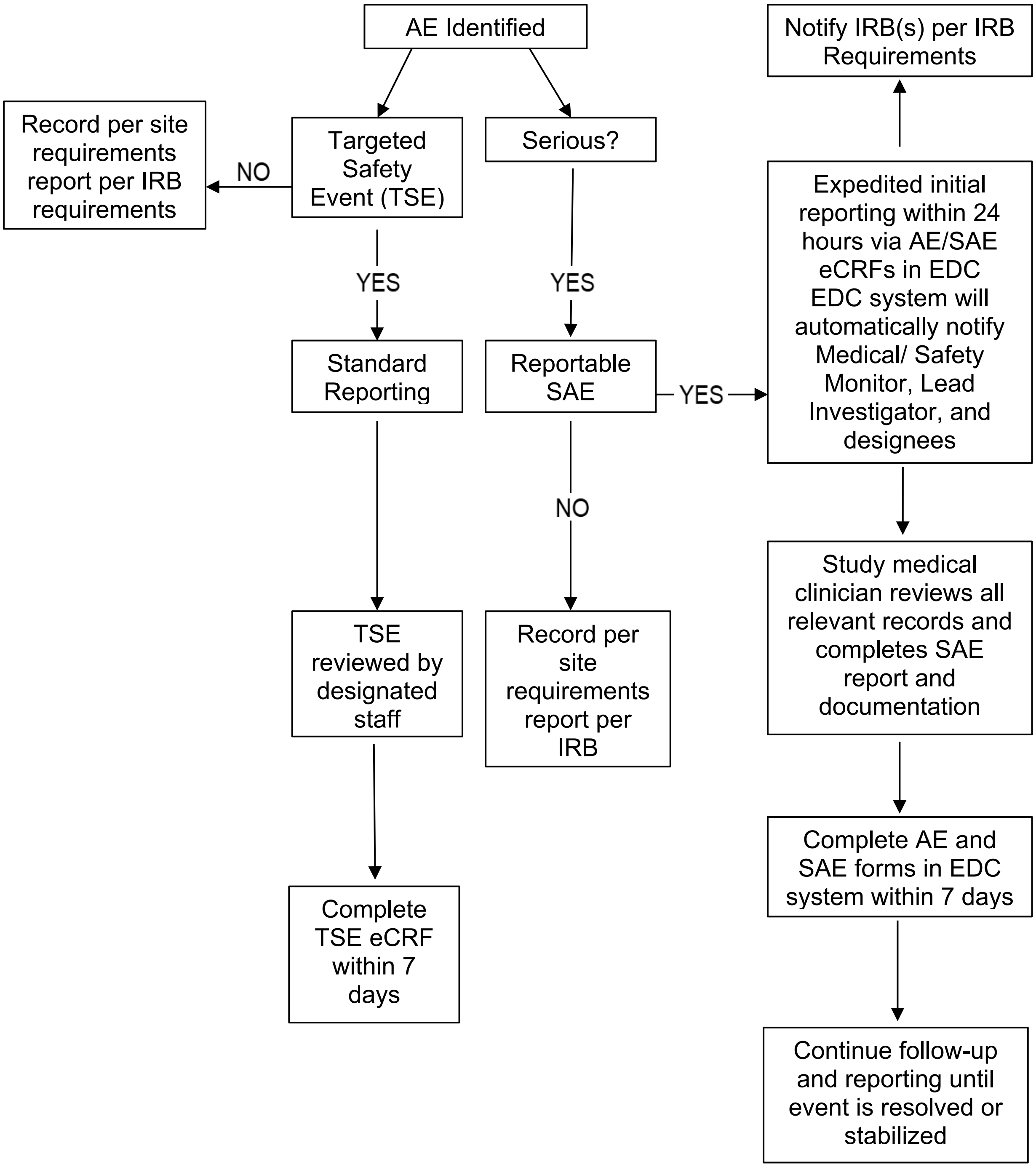

23.0 APPENDIX B: DATA AND SAFETY MONITORING PLAN

1.0 BRIEF STUDY OVERVIEW

This is an open-label, multi-center, stepped-wedge cluster randomized trial. It will be conducted in 6 CTN-affiliated Community Treatment Programs (CTPs) that provide inpatient/residential detoxification services and have the capacity to provide ongoing outpatient treatment with extended-release naltrexone (XR-NTX). All sites will first implement the control procedure (Standard, 14-day procedure; SP), to establish the within-site comparison condition, and then at selected staggered time-points (steps) will switch to implementing only the experimental procedure (Rapid, 5-7-day procedure: RP). Implementation of RP at study sites will be staggered by 4 months and the order in which sites will cross-over to RP will be randomly chosen. The primary goal of the study is to determine whether the Rapid method of initiating treatment with XR-NTX is non-inferior to the standard method. Secondary objectives include comparing RP and SP for craving, withdrawal, retention, abstinence, depression and anxiety, and safety measures. Other exploratory outcomes include predictors of induction success, economic analyses, and barriers to implementation.

2.0 OVERSIGHT OF CLINICAL RESPONSIBILITIES

A. Site Principal Investigator

Each participating site's Principal Investigator (PI) is responsible for study oversight, including ensuring human research subject protection by designating appropriately qualified, trained research staff and medical clinicians to assess, report, and monitor adverse events.

All safety events (TSEs and SAEs) occurring during the course of the clinical trial will be collected, documented, and reported by the investigator or sub-investigators according to the protocol.

The occurrence of TSAEs and serious adverse events (SAEs) will be assessed at each clinic visit during the study. Serious adverse events will be followed until considered resolved or stable.

Reportable TSAEs are required to be entered into the data system within 7 days of the site staff becoming aware of the event. Reportable SAEs (including death and life-threatening events) are required to be entered into the data system within 24 hours of site's knowledge of the event.

B. CCC Safety Monitor/Medical Monitor

The NIDA CTN Clinical Coordinating Center's (CCC) Safety Monitor/Medical Monitor or designee is responsible for reviewing all targeted safety events and serious adverse events reported. The CCC Safety Monitor/Medical Monitor is alerted via email each time an SAE is reported in the EDC. All SAEs will be reviewed at the time they are reported in the EDC. The Safety Monitor/Medical Monitor or designee will also indicate concurrence or not with the details of the report provided by the site PI. Where further information is needed, the Safety Monitor/Medical Monitor or designee will discuss the event with the site staff. Reviews of SAEs by the CCC Safety Monitor/Medical Monitor or designee will be documented in the Advantage eClinical data system and will be a part of the safety database. All TSEs are reviewed on a weekly basis to observe trends or unusual events.

C. Voluntary Regulatory Reporting in non-IND Trials:

For non-IND trials, if an event meets expedited reporting criteria (serious, related and unexpected) the CCC Safety Monitor/Medical Monitor will provide an SAE narrative summary.

D. Data and Safety Monitoring Board (DSMB)

The NIDA CTN DSMB affiliated with this trial will be responsible for conducting periodic reviews of accumulating safety, trial performance, and outcome data. Reports will be generated and presented for Data and Safety Monitoring Board (DSMB) meetings. The DSMB will receive listings of targeted safety events and narratives of all SAEs at a frequency requested by the DSMB, but at least annually. Furthermore, the DSMB will be informed of expedited reports of SAEs. The DSMB will make recommendations to NIDA CCTN as to whether there is sufficient support for continuation of the trial, evidence that study procedures should be changed, or evidence that the trial (or a specific site) should be halted for reasons relating to safety of the study participants or inadequate trial performance (e.g., poor recruitment).

Following each DSMB meeting, the NIDA CCTN will communicate the outcomes of the meeting, based on DSMB recommendations, in writing to the study Lead Investigator. This communication summarizing study safety information will be submitted to participating IRBs.

E. Quality Assurance (QA) Monitoring

The monitoring of the study site(s) will be conducted on a regular basis using a combination of NIDA CCTN CCC monitors (if applicable) and the local Node QA Monitors (if applicable). Investigators will host periodic visits for the monitors. The purpose of these visits is to assess compliance with the protocol, GCP requirements, and other applicable regulatory requirements, as well as to document the integrity of the trial progress. The investigative site will provide direct access to all trial related sites (e.g., pharmacy, research office), source data/documentation, and reports for the purpose of monitoring and auditing by the monitors, as well as inspection by local and regulatory authorities. Areas of particular concern will be the review of inclusion/exclusion criteria, participant Informed Consent Forms, protocol adherence, safety monitoring, IRB reviews and approvals, regulatory documents, participant records, study drug accountability, and Principal Investigator supervision and involvement in the trial. The monitors will interact with the site staff to identify issues and re-train the site as needed to enhance research quality.

Site Visit Reports will be prepared by the NIDA CCC monitors following each site visit. These reports will be sent to the site Principal Investigator, the study Lead Investigator and NIDA CCTN.

Local Node QA site visit reports will be prepared following each site visit, as applicable. These reports are sent to those entities required of them by the Lead Investigative team, generally including the Lead Investigator, site Principal Investigator, Node PI and a CCC representative, usually the protocol specialist for the study.

F. Management of Risks to Participants Confidentiality

Confidentiality of participant records will be secured by the use of study codes for identifying participants on CRFs, and secure storage of any documents that have participant identifiers on

site, as well as secure computing procedures for entering and transferring electronic data. The documents or logs linking the study codes with the study participant on site will be kept locked/securely stored separately from the study files and the medical records. No identifying information will be disclosed in reports, publications or presentations.

3.0 Information That Meets Reporting Requirements

The consent form will specifically state the types of information that are required for reporting and that the information will be reported as required. These include suspected or known sexual or physical abuse of a child or elders, or threatened violence to self and/or others.

4.0 Participant Protection

The site's study clinician or other designated and qualified individual will evaluate all pertinent screening assessments prior to participant enrollment to ensure that the participant is eligible and safe to enter the study. Safety events and concomitant medications will be assessed and documented at each study visit. Individuals who experience an safety event that compromises safe participation in a study will be discontinued from further medication administration/intervention and provided referrals for other treatment or to specialized care. Study personnel will request that the participant complete an end-of medication visit to assure safety and to document end-of-medication outcomes.

5.0 Pregnancy

Participants who are found to be pregnant during the screening will not be enrolled in the study. Pregnant women should be engaged by their site medical provider in a shared decision-making process to determine whether they remain on medication for OUD and which medication to use. The site staff will follow the participant until an outcome of the pregnancy is known and reported.

6.0 Study Specific Risks

Risk of Overdose

Opioid detoxification carries a moderately high risk of relapse and a risk of opioid overdose if one uses a significant amount of opioids after losing tolerance. Self-administration of large doses of opiates may override the blockade produced by naltrexone resulting in an opiate overdose with its attendant risks, including respiratory depression and death. Participants will be warned of the severe danger of using opiates, including trying to override the blockade. Also, participants who have stopped naltrexone and resume opiates will not be tolerant initially, so that the quantities of opiates self-administered prior to treatment, when they were tolerant, may be quite dangerous in the non-tolerant state. Participants will be warned of this. Participants who self-administer opiates to the point of somnolence or stupor will be removed from the trial and referred to inpatient detoxification or agonist maintenance treatment. Until such individuals are accepted for treatment elsewhere, they will remain at the site where they will be offered therapy and treated in the open label manner for the duration of trial.

Risks of Naltrexone

The possible side effects of naltrexone in individuals with opioid dependence may include: a period

of more severe withdrawal symptoms due to precipitated withdrawal, difficulty sleeping, anxiety, nervousness, abdominal pain/cramps, nausea and/or vomiting, low energy, joint muscle pain, and headaches. Less common side effects include loss of appetite, diarrhea, constipation, increased thirst, increased energy, feeling down, irritability, dizziness, skin rash, delayed ejaculation and chills.

Naltrexone has been associated with reversible hepatocellular injury indicated by elevated liver enzymes when administered at doses substantially greater than the 50mg per day dose recommended for maintenance treatment of OUD and proposed for the present study. When used in the recommended dose range in opiate-dependent patients, this risk is remote (Brahen *et al.*, 1988). Naltrexone is therefore contraindicated in patients with acute hepatitis or liver failure, and such patients are excluded from the study. Patients with hepatic enzyme levels greater than 5 times the upper limit of normal are therefore excluded.

In the event of a medical emergency requiring opiate analgesia, an individual on naltrexone will require higher doses of opiates than normally administered. Participants will be informed of this and will be given a naltrexone medication card to carry in their wallet.

Naltrexone does not block intoxicating effects of other drugs such as cocaine, sedatives, or alcohol and it does not reduce the risk of using these substances.

<u>Risks of Opioid Withdrawal</u>

During XR-NTX induction procedure, participants are likely to experience mild to moderate severity opioid withdrawal, and it is possible that the withdrawal will be more severe in the group receiving Rapid XR-NTX induction. Opioid withdrawal may cause agitation, bone and joint aches, restlessness, runny nose and tearing, nausea, vomiting, diarrhea, anxiety or irritability, elevated pulse and blood pressure and other signs of sympathetic arousal, and sometimes confusion. Adjunctive medications will be used to alleviate the severity of withdrawal.

<u>Risks of Ancillary Medications</u>

Opioid withdrawal symptoms are treated with clonidine, an alpha-2-adrenergic receptor agonist which reduces sympathetic nervous system output produced by opiate withdrawal; clonazepam, a benzodiazepine which reduces the anxiety and dysphoria and permits sleep; and zolpidem or trazodone for insomnia. Clonidine may produce somnolence, dry mouth, drowsiness, dizziness, constipation, hypotension, syncope, bradycardia and QT prolongation. Clonazepam commonly may produce somnolence, poor muscle control, loss of inhibition, constipation, respiratory infection and infection. Trazodone may produce drowsiness, sedation, dizziness, constipation, and blurred vision. Zolpidem may produce drowsiness, sedation, and headache. The procedure is conducted in an inpatient setting with a medical professional present at all times, to permit close monitoring of vital signs and mental status.

<u>Risks of XR-NTX injection</u>

Risk of XR-NTX injection involves possible irritation at the injection site. XR-NTX injections may be followed by a period of, suicidal thoughts, pain, tenderness, induration, redness, swelling,

abscess, sterile abscess, and pruritus. Cases of serious injection site reactions, some of which involved surgical intervention, have been reported.

3.0 DATA MANAGEMENT PROCEDURES

This protocol will utilize a centralized Data and Statistics Center (DSC). A web-based distributed data entry model will be implemented. This electronic data capture system (Advantage eClinical) will be developed to ensure that guidelines and regulations surrounding the use of computerized systems in clinical trials are upheld.

4.0 DATA AND STATISTICS CENTER RESPONSIBILITIES

The DSC will: 1) develop and apply data management procedures to ensure the collection of accurate and good-quality data, 2) provide source documents and electronic Case Report Forms (eCRFs) for the collection of all data required by the study, 3) develop data dictionaries for each eCRF that will comprehensively define each data element, 4) prepare instructions for the use of Advantage eClinical and for the completion of eCRFs, 5) conduct ongoing monitoring and quality assurance activities on study data collected from all participating sites, and 6) perform data cleaning activities prior to the final study database lock for assessments collected in Advantage eClinical.

A. DATA COLLECTION AND ENTRY

Data will be collected at the study sites on source documents and entered by the site into eCRFs in Advantage eClinical or will be collected via direct entry into the eCRF. In the event that Advantage eClinical is not available, the DSC will provide the sites with a final set of guided source documents with instructions for completion. Data will be entered into Advantage eClinical in accordance with the instructions provided during protocol-specific training and guidelines established by the DSC. Data entry into the eCRFs will be performed by authorized individuals who have satisfactorily completed the relevant training. Selected source documents and eCRFs may also require the investigator’s signature (wet or electronic). In some situations, data collected on source documents will not be entered into Advantage eClinical, but when it is entered, it will follow the guidelines stated above.

The Principal Investigator at the site is responsible for maintaining accurate, complete and up-to-date research records. In addition, the Principal Investigator is responsible for ensuring the timely completion of eCRFs for each research participant.

B. DATA MONITORING, CLEANING AND EDITING

eCRFs will be monitored for completeness and accuracy throughout the study. Dynamic reports listing missing values and missing forms are available to sites at all times in Advantage eClinical. These reports will be monitored regularly by the DSC. In addition, the DSC will identify inconsistencies within eCRFs and between eCRFs and post queries in Advantage eClinical on a scheduled basis. Sites will resolve data queries by entering all corrections and changes directly into Advantage eClinical or by verifying the data are correct as is.

As described above, the CCC will conduct regular monitoring visits, during which audits

comparing source documents to the data entered on the eCRF will be performed. Any discrepancies identified between the source document and the eCRF will be corrected by the site.

Trial progress and data status reports, which provide information on items such as recruitment, availability of primary outcome data, treatment exposure, attendance at long term follow-up visits, regulatory status, and data quality, will be generated daily and posted to a secure website. These reports are available to the site staff, local Node staff, the Lead Investigator, the coordinating centers, and NIDA CCTN, to monitor each site's progress on the study.

C. DATABASE LOCK AND TRANSFER

Study participant research data, which is for purposes of statistical analysis and scientific reporting, will be transmitted to and stored at the DSC. Individual participants and their research data will be identified by a unique study identification number; further, some identifiable data may be collected in eClinical. The study data entry and study management systems used by clinical sites and by DSC staff will be secured and password protected in compliance with regulatory standards.

At the conclusion of data collection for the study, the DSC will perform final data cleaning activities and will "lock" the study database from further modification. The final raw datasets will be transferred to the Lead Investigator or designee. De-identified versions of these datasets will also be provided to the NIDA CCTN-designated party for storage and archiving. These datasets may be posted on the NIDA Data Share website.

Reference: http://grants.nih.gov/grants/guide/notice-files/not98-084.html
