# Supplementary material for: Rapid Initiation of Injection Naltrexone for Opioid Use Disorder: A Stepped-Wedge Cluster Randomized Clinical Trial
Source: JAMA Netw Open. 2024 May 8;7(5):e249744. doi: 10.1001/jamanetworkopen.2024.9744 (PMC11079685; doi:10.1001/jamanetworkopen.2024.9744)
Supplement: Supplement 2. — eTable 1. Medication Regimen by Induction Procedure eTable 2. Summary of Adjunctive Medications by Induction Procedure eTable 3. Study Assessments for Participants (RP and SP) eTable 4. Reasons for Not Attempting XR-NTX Induction eAppendix 1. Rapid Procedure Example Order Set eAppendix 2. Shared Decision-Making Tool for Medications for Opioid Use Disorder [file jamanetwopen-e249744-s002.pdf]

Supplementary Online Content

Shulman M, Greiner MG, Tafessu HM, et al. Rapid initiation of injection naltrexone for opioid use disorder: a stepped-wedge cluster randomized clinical trial. *JAMA Netw Open*. 2024;7(5):e249744. doi:10.1001/jamanetworkopen.2024.9744

eTable 1. Medication Regimen by Induction Procedure

eTable 2. Summary of Adjunctive Medications by Induction Procedure

eTable 3. Study Assessments for Participants (RP and SP)

eTable 4. Reasons for Not Attempting XR-NTX Induction

This supplementary material has been provided by the authors to give readers additional information about their work.

© 2024 Shulman M et al. *JAMA Network Open.*

eTable 1: Medication regimen by induction procedure.

Protocol Day	Rapid Procedure (RP)	Standard Procedure (SP)
1	Buprenorphine 2-10mg	Buprenorphine 8 mg
2	-(washout)[a]	Buprenorphine 6 mg
3	Naltrexone 1 mg	Buprenorphine 4 mg
4	Naltrexone 2 mg	Buprenorphine 3 mg
5	Naltrexone 6 mg	Buprenorphine 2 mg
6	Naltrexone 6 mg and XR-naltrexone 380mg IM	-(washout)[a]
15		XR-naltrexone 380mg IM
	Scheduled adjunctive medications Days 1-6[b]	Adjunctive medications as per site's usual practices[c]

[a] Washout period with no opioids administered (24-hour period for RP; 7- to 10-day period for SP)

[b] Adjunctive medications for RP included: clonidine 0.1-0.2 mg every four hours (with hold parameters for systolic blood pressure < 90 mm Hg, or heart rate < 60 beats per minute), clonazepam 1 mg every six hours (with hold for oversedation), antinausea medication daily (prochlorperazine 10 mg or ondansetron 8 mg), sleep aid at bedtime (zolpidem 5-10 mg, trazodone 50-100 mg, or other sleep aids), and nicotine replacement therapy (patch and gum) if tobacco use. Additional doses of adjunctive medications could be given as needed and included antiemetics, sleep aids, and acetaminophen or ibuprofen.

[c] Adjunctive medications offered for SP varied based on sites' usual practices.

© 2024 Shulman M et al. *JAMA Network Open.*

eTable 2: Summary of adjunctive medications by induction procedure.

		Induction Procedure		
		**Standard (N = 190)**	**Rapid (N = 225)**	**Total (N = 415)**
Clonidine	Number of participants	131 (68.9%)	221 (98.2%)	352 (84.8%)
	Average number of days	4.7	5.3	5.1
	Average total daily dose (mg)	0.2	0.4	0.3
Clonazepam	Number of participants	45 (23.7%)	220 (97.8%)	265 (63.9%)
	Average number of days	6.2	4.7	4.9
	Average total daily dose (mg)	2.0	2.7	2.5
Other Benzodiazepines				
Diazepam	Number of participants	17 (8.9%)	2 (0.9%)	19 (4.6%)
	Average number of days	4.4	2.0	4.2
	Average total daily dose (mg)	10.8	18.8	11.2
Chlordiazepoxide	Number of participants	15 (7.9%)	22 (9.8%)	37 (8.9%)
	Average number of days	3.0	2.3	2.6
	Average total daily dose (mg)	77.8	102.0	90.5
Lorazepam	Number of participants	52 (27.4%)	88 (39.1%)	140 (33.7%)
	Average number of days	3.7	2.3	2.8
	Average total daily dose (mg)	2.1	2.5	2.3
Antiemetic				
Prochlorperazine	Number of participants	0 (0%)	42 (18.7%)	42 (10.1%)
	Average number of days	-	2.9	2.9
	Average total daily dose (mg)	-	11.5	11.5
Promethazine	Number of participants	51 (26.8%)	80 (35.6%)	131 (31.6%)
	Average number of days	5.6	4.3	4.8
	Average total daily dose (mg)	17.2	45.3	32.5

© 2024 Shulman M et al. *JAMA Network Open.*

		Induction Procedure		
		Standard (N = 190)	Rapid (N = 225)	Total (N = 415)
Ondansetron	Number of participants	32 (16.8%)	81 (36.0%)	113 (27.2%)
	Average number of days	1.7	4.3	3.5
	Average total daily dose (mg)	4.9	7.2	6.9
Antidiarrheal				
Loperamide	Number of participants	25 (13.2%)	96 (42.7%)	121 (29.2%)
Sleep agent				
Trazodone	Number of participants	134 (70.5%)	189 (84.0%)	323 (77.8%)
	Average number of days	5.9	4.4	5.0
	Average total daily dose (mg)	95.3	100.6	98.0
Zolpidem	Number of participants	0 (0%)	27 (12.0%)	27 (6.5%)
	Average number of days	-	1.9	1.9
	Average total daily dose (mg)	-	11.8	11.8
Mirtazapine	Number of participants	14 (7.4%)	7 (3.1%)	21 (5.1%)
Doxepin	Number of participants	2 (1.1%)	4 (1.8%)	6 (1.4%)
Melatonin	Number of participants	15 (7.9%)	8 (3.6%)	23 (5.5%)
Non-Steroidal Anti-Inflammatory Agent				
Ibuprofen	Number of participants	120 (63.2%)	187 (83.1%)	307 (74.0%)
Aspirin	Number of participants	2 (1.1%)	1 (0.4%)	3 (0.7%)
Naproxen	Number of participants	15 (7.9%)	2 (0.9%)	17 (4.1%)
Nicotine Replacement Therapy				
Nicotine patch	Number of participants	38 (20.0%)	27 (12.0%)	65 (15.7%)
Nicotine patch plus other	Number of participants	24 (12.6%)	1 (0.4%)	25 (6.0%)
Nicotine gum	Number of participants	6 (3.2%)	3 (1.3%)	9 (2.2%)
Nicotine lozenge	Number of participants	16 (8.4%)	0 (0%)	16 (3.9%)
Anxiety/Antihistamine Agents				
Hydroxyzine	Number of participants	90 (47.4%)	75 (33.3%)	165 (39.8%)

© 2024 Shulman M et al. *JAMA Network Open.*

		Induction Procedure		
		Standard (N = 190)	Rapid (N = 225)	Total (N = 415)
Diphenhydramine	Number of participants	25 (13.2%)	26 (11.6%)	51 (12.3%)
Other	Number of participants	6 (3.2%)	6 (2.7%)	12 (2.9%)
GABA Agents/Muscle Relaxants				
Gabapentin	Number of participants	57 (30.0%)	42 (18.7%)	99 (23.9%)
Pregabalin	Number of participants	1 (0.5%)	2 (0.9%)	3 (0.7%)
Baclofen	Number of participants	60 (31.6%)	55 (24.4%)	115 (27.7%)
Cyclobenzaprine	Number of participants	45 (23.7%)	48 (21.3%)	93 (22.4%)
Other	Number of participants	11 (5.8%)	58 (25.8%)	69 (16.6%)
Neuroleptics				
Quetiapine	Number of participants	41 (21.6%)	42 (18.7%)	83 (20.0%)
Olanzapine	Number of participants	3 (1.6%)	0 (0%)	3 (0.7%)
Risperidone	Number of participants	1 (0.5%)	0 (0%)	1 (0.2%)
Haloperidol	Number of participants	1 (0.5%)	3 (1.3%)	4 (1.0%)
Chlorpromazine	Number of participants	1 (0.5%)	0 (0%)	1 (0.2%)

© 2024 Shulman M et al. *JAMA Network Open.*

eTable 3: Study assessments for participants (RP and SP)[a]

Assessment	Frequency
Informed consent and medical release	Baseline
General Measures	
Inclusion/exclusion	Baseline, confirmed prior to RP/SP enrollment
Locator form	Baseline, post-induction Week 3, EOM
Demographics form	Baseline
PhenX Tier 1 assessments (30)	Baseline
Prisoner status assessment	Baseline, post-induction Week 3
Motivation scale	Baseline
End of induction form	At discharge from inpatient detoxification unit
End of medication treatment form	EOM
Treatment satisfaction survey	Post-induction Week 6
Study completion form	Post-induction Week 8
Safety and Medical Measures	
Medical and psychiatric history	Baseline
HIV and hepatitis assessments	Baseline
Pregnancy and birth control assessment	Baseline, before each XR-NTX injection
Fagerström test for nicotine dependence (FTND) (31)	Baseline
NIDA cannabis assessment	Baseline
DSM-5 criteria (18)	Baseline
Generalized anxiety disorder (GAD-7) questionnaire (32)	Baseline
Patient health questionnaire panic disorder (PHQ-PD) (33)	Baseline
PTSD checklist for DSM-5 (PCL-5) (34)	Baseline
ADHD self-report scale (ASRS) (35)	Baseline
Daily medication administration log	Baseline, daily during induction phase
Concomitant medications	Post-induction Weeks 1, 2, 3, 4, 6, 8
Adverse events and serious adverse events	Baseline, daily during induction phase, post-induction Weeks 1, 2, 3, 4, 6, 8
Medication injection site abnormality log	As needed throughout study
Opioid overdose questionnaire	Baseline, post-induction Weeks 1, 2, 3, 4, 6, 8
Fatal opioid overdose form	As needed throughout study
Death form	As needed throughout study
Treatment Compliance Measures	
Medical management log	At each study visit
Psychosocial participation log	At each study visit
Outcome Measures	
Timeline Followback (TLFB) for substance use (36)	Baseline, post-induction Weeks 1, 2, 3, 4, 6, 8

© 2024 Shulman M et al. *JAMA Network Open.*

Timeline Followback (TLFB) for MOUD	Baseline, post-induction Weeks 1, 2, 3, 4, 6, 8
Urine drug screen (UDS) (with fentanyl testing)	Baseline, before each XR-NTX injection
Clinical opiate withdrawal scale (COWS) (37)	Baseline, daily or more frequently (depending on RP or SP) during induction phase
Subjective opioid withdrawal scale (SOWS) (38)	Baseline, daily during induction phase, post-induction Weeks 1, 2, 3, 4, 6, 8
Visual analog scales (VAS) opioid craving	Baseline, daily during induction phase, post-induction Weeks 1, 2, 3, 4, 6, 8
Visual analog scales (VAS) opioid response	As needed throughout post-induction phase
Patient health questionnaire for depression (PHQ-9) (39)	Baseline, weekly during induction phase, post-induction Weeks 1, 2, 3, 5, 6, 8
Mental health assessment (for suicidality)	As needed throughout the study
Health Services Measures	
Patient-reported outcomes measurement information (PROMIS) (40)	Baseline, post-induction Weeks 4, 8
Non-medical and other services (NMS) (41,42)	Baseline, post-induction Weeks 4, 8
Genetics Measures	
Genetics sample	Baseline
Family origin assessment	Baseline

EOM=end of medication

[a]Site-level and implementation assessments will be described separately.

eTable 4: Reasons for not attempting XR-NTX Induction

Reason	*Count*	*Percent*
Prefers buprenorphine maintenance	848	34.7%
Prefers methadone maintenance	1085	44.4%
Prefers detoxification without MOUD afterwards	280	11.5%
Previous side effects from XR-NTX	10	0.4%
Previous attempt to XR-NTX failed to maintain abstinence	13	0.5%
Does not like injections	24	1.0%
There is a concern about finding a provider for vivitrol injections after the study	4	0.2%
There is a concern about paying for the shots after the study	2	0.1%
There is a concern about their insurance coverage for the inpatient induction	4	0.2%
Other	196	8.0%

Percentages are calculated with a denominator of the number of pre-screen participants who did not attempt XR-NTX induction (N=2443) and sum to greater than 100% because multiple reasons may be selected.

© 2024 Shulman M et al. *JAMA Network Open.*

Supplemental Resource 1: Rapid Procedure Example Order Set

Rapid Procedure Example Order Set

RAPID PROCEDURE				
STEP 1	STEP 2	STEP 3	STEP 4	STEP 5
BUPRENORPHINE INDUCTION	WASHOUT	LOW-DOSE NALTREXONE (0.5mg/1mg)	LOW-DOSE NALTREXONE (3mg/3mg)	XR-NTX (Vivitrol®) (PO NTX 6mg/ XR-NTX 380mg IM)

STANDING MEDICATIONS/ORDERS *(throughout all steps in Rapid Procedure)*:

- clonidine 0.2mg q4h, hold for sBP<90 or HR<60 or HR>120 and notify prescriber *(may reduce to 0.1mg q4h)*
- clonazepam 1mg po q4h (MDD=4mg), hold for oversedation
- prochlorperazine 10mg q24h for nausea and vomiting
- trazodone 50-100mg po qhs for sleep aid
- if tobacco use--nicotine patch (7mg/14mg/21mg) topically daily
- oral electrolyte solution (Gatorade) 8 ounces po q4h

PRN MEDICATIONS:

- prochlorperazine 10 mg q8h PRN for nausea/vomiting
- aluminum hydroxide/magnesium hydroxide (Maalox) 30 ml q4h PRN for dyspepsia
- loperamide 4mg po q24h PRN for diarrhea
- PRN sleep aid (e.g., Zolpidem 5-10mg, Benadryl 50mg, or mirtazapine 7.5mg)
- ibuprofen 600 mg q8h PRN for muscle aches and/or pain (maximum daily dosing=3200mg)
- acetaminophen 650mg q4h PRN for HA or mild pain (maximum daily dosing=4gm)
- PRN nicotine gum/lozenges

SWIFT PROTOCOL STEPS (each step ~24h) *(to be reviewed and ordered by prescribing clinician)*:

STEP 1: buprenorphine induction

- Confirm active orders for standing medications as listed above (i.e., clonidine, clonazepam,…)
- buprenorphine 2mg x1 if **COWS ≥8**
- PRN buprenorphine 2mg q1h if **COWS ≥ 7**(optimal total daily dose 6-8mg; do not exceed 10mg for SWIFT protocol)

STEP 2: washout (no opioid agonists given for 24h)

- confirm active orders for standing medications as listed above and that patient is hydrated and taking these medications

STEP 3: low-dose naltrexone titration

- Confirm standing adjunctive medications as listed above
- 1-2 hours after adjunctive meds -- **naltrexone 0.5mg po** x1 if **COWS ≤ 5**

IF COWS > 5, notify prescriber and can reassess later

- 3-4 hours after 1st naltrexone dose -- **naltrexone 1mg** po x1 **if COWS does not increase 3+ from last assessment**
  IF COWS increases by 3 or more, *then do not give the second naltrexone dose and start with naltrexone 1mg tomorrow (instead of 3mg) and the second dose is still naltrexone 3mg*

STEP 4: low-dose naltrexone titration**

- Confirm standing adjunctive medications as listed above
- 1-2 hours after adjunctive meds -- **naltrexone 3mg po**x1 if **COWS ≤ 5 OR naltrexone 1mg** if patient only received naltrexone 0.5mg yesterday
- 3-4 hours after 1st naltrexone dose -- **naltrexone 3mgpo** x1 **if COWS does not increase 3+ from last assessment**
  IF COWS increases by 3 or more, *then do not give the second naltrexone dose and goto the following day and start with naltrexone 6mg*

IF patient is requesting to leave this day, then consider administration of Vivitrol 380mg IM if both naltrexone 3mg doses tolerated (COWS ≤ 5)**.

STEP 5: low-dose naltrexone titration and Vivitrol administration

- Confirm standing adjunctive medications as listed above
- 1-2hours after adjunctive meds -- **naltrexone 6mg po** x1 if **COWS ≤ 5**
- 3-4 hours after 1st naltrexone dose -- **Vivitrol 380mg IM**x1 **if COWS does not increase 3+ from last assessment**

EXAMPLE DOSING SCHEDULE ON NALTREXONE DAY:

9AM: clonidine, clonazepam, prochlorperazine, PO hydration

10AM: COWS assessment, 1st naltrexone dose

1PM: clonidine and clonazepam, PO hydration (other PRN adjunctive meds)

2PM: COWS assessment, 2nd naltrexone dose

5PM: clonidine and clonazepam, PO hydration (other PRN adjunctive meds)

9PM: clonidine and clonazepam, PO hydration (other PRN adjunctive meds)

VITALS: q4h with clonidine dosing

Gatorade: q4h

ADJUNCTIVE MEDICATIONS ON DISCHARGE:

- Clonidine and clonazepam (tapering doses), see example taper plans below (tapers may need to be adjusted if concurrent alcohol or sedative dependence)
- PRN prochlorperazine 10mg q24h x7 days
- PRN trazodone 50-100mg OR zolpidem 5-10mg in the evening once daily x7 days
- Nicotine replacement therapy if tobacco use and interested in on dc

EXAMPLE TAPER PLANS for clonidine and clonazepam on discharge:

© 2024 Shulman M et al. *JAMA Network Open.*

[]Clonidine Example)

- *clonidine 0.1 mg 4 times a day, to be tapered by 0.1 mg every 2 days*
- Days 1 and 2: clonidine 0.1mg 4 times a day
- Days 3 and 4: clonidine 0.1mg 3 times a day
- Days 5 and 6: clonidine 0.1mg 2 times a day
- Day 7: clonidine 0.1mg once and then discontinue

[]Clonazepam Example)

- *clonazepam 0.5 mg two times a day, to be tapered by 0.25 mg (half a tablet) per day every 2 days*
- Days 1 and 2: clonazepam 1mg (TDD)
- Days 3 and 4: clonazepam 0.75mg
- Days 5 and 6: clonazepam 0.5mg
- Days 7 and 8: clonazepam 0.25mg and then discontinue on Day 8

© 2024 Shulman M et al. *JAMA Network Open.*

Supplemental Resource 2: Shared Decision-Making Tool for Medications for Opioid Use Disorder

© 2024 Shulman M et al. *JAMA Network Open.*

QUIT OPIOIDS

FOR GOOD

WITH MEDICATION

REASONS YOU MIGHT CHOOSE MEDICATION

AND

WHICH MEDICATION IS BETTER FOR YOU?

© 2024 Shulman M et al. *JAMA Network Open.*

Few words about opioid addiction

- Opioid addiction is **a chronic medical condition**, once it develops it remains present whether people are using opioids or not
- Patients with opioid addiction have abnormal function of some brain centers which cause them to have strong, and difficult to resist urges to use opioid drugs
- In people with opioid addiction, stopping opioid use is a first step to improve quality of their life, **BUT**, most people are unable to resist using opioids **without the help of a medication**
- Many who go to the hospital to detoxify off opioids and return home **without medication relapse within few weeks, and some die of overdose**
- There are three medications to help with craving and the urge to use to prevent relapse:

methadone	buprenorphine (Suboxone)	naltrexone (Vivitrol)

Each of these medications have a different mechanism of action

Your chance of having good medication response is best if you take the medication that fits your personal needs, preferences, and treatment goals

- The doctor is here to help you make a choice about your path to the recovery

Addiction is difficult to cure, but it is a disorder that can be managed, like diabetes or hypertension, with medications and lifestyle changes

© 2024 Shulman M et al. *JAMA Network Open.*

Good things about taking medications

- People relapse because they cannot stop thinking about the drug. With the right medication, craving and intrusive thoughts go away and decisions to abstain from the drug are easier to make.
- Medication reduces rate of overdose by half for those who stay on medication
- Medication can be used together with therapy or can be used without it, both strategies are effective
- Medication can help with the recovery effort, they are bridge to long term recovery
- You may not need to remain on medication once you are stable in your recovery. Working with your doctor you can re-evaluate if it is safe to come off the medication.

Bad things about taking medications

- It can take time to get used to the medication
- Medication can cause side-effects
- Medications and doctor's visits can be expensive, though some insurance may help to cover it
- Medications can be a reminder of having a problem with drugs and people may be judgmental about you taking them.

If you are not sure about being on a medication, you can always try it for 1-2 months to see if it is helpful for you

© 2024 Shulman M et al. *JAMA Network Open.*

What medications can I take?

METHADONE

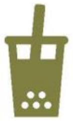

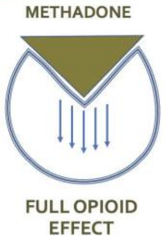

- A "gold standard" in the treatment of opioid addiction, it is considered to be the most effective medication
- Methadone strongly activates opioid receptors in the brain (similarly to heroin), and eliminates withdrawal, reduces craving, and blocks the high from heroin/painkillers
- Patients taking methadone either stop using heroin or other opioids, or use much less, have fewer medical problems, and have better social and work functioning

METHADONE DOWNSIDES

- Methadone can only be given in a specialized methadone clinic, where you need to go every day at the beginning of treatment to receive the dose and be observed
- Needs to be taken every day and if you miss the dose you may go into withdrawal
- Methadone is a potent medication and can cause sedation and overdose if not taken properly and if mixed with alcohol or sedatives
- Methadone may produce side-effects like nausea, sweating, constipation, sexual problems, and heart problems

© 2024 Shulman M et al. *JAMA Network Open.*

BUPRENORPHINE
(Suboxone, Zubsolv)

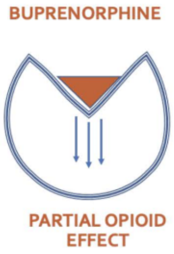

- Works similarly to methadone, eliminates withdrawal and drug craving, but it only partially activates opioid receptors and therefore is safer and has fewer side effects
- Buprenorphine is safer than methadone, and therefore it can be prescribed by a doctor or a nurse and taken at home
- Both methadone and buprenorphine will protect you from the overdose, but only if you keep taking the medication every day
- Both methadone and buprenorphine cannot be stopped abruptly, or you will experience withdrawal symptoms

BUPRENORPHINE DOWNSIDES

- Need to be in withdrawal before taking the first dose
- Occasional side-effects such as nausea, constipation, headache, and drowsiness
- Overdose can occur if buprenorphine is mixed with large amount of sedating medications or alcohol

© 2024 Shulman M et al. *JAMA Network Open.*

NALTREXONE
(Vivitrol)

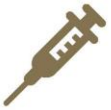

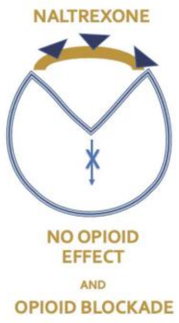

- Naltrexone works differently from methadone or buprenorphine. It completely blocks opioid receptors
- It can be used only after completing detoxification
- Naltrexone can decrease your craving for heroin to prevent relapse
- If you use heroin while on naltrexone, it will block heroin effects and will prevent craving for more drugs
- It has no abuse potential, no overdose risk, and there is no withdrawal when it is stopped
- Is available as a monthly injection give into the buttock

NALTREXONE DOWNSIDES

- You can only receive the first naltrexone injection after you have been fully withdrawn from the opioids (aka detoxified), which can be difficult because of withdrawal symptoms
- It usually takes a week or more from your last dose of heroin to be ready for naltrexone injection, which can be difficult because you may continue having urges to use while waiting
- When you take naltrexone, opioid painkillers will not control your pain, you would need to use different medication/procedures
- Injection of naltrexone can be painful and irritate injection site and it can cause liver problems
- It may take few weeks for people to feel completely well after receiving first injection of naltrexone

© 2024 Shulman M et al. *JAMA Network Open.*

MYTHS about medications for opioid addiction

There are no good medication treatments and in order to get better you have to have the willpower to want to stop, go to a residential rehabilitation program, and go to meetings afterwards

TRUTHS

- Medications are very effective, for many they are more effective and less expensive than going to a residential program
- The risk of relapse to opioids is high among people discharged from a residential program without medication; the risk of overdose is also increased
- The risk of relapse and overdose can be reduced by starting medication before leaving the residential program

Medication like buprenorphine replaces one drug with another and keeps you from reaching true recovery

TRUTHS

- Buprenorphine and a steady dose of methadone does not produce high, which is what perpetuates craving and makes the heroin addictive, on buprenorphine or methadone you feel "normal" all the time
- Buprenorphine replaces some of the effects of heroin which is why it takes away craving and withdrawal
- It is much easier to work towards the recovery if you are on the medication and you do not have to cope with incessant craving

You only need the buprenorphine for a short time, if you take it for too long you will get addicted

TRUTHS

- The longer you stay on buprenorphine the more it can help to improve the quality of your life and protect you against the relapse and overdose
- Over time, most people are taking lower doses and have no side-effects

When you feel well for few months it means it is time to stop the medication

TRUTHS

- This is a common misconception - feeling well means that the medication is a good choice for you and you should continue taking it
- Stopping the medication too soon puts you are a very high risk of relapse
- The most important things to consider in considering stopping medication is how much your life has changed since you stopped using illicit opioids.

© 2024 Shulman M et al. *JAMA Network Open.*

TREATMENT PLAN

You and your doctor have discussed ALL medication options

methadone

buprenorphine (Suboxone)

naltrexone (Vivitrol)

and made a decision today about the BEST medication to help you overcome your addiction to opioids

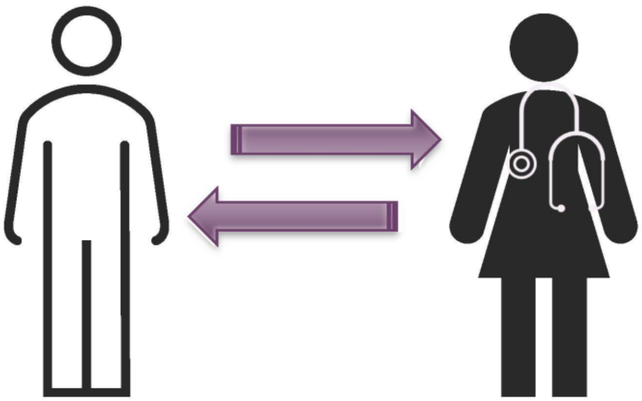

MEDICATION NAME

____

© 2024 Shulman M et al. *JAMA Network Open.*
