# Supplementary material for: Rapid Initiation of Injection Naltrexone for Opioid Use Disorder: A Stepped-Wedge Cluster Randomized Clinical Trial
Source: JAMA Netw Open. 2024 May 8;7(5):e249744. doi: 10.1001/jamanetworkopen.2024.9744 (PMC11079685; doi:10.1001/jamanetworkopen.2024.9744)
Supplement: Supplement 3. — Statistical Analysis Plan [file jamanetwopen-e249744-s003.pdf]

Statistical Analysis Plan for NIDA Protocol CTN-0097

Surmounting Withdrawal to Initiate Fast Treatment with Naltrexone: Improving the Real-World Effectiveness of Injection Naltrexone for Opioid Use Disorder (SWIFT)

Lead Investigator: Adam Bisaga, MD

Co-Lead Investigator(s): Edward V. Nunes, MD, John Rotrosen, MD

Version 2.0

April 13, 2023

Prepared by:

NIDA CTN Data and Statistics Center

SIGNATURE PAGE

Lead Investigator: Adam Bisaga, MD

Signature: Adam Bisaga I am approving this document. Date: 17/Apr/2023 03:30 PM EDT

Adam Bisaga

Co-Lead Investigator: Edward V. Nunes, MD

Signature: Edward Nunes I am approving this document. Date: 24/Oct/2023 03:52 PM EDT

Edward Nunes

CCTN Scientific Officer: Udi Ghitza, PhD

Signature: Udi Ghitza I am approving this document. Date: 24/Oct/2023 01:44 PM EDT

Udi Ghitza

DSC Statistics Leadership: Aimee Wahle, MS

Signature: Aimee Wahle I am approving this document. Date: 24/Oct/2023 02:08 PM EDT

DSC Leadership: Kathryn Hefner, PhD

Signature: Kathryn Hefner I am approving this document. Date: 24/Oct/2023 03:17 PM EDT

Kathryn Hefner

TABLE OF CONTENTS

LIST OF ABBREVIATIONS .... **v**
LIST OF eCRFs .... **vii**
1.0 INTRODUCTION **1**
2.0 SUMMARY OF STUDY DESIGN AND PROCEDURES **1**
2.1 Study Objectives 1
2.1.1 Primary Objective 1
2.1.2 Secondary Objectives 2
2.1.3 Exploratory Objectives 2
2.1.4 Implementation Objectives 3
2.2 Study Design and Procedures 3
2.2.1 Study Design 3
2.2.2 Study Assessments 4
2.2.3 Study Procedures 5
2.2.4 Randomization 7
2.2.5 Blinding 7
2.2.6 Eligibility Criteria for Selection of Study Population 7
2.3 Participant Inclusion Criteria 7
2.4 Participant Exclusion Criteria 8
3.0 GENERAL ANALYSIS POPULATIONS, DEFINITIONS, AND CONVENTIONS **8**
3.1 Analysis Populations 8
3.1.1 Pre-screened Population 8
3.1.2 Screened Population 9
3.1.3 Intent-to-Treat Population 9
3.1.4 Safety Population 9
3.1.5 Inducted Population 9
3.1.6 Provider/Staff Population 9
3.2 General Definitions 9
3.2.1 Induction Phase 9
3.2.2 Post-induction Phase 9
3.2.3 Baseline Visit 9
3.2.4 Baseline Value 10
3.2.5 Safety Window 10
3.2.6 Targeted Safety Event 10
3.2.7 Serious Adverse Event 10
3.3 Table, Figures and Listings Conventions 11
4.0 PARTICIPANT ENROLLMENT, DISPOSITION, AND VISIT ATTENDANCE **11**
4.1 Participant Enrollment 11
4.2 Participant Disposition 11
4.3 Visit Attendance at Post-induction Visits 11

5.0 ANALYSIS OF PARTICIPANT BASELINE CHARACTERISTICS ... 12
6.0 MEDICATIONS ADMINISTERED DURING INDUCTION PHASE ... 12
7.0 TREATMENT EXPOSURE ... 12
8.0 EFFECTIVENESS ANALYSIS ... 13
8.1 Definition of the Primary Outcome Measure ... 13
8.2 Analysis of the Primary Outcome Measure ... 13
8.3 Supportive Analyses of the Primary Outcome Measure ... 14
8.4 Definition of the Secondary Outcome Measures ... 15
8.5 Analyses of the Key Secondary Outcome Measures ... 17
8.6 Definition of the Exploratory Outcome Measures ... 19
8.7 Missing Data Analysis ... 19
9.0 IMPLEMENTATION OUTCOME ANALYSIS ... 19
10.0 SAFETY OUTCOMES AND ANALYSIS ... 20
10.1 Targeted Safety Events ... 20
10.2 Serious Adverse Events ... 20
10.3 Death ... 20
10.4 Injection Site Abnormalities ... 20
10.5 Overdoses ... 21
10.6 Suicide Risk ... 21
10.7 Pregnancy ... 21
11.0 SIGNIFICANCE TESTING AND MULTIPLICITY ... 21
12.0 SAMPLE SIZE AND POWER ... 21
13.0 INTERIM ANALYSES AND DATA MONITORING ... 28
13.1 Safety Interim Analyses ... 28
14.0 DATA QUALITY ... 28
14.1 Data Audits ... 28
14.2 Protocol Deviations ... 28
15.0 SOFTWARE TO BE USED FOR ANALYSES ... 28
16.0 UPDATES TO THE STATISTICAL ANALYSIS PLAN ... 28
17.0 REFERENCES ... 30
18.0 LIST OF PROPOSED TABLES, FIGURES, AND LISTINGS ... 31
19.0 APPENDICES ... 36
19.1 SHELLS FOR PROPOSED TABLES, FIGURES AND LISTINGS ... 36
19.1.1 Enrollment, Participant Disposition, and Visit Attendance ... 36
19.1.2 Participant Characteristics at Baseline ... 55
19.1.3 Medications Administered During Induction Phase ... 96
19.1.4 Early Induction Termination ... 119
19.1.5 Treatment Exposure ... 121
19.1.6 Primary Outcome Analyses ... 124
19.1.7 Supportive Analyses of Primary Outcome ... 126
19.1.8 Key Secondary Outcome Analyses ... 131

19.1.9 Safety 162
19.1.10 Data Quality 183

LIST OF ABBREVIATIONS

Abbreviation	Definition
AE	Adverse Event
BUP	Buprenorphine
CCC	Clinical Coordinating Center
CCTN	Center for the Clinical Trials Network
CFIR	Consolidated Framework for Implementation
COWS	Clinical Opiate Withdrawal Scale
CRF	Case Report Form
CTN	Clinical Trials Network
DSC	Data and Statistics Center
DSM-5	Diagnostic and Statistical Manual of Mental Disorders Fifth Edition
DSMB	Data and Safety Monitoring Board
eCRF	Electronic Case Report Form
EDC	Electronic Data Capture
EMR	Electronic Medical Record
EOS	End of Study
EMT	End of Medication Treatment
GAD-7	Generalized Anxiety Disorder Screener
HIV	Human Immunodeficiency Virus
ICC	Intraclass Correlation Coefficient
IF	Implementation Facilitation
ITT	Intent-to-Treat
IV	Intravenous
LI	Lead Investigator
MedDRA	The Medical Dictionary for Regulatory Activities
Mg	Milligrams
mL	Milliliters
MOUD	Medications for Opioid Use Disorder
NIDA	National Institute on Drug Abuse
NIH	National Institutes of Health
NTX	Naltrexone
NX	Naloxone
ORCA	Organizational Readiness to Change Assessment
OUD	Opioid Use Disorder
PHQ-9	Patient Health Questionnaire-9
PI	Principal Investigator
RP	Rapid Procedure
SAE	Serious Adverse Event

Abbreviation	Definition
SOWS	Subjective Opiate Withdrawal Scale
SP	Standard Procedure
SW	Stepped-Wedge
TAU	Treatment as Usual
TLFB	Timeline Followback
TSE	Targeted Safety Event
UDS	Urine Drug Screen
VAS	Visual Analog Scale
XR-NTX	Extended-Release Naltrexone (Vivitrol®)

LIST OF eCRFs

AAS	Adult ADHD Self-Report Screening Scale for DSM-5
AD1	Adverse Event
AD2	Serious Adverse Event Summary
AD3	Serious Adverse Event Medical
ASU	Alcohol and Substance Use
C97	Critical Action Checklist
CMX	Concomitant Medications
COW	Clinical Opioid Withdrawal Scale
CUT	Clinic Urine Toxicology
CVD	COVID-19 Impact Assessment
D97	Study Demographics (RA/RC Administered)
DEM	Demographics
DMA	Daily Medication Administration Log
DSM	DSM-5 Checklist
DTH	Death Form
EIP	End of Induction Survey
ENRA	Enrollment into Segment A
ENRB	Enrollment into Segment B
ENRC	Enrollment into Segment C
ENRD	Enrollment into Segment D
ENRE	Enrollment into Segment E
EO2	End of Medication (Self-Reported)
EOI	End of Induction
EOM	End of Medication
FAM	Family Origin
FC1	Pre-Implementation Fidelity to Implementation Checklist
FC2	Post-Implementation Fidelity to Implementation Checklist
FND	Fagerström Test for Nicotine Dependence
FOO	Fatal Opioid Overdose
GA7	Generalized Anxiety Disorder
GEN	Genetics
HEP	Self-Report of Hepatitis Testing and Treatment
HIV	Self-Report of HIV Testing
INA	Injection Site Abnormality
INB	Induction Buprenorphine
INN	XR-NTX Administration
LDN	Low Dose Naltrexone Titration
LIF	Locator Information Form

M97	Timeline Followback Medications
MGT	Medical Management
MHA	Mental Health Follow-up Assessment
MHX	Medical Psychiatric History
MJA	Cannabis Use Assessment
MTV	Motivation Scale
NM2	Non-Medical and Other Services - Page 2
NMS	Non-Medical and Other Services
OCI	Opioid Craving Scale
OCO	Opioid Craving Scale
ODQ	Overdose Questionnaire
OES	Opioid Effect Scale
OLC	Organization Level Clinical Implementation
OR1	Pre-Implementation Organizational Readiness to Change Assessment
OR2	Post-Implementation Organizational Readiness to Change Assessment
PBC	Pregnancy and Birth Control Assessment
PCL	PTSD Checklist for DSM-5
PDA	Panic Disorder Assessment
PHQ	Patient Health Questionnaire (PHQ-9)
PO1	Pregnancy Outcome 1
PO2	Pregnancy Outcome 2
PO3	Pregnancy Outcome 3
PO4	Pregnancy Outcome 4
PRG	Confirmed Pregnancy and Outcome
PRO	PROMIS
PSA	Prisoner Status Assessment
PST	Psychosocial Treatment
QLP	Quality of Life
S97	Additional Demographics
SBW	The Subjective Opiate Withdrawal Scale
STC	Study Completion
T97	Timeline Followback
TAP	TLFB Assessment Period
TS5	Treatment Satisfaction Survey
TSE	Targeted Safety Event
TUH	Tobacco Use History
UDS	Urine Drug Screen
V97	Missed Visit and Visit Documentation

1.0 INTRODUCTION

The Statistical Analysis Plan (SAP) for CTN-0097 Surmounting Withdrawal to Initiate Fast Treatment with Naltrexone: Improving the Real-World Effectiveness of Injection Naltrexone for Opioid Use Disorder (SWIFT) expands upon the statistical information presented in the protocol and describes all planned analyses for the primary, secondary, exploratory, implementation, and safety outcome measures occurring after data lock. The Clinical Trial Network (CTN)'s Data and Statistics Center (DSC) will conduct the analyses for the Final Study Report (FSR) as listed in Table 1 below and the Lead Node (LN) will conduct the analyses as noted.

Table 1: Analysis Responsibilities

Content	Section Number	Responsible for Analysis
Participant Enrollment, Disposition, and Follow-up	4.0	DSC
Participant Baseline Characteristics	5.0	DSC
Medications Administered During Induction Phase	6.0	DSC
Analyses of Treatment Exposure	7.0	DSC
Analyses of Primary Outcome	8.2	DSC
Supportive Analyses of Primary Outcome[1]	8.3	DSC/LN
Analyses of the Key Secondary Outcome Measures	8.5	DSC
Analyses of the Other Secondary Outcome Measures	8.5	LN
Analyses of the Exploratory Outcome Measures	8.6	LN
Analyses of the Implementation Outcomes	9.0	LN
Safety Outcomes	10.0	DSC
Data Quality	14.0	DSC

[1] Details will be provided in Section 8.3.

2.0 SUMMARY OF STUDY DESIGN AND PROCEDURES

2.1 Study Objectives

2.1.1 Primary Objective

The primary goal of the study is to determine whether the Rapid Procedure (RP) method of initiating treatment with XR-NTX is non-inferior to the Standard Procedure (SP) method recommended in the XR-NTX Prescribing Information. The primary outcome measure is a dichotomous measure of treatment initiation success defined as receipt of the first injection of XR-NTX while inpatient on a detoxification or residential rehabilitation unit. The hypothesis is that the RP will be non-inferior to SP in terms of proportion of successful initiations of XR-NTX.

2.1.2 Secondary Objectives

The key secondary objective is to summarize the below outcome to confirm expected characteristics of the RP compared to SP:

1. Time to receipt of first injection of XR-NTX from day of admission for participants that receive first injection of XR-NTX while on the unit.

Additional key secondary objectives are to compare the below outcomes of RP versus SP for all enrolled participants, intent-to-treat (ITT) population.

2. Craving for opioids measured by Visual Analog Scales (VAS).
3. Opioid withdrawal symptoms as measured by the Subjective Opioid Withdrawal Scale (SOWS) and the Clinical Opiate Withdrawal Scale (COWS).
4. Safety, as measured by overdose questionnaire, targeted safety events and serious adverse events.

Other secondary objectives are to compare the below outcomes of RP vs. SP for participants who receive the first injection while on the unit.

5. Retention in the trial to receive the second and the third XR-NTX injections.
6. Craving for opioids measured by Visual Analog Scales.
7. Opioid withdrawal symptoms following XR-NTX injection as measured by the Subjective Opioid Withdrawal Scale.
8. Safety, as measured by overdose questionnaire, targeted safety events and serious adverse events.
9. Opioid abstinence, as measured by the Timeline Followback (TLFB) (self-report days using opioids) and proportion of opioid-positive urine tests (UDS).

2.1.3 Exploratory Objectives

Exploratory objectives include:

1. Explore baseline demographic and clinical features (e.g., the primary opioid of dependence (heroin/fentanyl vs. prescription opioid)) as: a) predictors of induction success, secondary outcomes and retention during the trial (main effect of predictors), and b) as moderators of differential treatment effect (moderator by treatment interaction).
2. Compare duration of treatment on the unit and the associated costs from the time when detoxification is initiated to the time that XR-NTX is administered to permit analyses of economic costs and benefits of the two induction procedures.
3. Compare RP versus SP for all enrolled participants in terms of time from day of admission to XR-NTX initiation failure (day of discontinuation of detoxification period that resulted in failure to receive first XR-NTX) and reasons for failure.
4. Compare RP versus SP for all enrolled participants in terms of other depressive, anxiety, and subacute withdrawal symptoms as measured by the Patient Health Questionnaire-9 (PHQ-9) and General Anxiety Disorder-7 (GAD-7).
5. Compare RP versus SP use of alcohol and other drugs of abuse (e.g., cocaine, other stimulants, cannabis, benzodiazepines), by self-report and urine drug screens for all enrolled participants.

6. Explore engagement with medical visits and therapy (based on Medical Management Log, Psychosocial Log, XR-NTX Administration Form, TLFB).
7. Compare RP versus SP for the percentage of patients inducted onto any MOUD (XR-NTX, buprenorphine, or methadone, as measured by TLFB for MOUD), both before discharge from inpatient unit and after discharge from inpatient unit).
8. Investigate the percentage of induction failure participants that receive XR-NTX during the course of the study overall and for each induction procedure as measured by patient self-report on TLFB).
9. Compare RP versus SP for percentage of participants inducted on XR-NTX (during both induction and post-induction phases of the trial).

2.1.4 Implementation Objectives

The objective of the implementation component of the study is to understand facilitators and barriers to implementation of Rapid initiation of XR-NTX and to iteratively develop an implementation facilitation manual which can be used to disseminate XR-NTX initiation methods across the treatment system. Measurement will be grounded in the CFIR (Damschroder and Hagedorn, 2011) and will assess intervention characteristics (provider survey), inner setting factors (e.g., readiness, clinical leadership structure, resources; survey and environmental scan), individual provider characteristics (provider survey), and implementation process through fidelity measures. Following each stepped wedge, this information will be integrated into the IF manual (by adding new information and/or making modifications to existing information) to improve the procedures. The ultimate goal will be to produce a high quality IF manual at the conclusion of trial which could facilitate widespread implementation of XR-NTX induction across community-based treatment settings, as well as be tested within its own right in a larger implementation-focused trial.

2.2 Study Design and Procedures

2.2.1 Study Design

This is a six-center, stepped-wedge, cluster randomized trial comparing effectiveness and safety of Rapid (5-7 days) versus Standard (13 days) XR-NTX induction procedure (RP vs. SP). The study will proceed in five steps, with each step lasting 14 weeks, for the total of 70 weeks of the study (Table 2). A total of 450 participants (15 per site per step) eligible for and seeking treatment for opioid disorder with XR-NTX and consenting to research assessments during their course of XR-NTX treatment are planned to be enrolled in the study. Enrolled participants will receive the naltrexone initiation regimen being offered at the site at the time of admission, either SP or RP depending on the random assignment as per study design. At the beginning of each step a randomly selected site will begin implementing RP and will continue to offer it for the remainder of the trial, according to the optimized stepped-wedge study design (Thompson et al., 2017). Note the first randomized site will offer only RP whereas the last remining site will offer only SP for the entire duration of the trial. After sites transition to RP, this will be the only regimen available for patients interested in participating in the research study (i.e., SP will no longer be offered as a part of the study). Before each stepped wedge of the study, an 8-week pre-implementation or preparation phase will occur for each site randomized to RP. During the implementation phase of RP, the implementation team will meet routinely to review the implementation process and identify modifiable barriers or facilitators to facilitate RP implementation. After each stepped wedge, the study team with expertise in implementation will meet to review identified barriers and facilitators to implementing the RP intervention and modify or adapt the RP Implement Facilitation package accordingly. Site level and provider/staff level surveys will be completed before and after implementation of RP.

Table 2: Schematic of study design with six study sites and five steps, each lasting 14 weeks

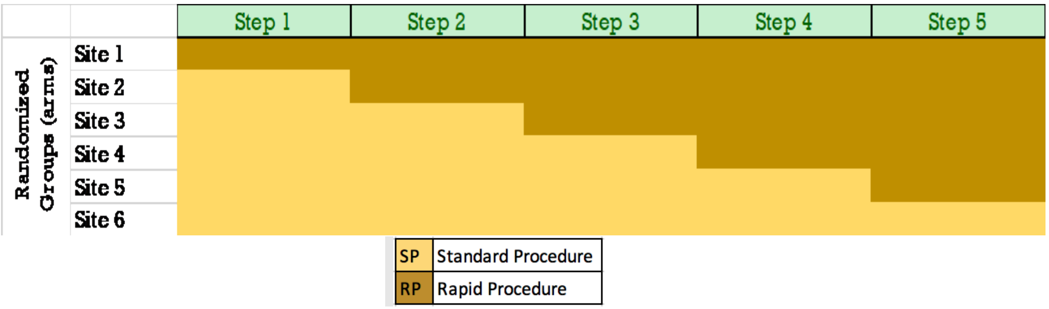

2.2.2 Study Assessments

All assessments and corresponding case report forms by study phase and study visit are described below.

During pre-screening (Day -7 up to Day 3 of admission), potential participants will be assessed for basic eligibility to move forward in the study. Patients that meet pre-screening basic eligibility criteria will be asked to sign the informed consent (IC) no later than Day 4 of admission. Patients that sign the IC enter the screening phase and are assessed for eligibility to enroll in the study no later than Day 4 of admission. The following assessments are performed during screening: Demographics (DEM), Prisoner Status Assessment (PSA), Locator Information Form (LIF), Concomitant Medications (CMX), Medical Psychiatric History (MHX), DSM-5 Checklist (DSM), Pregnancy and Birth Control Assessment (PBC), and Urine Drug Screen (UDS).

Following the final confirmation of eligibility for study enrollment participants complete baseline assessments during Days 1-4: Study Demographics (D97), Genetics (GEN), Family Origin (FAM), Alcohol and Substance use (ASU), Non-Medical and other Services (NMS/NM2), Timeline Follow-back – Substance and MOUD (TAP/T97/M97), Concomitant Medications (CMX), Pregnancy and Birth Control Assessment (PBC), Urine Drug Screen (UDS), Daily Medication Administration Log (DMA), Clinical Opiate Withdrawal Scale (COW), and Medical Management (MGT). In addition, the following self-reported baseline assessments will be collected from participants: Additional Demographics (S97), Opioid Craving Scale Inpatient (OCI), Overdose Questionnaire (ODQ), The Subjective Opioid Withdrawal Scale (SBW), Cannabis Use Assessment (MJA), Tobacco Use History (TUH), Fagerström Test for Nicotine Dependence (FND), Motivation Scale (MTV), Quality of Life (QLP), PROMIS (PRO), Panic Disorder Assessment (PDA), Generalized Anxiety Disorder (GA7), PTSD Checklist for DSM-5 (PCL), Patient Health Questionnaire-9 (PHQ), Mental Health Assessment (MHA), Adult ADHD Self-Report Screening Scale for DSM-5 (AAS), Self-Report of Hepatitis Testing and Treatment (HEP), Self-Report of HIV Testing (HIV) and COVID-19 Impact Assessment (CVD).

Induction Phase Assessments (Day 1 – Day 30)

The induction phase for enrolled participants (within both RP and SP) begins with day of admission (induction phase Day 1) and ends on the day of XR-NTX receipt or day of failure of the induction protocol. The maximum length of the induction phase is 30 days. For participants who leave the unit prior to Day 30, the date of leaving the unit without a medication would be equivalent to date of failure as recorded on the EOI form per the CRF manual. Daily and weekly assessments will be conducted during the induction phase as follows:

- Daily assessments (DMA, Low Dose Naltrexone Titration [LDN] for the Rapid Protocol only, COW, OCI, SBW, Psychosocial and Medical Treatment Participation log [PST]).

- Weekly assessments (GA7, PHQ).
- Assessments collected prior to first XR-NTX injection include UDS (all participants) and PBC (females only).
- Data will be collected retrospectively starting with induction phase Day 1, using medication flowsheets and EMR abstraction (performed and confirmed by study staff). The following assessments may be retrospectively collected/abstracted: DMA, COW, UDS and PBC.
- End of Induction (EOI) and End of Induction Survey (EIP) are collected at the end of the induction phase regardless of whether the participant receives their first dose of XR-NTX on the unit, if the participant terminates the induction protocol early, or if they exceed the maximum window allowed for the induction phase.

Post-Induction Phase Assessments (Week 1 – Week 8)

The post-induction phase will begin the day after receiving XR-NTX injection or the day after failure of the induction protocol (Day 1 post-induction). During the 8-week post-induction phase, participants will receive medication management visits per the site's standard clinical practice, plus an additional two XR-NTX injections, expected at four and eight weeks after the first XR-NTX injection, along with other psychosocial treatment as per the standard of care provided by each site. The following assessments will be conducted during post-induction phase at Weeks 1, 2, 3, 4, 6 and 8: Missed Visit and Visit Documentation (V97), TAP/T97/M97, MGT, OCO, ODQ, SBW, OES, GA7, PHQ, PST. UDS (all participants) and PBC (females only) will be assessed prior to each XR-NTX injection. Additional assessments will be performed at week 3 (PSA) and week 4 and 8 (INN, NMS/NM2).

Note that participants who fail the induction phase will still be enrolled into the post-induction phase but only assessed at Weeks 4 and 8. Participants who discontinue XR-NTX early (i.e.do not receive both injections) will complete the Early End of Medication Visit and the following assessments will be performed: V97, CMX, MGT, End of Medication (EOM), and End of Medication – Self-Reported (EO2).

Other assessments will be performed as needed: Injection Site Abnormality (INA), Mental Health Follow-Up Assessment (MHA), Study Completion (STC).

In addition to the above assessments, the study will collect data on implementation facilitation outcomes: Organization Level Clinical Implementation (OLC) at the start of each step at all sites, Pre/Post Organizational Readiness to Change Assessment (ORCA) (OR1/OR2) prior to and at the end of RP implementation step at the randomized site, Pre/Post Fidelity to Implementation Checklist (FC1/FC2) at the start and end of RP implementation step at the randomized site. OR1/OR2 collect provider/staff level data whereas OLC and FC1/FC2 collect site level data.

2.2.3 Study Procedures

Participants are inducted onto their assigned XR-NTX induction regimen: SP (Table 3) or RP (Table 4). These regimens are guidelines to be applied by the clinical teams with coaching from the lead team and may be modified by the site clinician based on the clinical status of each patient.

Table 3: Standard Induction Procedure	
**Day 1**	All participants will receive buprenorphine 6 mg. If participants continue to experience significant withdrawal (COWS > 6) they can receive additional 2-4 mg.
**Day 2**	Buprenorphine 6 mg in AM
**Day 3**	Buprenorphine 4 mg in AM
**Day 4**	Buprenorphine 2 mg in AM
**Day 5**	Buprenorphine 1 mg in AM
**Days 6-13**	Adjunctive medications as needed (i.e., clonidine and clonazepam)
**Day 13 or 14**	XR-NTX eligibility determination followed by the XR-NTX injection

Table 4: Rapid Induction Procedure	
**Day 1**	All participants will receive buprenorphine 6 mg. If participants continue to experience significant withdrawal (COWS > 6) they can receive additional 2-4 mg. Adjunctive medications will be initiated, if necessary, to alleviate residual withdrawal persisting after administration of buprenorphine 10 mg.
**Day 2**	Standing doses of clonidine and clonazepam, with additional adjunctive medications as needed*.
**Day 3**	Standing doses of clonidine and clonazepam, with additional adjunctive medications as needed*.<br>Following the first morning dose of adjunctive medication, participants will receive naltrexone 0.5 mg. If there is no significant increase in withdrawal over the subsequent 120 minutes (COWS increase < 3) they will receive additional naltrexone 0.5 mg.
**Day 4**	Standing doses of clonidine and clonazepam, with additional adjunctive medications as needed*.<br>Following the first morning dose of adjunctive medication naltrexone 1 mg. If there is no significant increase in withdrawal over the subsequent 120 minutes (COWS increase < 3) they will receive additional naltrexone 1 mg.
**Day 5**	Standing doses of clonidine and clonazepam, with additional adjunctive medications as needed*.<br>Following the first morning dose of adjunctive medication they will receive naltrexone 3 mg. If there is no significant increase in withdrawal over the subsequent 120 minutes (COWS increase < 3) they will receive additional naltrexone 3 mg.
**Day 6**	Standing doses of clonidine and clonazepam, with additional adjunctive medications as needed*.<br>Following the first morning dose of adjunctive medication they will receive naltrexone 6 mg. If there is no significant increase in withdrawal over the subsequent 4 hours (COWS increase < 3) participant will receive an injection of XR-NTX 380 mg IM. For those who were not able to tolerate naltrexone 6mg, please refer to Day 7 procedure.
**Day 7**	Standing doses of clonidine and clonazepam, with additional adjunctive medications as needed*.<br>Participants that were not able to tolerate naltrexone 6 mg during Day 6 will have procedures from Day 6 repeated.

Table 4: Rapid Induction Procedure	
***Adjunctive medication to be administered during days 1-7**	• clonidine 0.2 mg every 4 hours (lower or withhold the dose if SBP< 90 or HR< 50), MDD=1.2 mg/d<br>• clonazepam 1 mg every 6 hours (withhold the dose if the patient is difficult to arouse), MDD=4 mg/d<br>• trazodone 100 mg at night *as needed* for insomnia<br>• prochloperazine 10 mg every 8 hours *as needed* for nausea<br>• zolpidem 10 mg at night *as needed* for insomnia<br>• ibuprofen 600 mg po every 8 hours *as needed* for muscle pain

2.2.4 Randomization

Randomization will be performed at the site (cluster) level. Six sites that have the required characteristics to be included in this study will be randomized to the order in which they begin to implement the RP for XR-NTX induction in place of SP. Patient-participants will be treated with the SP or RP according to which procedure is being used at the site at the time the patient is enrolled. The randomization of each site will follow the stepped-wedge design (Table 2). At the selected time points each 14 weeks apart (week 1 = start of enrollment at all 6 sites, weeks 14, 28, 42, and 56), one randomly selected site will make a “step” and will cross-over from the SP to RP. Note that the first randomized site will be in the RP arm for the entire duration of the study whereas the 6th site (after 5 sites have been randomized to the RP) will be in the SP arm for the entire duration of the study. The randomization procedure will be conducted centrally through the NIDA Data and Statistics Center (DSC). The DSC statistician will conduct the randomization at each step and inform the lead node (LN) team and NIDA representatives via email 8 weeks before the study starts (i.e., all sites are ready to start enrollment) or 8 weeks before each transition to the next step.

2.2.5 Blinding

This is a pragmatic unblinded open-label study and thus there is no blinding to the induction procedure being implemented at each site.

2.2.6 Eligibility Criteria for Selection of Study Population

2.3 Participant Inclusion Criteria

Study participants must meet all of the following inclusion criteria in order to be eligible to participate in the study:

1. 18 years of age or older.
2. Meets DSM-5 criteria for current opioid use disorder.
3. Seeking treatment for opioid use disorder, willing to accept treatment with XR-NTX and, in the judgment of the treating physician, is a good candidate for naltrexone-based treatment.
4. Willing and able to provide written informed consent.
5. Able to speak English sufficiently to understand the induction procedures and provide written informed consent to participate in the study.
6. If female of childbearing potential, willing to practice an effective method of birth control for the duration of participation in the study.

2.4 Participant Exclusion Criteria

Participants meeting any of the following exclusion criteria will be excluded from study participation:

1. Serious medical, psychiatric or substance use disorder that, in the opinion of the study physician, would make a detoxification and naltrexone initiation, or maintenance treatment with XR-NTX, hazardous (relative contra-indications) or requires a different level of care. Examples include:
    a) Disabling or terminal medical illness (e.g., uncompensated heart failure, severe acute hepatitis, cirrhosis or end-stage liver disease) as assessed by medical history and/or review of systems.
    b) Severe, untreated or inadequately treated mental disorder (e.g., active psychosis, uncontrolled manic-depressive illness) as assessed by history and/or clinical interview.
    c) Current severe alcohol, benzodiazepine, or other depressant or sedative hypnotic use likely to require a complicated medical detoxification (routine alcohol and sedative detoxifications may be included).
    d) Suicidal or homicidal ideation that requires immediate attention.
2. Known allergy or sensitivity to buprenorphine, naloxone, naltrexone, polylactide-co-glycolide, carboxymethylcellulose, or other components of the Vivitrol® diluent.
3. Maintenance treatment with methadone within 14 days of consent.
4. Maintenance treatment with buprenorphine unless the patient is determined to have a poor treatment response (in the form of buprenorphine non-adherence with or without the use of illicit opioids), warranting change to XR-NTX treatment.
5. Presence of pain of sufficient severity as to require ongoing pain management with opioids.
6. Circumstances (legal, personal, occupational) that would threaten the feasibility of XR-NTX treatment or make another treatment (e.g., buprenorphine or methadone) a better choice.
7. Are currently in jail, prison or other overnight facility as required by court of law or have pending legal action that could prevent participation in study activities.
8. If female, currently pregnant or breastfeeding, or planning on conception.
9. Body habitus that, in the judgment of the study physician, precludes safe intramuscular injection of XR-NTX (e.g., BMI>40, excess fat tissue over the buttocks, emaciation).
10. Admitted to the inpatient detoxification or residential rehabilitation unit more than 4 calendar days prior to enrollment into to SP or RP.

3.0 GENERAL ANALYSIS POPULATIONS, DEFINITIONS, AND CONVENTIONS

3.1 Analysis Populations

3.1.1 Pre-screened Population

The pre-screened population consists of all patients evaluated for candidacy for XR-NTX and for basic study eligibility criteria as listed on the ENRA form.

3.1.2 Screened Population

The screened population consists of participants who sign the informed consent (IC) at the initiation of the screening process. All screened participants are captured on the ENRB form. Screen failures and reasons for failure are captured on the ENRZ form.

3.1.3 Intent-to-Treat Population

The Intent-to-Treat (ITT) population consists of participants who enroll in the study in either Standard Procedure (Segment C) or Rapid Procedure (Segment D). This will be determined by the induction procedure the site is assigned to at the time of participant enrollment.

3.1.4 Safety Population

This study records safety events for all participants that sign IC. Therefore, the safety population consists of all participants who sign IC.

3.1.5 Inducted Population

The inducted population consists of enrolled participants that receive the first XR-NTX while in the unit (i.e., all success for the primary outcome).

3.1.6 Provider/Staff Population

The provider/staff population includes providers and staff at the sites who complete the Organizational Readiness to Change (ORCA) survey (OR1/OR2), and/or the site-level Organization Level Clinical Implementation (OLC), and Fidelity to Implementation Checklist (FC1/FC2) assessments.

3.2 General Definitions

3.2.1 Induction Phase

The induction phase Day 1 is defined as the day of the admission (E97ADMDT) as recorded on the ENRC or ENRD forms. The induction phase for enrolled participants begins with day of admission (induction phase Day 1) and ends on the day of XR-NTX receipt or day of failure of the induction protocol. The maximum length of the induction phase is 30 days (Day 1 – Day 30). Note that the end of the induction phase does not always coincide with the participants being discharged from the unit.

3.2.2 Post-induction Phase

The post-induction phase Day 1 is defined as the day after receipt of the first XR-NTX injection (EOI.EOIINJDT) or the day after failing the induction protocol (EOI.EITERMDT). The planned length of the post-induction phase is 56 days (Week 1 – Week 8) with the upper window for the last post-induction visit set at Day 62 (Day 56 + 6-day window). For successful inductions (i.e., those that receive first XR-NTX while on the unit), study visits are planned at Week 1 (Day 6+/- 4 days), Week 2 (Day 14+/- 3 days), Week 3 (Day 21+/- 3 days), Week 4 (Day 28+/- 3 days), Week 6 (Day 42+/- 6 days), and Week 8 (Day 56 +/- 6 days). For induction failure (i.e., those that do not receive first XR-NTX while on the unit), study visits are planned at Week 4 (Day 28+/- 3 days), and Week 8 (Day 56 +/- 6 days).

3.2.3 Baseline Visit

The baseline visit is performed on day of enrollment in the study Segments C or D or after for all participants that sign the IC. The baseline dates may vary across different assessments.

3.2.4 Baseline Value

The baseline value will be defined as the assessment collected at the Baseline Visit or the first available record starting with the admission day.

3.2.5 Safety Window

The safety window for all participants enrolled in the study begins at day of signing the IC and ends at 30 days post last study visit.

3.2.6 Targeted Safety Event

The targeted safety events (TSEs) for this study are the events listed below and recorded on the Targeted Safety Event (TSE) form:

- Fall event (related to medical/psychiatric condition such as dizziness, confusion with head injury)
- Acute change in mental status (i.e., disorientation, amnesia, cerebrovascular accident, coma)
- Acute medical complication likely exacerbated by the stress of withdrawal (i.e., hypertensive crisis, hypotensive event with medical sequelae such as fall and/or requiring urgent fluid resuscitation, severe chest pain, acute respiratory decompensation, asthma attack, diabetic ketoacidosis, severe hypoglycemia, severe electrolyte abnormalities (hyper-/hyponatremia, hyper-/hypokalemia), precipitated withdrawal)
- Acute psychiatric symptoms (i.e., psychosis, hypomania, severe agitation, violence)

Other safety events will also be captured on study specific forms (for example, COWS, SOWS, PHQ-9, Medication Injection Site Abnormality Log and Overdose Questionnaire).

TSEs and other safety events captured on study specific forms will not also be reported as an AE unless the event meets the serious adverse event (SAE) definition.

3.2.7 Serious Adverse Event

An AE is any untoward medical occurrence in humans, whether or not considered study medication/intervention related which occurs during the conduct of a clinical trial. Any change from baseline in clinical status that is considered clinically significant by the site medical clinician are considered AEs. A suspected adverse reaction is any AE for which there is a reasonable possibility that the study medication/intervention caused the AE. A reasonable possibility implies that there is evidence that the study medication/intervention caused the event. Adverse reaction is any AE caused by the study medication/intervention.

An adverse event, suspected adverse reaction, or adverse reaction is considered serious (i.e., a serious adverse event, serious suspected adverse reaction or serious adverse reaction) if, in the view of either the site medical clinician or sponsor, it:

- Results in death: A death occurring during the study, or which comes to the attention of the study staff during the protocol-defined follow-up period, whether or not considered caused by the study medication/intervention, must be reported.
- Is life-threatening: Life-threatening means that the study participant was, in the opinion of the medical clinician or sponsor, at immediate risk of death from the reaction as it occurred and required immediate intervention.
- Requires inpatient hospitalization or prolongation of existing hospitalization.

- Results in persistent or significant incapacity or substantial disruption of the ability to conduct normal life functions.
- Is a congenital abnormality or birth defect.
- Is an important medical event that may not result in one of the above outcomes but may jeopardize the health of the study participant or require medical or surgical intervention to prevent one of the outcomes listed in the above definition of serious event.

3.3 Table, Figures and Listings Conventions

Data for pre-screened and screened populations will be summarized by site and step. Data for the ITT population will be summarized by induction procedure (SP or RP). Additionally, most analyses for the ITT population will also be summarized by site excluding supportive analysis for primary outcome and key secondary outcomes. Primary outcome will be summarized by site, step and induction procedure (SP or RP). Analyses for the safety population will be summarized by induction procedure (SP or RP). Data audits and data quality (e.g., protocol deviations) will be summarized by site.

Continuous variables will be summarized using the following descriptive statistics: n (non-missing sample size), mean, standard deviation, percentiles (median, 25th and 75th percentiles, maximum and minimum). Categorical variables will be summarized in terms of frequencies and/or percentages.

Any deviations from the above general conventions will be noted in the subsequent sub-sections.

4.0 PARTICIPANT ENROLLMENT, DISPOSITION, AND VISIT ATTENDANCE

4.1 Participant Enrollment

The number of participants pre-screened and screened and the reasons for ineligibility on pre-screening and screening will be summarized by site and step. Note that participants might be pre-screened twice. For participants who were pre-screened twice, they will only be considered for the second screening.

The trajectory of actual enrollment versus the expected number of enrollments according to the start date of each step of the stepped wedge design and based on a monthly expected enrollment rate of 4.675 per site will be graphed by site and overall. Proposed versus actual enrollments will be summarized by site in a tabular fashion. The distribution of enrollments by site, step, and induction procedure will be presented.

4.2 Participant Disposition

Participants are defined as study completers if the Week 8 Post-induction Visit is completed as indicated on the Study Completion (STC) form and are considered early study terminations if this visit is not completed. Participant disposition will be summarized by site and induction procedure for the number of participants completing the study, the number of participants early terminating from the study and the reasons for early study termination.

The CONSORT flow diagram will be generated (Moher et al., 2010).

4.3 Visit Attendance at Post-induction Visits

The number and percentage of participants who attend the post-induction period study visits during Weeks 1 (Day 7), 2 (Day 14) ,3 (Day 21), 4 (Day 28), 6 (Day 42), and 8 (Day 56) will be presented by induction procedure and by site. Information on missed visits will also be presented by induction procedure and by site and will include the number of missed visits, the number of participants with at least one missed visit, and the reasons for the missed visits. The expected

number of visits during the post-induction period is calculated based on the general rule that 6 visits are expected per participant who receive the first XR-NTX and two visits (weeks 4 and 8) are expected for participants who fail to receive the first XR-NTX while on the unit during the induction period. The average number of missed visits per participant will be calculated by dividing the number of missed visits by the number of participants. For early study terminations, visits are only considered missed during active study participation if they occur before the study termination date.

5.0 ANALYSIS OF PARTICIPANT BASELINE CHARACTERISTICS

Baseline demographics and characteristics including sex (at birth) (DEM), age, ethnicity, race, education level, marital status, and employment will be summarized by site and induction procedure for the ITT population. The following additional important baseline characteristics will also be summarized by site and induction procedure: gender and sexual orientation (S97), homelessness status (NMS), first COWS score recorded on Day 1 of admission (COW), history of overdose (ODQ), number of days since last self-reported opioid use at Day 1 of admission (TAP), baseline substance use (UDS and TLFB), medical and psychiatric history (MHX), baseline mental health screening: Adult ADHD Self-Report Screening Scale for DSM-5 (Score ≥ 14) (AAS), PTSD Checklist for DSM-5 (Score ≥ 31) (PCL), Generalized Anxiety Disorder (Score ≥ 8) (GA7), Patient Health Questionnaire (PHQ-9) (Score ≥ 10), criminal history (NMS), health insurance/care plan (NMS), history of medication for opioid use disorder (NMS), number of times attempted and completed opioid detoxification (MHX) and previous unsuccessful inductions onto XR-NTX (MHX). Age will be summarized as a continuous and categorial variable. A summary of baseline demographics and characteristics will also be presented for study completers. If differences between induction procedures are suspected, statistical testing may be performed.

6.0 MEDICATIONS ADMINISTERED DURING INDUCTION PHASE

The daily medications for an enrolled participant for each day of the induction phase are reported on the Daily Medication Log (DMA) form. These data are abstracted from medical records starting with admission Day 1 and up to the last day of the induction phase. The number of participants with at least one dose of each medication will be summarized by induction procedure. The daily medication log will be summarized for each day of the induction phase by induction procedure. Medications administered in the Standard Procedure will be summarized by Buprenorphine Day, Buprenorphine Washout Day, Naltrexone Day, Day of XR-NTX Injection Success or Day of XR-NTX Injection Failure. Medications administered in the Rapid Procedure will be summarized by Pre-Buprenorphine Day, Buprenorphine Day, Buprenorphine Washout Day, Naltrexone Day, and Day of XR-NTX Injection Success or Day of XR-NTX Injection Failure.

7.0 TREATMENT EXPOSURE

During the study (induction and post-induction phase), three XR-NTX study injections are expected per participant. First study injection is recorded on the EOI form, and study injections 2 and 3 are recorded on the INN form. The treatment exposure percentage is calculated as the number of injections administered divided by the number of injections expected. Regardless of induction procedure, participants are expected to receive the first XR-NTX injection within 30 days maximum from admission, and second and third XR-NTX injections at Day 28 and at Day 56 post first injection, respectively. Treatment exposure will be summarized by site and by induction procedure. Note that a study injection is defined as any XR-NTX injection given from the CTN-0097 study supply or from CTN-0100 supply for cross-over participants. Any XR-NTX injection received outside of the CTN-0097 or CTN-0100 supply is not considered study injection, and is recorded on the Timeline Followback Medications (M97) form. XR-NTX injections received by

participants who failed their initial induction while on the but later received an injection during the post-induction period are recorded on the M97 form and will be summarized by site and original induction procedure separately.

8.0 EFFECTIVENESS ANALYSIS

8.1 Definition of the Primary Outcome Measure

The primary outcome measure is the proportion of participants who receive the first XR-NTX injection (dichotomous: participant did or did not receive first injection of XR-NTX) while on the treatment unit within 30 days of admission. The primary objective of the study is to show RP is non-inferior to SP XR-NTX induction method. The hypothesis is that RP will be non-inferior to SP in terms of proportion of participants with successful inductions (receipt of first XR-NTX injection) while on the unit. The primary outcome will be determined from the EOI form using the EINTXIND variable for Segment C and D.

8.2 Analysis of the Primary Outcome Measure

A summary of the number of participants who receive their first injection will be presented by site, by step and by induction procedure. The main analyses for the primary outcome will use the ITT population. As is standard for the analysis of stepped-wedge designs (Barker et al., 2016; Hussey and Hughes, 2007), the primary outcome analysis for non-inferiority will be performed using a generalized linear mixed-effects model with a logistic link. The log odds of a participant receiving the first XR-NTX injection (yes/no) will be modeled as a function of which induction procedure (RP vs SP), step 1 through 5 (to control for secular trends), and a random effect for site to control for nesting of participants within site. The null hypothesis of inferiority of RP to SP will be rejected if the lower 95% confidence limit of the odds ratio of success [odds(RP)/odds(SP)] exceeds 0.67. If null hypothesis of inferiority of RP to SP is rejected, then superiority of RP will be tested. If the 95% confidence interval for the odds ratio is above 0.67 and is also above 1, then there will be evidence of superiority and it is acceptable to calculate the p-value (CPMP, 2001). The covariance structure compound symmetry (CS, correlations over time are constant) will be used for the observations from a particular site. Other correlation structures will be examined (Hemming et al., 2017). The best model will be selected based on the lowest Bayesian Information Criterion (BIC). The Satterthwaite method to adjust for denominator degrees of freedom for tests of the fixed effects will be used.

Logistic model will be used:

$$logit(p_{ijs}) = J_{js} * \delta + \theta_s + \alpha_j$$

$$\alpha_j \sim N(0, \tau^2)$$

Alternative logistic model with autoregressive lag-1(AR(1)) correlation structure:

$$logit(p_{ijs}) = J_{js} * \delta + \theta_s + \alpha_{js}$$

$$\alpha_{js} \sim N(0, \tau^2)$$
$$Cov(\alpha_{js}, \alpha_{jt}) = \tau^2 \rho^{|t-s|}$$

where:

- j indexes the site
- s indexes the step
- i indexes the individual within site j at time s
- θ_s is the fixed effect of time

- α_j is the random site effect
- p_{ijs} is the probability of success for individual i within site j at time s
- J_{js} is the treatment indicator for site j at time s
- δ is the treatment effect
- t and s index two different steps (in AR(1) model)

SAS code example:

```
proc glimmix data = primout method = quad IC = PQ;
  class site trt (ref='SP') step (ref='1');
  model success (event='1') = trt step / dist = binary link = logit solution oddsratio;
  random intercept / subject = site;
run;
```

SAS code example AR(1):

```
proc glimmix data = primout method = quad IC=PQ;
  class site trt(ref='SP') step (ref=1');
  model success (event='1')= trt  step / dist = binary link = logit solution oddsratio;
  random step / subject=site type=ar(1);
run;
```

where:

- success - is the XR-NTX injection status (Yes=1 or No=0)
- trt - is the variable that defines the induction procedure (RP or SP)
- step - is 1 through 5
- site - is the site code for the 6 sites.

The Number Needed to Treat (NNT) will be calculated as the inverse of the absolute difference in rates of success i.e., 1 / (rate of success in RP – rate of success in SP).

8.3 Supportive Analyses of the Primary Outcome Measure

The DSC will perform the following supportive analyses related to the primary objective:

1. Subgroup analyses for age (18 – 25 years, 26 years or greater), sex (Male, Female), race (Black, White, Other), and ethnicity (Hispanic or Latino, Not Hispanic or Latino) will be performed as required by the NIH (NIH, 2016). Four models similar to the main primary outcome analyses will be performed, with the inclusion of an interaction term between induction procedure and the demographic subgroup. Forest plot of odds ratios for each subgroup will be provided.

2. Exclude six participants from the ITT as noted below and repeat the analysis described in Section 8.2.

   The study exclusion criteria #10 was updated in the amended protocol v3.0 from the initial criteria that stated: “Admitted to the inpatient detoxification or residential rehabilitation unit more than 3 days prior to consent” and was changed to “Admitted to the inpatient detoxification or residential rehabilitation unit more than 4 calendar days prior to enrollment into to SP or RP”. Under protocol v2.0, six participants enrolled in the study who were admitted to inpatient detoxification or residential rehabilitation more than 4 calendar days prior to enrollment. A sensitivity analysis will be conducted for the primary outcome measure that excludes these six participants, per DSMB recommendations on May 5, 2022.

3. For sites that transition from SP to RP a sensitivity analysis will be conducted excluding all participants in the SP in the 8 weeks before crossing over to the RP (i.e., pre-implementation phase) to account for any possible contamination of the SP arm with the RP training.
4. Test for an induction procedure by study month interaction, which, if significant, would indicate differential impact of induction procedure after a longer period of experience.
5. Repeat primary outcome analysis with the inclusion of a covariate for fentanyl use (positive vs. negative) as assessed on the Urine Drug Screen (UDS) form at screening or baseline assessments.
6. Summarize the primary outcome by induction procedure in the subset of participants who initiated buprenorphine.

Other supportive analysis to be performed by LN related to the primary outcome include:

- Repeating analyses in Section 8.2. with the inclusion of potential baseline covariates that were found to be unbalanced between sites or any other baseline covariates that are deemed important predictors of the outcome.
- Prediction in terms of participant level factors that predict success versus failure to initiate XR-NTX adjusting for the following covariates: severity of opioid use disorder based on route of use (IV vs. non-IV) with IV users having a more severe disorder, type of opioid (fentanyl vs. heroin vs. prescription opioid) with fentanyl users being most severe and prescription opioid users least severe, as well as psychiatric and substance use disorder comorbidity will be explored. Site level factors, measured with the implementation measures will also be explored. Although, with 6 sites power for that would be limited. It could be a nested prediction model with patients nested within sites, and site level factors (like staff knowledge and attitudes) in the model.

8.4 Definition of the Secondary Outcome Measures

<u>Key secondary analyses to be conducted by the DSC</u>:

A key secondary objective is to summarize the below outcome to confirm expected characteristics of the RP compared to SP:

1. The time to receipt of first injection of XR-NTX as measured by the number of days from admission to first XR-NTX injection. This will be conducted on the successfully inducted population only. Day of admission is defined as Day 1 (E97ADMDT) as recorded on the ENRC or ENRD forms, and day of first XR-NTX injection (EOIINJDT) is captured on the End of Induction form (EOI). It will be calculated as Date of injection (EOI.EOIINJDT) – Date of admission (ENRC/ENRD.E97ADMDT) +1.

Additional key secondary objectives are to compare the below outcomes of RP versus SP for all enrolled participants (ITT population).

2. Craving for opioids measured by Visual Analog Scales (VAS) as captured daily on the self-reported inpatient opioid craving form (OCI) during induction phase. The OCI form collects intensity of craving both at time of assessment and within the last 24 hours prior to assessment. If either measurement is collected multiple times on the same assessment date, the average measurement will be used for analysis.
3. Opioid withdrawal symptoms as measured by the Subjective Opioid Withdrawal Scale (SOWS) and the Clinical Opiate Withdrawal Scale (COWS). SOWS is captured on the SBW form at baseline and daily during induction phase. COWS is captured on the COW

form starting with day of admission and daily during the induction phase leading to the fist XR-NTX injection. Note that during induction, COWS may be measured multiple times per day while SOWS is expected to be assessed only once per day. If multiple SOWS measures are collected on the same assessment date, the average measurement will be used for analysis. If multiple COWS measures are collected on the same assessment, the peak measurement will be used.

4. Retention in the trial to receive at least one XR-NTX injection after the first injection as captured on the XR-NTX Administration (INN) form.
5. Safety, as measured by targeted safety events, overdoses, and serious adverse events related to study medication during the induction phase and during eight weeks of post-induction. Detail on safety outcome analysis is provided in Section 9.0.

<u>Other secondary analyses to be conducted by LN</u>:

Other secondary objectives to be analyzed by the LN are to compare the below outcomes of RP vs. SP for participants who receive the first injection while on the unit (Inducted Population):

- Opioid abstinence, as measured weekly by the Timeline Followback (TLFB) (self-report days using opioids) during the eight weeks of post-induction and proportion of opioid-positive urine tests as captured on the Urine Drug Screen (UDS) form at post-induction 4 week and 8 week visits.
- In addition, craving for opioids, opioid withdrawal symptoms, and safety events will be assessed in the inducted population similar to the key secondary outcomes.

Table 5: Secondary Clinical Outcomes, Outcome Measures and Hypotheses

Outcome	Outcome Measure	Hypothesis
Time from admission to first XR-NTX injection	Days to first XR-NTX injection	Participants in the RP will receive their first injection significantly faster than those in the SP. Significance/Rationale: The RP decreases the time to the first injection (which has a potential to decrease costs and staff burden).
Opioid craving (VAS) over time	Mean for opioid craving measured by VAS daily during days leading to the first XR-NTX injection, and during post-induction Weeks 1-8	Participants in RP and SP will have comparable intensity of craving during 1) the inpatient treatment period, and 2) during the first week after the first XR-NTX injection.<br>Significance/Rationale: Earlier trial showed comparable craving severity in both procedures (Sullivan *et al.*, 2017).
Opioid withdrawal (SOWS and COWS) over time	Mean for opioid withdrawal measured by SOWS and COWS score, daily (starting with day of admission) during days leading to the first XR-NTX injection and during post-induction Weeks 1-8	Participants in RP and SP will have comparable severity of opioid withdrawal during: 1) the inpatient treatment period, and 2) during the first four weeks after the first XR-NTX injection.<br>Significance/Rationale: Earlier trial showed comparable withdrawal severity in both procedures (Sullivan *et al.*, 2017).

Table 5: Secondary Clinical Outcomes, Outcome Measures and Hypotheses

Outcome	Outcome Measure	Hypothesis
Targeted safety events, overdoses and SAEs, related to study medications	Frequency of targeted safety events, overdose episodes and SAEs by relationship to study medication during the induction period and during eight weeks of post-induction treatment	RP and SP will produce equivalent rates of targeted safety events and SAEs during the induction and during the first eight weeks of treatment with XR-NTX.<br>Significance/Rationale: Careful documentation of SAEs, targeted safety events, and overdose episodes, would be considered essential safety data, and important component of a comparative effectiveness trial.
Receive second and third injections (binary: did or did not receive second and third dose of XR-NTX)	Proportion of participants that receive second and third injection of XR-NTX (at 4 weeks and 8 weeks, from first injection)	Participants in RP and SP will have comparable rates of treatment retention.<br>Significance/Rationale: Because there is no difference in tolerability of the first and second XR-NTX injections in both study arms, we do not expect differential treatment dropout.
Use of opioids over time during the 8-week of post-induction treatment while on study medication (Weekly TLFB, confirmed by urine drug screens when available)	Percent of participants positive for opioids using weekly TLFB during eight weeks of post-induction, and urine drug screens at week 4 and 8.	RP and SP will produce comparable levels of opioid use.<br>Significance/Rationale: XR-NTX produces complete blockade of opioid effects, so that during treatment with monthly injections, opioid use can be expected to be minimal and no different between study arms.

8.5 Analyses of the Key Secondary Outcome Measures

The key secondary outcome, days to first injection, will be summarized by induction procedure. It will be compared between the two induction procedures in the inducted population using a Cox proportional hazards model. The log hazard rate of receiving the first XR-NTX injection will be modeled as a function of induction procedure (RP vs SP), step and site. The SAS PHREG procedure will be used to estimate the hazard ratio for the induction procedures. The 95% Wald Confidence limits will be estimated.

SAS Code example:

```
proc phreg data=inject;
  class trt step site;
  model time*censor (0) = trt step site;
  hazardratio trt;
run;
```

where:

- time - is the time to event variable, i.e., the days to first injection.
- censor - is the censoring indicator variable. The censoring value (censor=0) means censored. However, given the analysis is conducted in the inducted population only, there will be no censored observations, i.e., censor=1 for all observations.
- trt - is the variable that defines the induction procedure (RP or SP)

- step - is 1 through 5
- site - is the site code for the 6 sites.

The other key secondary outcomes including craving for opioids (VAS) and opioid withdrawal measures (COW and SOW) will be summarized in tabular and graphical form for each day of the induction phase by induction procedure. In the Standard Procedure, scores will be summarized by Buprenorphine Day, Buprenorphine Washout Day, Naltrexone Day, Day of XR-NTX Injection Success or Day of XR-NTX Injection Failure. In the Rapid Procedure, scores will be summarized by Pre-Buprenorphine Day, Buprenorphine Day, Buprenorphine Washout Day, Naltrexone Day, Day of XR-NTX Injection Success or Day of XR-NTX Injection Failure.

Longitudinal opioid craving and withdrawal scores may be analyzed using mixed-effects models adjusting for induction procedure, site, step, and days since admission. The presence of at least one moderate to severe daily COWS score (maximum score ≥12) and presence of moderate to severe average daily COWS score (average score ≥12) will be analyzed using a mixed-effects model with logit-link function.

SAS code example that may be used to analyze binary COWS outcome:

```
proc glimmix data = secondout method = quad IC=PQ;
  class patid site trt(ref='SP') step (ref='1');
  model outcome (event='1')= trt site day step/ dist = binary link = logit solution oddsratio;
  random intercept / subject=patid type=ar(1);
run;
```

where:

- trt - is the variable that defines the induction procedure (RP or SP)
- outcome - is the presence of moderate to severe daily COW score (Yes=1 or No=0)
- step - is 1 through 5
- site - is the site code for the 6 sites.
- day- days since admission to the unit
- patid- participant ID

SAS code example that may be used to analyze continuous SOWS and VAS outcomes:

```
proc mixed data = secondout;
  class patid site day_cat trt(ref='SP') step (ref='1');
  model outcome = trt site day step/ solution cl;
  random intercept / subject = patid;
  repeated day_cat/ type = AR(1) subject = patid;
  estimate "trt effect" trt 1 -1;
run;
```

where:

- trt - is the variable that defines the induction procedure (RP or SP)
- outcome - is either mean SOWS or mean VAS score
- step - is 1 through 5
- site - is the site code for the 6 sites
- day - days since admission to the unit (continuous)
- day_cat- days since admission to the unit (categorical) as required by the repeated statement in the model
- patid- participant ID

Retention in the trial to receive at least one XR-NTX injection after the first inpatient injection as captured on the XR-NTX Administration (INN) form will be analyzed similarly to the primary outcome using a mixed-effects model with logit-link function.

Frequency of targeted safety events, overdose episodes and SAEs by relationship to study medication will be reported. Safety data analysis is provided in detail in Section 9.0.

8.6 Definition of the Exploratory Outcome Measures

Exploratory objectives include:

1. Explore baseline demographic and clinical features (e.g., the primary opioid of dependence (heroin/fentanyl vs. prescription opioid)) as: a) predictors of induction success, secondary outcomes and retention during the trial (main effect of predictors), and b) as moderators of differential treatment effect (moderator by treatment interaction).
2. Compare duration of inpatient treatment and the associated costs from the time when detoxification is initiated to the time that XR-NTX is administered to permit analyses of economic costs and benefits of the two induction procedures. Also compare duration of inpatient treatment for all patients regardless of whether XR-NTX was initiated, across RP and SP for ITT population. Participants who failed induction will be evaluated to see if they received any other MOUD.
3. Compare RP versus SP for all enrolled participants in terms of time from day of admission to XR-NTX initiation failure (day of discontinuation of detoxification period that resulted in failure to receive first XR-NTX) and reasons for failure.
4. Compare RP versus SP for all enrolled participants in terms of other depressive, anxiety, and subacute withdrawal symptoms as measured by the Patient Health Questionnaire-9 (PHQ-9) and General Anxiety Disorder-7 (GAD-7).
5. Compare RP versus SP use of alcohol and other drugs of abuse (e.g., cocaine, other stimulants, cannabis, benzodiazepines), by self-report and urine drug screens for all enrolled participants.
6. Explore engagement with medical visits and therapy (based on Medical Management Log, Psychosocial Log, XR-NTX Administration Form, TLFB).
7. Compare RP versus SP for all enrolled participants in terms of use of MOUD as measured by patient self-report on Timeline Followback (TLFB).
8. Investigate the percentage of induction failure participants that receive XR-NTX during the course of the study overall and for each induction procedure as measured by patient self-report on Timeline Followback.
9. Compare RP versus SP for percentage of participants inducted on XR-NTX (during both induction and post-induction phases of the trial).

8.7 Missing Data Analysis

No missing data is expected for the primary outcome. Data on whether participants received the first XR-NTX injection while on the treatment unit is always available.

9.0 IMPLEMENTATION OUTCOME ANALYSIS

Implementation outcomes, such as participant ratings of acceptability and satisfaction with treatment, and clinicians' ratings of knowledge and attitudes toward XR-NTX and XR-NTX induction methods before and after sites' protocol participation, will be modelled as continuous outcomes using mixed-effects linear regression models, with random intercepts for site. Additional

implementation outcome measures including acceptability and barriers are measured qualitatively.

10.0 SAFETY OUTCOMES AND ANALYSIS

Safety outcomes for this study include targeted safety events, serious adverse events, death, injection site abnormalities, overdoses, suicide risk, and pregnancy. Safety outcomes analysis will be performed on the safety analysis population as defined in Section 3.1.4. Safety outcomes will be summarized by induction procedure (SP or RP) and overall. The induction procedure will be determined by the induction procedure the site was assigned to at the time of participant enrollment.

10.1 Targeted Safety Events

Study defined targeted safety events (TSEs) are defined in Section 3.2.7. TSEs will be summarized by presenting the number and types of TSEs, relatedness to study medication, SAE classification, and number of participants experiencing TSEs by induction procedure and phase (i.e., Screening, Baseline, Induction and Post-Induction). Comparison of TSEs by induction procedure will be conducted using Fisher's exact test.

Detailed listings of TSEs by induction procedure will be provided. The listing will include Participant ID, date of IC, date of enrollment, date of TSE, TSE type and detail, severity of TSE, relatedness to study medication, SAE classification, and any additional comments collected on the form.

10.2 Serious Adverse Events

Serious adverse events (SAEs) are defined in Section 3.2.8. SAEs will be summarized by presenting the number of events, number of participants experiencing SAEs, and the relatedness and type of SAEs by induction procedure.

All SAEs will be coded using MedDRA® dictionary version v25.0. The number and proportion of participants experiencing SAE will be provided by induction procedure and overall. SAEs will be summarized by System Organ Class (SOC) and Preferred Term (PT). The proportion will be calculated as the number of participants who experience the event at least once divided by the number of participants in the induction procedure or overall. Proportions will be calculated at the preferred term level, at the SOC level, and for participants with at least one SAE. If a participant experiences multiple episodes of an event, then the event is only counted once. Detailed listings of SAEs by induction procedure will be provided. The listing will include Participant ID, date of IC, date of enrollment, SAE onset date, description, severity of AE, relatedness to study medication, outcome, resolution date, reason reported as SAE, and MedDRA® coded preferred term and system organ class. Narratives for all serious adverse events will be provided.

10.3 Death

A listing of deaths and narratives by induction procedure will be provided.

10.4 Injection Site Abnormalities

The Injection Site Abnormality (INA) form records any time an injection is given and there is an abnormal reaction observed at the injection site. Injection site abnormalities will be summarized by number of injection site abnormalities, type of abnormality, and the severity of the abnormality. A detailed listing of injection site abnormalities for injections by induction procedure will be provided. The listing will include Participant ID, date of IC, date of enrollment, date of injection, injection number, injection location, abnormality start and resolution date, symptom, severity, and SAE classification.

10.5 Overdoses

The Overdose Questionnaire (ODQ) form assesses each participant's self-reported opioid overdose history, as well as information regarding the most recent overdose event. A summary table of non-fatal opioid overdoses will be provided by induction procedure and by study visit (at baseline and at each post induction visit). At the baseline visit, participants are asked about any opioid overdoses prior to enrollment. At the post-induction visits, participants are asked about opioid overdoses since their last visit. The number of participants with at least one opioid overdose and the number participants with the most recent overdose where NARCAN (naloxone) was used to reverse overdose and resulted in being admitted to the hospital will be reported. A detailed listing of non-fatal opioid overdoses by induction procedure will be provided. The listing will include Participant ID, date of IC, date of enrollment, visit, date of assessment, overdose history, NARCAN use, hospital admission, substance used, and the five questions asked on a scale of 0 to 10.

10.6 Suicide Risk

The Patient Health Questionnaire-9 (PHQ-9) screeners are conducted at baseline, weekly during the induction phase, and at each post-induction visit to assess suicide risk. A summary table of participants endorsing suicidality at least once on PHQ-9 during baseline, induction and post-induction phase will be presented by induction procedure. On the PHQ-9, a participant is considered to have endorsed suicidality if they indicate several days, more than half the days, and nearly every day having thoughts they are better off dead or of hurting themselves. A listing of visits for participants who endorse suicidality at any visit will be provided by induction procedure.

10.7 Pregnancy

A listing of pregnancies and pregnancy outcomes for any participant in the safety population will be generated by induction procedure. Narratives will also be provided.

11.0 SIGNIFICANCE TESTING AND MULTIPLICITY

There is only one primary outcome and therefore no adjustments for multiple testing are planned for the primary outcome analysis. The primary outcome and the supportive analysis for the primary outcome will use a one-sided test with 2.5% type I error rate. A similar error rate will be used if the test of superiority is performed. A restricted list of secondary outcomes was chosen a priory, and, in addition, the key secondary outcomes are not intended to be confirmatory in nature. Therefore, no multiple adjustments are planned for analyzing multiple secondary outcome measures. When multiple tests are conducted, the chance of finding a significant difference in one of the tests, when in fact no difference exists, is greater than the stated type I error rate. The investigators are aware of the issues associated with multiple testing and will interpret results with caution. The secondary outcomes will use a two-sided test with 5% type I error rate.

12.0 SAMPLE SIZE AND POWER

The CTN-0097 study design is represented schematically as the Design Pattern matrix of Figure 1. The design pattern matrix gives table a schematic representation of the optimized stepped-wedge design compared with a standard stepped-wedge design alternative. Rows are sites, columns are blocks of time (steps), and cells contain 0 for SP or 1 for RP. The optimized stepped-wedge design provides more power to detect a treatment effect than a similar standard stepped-wedge design (Thompson *et al.*, 2017).The primary outcome measure is the proportion of patients who receive the first XR-NTX injection at the end of the induction phase, which is approximately Day 6 in the RP and Day 13 in the in the SP (binary: did or did not receive first injection of XR-

NTX).

Figure 1: Design Pattern Matrix

Optimized Stepped-Wedge Design

Time Block

		1	2	3	4	5
	1	1	1	1	1	1
	2	0	1	1	1	1
Site	3	0	0	1	1	1
	4	0	0	0	1	1
	5	0	0	0	0	1
	6	0	0	0	0	0

Standard Stepped-Wedge Design

Time Block

		1	2	3	4
	1	0	1	1	1
	2	0	1	1	1
Site	3	0	0	1	1
	4	0	0	1	1
	5	0	0	0	1
	6	0	0	0	1

Values of Parameters Underlying Power Simulations

Probability of Success in the SP arm: The assumed success probability in the SP initiation regimen is based on the mean success rate in the XR-NTX arm of CTN-0051 (Lee *et al*., 2018), which is 55% for sites 02011, 02017, and 02052 pooled together. These are sites that are similar to the types of sites that would be eligible for this study.

Probability of Success in the RP arm: A difference in the proportion of successes of 15% with an overall success of 55% in the SP arm (as in study CTN-0051) and 70% in the RP are assumed. Estimates are based on the results of a prior controlled study, which compared Standard and Rapid XR-NTX procedures and found a 23.4% difference (56.1% vs. 32.7%) (Sullivan *et al.*, 2017). A slightly smaller true treatment difference (15%) is assumed, due to the more structured, inpatient/short-term residential setting of sites in the current study

Margin of non-inferiority: To show non-inferiority of RP to SP, a 10% margin of non- inferiority is assumed, which corresponds to an odds ratio of 0.67 (proportion of success in RP = 0.45, proportion of successes in SP = 0.55). To show RP is non-inferior to SP, the lower bound of the two-sided 95% CI for the odds ratio for RP vs SP needs to be higher than 0.67.

Under the above assumptions, the null and alternative hypotheses can be stated as follows: In terms of proportions (p):

Null (inferiority of RP to SP): p(RP) - p(SP) <=-10%

Alternative (non-inferiority of RP to SP under which power was calculated): p(RP) - p(SP)

> 15%

In terms of odds ratios (OR):

Null (inferiority of RP to SP): OR [odds(RP)/odds(SP)] < = 0.67

Alternative (non-inferiority of RP to SP under which power was calculated): OR [odds(RP)/odds(SP)] > 1.91

Intraclass Correlation Coefficient: The ICC estimate is based on a logistic regression of induction success/failure in CTN-0051 on treatment arm, with a random site effect whose variance is allowed to depend on arm. It is assumed that any extra covariates added to the model will improve

analysis accuracy, implying that current power estimates are conservative. The estimated site standard deviation in the XR-NTX arm from the regression model using data from CTN-0051 is 0.86, leading to an estimated ICC in the SP arm of 0.14 under the generalized mixed model. The ICC represents the correlation between two individuals chosen randomly without replacement from the XR-NTX arm of CTN-0051, given that they come from the same site.

In summary, the assumptions used for the power calculations for this non-inferiority optimized stepped-wedge cluster randomized trial are as follows:

- The projected number of clusters is 6 (sites), that will enroll participants across 5 periods of time, each period 14 weeks long.
- The projected total number of participants enrolled is 450, with 15 participants enrolled per cluster per time period (equal allocation to sites per time period).
- The outcome of interest is a binomial outcome (the participant received or did not receive the first XR-NTX injection).
- The probability of success in the SP arm is 0.55.
- The probability of success in the RP arm is 0.70 (i.e., an effect size of 0.15).
- The margin of non-inferiority is 10%, which corresponds to an odds ratio of 0.67 (based on proportion of success in RP = 0.45 and proportion of successes in SP = 0.55). Refer to Figure 5 for the relationship between non-inferiority margin as a difference in proportions versus non-inferiority margin as an odds ratio.
- Observations are equally correlated within cluster, regardless of time or induction method with a site standard deviation of 0.86. The corresponding ICC in the SP arm based on a logistic regression with random effects is 0.14.

The above assumptions are varied to evaluate the sensitivity of the power calculations to different parameter values such as different ICCs, effect sizes, or number of sites.

For the power calculations, correlated binary data were simulated using the Parzen algorithm [*Parzen, M., 2009*] and power analyses were performed using the logistic model below.

Logistic model used for power calculations:

$$logit(p_{ijs}) = J_{js} * \delta + \theta_s + \alpha_j$$

$$\alpha_j \sim N(0, \tau^2)$$

$$y_{ijs} \sim \mathrm{Bernoulli}(\mathrm{p_{ijs}})$$

where:

- j indexes the site
- s indexes the 4-month block of time
- i indexes the individual within site j at time s
- θ_s is the fixed effect of time
- α_j is the random site effect
- p_{ijs} is the probability of success for individual i within site j at time s
- J_{js} is the treatment indicator for site j at time s
- δ is the treatment effect
- y_{ijs} is the outcome (0=Failure,1=Success) for individual i withing site j at time s

For all simulations, 10,000 iterations were used.

A SAS code snippet capable of estimating the above model follows:

```
proc glimmix data = simul method = quad;
  class site trt time;
  model success = trt time / dist = binary link = logit solution oddsratio;
  random intercept / subject = site;
run;
```

Power Curve

Two-tailed power to show non-inferiority of RP to SP at alpha level 0.05 is shown in Figure 2 as a function of ICC. For the sake of clarity, the vertical power scale runs from 0.6 to 1 instead of the conventional 0 to 1. Horizontal and vertical reference lines denote power = 80% and ICC = 0.14, respectively. Figure 2 depicts two power curves (explained below), but the uppermost curve (black line) shows power for the optimized stepped-wedge design. For the optimized stepped-wedge design, the expected power exceeds 88% for all possible values of the ICC. As the ICC increases, power first declines, then increases. This reflects that the source of power in a stepped-wedge design shifts from across-site comparisons to within-site comparisons as ICC increases. Note that the power for a similar standard stepped-wedge design was 3% to 8% lower compared to the optimized stepped-wedge design, across different ICC values.

Figure 2: Two Power Curves as Functions of ICC

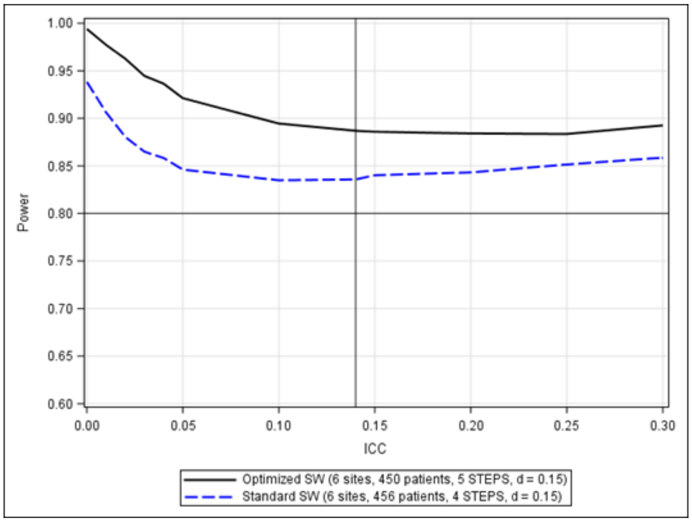

Figure 2: Two power curves as functions of ICC corresponding to the optimized and standard stepped-wedge (SW) design to show non-inferiority of RP to SP, assuming a 15% treatment effect (d = 0.15). The non-inferiority margin is 10%, operationalized here as an odds ratio of 0.67. In other words, we reject the null of inferiority of RP to SP if the lower 95% confidence limit of the treatment success odds ratio [odds(RP)/odds(SP)] exceeds 0.67.

Table 6: Power for ICC = 0.14 corresponding to the optimized and standard stepped-wedge (SW) design to show non-inferiority of RP to SP, assuming a 15% treatment effect (d = 0.15) and a margin of 10%									
**Non-inferiority SW Design**	**Sites**	**Steps**	**SP Success**	**RP Success**	**Effect Size**	**ICC**	**Power**	**Participants per Site per Time Period**	**Total Sample Size**
Optimized	6	5	0.55	0.70	0.15	0.14	0.887	15	**450**
Standard	6	4	0.55	0.70	0.15	0.14	0.836	19	456

SP = Standard Procedure; RP = Rapid Procedure; SW – Stepped – wedge; ICC – intra class correlation coefficient.

Power in Unforeseen Circumstances

As remarked above, for the parameters chosen, power is expected to be at least 88%, irrespective of the true ICC. However, there might be unforeseen circumstances, such as sites failing to enroll or rates of enrollment to be lower than anticipated. Figure 3 explores the impact upon power if a site drops or if enrollment is 10% lower than expected. Figure 3 suggests that for this study design, losing site 3 costs more than losing site 1 or 5, or having a 10% less enrollment, in terms of power loss. However, there is still reasonable power (79% or more) to show non-inferiority of RP to SP regardless of which site drops or if enrollment is 10% lower, and irrespective of the true ICC. For ICC = 0.14, the power to show RP non-inferior to SP is 80% or more regardless of which site drops or if the enrollment is 10% lower.

In addition, loss of power was explored when the true treatment effect is smaller than anticipated. Figure 4 suggests that even for a smaller treatment effect of 13% there is still 80% power or more to show RP is non-inferior to SP, irrespective of the ICC values; for a treatment effect of 12% there is 78% power or more, irrespective of the ICC values; however, for a treatment effect smaller than 11% there will not be enough power to show non-inferiority of RP to SP, for larger values of ICC. If the treatment effect is 11% or 10%, the power drops to 75% and 71%, respectively, for ICC=0.14 (or even lower for higher ICC values).

Figure 3: Power curves as functions of ICC corresponding to the optimized stepped-wedge (SW) design to show non-inferiority of RP to SP, assuming a 15% treatment effect (d = 0.15) and a 10% margin when a site drops or when enrollment rates are lower than anticipated

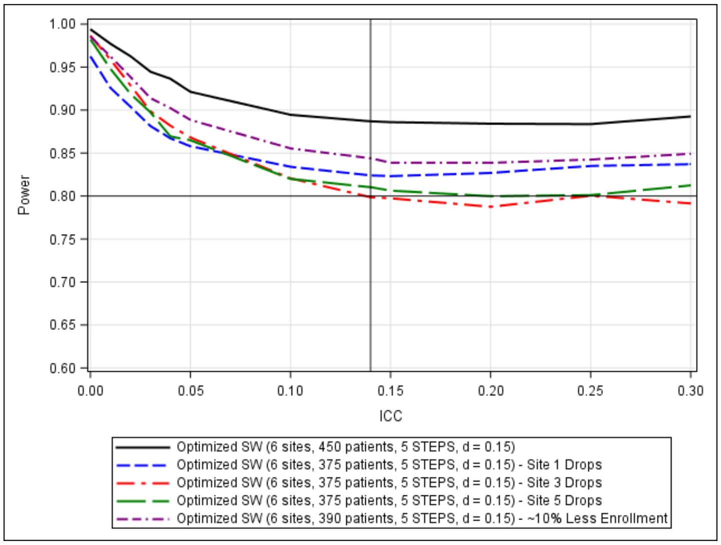

Table 7: Power for ICC = 0.14 for the optimized stepped-wedge (SW) design to show non-inferiority of RP to SP, assuming a 15% treatment effect (d = 0.15) and a 10% margin when a site drops or when enrollment rates are lower than anticipated

Non-inferiority Optimized SW Design	Sites	Steps	Participants per Site per Step	Total Sample Size	ICC	SP Success	RP Success	Effect Size	Power
No Drop-out	6	5	15	450	0.14	0.55	0.7	0.15	0.887
Site 1 Drops	5	5	15	375	0.14	0.55	0.7	0.15	0.824
Site 3 Drops	5	5	15	375	0.14	0.55	0.7	0.15	0.799
Site 5 Drops	5	5	15	375	0.14	0.55	0.7	0.15	0.810
~10% Less Enrollment	6	5	13	390	0.14	0.55	0.7	0.15	0.844

SP = Standard Procedure; RP = Rapid Procedure; SW – Stepped – wedge; ICC – intra class correlation coefficient.

Figure 4: Power curves as functions of ICC corresponding to the optimized stepped-wedge (SW) design to show non-inferiority of RP to SP with a 10% margin, assuming different treatment effects between 15% and 10%

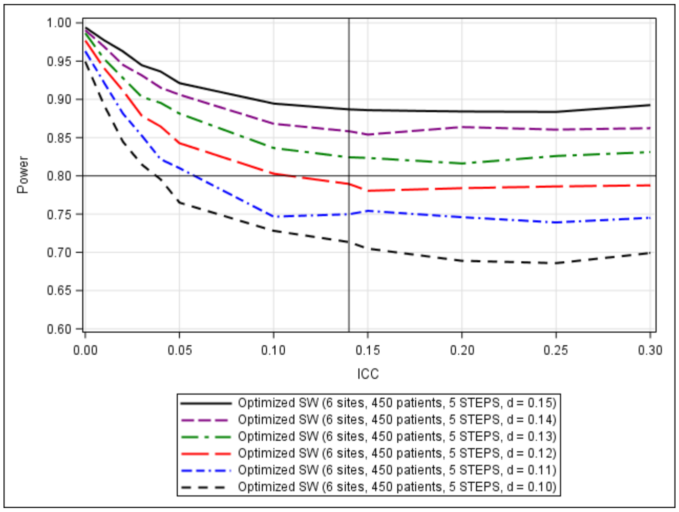

Table 8: Power for ICC = 0.14 corresponding to the optimized stepped-wedge (SW) design to show non-inferiority of RP to SP with a 10% margin, assuming different treatment effects between 15% and 10%

Non-inferiority Optimized SW Design	Sites	Steps	Participants per Site per Step	Total Sample Size	ICC	SP Success	RP Success	Effect Size	Power
Optimized SW	6	5	15	450	0.14	0.55	0.70	0.15	0.887
Optimized SW	6	5	15	450	0.14	0.55	0.69	0.14	0.858
Optimized SW	6	5	15	450	0.14	0.55	0.68	0.13	0.824
Optimized SW	6	5	15	450	0.14	0.55	0.67	0.12	0.789
Optimized SW	6	5	15	450	0.14	0.55	0.66	0.11	0.750
Optimized SW	6	5	15	450	0.14	0.55	0.65	0.10	0.713

SP = Standard Procedure; RP = Rapid Procedure; SW – Stepped – wedge; ICC – intra class correlation coefficient.

Figure 5: Non-inferiority margin as a difference in proportions versus non-inferiority margin as an odds ratio

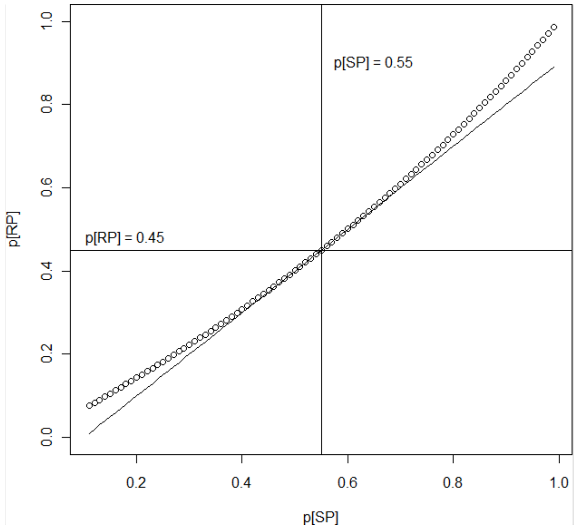

In Figure 5, the vertical and horizontal reference lines represent the proportion of successes in the RP (p[RP] = 0.45) and proportion of successes in the SP (p[SP] = 0.55) used to calculate the margin of non-inferiority in terms of odds ratios [OR=0.67]. The straight line represents the margin of non-inferiority as a difference in proportions i.e., the line p[RP]-p[SP] = -10%. The curved line represents the margin of non-inferiority as an odds ratio i.e. odds[RP]/odds[SP] = 0.67. The graph suggests that the margin of non-inferiority as a difference in proportions versus odds ratio, and in general, corresponds to similar probabilities of success except for extreme values of p[SP] and p[RP] (i.e., less than 0.3 or greater than 0.7).

Conclusion

The optimized stepped-wedge design, with 6 sites (clusters) and 5 periods of time (steps), enrolling a total of 450 participants (15 participants per cluster per time period), assuming 0.55 probability of success in the SP and 0.70 in the RP and a non-inferiority margin of 10%, will provide 88% or more power to show non-inferiority of RP to SP. For the sample size, and probabilities of success hypothesized for CTN-0097, power to show RP is non-inferior to SP is at least 88%, irrespective of the value of the ICC. In addition, the study is adequately powered to account for simulated site drop-out, lower rates of enrollment or for slightly smaller true treatment effects.

13.0 INTERIM ANALYSES AND DATA MONITORING

No interim analyses for efficacy or futility are planned for this study. A stepped-wedge design requires all steps to be completed to estimate the treatment effect.

13.1 Safety Interim Analyses

Safety interim reports will be prepared for the regular Data and Safety Monitoring Board (DSMB) meetings. This will include analysis of adverse events and narrative report on serious adverse events.

14.0 DATA QUALITY

14.1 Data Audits

A summary of data audit results from site interim monitoring visits conducted by CCC monitors will be presented by site, including total fields audited, total data discrepancies, and error rate.

14.2 Protocol Deviations

Protocol deviations will be summarized by site and will include the number of deviations reported, the number of participants each deviation affects, frequencies for the types of protocol deviations, and information on whether the protocol deviation was deemed minor or major. A detailed listing of protocol deviations by deviation category will be provided. The listing will include site, participant ID, date of protocol deviation, date protocol deviation entered in EDC (Electronic Data Capture), deviation type, reason for protocol deviation, relatedness to COVID-19, deviation description, corrective action to be taken, plan to prevent recurrence, IRB reporting required, IRB notification at continuing review, and planned or actual IRB report date.

15.0 SOFTWARE TO BE USED FOR ANALYSES

All analyses performed by the DSC will use SAS® Version 9.4 software.

16.0 UPDATES TO THE STATISTICAL ANALYSIS PLAN

Table 9: SAP Revision History

SAP Version	Date of Approval	Summary of Changes
1.0	20-DEC-2022	Initial Version
2.0	13-APR-2023	Clarified description of exploratory outcome #7 in Section 2.1.3.<br>Revised Section 5.0 to add summary table of baseline characteristics in study completers and clarify description of summary tables by site and induction procedure.

		Revised definition of exploratory outcome #2 in Section 8.6.<br>Added shells for tables, listing and figures in Appendix in Section 19.1.

17.0 REFERENCES

1. Barker, D., McElduff, P., D'Este, C., & Campbell, M. J. (2016). Stepped wedge cluster randomised trials: a review of the statistical methodology used and available. BMC medical research methodology, 16(1), 1-19.

2. Committee for Proprietary Medicinal Products (CPMP). (2001). Points to consider on switching between superiority and non-inferiority. British journal of clinical pharmacology, 52(3), 223.

3. Damschroder, L. J. & Hagedorn, H. J. (2011). A guiding framework and approach for implementation research in substance use disorders treatment. Psychol Addict Behav 25, 194-205.

4. Hemming, K., Taljaard, M., & Forbes, A. (2017). Analysis of cluster randomised stepped wedge trials with repeated cross-sectional samples. Trials, 18(1), 1-11.

5. Hussey MA, Hughes JP. Design and analysis of stepped wedge cluster randomized trials. Contemporary clinical trials. 2007;28(2):182-191.

6. Lee, J. D., Nunes Jr, E. V., Novo, P., Bachrach, K., Bailey, G. L., Bhatt, S., & Rotrosen, J. (2018). Comparative effectiveness of extended-release naltrexone versus buprenorphine-naloxone for opioid relapse prevention (X: BOT): a multicentre, open-label, randomised controlled trial. The Lancet, 391(10118), 309-318.

7. Moher D, Hopewell S, Schulz KF, Montori V, Gøtzsche PC, Devereaux PJ, Elbourne D, Egger M, Altman DG. (2010). CONSORT 2010 explanation and elaboration: updated guidelines for reporting parallel group randomised trials. J Clin Epi, 63(8): e1-e37.

8. National Institutes of Health (NIH). (2016). Guidelines for the Review of Inclusion on the Basis of Sex/Gender, Race, Ethnicity, and Age in Clinical Research.

   https://grants.nih.gov/grants/peer/guidelines_general/Review_Human_subjects_Inclusion.pdf

9. Parzen, M. (2009). Random effects model for simulating clustered binary data. submitted to Statistics and Computing.

10. Sullivan, M., Bisaga, A., Pavlicova, M., Choi, C. J., Mishlen, K., Carpenter, K. M., Levin, F. R., Dakwar, E., Mariani, J. J. & Nunes, E. V. (2017). Long-Acting Injectable Naltrexone Induction: A Randomized Trial of Outpatient Opioid Detoxification With Naltrexone Versus Buprenorphine. Am J Psychiatry 174, 459-467.

11. Thompson, J. A., Fielding, K., Hargreaves, J. & Copas, A. (2017). The optimal design of stepped wedge trials with equal allocation to sequences and a comparison to other trial designs. Clin Trials 14, 639-647.

18.0 LIST OF PROPOSED TABLES, FIGURES, AND LISTINGS

The below listing contains the tables, figures, and listings which will be provided by the DSC.

Section	Title	Population
Enrollment, Participant Disposition, and Follow-up	Summary of Pre-screens by Site	Pre-screened
	Summary of Pre-screens by Step	Pre-screened
	Summary of Screen Failures by Site	Screened
	Summary of Screen Failures by Step	Screened
	Summary of Pre-screens, Screens, and Enrollment by Site and Step	Pre-screened
	Summary of Pre-screens, Screens, and Enrollment by Site	Pre-screened
	Enrollments by Site and Step Based on Expected Recruitment Rate	ITT
	Enrollments by Site and Induction Procedure	ITT
	Proposed and Actual Enrollments by Site	ITT
	Figure of Expected versus Actual Enrollments Overall	ITT
	Figure of Expected versus Actual Enrollments by Site	ITT
	Summary of Participant Disposition by Site	ITT
	Summary of Participant Disposition by Induction Procedure	ITT
	CONSORT Diagram	ITT
	Summary of Attendance at Post Induction Visits by Site	ITT
	Summary of Attendance at Post Induction Visits by Induction Procedure	ITT
	Summary of Missed Visits by Site	ITT
	Summary of Missed Visits by Induction Procedure	ITT
Participant Characteristics at Baseline	Summary of Baseline Characteristics by Site	ITT
	Summary of Baseline Characteristics by Induction Procedure	ITT
	Summary of Baseline Characteristics in Study Completers by Induction Procedure	Study Completers

Section	Title	Population
Medication Administered During Induction Phase	Summary of Daily Medications Administered by Induction Procedure	ITT
	Summary of Daily Medications Administered in Standard Procedure by Inpatient Day	ITT
	Summary of Daily Medications Administered in Rapid Procedure by Inpatient Day	ITT
	Summary of Daily Medications Administered to Participants Who Initiated Buprenorphine in Standard Procedure by Procedure Phase	ITT
	Summary of Daily Medications Administered to Participants Who Initiated Buprenorphine in Standard Procedure by Procedure Phase	ITT
Early Induction Terminations	Summary of Early Induction Terminations by Site	ITT
	Summary of Early Induction Terminations by Induction Procedure	ITT
Treatment Exposure	Summary of Treatment Exposure by Site	ITT
	Summary of Treatment Exposure by Induction Procedure	ITT
	Summary of XR-NTX Injections in Induction Failure Participants by Site	ITT
	Summary of XR-NTX Injections in Induction Failure Participants by Induction Procedure	ITT
Primary Outcome Analyses	Summary of Primary Outcome by Induction Procedure	ITT
	Summary of Primary Outcome by Site	ITT
	Summary of Primary Outcome by Step	ITT
	Summary of Primary Outcome by Site and Step	ITT

Section	Title	Population
Supportive Analyses of the Primary Outcomes	Summary of Primary Outcome by Sex and Induction Procedure	ITT
	Summary of Primary Outcome by Age and induction Procedure	ITT
	Summary of Primary Outcome by Race and Induction Procedure	ITT
	Summary of Primary Outcome by Ethnicity and Induction Procedure	ITT
	Forest Plot of Odds Ratios by Sub-groups	ITT
	Summary of Primary Outcome Excluding Participants Admitted Four Calendar Days Prior to Enrollment by Induction Procedure	ITT
	Summary of Primary Outcome Excluding Participants Enrolled During Pre-Implementation Phase by Induction Procedure	ITT
	Summary of Primary Outcome Including Term for Baseline Fentanyl Use	ITT
	Summary of Primary Outcome by Induction Procedure in Participants Who Initiated Buprenorphine.	ITT
Key Secondary Outcomes	Summary of Days to First XR-NTX Injection by Induction Procedure	Inducted
	Summary of Average Daily Opioid Withdrawal and Craving Scores by Induction Procedure	ITT
	Figure of Average Daily Maximum COWS Score by Induction Procedure	ITT
	Figure of Average Daily COWS Score by Induction Procedure	ITT
	Figure of Average Daily SOWS Score by Induction Procedure	ITT
	Figure of Average Daily VAS Craving Score at Time of Assessment by Induction Procedure	ITT
	Figure of Average Daily Maximum VAS Craving Score within 24 Hours by Induction Procedure	ITT
	Summary of Average Opioid Withdrawal and Craving Scores by Procedure Phase and Induction Procedure	ITT

Section	Title	Population
	Figure of Average Daily Maximum COWS Score by Induction Procedure and Phase	ITT
	Figure of Average Daily COWS Score by Induction Procedure and Phase	ITT
	Figure of Average Daily SOWS Score by Induction Procedure and Phase	ITT
	Figure of Average VAS Craving Score at Time of Assessment by Induction Procedure and Phase	ITT
	Figure of Average Maximum VAS Craving Score within 24 Hours by Induction Procedure and Phase	ITT
	Summary of Average Opioid Withdrawal and Craving in Standard Procedure by Procedure Phase	ITT
	Figure of Average Daily Maximum COWS Score in Standard Procedure by Phase	ITT
	Figure of Average Daily COWS Score in Standard Procedure by Phase	ITT
	Figure of Average Daily SOWS Score in Standard Procedure by Phase	ITT
	Figure of Average VAS Craving Score at time of Assessment 24 in Standard Procedure by Phase	ITT
	Figure of Average Maximum VAS Craving Score within 24 Hours in Standard Procedure by Phase	ITT
	Summary of Average Opioid Withdrawal and Craving in Rapid Procedure by Procedure Phase	ITT
	Figure of Average Daily Maximum COWS Score in Rapid Procedure by Phase	ITT
	Figure of Average Daily COWS Score in Rapid Procedure by Phase	ITT
	Figure of Average Daily SOWS Score in Rapid Procedure by Phase	ITT
	Figure of Average VAS Craving Score at Time of Assessment in Rapid Procedure by Phase	ITT
	Figure of Average Maximum VAS Craving Score within 24 Hours in Rapid Procedure by Phase	ITT

Section	Title	Population
	Covariate Adjusted Modeling Results for Opioid Withdrawal as Measured by COWS and SOWS During the Induction Phase	ITT
	Covariate Adjusted Modeling Results for Craving for Opioids During the Induction Phase	ITT
	Retention in the Study to Receive at Least One XR-NTX Injection after Induction Success	Inducted
Safety	Summary of Targeted Safety Events by Induction Procedure	Safety
	Summary of Serious Adverse Events by Induction Procedure	Safety
	Summary of MedDRA-coded Serious Adverse Events by Induction Procedure	Safety
	Listing of Deaths by Induction Procedure	Safety
	Summary of Study Injection Site Examinations	Safety
	Summary of Non-Fatal Opioid Overdoses by Study Phase and Induction Procedure	Safety
	Summary of Suicide Risk by Induction Procedure	Safety
	Listing of Serious Adverse Events by Induction Procedure	Safety
	Listing of Targeted Safety Events by Induction Procedure	Safety
	Listing of Injection Site Abnormalities by Induction Procedure	Safety
	Listing of Non-Fatal Opioid Overdoses by Induction Procedure	Safety
	Listing of Suicide Risk by Induction Procedure	Safety
	Listing of Pregnancies by Induction Procedure	Safety
Data Quality	Summary of Data Audits by Site	N/A
	Summary of Protocol Deviations by Site	N/A
	Listing of Protocol Deviations	N/A

19.0 APPENDICES

19.1 SHELLS FOR PROPOSED TABLES, FIGURES AND LISTINGS

19.1.1 Enrollment, Participant Disposition, and Visit Attendance

Table 10: Summary of Pre-screening by Site

	Gibson Recovery Center	Nexus Recovery Center	Stony Brook Eastern Long Island Hospital	Aspire Health Partners	Avery Road Treatment Center	ADAPT	Total
Number pre-screened	N						
Number of pre-screen failures	N (X.x%)						
Criterion resulting in ineligibility[1]							
No current and active OUD	N (X.x%)						
Not eligible for XR-NTX							
Not attempting XR-NTX induction							
Not satisfying basic eligibility to move forward in the study							

[1] Percentages were calculated based on the denominator of the number of pre-screens failures.

Table 11: Summary of Pre-screening by Step						
	**Step 1**	**Step 2**	**Step 3**	**Step 4**	**Step 5**	**Total**
Number pre-screened	N					
Number of pre-screen failures	N (X.x%)					
Criterion resulting in ineligibility[1]						
No current and active OUD	N (X.x%)					
Not eligible for XR-NTX						
Not attempting XR-NTX induction						
Not satisfying basic eligibility to move forward in the study						

[1] Percentages were calculated based on the denominator of the number of pre-screens failures.

Table 12: Summary of Screen Failures by Site

	Gibson Recovery Center	Nexus Recovery Center	Stony Brook Eastern Long Island Hospital	Aspire Health Partners	Avery Road Treatment Center	ADAPT	Total
Number screened	N						
Number of ineligible screens	N (X.x%)						
Did not meet the following eligibility criteria[1]							
Inclusion criteria							
18 years or older	N (X.x%)						
DSM-5 criteria for current opioid use disorder							
Seeking treatment for OUD, willing to accept XR-NTX, and a good candidate for naltrexone based treatment							
Willing and able to provider written informed consent							
Able to speak English sufficiently to understand study procedures and provide written informed consent							
If of childbearing potential, willing to practice effective birth control method during the study							
Exclusion criteria							
Has a serious medical, psychiatric or substance use disorder that would make detox and naltrexone initiation or maintenance treatment with XR-NTX hazardous	N (X.x%)						
Known allergy or sensitivity to buprenorphine, naloxone, naltrexone, polylactide-co-glycolide, carboxmethylcellulose or other components of the Vivitrol diluent							
On maintenance treatment with methadone							
On maintenance treatment with buprenorphine unless the patient is determined to have a poor treatment response warranting change to XR-NTX							
Experiencing the presence of pain of sufficient severity as to require ongoing pain management with opioids							

Table 12: Summary of Screen Failures by Site							
	**Gibson Recovery Center**	**Nexus Recovery Center**	**Stony Brook Eastern Long Island Hospital**	**Aspire Health Partners**	**Avery Road Treatment Center**	**ADAPT**	**Total**
Experiencing circumstances (legal, personal, occupational) that would threaten the feasibility of XR-NTX and make another treatment a better choice							
Currently in jail, prison or other overnight facility as required by law or have pending legal action that could prevent participation							
If female, currently pregnant or breastfeeding or planning on conception							
Body habitus that precludes safe intramuscular injection of XR-NTX							
Admitted to the inpatient detox or rehabilitation unit more than 4 calendar days prior to consent							
Other reasons for screen failure[1]							
Withdrew consent	N (X.x%)						
Left hospital (AMA or discharged) prior to completing screening							
Number of participants eligible but not enrolled	N (X.x%)						
Reasons for not being enrolled[2]							
No longer interested in participating in the study	N (X.x%)						
Judgment of site/research staff							
Time commitment							
Left prior to completion							
COVID-19: Illness							
COVID-19: Public health measures							
COVID-19: Other							
Other							

[1] Percentages were calculated based on the denominator of the number of ineligible screens and may exceed 100% if multiple eligibility criteria were not met for potential participants.
[2] Percentages were calculated based on the denominator of the number of participants eligible but not enrolled.

Table 13: Summary of Screen Failures by Step						
	**Step 1**	**Step 2**	**Step 3**	**Step 4**	**Step 5**	**Total**
Number screened	N					
Number of ineligible screens	N (X.x%)					
Did not meet the following eligibility criteria[1]						
Inclusion criteria						
18 years or older	N (X.x%)					
DSM-5 criteria for current opioid use disorder						
Seeking treatment for OUD, willing to accept XR-NTX, and a good candidate for naltrexone based treatment						
Willing and able to provider written informed consent						
Able to speak English sufficiently to understand study procedures and provide written informed consent						
If of childbearing potential, willing to practice effective birth control method during the study						
Exclusion criteria						
Has a serious medical, psychiatric or substance use disorder that would make detox and naltrexone initiation or maintenance treatment with XR-NTX hazardous	N (X.x%)					
Known allergy or sensitivity to buprenorphine, naloxone, naltrexone, polylactide-co-glycolide, carboxmethylcellulose or other components of the Vivitrol diluent						
On maintenance treatment with methadone						
On maintenance treatment with buprenorphine unless the patient is determined to have a poor treatment response warranting change to XR-NTX						
Experiencing the presence of pain of sufficient severity as to require ongoing pain management with opioids						

Table 13: Summary of Screen Failures by Step						
	**Step 1**	**Step 2**	**Step 3**	**Step 4**	**Step 5**	**Total**
Experiencing circumstances (legal, personal, occupational) that would threaten the feasibility of XR-NTX and make another treatment a better choice						
Currently in jail, prison or other overnight facility as required by law or have pending legal action that could prevent participation						
If female, currently pregnant or breastfeeding or planning on conception						
Body habitus that precludes safe intramuscular injection of XR-NTX						
Admitted to the inpatient detox or rehabilitation unit more than 4 calendar days prior to consent						
Other reasons for screen failure[1]	N (X.x%)					
Withdrew consent						
Left hospital (AMA or discharged) prior to completing screening						
Number of participants eligible but not enrolled	N (X.x%)					
Reasons for not being enrolled[2]						
No longer interested in participating in the study	N (X.x%)					
Judgment of site/research staff						
Time commitment						
Left prior to completion						
COVID-19: Illness						
COVID-19: Public health measures						
COVID-19: Other						
Other						

[1] Percentages were calculated based on the denominator of the number of ineligible screens and may exceed 100% if multiple eligibility criteria were not met for potential participants.
[2] Percentages were calculated based on the denominator of the number of participants eligible but not enrolled.

Table 14: Summary of Pre-screens, Screens, and Enrollment by Site and by Step							
**Site**		**Step 1**	**Step 2**	**Step 3**	**Step 4**	**Step 5**	**Total**
Gibson Recovery Center	Pre-screened	N					
	Screened	N					
	Enrolled	N					
Nexus Recovery Center	Pre-screened						
	Screened						
	Enrolled						
Stony Brook Eastern Long Island Hospital	Pre-screened						
	Screened						
	Enrolled						
Aspire Health Partners	Pre-screened						
	Screened						
	Enrolled						
Avery Road Treatment Center	Pre-screened						
	Screened						
	Enrolled						
ADAPT	Pre-screened						
	Screened						
	Enrolled						
Total	Pre-screened						
	Screened						
	Enrolled						

Table 15: Summary of Pre-screens, Screens, and Enrollment by Site

Site	Number of Pre-screens	Number of Screens	Percent of Eligible Pre-screens Screened	Number of Screen Fails	Percent of Screens who Screen Fail	Number Eligible but Not Enrolled	Number in Screening	Number Enrolled	Percent of Eligible Pre-screens Enrolled	Percent of Screens Enrolled
Gibson Recovery Center	N	N	X.x%	N	X.x%	N	N	N	X.x%	X.x%
Nexus Recovery Center										
Stony Brook Eastern Long Island Hospital										
Aspire Health Partners										
Avery Road Treatment Center										
ADAPT										
Total										

Table 16: Enrollments by Site and Step Based on Expected[1] Recruitment Rate

Site	Step 1	Step 2	Step 3	Step 4	Step 5	Total
Gibson Recovery Center	n/15 (X.x%)	n/15 (X.x%)	n/15 (X.x%)	n/15 (X.x%)	n/15 (X.x%)	n/75 (X.x%)
Nexus Recovery Center						
Stony Brook Eastern Long Island Hospital						
Aspire Health Partners						
Avery Road Treatment Center						
ADAPT						
Total	n/90 (X.x%)	n/90 (X.x%)	n/90 (X.x%)	n/90 (X.x%)	n/90 (X.x%)	n/450 (X.x%)

[1] The expected enrollment for each step and site was 15 participants.
Grey highlighted cells for each site and step indicate number enrolled in the Rapid Procedure and white cells indicate number enrolled in the Standard Procedure.

Table 17: Enrollments by Site and Induction Procedure

	Induction Procedure		
**Site**	**Standard (N=XX)**	**Rapid (N=XX)**	**Total (N=XX)**
Gibson Recovery Center	N (X.x%)		
Nexus Recovery Center			
Stony Brook Eastern Long Island Hospital			
Aspire Health Partners			
Avery Road Treatment Center			
ADAPT			
Total			

Table 18: Proposed and Actual Enrollments by Site

**Site**	**Proposed Enrollments**	**Date Site Opened for Enrollment**	**Date of First Enrollment**	**Actual Enrollments**	**Actual/ Proposed (%)**	**Date of Last Enrollment**
Gibson Recovery Center	N	dd/mm/yyyy	dd/mm/yyyy	N	X.x%	dd/mm/yyy
Nexus Recovery Center						
Stony Brook Eastern Long Island Hospital						
Aspire Health Partners						
Avery Road Treatment Center						
ADAPT						
Total						

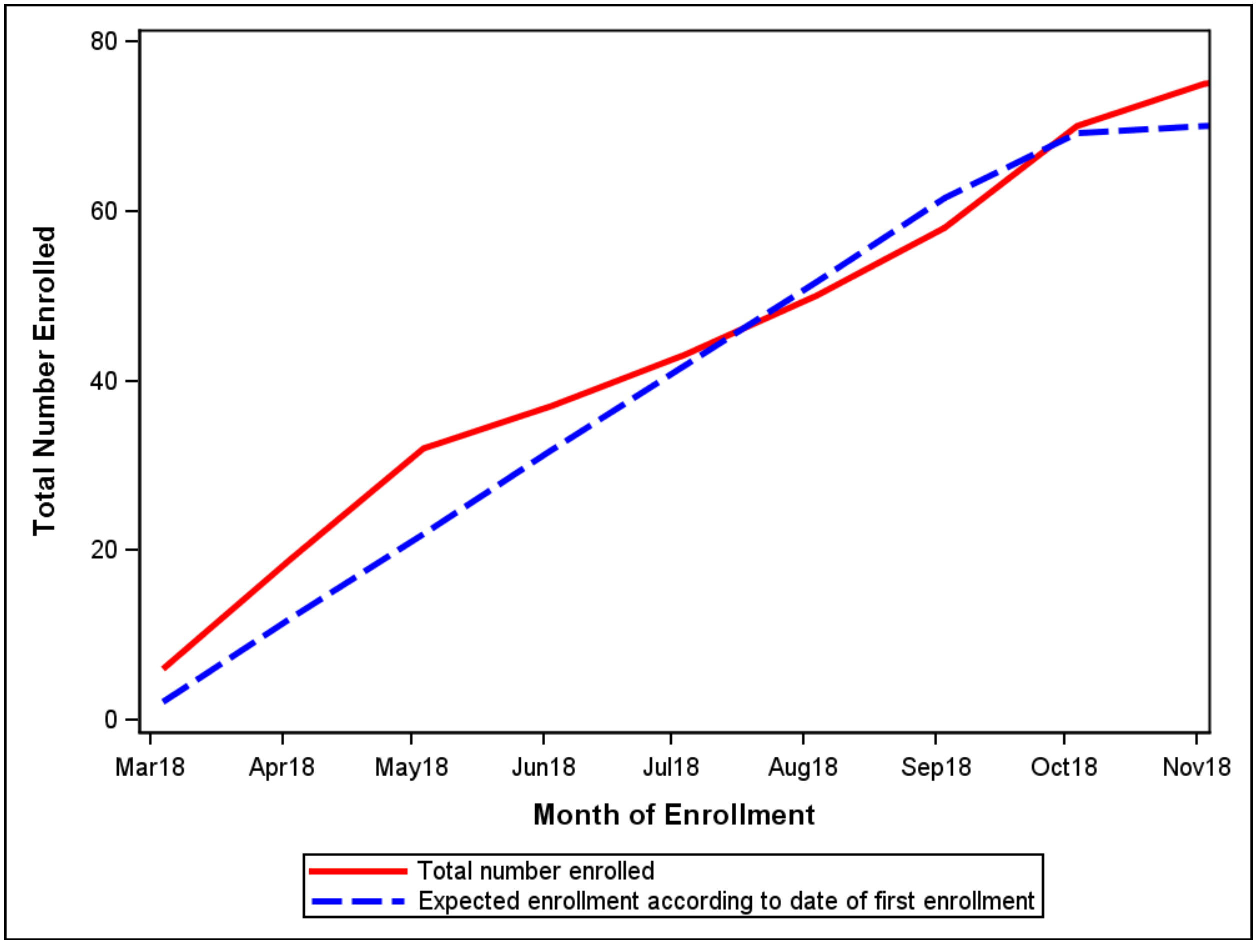

Figure 6: Expected versus Actual Enrollments Overall

Example figure provided.

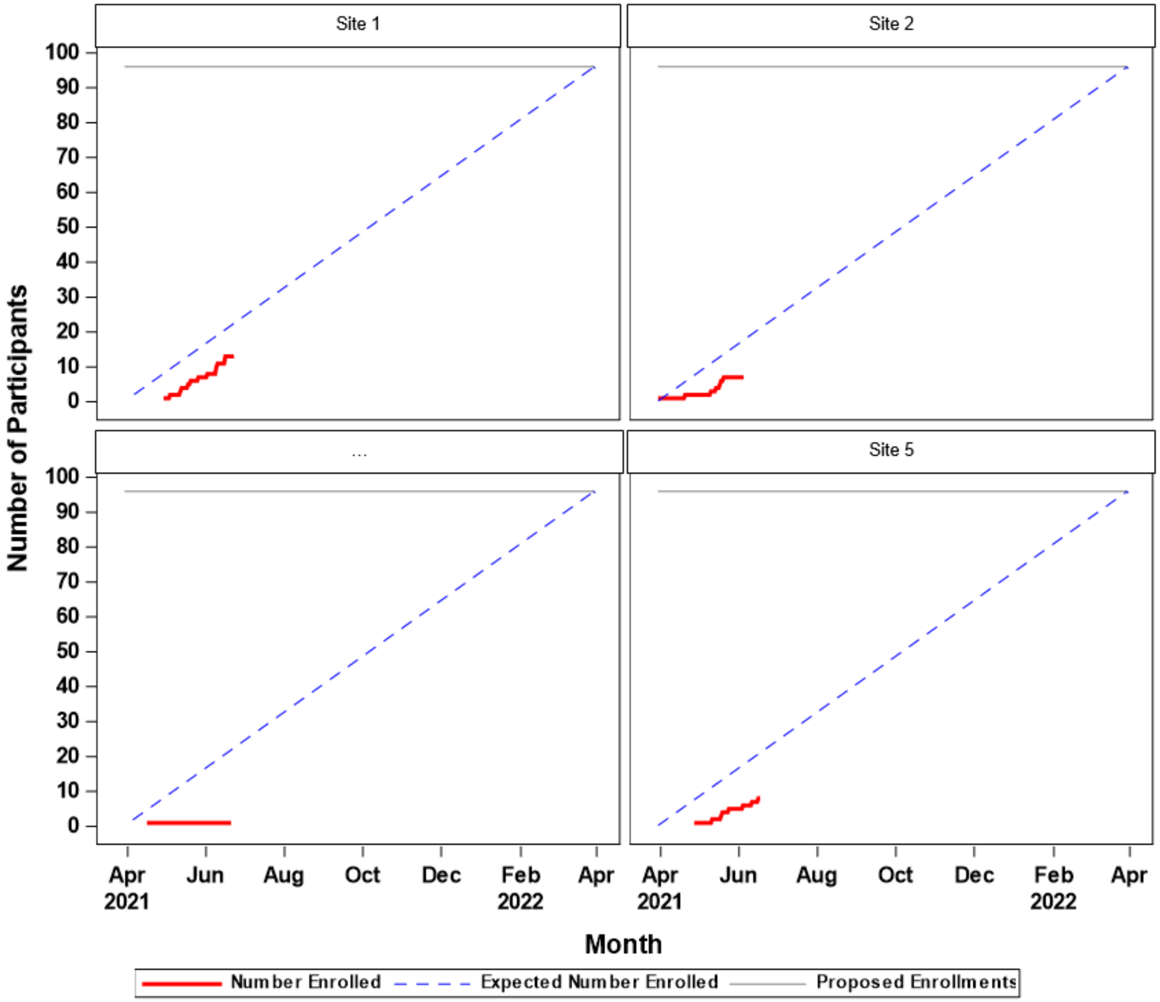

Figure 7: Expected versus Actual Enrollments by Site

Example figure provided.

Table 10: Summary of Participant Disposition by Site							
	Gibson Recovery Center	Nexus Recovery Center	Stony Brook Eastern Long Island Hospital	Aspire Health Partners	Avery Road Treatment Center	ADAPT	Total
Number of participants enrolled	N						
Number of study completers[1]	N (X.x%)						
Number of early study terminations[2]	N (X.x%)						
Reasons for early study termination[3]							
Participant failed to return to clinic and unable to contact	N (X.x%)						
Participant stopped participation due to practical problems (e.g., no childcare or transportation)							
Participant moved from area							
Participant terminated due to AE/SAE							
Participant terminated for other clinical reasons							
Participant deceased							
Participant terminated for administrative issues							
Site closed							
Participant uncomfortable answering questions							
Research staff unable to complete interview (unrelated to participant)							
Technical difficulties (unrelated to participant)							
Participant was ineligible and should not have been enrolled in study							
Unable to contact participant							
Participant incarcerated and unable to complete assessments							
Participant no longer wishes to complete assessments due to time involved and inconvenience							

Table 10: Summary of Participant Disposition by Site

	Gibson Recovery Center	Nexus Recovery Center	Stony Brook Eastern Long Island Hospital	Aspire Health Partners	Avery Road Treatment Center	ADAPT	Total
Participant withdrew consent/assent for other reasons							
Participant in hospital, in-patient or residential treatment and not available for assessment							
Participant terminated due to COVID-19: Illness							
Participant terminated due to COVID-19: Public health measures							
Participant terminated due to COVID-19: Other							
Participant terminated for other reason							

[1] Participants were defined as study completers if they had a completed STC form indicating study completion.
[2] Participants were defined as early terminations if they had a completed STC form indicating early study termination.
[3] The percentage was calculated with the denominator as number of early study terminations.

Table 11: Summary of Disposition by Induction Procedure

	Induction Procedure		
	**Standard**	**Rapid**	**Total**
Number of participants enrolled	N		
Number of study completers[1]	N (X.x%)		
Number of early study terminations[2]	N (X.x%)		
Reasons for early study termination[3]			
Participant failed to return to clinic and unable to contact	N (X.x%)		
Participant stopped participation due to practical problems (e.g., no childcare or transportation)			
Participant moved from area			
Participant terminated due to AE/SAE			
Participant terminated for other clinical reasons			
Participant deceased			
Participant terminated for administrative issues			
Site closed			
Participant uncomfortable answering questions			
Research staff unable to complete interview (unrelated to participant)			
Technical difficulties (unrelated to participant)			
Participant was ineligible and should not have been enrolled in study			
Unable to contact participant			
Participant incarcerated and unable to complete assessments			
Participant no longer wishes to complete assessments due to time involved and inconvenience			
Participant withdrew consent/assent for other reasons			

Table 11: Summary of Disposition by Induction Procedure			
	**Induction Procedure**		
	**Standard**	**Rapid**	**Total**
Participant in hospital, in-patient or residential treatment and not available for assessment			
Participant terminated due to COVID-19: Illness			
Participant terminated due to COVID-19: Public health measures			
Participant terminated due to COVID-19: Other			
Participant terminated for other reason			

[1] Participants were defined as study completers if they had a completed STC form indicating study completion.
[2] Participants were defined as early terminations if they had a completed STC form indicating early study termination.
[3] The percentage was calculated with the denominator as number of early study terminations.

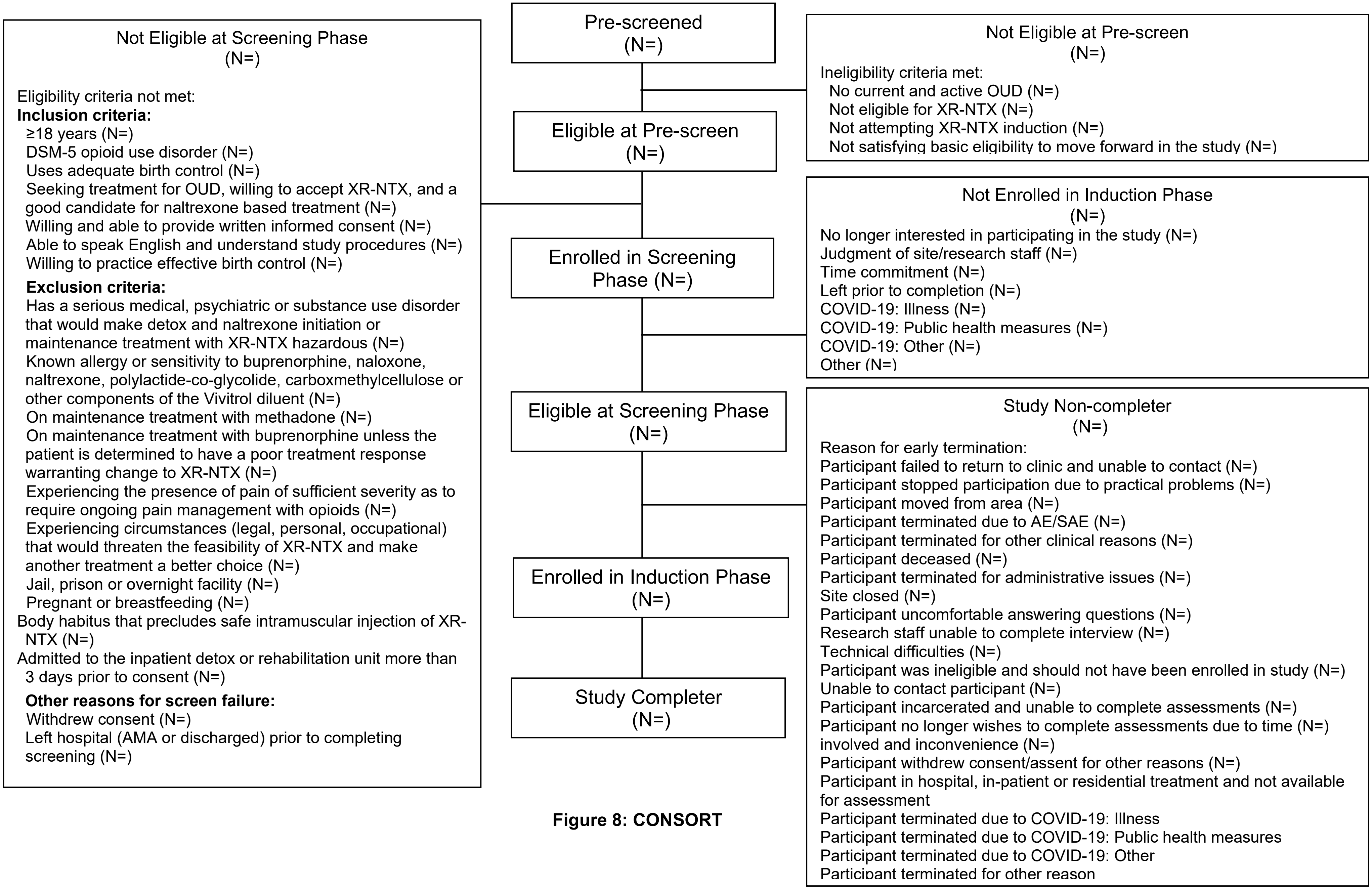

Figure 8: CONSORT

Table 12: Summary of Attendance at Post-Induction Visits by Site

Visit	Gibson Recovery Center (N=XX)	Nexus Recovery Center (N=XX)	Stony Brook Eastern Long Island Hospital (N=XX)	Aspire Health Partners (N=XX)	Avery Road Treatment Center (N=XX)	ADAPT(N =XX)	Total (N=XX)
28-Day Post-Induction Visit	N (X.x%)						
56-Day Post-Induction Visit							
Total[1]							

[1] Percentages were calculated with the denominator as the number of expected visits at both post-induction Day 28 and Day 56 visits.

Table 13: Summary of Attendance at Post-Induction Visits by Induction Procedure

	Induction Procedure		
Visit	Standard (N=XX)	Rapid (N=XX)	Total (N=XX)
28-Day Post-Induction Visit	N (X.x%)		
56-Day Post-Induction Visit			
Total[1]			

[1] Percentages were calculated with the denominator as the number of expected visits at both post-induction Day 28 and Day 56 visits.

Table 14: Summary of Missed Visits by Site

	Gibson Recovery Center (N=XX)	Nexus Recovery Center (N=XX)	Stony Brook Eastern Long Island Hospital (N=XX)	Aspire Health Partners (N=XX)	Avery Road Treatment Center (N=XX)	ADAPT (N=XX)	Total (N=XX)
Number of expected visits[1]	N						
Number of missed visits[2]	N (X.x%)						
Number of participants with at least one missed visit[3]	N (X.x%)						
Average number of missed visits per participant[4]	X.x						
Reason for missed visit[5]							
Participant on vacation	N (X.x%)						
Participant illness							
Participant in hospital, in-patient, or residential treatment							
Participant moved from the area							
Participant incarcerated							
Site closed							
Participant withdrew consent							
Participant deceased							
Participant unable to attend visit due to logistical barriers							
Participant failed to return to site and unable to contact							
Visit was not scheduled							
Unable to contact							
Site decision/error							
COVID-19: Illness							
COVID-19: Public health measures							
COVID-19: Other							
Other							
Unknown							

[1] Expected visits include post-induction Day 28 and Day 56 visits.
[2] Percentages were calculated based on the denominator of number of expected visits.
[3] Percentages were calculated based on the denominator of total number of participants.
[4] Average number of missed visits per participant with at least one missed visit. The maximum number of missed visits possible was 2 visits.
[5] Percentages were calculated based on the denominator of number of missed visits.

Table 15: Summary of Missed Visits by Induction Procedure			
	**Induction Procedure**		
	**Standard (N=XX)**	**Rapid (N=XX)**	**Total (N=XX)**
Number of expected visits[1]	N		
Number of missed visits[2]	N (X.x%)		
Number of participants with at least one missed visit[3]	N (X.x%)		
Average number of missed visits per participant[4]	X.x		
Reason for missed visit[5]			
Participant on vacation	N (X.x%)		
Participant illness			
Participant in hospital, in-patient, or residential treatment			
Participant moved from the area			
Participant incarcerated			
Site closed			
Participant withdrew consent			
Participant deceased			
Participant unable to attend visit due to logistical barriers			
Participant failed to return to site and unable to contact			
Visit was not scheduled			
Unable to contact			
Site decision/error			
COVID-19: Illness			
COVID-19: Public health measures			
COVID-19: Other			
Other			
Unknown			

[1] Expected visits include post-induction Day 28 and Day 56 visits.
[2] Percentages were calculated based on the denominator of number of expected visits.
[3] Percentages were calculated based on the denominator of total number of participants.
[4] Average number of missed visits per participant with at least one missed visit. The maximum number of missed visits possible was 2 visits.
[5] Percentages were calculated based on the denominator of number of missed visits.

19.1.2 Participant Characteristics at Baseline

Table 16: Summary of Baseline Characteristics by Site

	Gibson Recovery Center (N=XX)	Nexus Recovery Center (N=XX)	Stony Brook Eastern Long Island Hospital (N=XX)	Aspire Health Partners (N=XX)	Avery Road Treatment Center (N=XX)	ADAPT (N=XX)	Total (N=XX)
Sex							
Male	N (X.x%)						
Female							
Don't know							
Refused to answer							
Gender							
Missing	N (X.x%)						
Male							
Female							
Transgender male							
Transgender female							
Non-binary							
Not listed							
Age in years (Mean (SD))	x.x (x.xx)						
Age in years							
< 18	N (X.x%)						
18 - < 25							
25 - < 35							
35 - < 45							
45 - < 55							

Table 16: Summary of Baseline Characteristics by Site							
	**Gibson Recovery Center (N=XX)**	**Nexus Recovery Center (N=XX)**	**Stony Brook Eastern Long Island Hospital (N=XX)**	**Aspire Health Partners (N=XX)**	**Avery Road Treatment Center (N=XX)**	**ADAPT (N=XX)**	**Total (N=XX)**
55 - < 65							
65 - < 75							
75+							
Ethnicity							
Not Hispanic or Latino	N (X.x%)						
Hispanic or Latino							
Don't know							
Refused to answer							
Race							
American Indian or Alaska Native	N (X.x%)						
Asian							
Black or African American							
Native Hawaiian or Pacific Islander							
White							
Other							
Multiracial							
Don't know							
Refused to answer							
Education completed							
Less than high school diploma	N (X.x%)						
High school graduate							
GED or equivalent							

Table 16: Summary of Baseline Characteristics by Site

	Gibson Recovery Center (N=XX)	Nexus Recovery Center (N=XX)	Stony Brook Eastern Long Island Hospital (N=XX)	Aspire Health Partners (N=XX)	Avery Road Treatment Center (N=XX)	ADAPT (N=XX)	Total (N=XX)
Some college, no degree							
Associate's degree: occupational, technical, or vocational program							
Associate's degree: academic program							
Bachelor's degree							
Master's degree							
Professional school degree							
Doctoral degree							
Don't know							
Refused							
Marital status							
Married	N (X.x%)						
Widowed							
Divorced							
Separated							
Never married							
Living with partner							
Don't know							
Refused							
Sexual orientation							
Missing	N (X.x%)						
Heterosexual or straight							

Table 16: Summary of Baseline Characteristics by Site

	Gibson Recovery Center (N=XX)	Nexus Recovery Center (N=XX)	Stony Brook Eastern Long Island Hospital (N=XX)	Aspire Health Partners (N=XX)	Avery Road Treatment Center (N=XX)	ADAPT (N=XX)	Total (N=XX)
Gay or lesbian							
Bisexual							
Queer							
Not sure							
Something else							
Employment							
Working now	N (X.x%)						
Only temporarily laid off, sick leave, or maternity leave							
Looking for work, unemployed							
Retired							
Disabled permanently or temporarily							
Keeping house							
Student							
Other							
Location participant spent the night before coming to the unit							
Missing	N (X.x%)						
Own apartment, room or house - subsidized, for example Section 8 or living in public housing							
Own apartment, room or house - not subsidized							
Someone else's apartment, room or house							

Table 16: Summary of Baseline Characteristics by Site

	Gibson Recovery Center (N=XX)	Nexus Recovery Center (N=XX)	Stony Brook Eastern Long Island Hospital (N=XX)	Aspire Health Partners (N=XX)	Avery Road Treatment Center (N=XX)	ADAPT (N=XX)	Total (N=XX)
Hotel, SRO, or boarding home							
Halfway house, residential treatment program (focus: establishing sobriety)							
Transitional housing (focus: movement into permanent housing)							
Institution (hospital, nursing home, etc.)							
Homeless shelter							
Outdoors/street, abandoned/public building, vehicle, or other place not meant for human habitation							
Detox							
Other - homeless							
Other - stable housing							
Other							
Refused							
First COWS Score on Day 1 of admission							
N	N						
Mean	x.x						
SD	x.xx						
Minimum	x						
25th Percentile	x.x						
Median	x.x						
75th Percentile	x.x						
Maximum	x						

Table 16: Summary of Baseline Characteristics by Site

	Gibson Recovery Center (N=XX)	Nexus Recovery Center (N=XX)	Stony Brook Eastern Long Island Hospital (N=XX)	Aspire Health Partners (N=XX)	Avery Road Treatment Center (N=XX)	ADAPT (N=XX)	Total (N=XX)
History of lifetime opioid overdose	N (X.x%)						
Number of lifetime overdoses							
N	N						
Mean	x.x						
SD	x.xx						
Minimum	x						
25th Percentile	x.x						
Median	x.x						
75th Percentile	x.x						
Maximum	x						
Number of days since last self-reported opioid use at Day 1 of admission							
N	N						
Mean	x.x						
SD	x.xx						
Minimum	x						
25th Percentile	x.x						
Median	x.x						
75th Percentile	x.x						
Maximum	x						
Baseline substance use (Urine Drug Screen)							
Opiates	N (X.x%)						

Table 16: Summary of Baseline Characteristics by Site

	Gibson Recovery Center (N=XX)	Nexus Recovery Center (N=XX)	Stony Brook Eastern Long Island Hospital (N=XX)	Aspire Health Partners (N=XX)	Avery Road Treatment Center (N=XX)	ADAPT (N=XX)	Total (N=XX)
Oxycodone							
Methadone							
Buprenorphine							
Amphetamine							
Barbiturate							
Benzodiazepines							
Marijuana							
Cocaine							
Ecstasy (MDMA)							
Methamphetamine							
Phencyclidine							
Fentanyl							
TLFB substance use (at least once in the past 7 days)							
Heavy alcohol use (Females: 3 or more; Males: 4 or more (drinks per day))	N (X.x%)						
Heroin/Fentanyl							
Opioid analgesics							
Buprenorphine							
Methadone							
No opioids (Heroine/Fentanyl, Opioid Analgesics, Buprenorphine, Methadone)							
Other Amphetamine							

Table 16: Summary of Baseline Characteristics by Site

	Gibson Recovery Center (N=XX)	Nexus Recovery Center (N=XX)	Stony Brook Eastern Long Island Hospital (N=XX)	Aspire Health Partners (N=XX)	Avery Road Treatment Center (N=XX)	ADAPT (N=XX)	Total (N=XX)
Benzodiazepines							
Cannabis							
Cocaine							
Ecstasy (MDMA)							
Methamphetamine							
Inhalant							
Other drugs							
Missing							
TLFB substance use (at least once in the past 30 days)							
Heavy alcohol use (Females: 3 or more; Males: 4 or more (drinks per day))	N (X.x%)						
Heroin/Fentanyl							
Opioid analgesics							
Buprenorphine							
Methadone							
No opioids (Heroin/Fentanyl, Opioid Analgesics, Buprenorphine, Methadone)							
Amphetamine							
Benzodiazepines							
Cannabis							
Cocaine							
Ecstasy (MDMA)							

Table 16: Summary of Baseline Characteristics by Site							
	**Gibson Recovery Center (N=XX)**	**Nexus Recovery Center (N=XX)**	**Stony Brook Eastern Long Island Hospital (N=XX)**	**Aspire Health Partners (N=XX)**	**Avery Road Treatment Center (N=XX)**	**ADAPT (N=XX)**	**Total (N=XX)**
Methamphetamine							
Inhalants							
Other drugs							
Missing							
Route of heroin/fentanyl last use from TLFB at baseline							
No use	N (X.x%)						
Oral							
Nasal							
Smoking							
Injection							
Missing							
Route of prescription opioid last use from TLFB at baseline							
Nasal	N (X.x%)						
Smoking							
Injection							
Route of methadone last use from TLFB at baseline							
Nasal	N (X.x%)						
Injection							
Route of buprenorphine last use from TLFB at baseline							
Nasal	N (X.x%)						

Table 16: Summary of Baseline Characteristics by Site							
	**Gibson Recovery Center (N=XX)**	**Nexus Recovery Center (N=XX)**	**Stony Brook Eastern Long Island Hospital (N=XX)**	**Aspire Health Partners (N=XX)**	**Avery Road Treatment Center (N=XX)**	**ADAPT (N=XX)**	**Total (N=XX)**
Injection							
Substance use disorder							
Opioid use disorder	N (X.x%)						
Alcohol use disorder							
Amphetamine use disorder							
Cannabis use disorder							
Cocaine use disorder							
Sedative use disorder							
Medical and psychiatric history							
HIV	N (X.x%)						
Hepatitis C							
Anxiety or Panic Disorder							
Attention Deficit Hyperactivity Disorder							
Bipolar Disorder							
Eating Disorder							
Major Depressive Disorder							
Schizophrenia							
Suicidal ideation							
Suicidal behavior							
Homicidal ideation							
Violent behavior							
Psychotic episodes not specified above							

Table 16: Summary of Baseline Characteristics by Site

	Gibson Recovery Center (N=XX)	Nexus Recovery Center (N=XX)	Stony Brook Eastern Long Island Hospital (N=XX)	Aspire Health Partners (N=XX)	Avery Road Treatment Center (N=XX)	ADAPT (N=XX)	Total (N=XX)
Other psychiatric disorder							
Medication taken currently for medical and psychiatric condition							
HIV medication taken currently	N (X.x%)						
Hepatitis C medication taken currently							
Anxiety or Panic Disorder medication taken currently							
Attention Deficit Hyperactivity Disorder medication taken currently							
Bipolar Disorder medication taken currently							
Eating Disorder medication taken currently							
Major Depressive Disorder medication taken currently							
Schizophrenia medication taken currently							
Suicidal ideation medication taken currently							
Suicidal behavior medication taken currently							
Homicidal ideation medication taken currently							
Violent behavior medication taken currently							
Psychotic episodes not specified above medication taken currently							
Other psychiatric disorder medication taken currently							

Table 16: Summary of Baseline Characteristics by Site

	Gibson Recovery Center (N=XX)	Nexus Recovery Center (N=XX)	Stony Brook Eastern Long Island Hospital (N=XX)	Aspire Health Partners (N=XX)	Avery Road Treatment Center (N=XX)	ADAPT (N=XX)	Total (N=XX)
Baseline screening for mental health symptoms							
Adult ADHD Self-Report Screening Scale for DSM-5 (ASRS-5) (Score ≥ 14)	N (X.x%)						
PTSD Checklist for DSM-5 (PCL-5) (Score ≥ 31)							
Generalized Anxiety Disorder (GAD-7) (Score ≥ 8)							
Patient Health Questionnaire (PHQ-9) (Score ≥ 10)							
Under criminal justice supervision	N (X.x%)						
Has health insurance	N (X.x%)						
Medicaid	N (X.x%)						
Medicare							
Private Health Insurance							
Military Health Care							
Other							
Don’t know							
History of taking medication to treat opioid use disorder	N (X.x%)						
Buprenorphine-naloxone or buprenorphine daily sublingual	N (X.x%)						
Buprenorphine injection							
Buprenorphine 6-month implant							
Naltrexone daily							
Naltrexone monthly injection							

Table 16: Summary of Baseline Characteristics by Site

	Gibson Recovery Center (N=XX)	Nexus Recovery Center (N=XX)	Stony Brook Eastern Long Island Hospital (N=XX)	Aspire Health Partners (N=XX)	Avery Road Treatment Center (N=XX)	ADAPT (N=XX)	Total (N=XX)
Methadone daily							
Number of times opioid detoxification attempted							
N	N						
Mean	x.x						
SD	x.xx						
Minimum	x						
25th Percentile	x.x						
Median	x.x						
75th Percentile	x.x						
Maximum	x						
Number of opioid detoxifications completed							
N	N						
Mean	x.x						
SD	x.xx						
Minimum	x						
25th Percentile	x.x						
Median	x.x						
75th Percentile	x.x						
Maximum	x						
Previously attempted XR-NTX induction but failed	N (X.x%)						

Table 17: Summary of Baseline Characteristics by Induction Procedure			
	**Induction Procedure**		
	**Standard (N=XX)**	**Rapid (N=XX)**	**Total (N=XX)**
Sex			
Male	N (X.x%)		
Female			
Don't know			
Refused to answer			
Gender			
Missing	N (X.x%)		
Male			
Female			
Transgender male			
Transgender female			
Non-binary			
Not listed			
Age in years (Mean (SD))	x.x (x.xx)		
Age in years			
< 18	N (X.x%)		
18 - < 25			
25 - < 35			
35 - < 45			
45 - < 55			

**Table 17: Summary of Baseline Characteristics by Induction Procedure**			
	**Induction Procedure**		
	**Standard (N=XX)**	**Rapid (N=XX)**	**Total (N=XX)**
55 - < 65			
65 - < 75			
75+			
Ethnicity			
Not Hispanic or Latino	N (X.x%)		
Hispanic or Latino			
Don't know			
Refused to answer			
Race			
American Indian or Alaska Native	N (X.x%)		
Asian			
Black or African American			
Native Hawaiian or Pacific Islander			
White			
Other			
Multiracial			
Don't know			
Refused to answer			
Education completed			
Less than high school diploma	N (X.x%)		

Table 17: Summary of Baseline Characteristics by Induction Procedure			
	**Induction Procedure**		
	**Standard (N=XX)**	**Rapid (N=XX)**	**Total (N=XX)**
High school graduate			
GED or equivalent			
Some college, no degree			
Associate's degree: occupational, technical, or vocational program			
Associate's degree: academic program			
Bachelor's degree			
Master's degree			
Professional school degree			
Doctoral degree			
Don't know			
Refused			
Marital status			
Married	N (X.x%)		
Widowed			
Divorced			
Separated			
Never married			
Living with partner			
Don't know			
Refused			

Table 17: Summary of Baseline Characteristics by Induction Procedure			
	**Induction Procedure**		
	**Standard (N=XX)**	**Rapid (N=XX)**	**Total (N=XX)**
Sexual orientation			
Missing	N (X.x%)		
Heterosexual or straight			
Gay or lesbian			
Bisexual			
Queer			
Not sure			
Something else			
Employment			
Working now	N (X.x%)		
Only temporarily laid off, sick leave, or maternity leave			
Looking for work, unemployed			
Retired			
Disabled permanently or temporarily			
Keeping house			
Student			
Other			
Location participant spent the night before coming to the unit			
Missing	N (X.x%)		

Table 17: Summary of Baseline Characteristics by Induction Procedure			
	**Induction Procedure**		
	**Standard (N=XX)**	**Rapid (N=XX)**	**Total (N=XX)**
Own apartment, room or house - subsidized, for example Section 8 or living in public housing			
Own apartment, room or house - not subsidized			
Someone else's apartment, room or house			
Hotel, SRO, or boarding home			
Halfway house, residential treatment program (focus: establishing sobriety)			
Transitional housing (focus: movement into permanent housing)			
Institution (hospital, nursing home, etc.)			
Homeless shelter			
Outdoors/street, abandoned/public building, vehicle, or other place not meant for human habitation			
Detox			
Other - homeless			
Other - stable housing			
Other			
Refused			
First COWS Score on Day 1 of admission			
N	N		
Mean	x.x		
SD	x.xx		

Table 17: Summary of Baseline Characteristics by Induction Procedure			
	Induction Procedure		
	Standard (N=XX)	Rapid (N=XX)	Total (N=XX)
Minimum	x		
25th Percentile	x.x		
Median	x.x		
75th Percentile	x.x		
Maximum	x		
History of lifetime opioid overdose	N (X.x%)		
Number of lifetime overdoses			
N	N		
Mean	x.x		
SD	x.xx		
Minimum	x		
25th Percentile	x.x		
Median	x.x		
75th Percentile	x.x		
Maximum	x		
Number of days since last self-reported opioid use at Day 1 of admission			
N	N		
Mean	x.x		
SD	x.xx		
Minimum	x		

Table 17: Summary of Baseline Characteristics by Induction Procedure			
	**Induction Procedure**		
	**Standard (N=XX)**	**Rapid (N=XX)**	**Total (N=XX)**
25th Percentile	x.x		
Median	x.x		
75th Percentile	x.x		
Maximum	x		
Baseline substance use (Urine Drug Screen)			
Opiates	N (X.x%)		
Oxycodone			
Methadone			
Buprenorphine			
Amphetamine			
Barbiturate			
Benzodiazepines			
Marijuana			
Cocaine			
Ecstasy (MDMA)			
Methamphetamine			
Phencyclidine			
Fentanyl			
TLFB substance use (at least once in the past 7 days			
Heavy alcohol use (Females: 3 or more; Males: 4 or more (drinks per day))	N (X.x%)		

Table 17: Summary of Baseline Characteristics by Induction Procedure			
	**Induction Procedure**		
	**Standard (N=XX)**	**Rapid (N=XX)**	**Total (N=XX)**
Heroin/Fentanyl			
Opioid analgesics			
Buprenorphine			
Methadone			
No opioids (heroin/fentanyl, opioid analgesics, buprenorphine, methadone)			
Other amphetamine			
Benzodiazepines			
Cannabis			
Cocaine			
Ecstasy (MDMA)			
Methamphetamine			
Inhalant			
Other drugs			
Missing			
TLFB substance use at least once in the past 30 days			
Heavy alcohol use (Females: 3 or more; Males: 4 or more (drinks per day))	N (X.x%)		
Heroin/Fentanyl			
Opioid analgesics			
Buprenorphine			

Table 17: Summary of Baseline Characteristics by Induction Procedure			
	Induction Procedure		
	Standard (N=XX)	Rapid (N=XX)	Total (N=XX)
Methadone			
No opioids (heroin/fentanyl, opioid analgesics, buprenorphine, methadone)			
Amphetamine			
Benzodiazepines			
Cannabis			
Cocaine			
Ecstasy (MDMA)			
Methamphetamine			
Inhalant			
Other drugs			
Missing			
Route of heroin/fentanyl last use from TLFB at baseline			
No use	N (X.x%)		
Oral			
Nasal			
Smoking			
Injection			
Missing			

Table 17: Summary of Baseline Characteristics by Induction Procedure			
	**Induction Procedure**		
	**Standard (N=XX)**	**Rapid (N=XX)**	**Total (N=XX)**
Route of prescription opioid last use from TLFB at baseline			
Nasal	N (X.x%)		
Smoking			
Injection			
Route of methadone last use from TLFB at baseline			
Nasal	N (X.x%)		
Injection			
Route of buprenorphine last use from TLFB at baseline			
Nasal	N (X.x%)		
Injection			
Substance use disorder			
Opioid use disorder	N (X.x%)		
Alcohol use disorder			
Amphetamine use disorder			
Cannabis use disorder			
Cocaine use disorder			
Sedative use disorder			
Medical and psychiatric history			
HIV	N (X.x%)		
Hepatitis C			

Table 17: Summary of Baseline Characteristics by Induction Procedure			
	**Induction Procedure**		
	**Standard (N=XX)**	**Rapid (N=XX)**	**Total (N=XX)**
Anxiety or Panic Disorder			
Attention Deficit Hyperactivity Disorder			
Bipolar Disorder			
Eating Disorder			
Major Depressive Disorder			
Schizophrenia			
Suicidal ideation			
Suicidal behavior			
Homicidal ideation			
Violent behavior			
Psychotic episodes not specified above			
Other psychiatric disorder			
Medication taken currently for medical and psychiatric condition			
HIV medication taken currently	N (X.x%)		
Hepatitis C medication taken currently			
Anxiety or Panic Disorder medication taken currently			
Attention Deficit Hyperactivity Disorder medication taken currently			
Bipolar Disorder medication taken currently			
Eating Disorder medication taken currently			

Table 17: Summary of Baseline Characteristics by Induction Procedure			
	Induction Procedure		
	Standard (N=XX)	Rapid (N=XX)	Total (N=XX)
Major Depressive Disorder medication taken currently			
Schizophrenia medication taken currently			
Suicidal Ideation medication taken currently			
Suicidal behavior medication taken currently			
Homicidal ideation medication taken currently			
Violent behavior medication taken currently			
Psychotic episodes not specified above medication taken currently			
Other psychiatric disorder medication taken currently			
Baseline screening for mental health symptoms			
Adult ADHD Self-Report Screening Scale for DSM-5 (ASRS-5) (Score ≥ 14)	N (X.x%)		
PTSD Checklist for DSM-5 (PCL-5) (Score ≥ 31)			
Generalized Anxiety Disorder (GAD-7) (Score ≥ 8)			
Patient Health Questionnaire (PHQ-9) (Score ≥ 10)			
Under criminal justice supervision	N (X.x%)		
Has health insurance	N (X.x%)		
Medicaid	N (X.x%)		
Medicare			
Private Health Insurance			

Table 17: Summary of Baseline Characteristics by Induction Procedure

	Induction Procedure		
	**Standard (N=XX)**	**Rapid (N=XX)**	**Total (N=XX)**
Military Health Care			
Other			
Don't know			
History of taking medication to treat opioid use disorder	N (X.x%)		
Buprenorphine-naloxone or buprenorphine daily sublingual	N (X.x%)		
Buprenorphine injection			
Buprenorphine 6-month implant			
Naltrexone daily			
Naltrexone monthly injection			
Methadone daily			
Number of times opioid detoxification attempted			
N	N		
Mean	x.x		
SD	x.xx		
Minimum	x		
25th Percentile	x.x		
Median	x.x		
75th Percentile	x.x		
Maximum	x		

Table 17: Summary of Baseline Characteristics by Induction Procedure			
	**Induction Procedure**		
	**Standard (N=XX)**	**Rapid (N=XX)**	**Total (N=XX)**
Number of opioid detoxifications completed			
N	N		
Mean	x.x		
SD	x.xx		
Minimum	x		
25th Percentile	x.x		
Median	x.x		
75th Percentile	x.x		
Maximum	x		
Previously attempted XR-NTX induction but failed	N (X.x%)		

Table 18: Summary of Baseline Characteristics in Study Completers by Induction Procedure			
	Induction Procedure		
	Standard (N=XX)	Rapid (N=XX)	Total (N=XX)
Sex			
Male	N (X.x%)		
Female			
Don't know			
Refused to answer			
Gender			
Missing	N (X.x%)		
Male			
Female			
Transgender male			
Transgender female			
Non-binary			
Not listed			
Age in years (Mean (SD))	x.x (x.xx)		
Age in years			
< 18	N (X.x%)		
18 - < 25			
25 - < 35			
35 - < 45			

Table 18: Summary of Baseline Characteristics in Study Completers by Induction Procedure			
	**Induction Procedure**		
	**Standard (N=XX)**	**Rapid (N=XX)**	**Total (N=XX)**
45 - < 55			
55 - < 65			
65 - < 75			
75+			
Ethnicity			
Not Hispanic or Latino	N (X.x%)		
Hispanic or Latino			
Don't know			
Refused to answer			
Race			
American Indian or Alaska Native	N (X.x%)		
Asian			
Black or African American			
Native Hawaiian or Pacific Islander			
White			
Other			
Multiracial			
Don't know			
Refused to answer			

Table 18: Summary of Baseline Characteristics in Study Completers by Induction Procedure			
	**Induction Procedure**		
	**Standard (N=XX)**	**Rapid (N=XX)**	**Total (N=XX)**
Education completed			
Less than high school diploma	N (X.x%)		
High school graduate			
GED or equivalent			
Some college, no degree			
Associate's degree: occupational, technical, or vocational program			
Associate's degree: academic program			
Bachelor's degree			
Master's degree			
Professional school degree			
Doctoral degree			
Don't know			
Refused			
Marital status			
Married	N (X.x%)		
Widowed			
Divorced			
Separated			
Never married			
Living with partner			

Table 18: Summary of Baseline Characteristics in Study Completers by Induction Procedure			
	**Induction Procedure**		
	**Standard (N=XX)**	**Rapid (N=XX)**	**Total (N=XX)**
Don't know			
Refused			
Sexual orientation			
Missing	N (X.x%)		
Heterosexual or straight			
Gay or lesbian			
Bisexual			
Queer			
Not sure			
Something else			
Employment			
Working now	N (X.x%)		
Only temporarily laid off, sick leave, or maternity leave			
Looking for work, unemployed			
Retired			
Disabled permanently or temporarily			
Keeping house			
Student			
Other			

Table 18: Summary of Baseline Characteristics in Study Completers by Induction Procedure			
	**Induction Procedure**		
	**Standard (N=XX)**	**Rapid (N=XX)**	**Total (N=XX)**
Location participant spent the night before coming to the unit			
Missing	N (X.x%)		
Own apartment, room or house - subsidized, for example Section 8 or living in public housing			
Own apartment, room or house - not subsidized			
Someone else's apartment, room or house			
Hotel, SRO, or boarding home			
Halfway house, residential treatment program (focus: establishing sobriety)			
Transitional housing (focus: movement into permanent housing)			
Institution (hospital, nursing home, etc.)			
Homeless shelter			
Outdoors/street, abandoned/public building, vehicle, or other place not meant for human habitation			
Detox			
Other - homeless			
Other - stable housing			
Other			
Refused			
First COWS Score on Day 1 of admission			
N	N		

Table 18: Summary of Baseline Characteristics in Study Completers by Induction Procedure			
	**Induction Procedure**		
	**Standard (N=XX)**	**Rapid (N=XX)**	**Total (N=XX)**
Mean	x.x		
SD	x.xx		
Minimum	x		
25th Percentile	x.x		
Median	x.x		
75th Percentile	x.x		
Maximum	x		
History of lifetime opioid overdose	N (X.x%)		
Number of lifetime overdoses			
N	N		
Mean	x.x		
SD	x.xx		
Minimum	x		
25th Percentile	x.x		
Median	x.x		
75th Percentile	x.x		
Maximum	x		
Number of days since last self-reported opioid use at Day 1 of admission			
N	N		
Mean	x.x		

Table 18: Summary of Baseline Characteristics in Study Completers by Induction Procedure			
	Induction Procedure		
	Standard (N=XX)	Rapid (N=XX)	Total (N=XX)
SD	x.xx		
Minimum	x		
25th Percentile	x.x		
Median	x.x		
75th Percentile	x.x		
Maximum	x		
Baseline substance use (Urine Drug Screen)			
Opiates	N (X.x%)		
Oxycodone			
Methadone			
Buprenorphine			
Amphetamine			
Barbiturate			
Benzodiazepines			
Marijuana			
Cocaine			
Ecstasy (MDMA)			
Methamphetamine			
Phencyclidine			
Fentanyl			

Table 18: Summary of Baseline Characteristics in Study Completers by Induction Procedure			
	**Induction Procedure**		
	**Standard (N=XX)**	**Rapid (N=XX)**	**Total (N=XX)**
TLFB substance use (at least once in the past 7 days			
Heavy alcohol use (Females: 3 or more; Males: 4 or more (drinks per day))	N (X.x%)		
Heroin/Fentanyl			
Opioid analgesics			
Buprenorphine			
Methadone			
No opioids (heroin/fentanyl, opioid analgesics, buprenorphine, methadone)			
Other amphetamine			
Benzodiazepines			
Cannabis			
Cocaine			
Ecstasy (MDMA)			
Methamphetamine			
Inhalant			
Other drugs			
Missing			
TLFB substance use at least once in the past 30 days			
Heavy alcohol use (Females: 3 or more; Males: 4 or more (drinks per day))	N (X.x%)		

Table 18: Summary of Baseline Characteristics in Study Completers by Induction Procedure			
	Induction Procedure		
	Standard (N=XX)	Rapid (N=XX)	Total (N=XX)
Heroin/Fentanyl			
Opioid analgesics			
Buprenorphine			
Methadone			
No opioids (heroin/fentanyl, opioid analgesics, buprenorphine, methadone)			
Amphetamine			
Benzodiazepines			
Cannabis			
Cocaine			
Ecstasy (MDMA)			
Methamphetamine			
Inhalant			
Other drugs			
Missing			
Route of heroin/fentanyl last use from TLFB at baseline			
No use	N (X.x%)		
Oral			
Nasal			
Smoking			
Injection			

Table 18: Summary of Baseline Characteristics in Study Completers by Induction Procedure			
	**Induction Procedure**		
	**Standard (N=XX)**	**Rapid (N=XX)**	**Total (N=XX)**
Missing			
Route of prescription opioid last use from TLFB at baseline			
Nasal	N (X.x%)		
Smoking			
Injection			
Route of methadone last use from TLFB at baseline			
Nasal	N (X.x%)		
Injection			
Route of buprenorphine last use from TLFB at baseline			
Nasal	N (X.x%)		
Injection			
Substance use disorder			
Opioid use disorder	N (X.x%)		
Alcohol use disorder			
Amphetamine use disorder			
Cannabis use disorder			
Cocaine use disorder			
Sedative use disorder			
Medical and psychiatric history			
HIV	N (X.x%)		

Table 18: Summary of Baseline Characteristics in Study Completers by Induction Procedure			
	**Induction Procedure**		
	**Standard (N=XX)**	**Rapid (N=XX)**	**Total (N=XX)**
Hepatitis C			
Anxiety or Panic Disorder			
Attention Deficit Hyperactivity Disorder			
Bipolar Disorder			
Eating Disorder			
Major Depressive Disorder			
Schizophrenia			
Suicidal ideation			
Suicidal behavior			
Homicidal ideation			
Violent behavior			
Psychotic episodes not specified above			
Other psychiatric disorder			
Medication taken currently for medical and psychiatric condition			
HIV medication taken currently	N (X.x%)		
Hepatitis C medication taken currently			
Anxiety or Panic Disorder medication taken currently			
Attention Deficit Hyperactivity Disorder medication taken currently			
Bipolar Disorder medication taken currently			

Table 18: Summary of Baseline Characteristics in Study Completers by Induction Procedure

	Induction Procedure		
	**Standard (N=XX)**	**Rapid (N=XX)**	**Total (N=XX)**
Eating Disorder medication taken currently			
Major Depressive Disorder medication taken currently			
Schizophrenia medication taken currently			
Suicidal Ideation medication taken currently			
Suicidal behavior medication taken currently			
Homicidal ideation medication taken currently			
Violent behavior medication taken currently			
Psychotic episodes not specified above medication taken currently			
Other psychiatric disorder medication taken currently			
Baseline screening for mental health symptoms			
Adult ADHD Self-Report Screening Scale for DSM-5 (ASRS-5) (Score ≥ 14)	N (X.x%)		
PTSD Checklist for DSM-5 (PCL-5) (Score ≥ 31)			
Generalized Anxiety Disorder (GAD-7) (Score ≥ 8)			
Patient Health Questionnaire (PHQ-9) (Score ≥ 10)			
Under criminal justice supervision	N (X.x%)		
Has health insurance	N (X.x%)		
Medicaid	N (X.x%)		
Medicare			

Table 18: Summary of Baseline Characteristics in Study Completers by Induction Procedure			
	Induction Procedure		
	**Standard (N=XX)**	**Rapid (N=XX)**	**Total (N=XX)**
Private Health Insurance			
Military Health Care			
Other			
Don't know			
History of taking medication to treat opioid use disorder	N (X.x%)		
Buprenorphine-naloxone or buprenorphine daily sublingual	N (X.x%)		
Buprenorphine injection			
Buprenorphine 6-month implant			
Naltrexone daily			
Naltrexone monthly injection			
Methadone daily			
Number of times opioid detoxification attempted			
N	N		
Mean	x.x		
SD	x.xx		
Minimum	x		
25th Percentile	x.x		
Median	x.x		
75th Percentile	x.x		
Maximum	x		

Table 18: Summary of Baseline Characteristics in Study Completers by Induction Procedure			
	**Induction Procedure**		
	**Standard (N=XX)**	**Rapid (N=XX)**	**Total (N=XX)**
Number of opioid detoxifications completed			
N	N		
Mean	x.x		
SD	x.xx		
Minimum	x		
25th Percentile	x.x		
Median	x.x		
75th Percentile	x.x		
Maximum	x		
Previously attempted XR-NTX induction but failed	N (X.x%)		

19.1.3 Medications Administered During Induction Phase

Table 19: Summary of Daily Medications Administered by Induction Procedure

		Induction Procedure		
		**Standard (N =XX)**	**Rapid (N =XX)**	**Total (N =XX)**
Buprenorphine	Number of participants	N (X.x%)		
	Average number of days	X.x		
	Average total daily dose (mg)	X.x		
Oral Naltrexone	Number of participants	N (X.x%)		
	Average number of days	X.x		
	Average total daily dose (mg)	X.x		
Clonidine	Number of participants	N (X.x%)		
	Average number of days	X.x		
	Average total daily dose (mg)	X.x		
Clonazepam	Number of participants	N (X.x%)		
	Average number of days	X.x		
	Average total daily dose (mg)	X.x		
Other Benzodiazepines				
Diazepam	Number of participants	N (X.x%)		
	Average number of days	X.x		
	Average total daily dose (mg)	X.x		
Chlordiazepoxide	Number of participants	N (X.x%)		
	Average number of days	X.x		
	Average total daily dose (mg)	X.x		

Table 19: Summary of Daily Medications Administered by Induction Procedure

		Induction Procedure		Total (N =XX)
		Standard (N =XX)	Rapid (N =XX)	
Lorazepam	Number of participants	N (X.x%)		
	Average number of days	X.x		
	Average total daily dose (mg)	X.x		
Alprazolam	Number of participants	N (X.x%)		
	Average number of days	X.x		
	Average total daily dose (mg)	X.x		
Temazepam	Number of participants	N (X.x%)		
	Average number of days	X.x		
	Average total daily dose (mg)	X.x		
Oxazepam	Number of participants	N (X.x%)		
	Average number of days	X.x		
	Average total daily dose (mg)	X.x		
Other	Number of participants	N (X.x%)		
Antiemetic				
Prochlorperazine	Number of participants	N (X.x%)		
	Average number of days	X.x		
	Average total daily dose (mg)	X.x		
Promethazine	Number of participants	N (X.x%)		
	Average number of days	X.x		
	Average total daily dose (mg)	X.x		

Table 19: Summary of Daily Medications Administered by Induction Procedure				
		**Induction Procedure**		
		**Standard (N =XX)**	**Rapid (N =XX)**	**Total (N =XX)**
Meclizine	Number of participants	N (X.x%)		
	Average number of days	X.x		
	Average total daily dose (mg)	X.x		
Ondansetron	Number of participants	N (X.x%)		
	Average number of days	X.x		
	Average total daily dose (mg)	X.x		
Other	Number of participants			
Antidiarrheal				
Loperamide	Number of participants	N (X.x%)		
Diphenoxylate/atropine (Lomotil)	Number of participants	N (X.x%)		
Octreotide	Number of participants	N (X.x%)		
Other	Number of participants	N (X.x%)		
Sleep agent				
Trazodone	Number of participants	N (X.x%)		
	Average number of days	X.x		
	Average total daily dose (mg)	X.x		
Zolpidem	Number of participants	N (X.x%)		
	Average number of days	X.x		
	Average total daily dose (mg)	X.x		
Mirtazapine	Number of participants	N (X.x%)		

Table 19: Summary of Daily Medications Administered by Induction Procedure

		Induction Procedure		
		**Standard (N =XX)**	**Rapid (N =XX)**	**Total (N =XX)**
Doxepin	Number of participants	N (X.x%)		
Melatonin	Number of participants	N (X.x%)		
Suvorexant	Number of participants	N (X.x%)		
Eszopiclone	Number of participants	N (X.x%)		
Ramelteon	Number of participants	N (X.x%)		
Other	Number of participants	N (X.x%)		
Non-Steroidal Anti-Inflammatory Agent				
Ibuprofen	Number of participants	N (X.x%)		
Aspirin	Number of participants	N (X.x%)		
Naproxen	Number of participants	N (X.x%)		
Ketorolac	Number of participants	N (X.x%)		
Other	Number of participants	N (X.x%)		
Nicotine Replacement Therapy				
Nicotine patch	Number of participants	N (X.x%)		
Nicotine patch plus other	Number of participants	N (X.x%)		
Nicotine gum	Number of participants	N (X.x%)		
Nicotine lozenge	Number of participants	N (X.x%)		
Other	Number of participants	N (X.x%)		

Table 19: Summary of Daily Medications Administered by Induction Procedure				
		Induction Procedure		
		Standard (N =XX)	Rapid (N =XX)	Total (N =XX)
Alpha-2 Agonists				
Lofexidine	Number of participants	N (X.x%)		
Tizanidine	Number of participants	N (X.x%)		
Clonidine patch	Number of participants	N (X.x%)		
Guanfacine	Number of participants	N (X.x%)		
Other	Number of participants	N (X.x%)		
Anxiety/Antihistamine Agents				
Hydroxyzine	Number of participants	N (X.x%)		
Diphenhydramine	Number of participants	N (X.x%)		
Other	Number of participants	N (X.x%)		
GABA Agents/Muscle Relaxants				
Gabapentin	Number of participants	N (X.x%)		
Pregabalin	Number of participants	N (X.x%)		
Baclofen	Number of participants	N (X.x%)		
Cyclobenzaprine	Number of participants	N (X.x%)		
Other	Number of participants	N (X.x%)		
Antacids				
Calcium carbonate	Number of participants	N (X.x%)		
Simethicone	Number of participants	N (X.x%)		
Sodium bicarbonate	Number of participants	N (X.x%)		

Table 19: Summary of Daily Medications Administered by Induction Procedure				
		**Induction Procedure**		
		**Standard (N =XX)**	**Rapid (N =XX)**	**Total (N =XX)**
Other	Number of participants	N (X.x%)		
Neuroleptics				
Quetiapine	Number of participants	N (X.x%)		
Olanzapine	Number of participants	N (X.x%)		
Risperidone	Number of participants	N (X.x%)		
Haloperidol	Number of participants	N (X.x%)		
Chlorpromazine	Number of participants	N (X.x%)		
Other	Number of participants	N (X.x%)		
Naloxone	Number of participants	N (X.x%)		
Benzodiazepines (IM)	Number of participants	N (X.x%)		
Clonidine patch	Number of participants	N (X.x%)		
Buprenorphine patch	Number of participants	N (X.x%)		
Methadone	Number of participants	N (X.x%)		
Other	Number of participants	N (X.x%)		

Table 20: Summary of Daily Medications Administered in Standard Procedure by Inpatient Day

(N=XX)

**Inpatient Day**		**Buprenorphine**	**Clonidine**	**Clonazepam**
Day 1	Number of participants	N (X.x%)	N (X.x%)	N (X.x%)
	Average total daily dose (mg)	X.x	X.x	X.x
Day 2	Number of participants			
	Average total daily dose (mg)			
Day #	Number of participants			
	Average total daily dose (mg)			

Table 20: Summary of Daily Medications Administered in Standard Procedure by Inpatient Day

(N=XX)

**Inpatient Day**		**Diazepam**	**Chlordiazepoxide**	**Lorazepam**	**Alprazolam**	**Temazepam**	**Oxazepam**	**Other Benzodiazepine**
Day 1	Number of participants	N (X.x%)	N (X.x%)	N (X.x%)	N (X.x%)	N (X.x%)	N (X.x%)	N (X.x%)
	Average total daily dose (mg)	X.x	X.x	X.x	X.x	X.x	X.x	-
Day 2	Number of participants							
	Average total daily dose (mg)							
Day #	Number of participants							
	Average total daily dose (mg)							

Table 20: Summary of Daily Medications Administered in Standard Procedure by Inpatient Day						
(N=XX)						
**Inpatient Day**		**Prochlorperazine**	**Promethazine**	**Meclizine**	**Ondansetron**	**Other Antiemetic**
Day 1	Number of participants	N (X.x%)	N (X.x%)	N (X.x%)	N (X.x%)	N (X.x%)
	Average total daily dose (mg)	X.x	X.x	X.x	X.x	-
Day 2	Number of participants					
	Average total daily dose (mg)					
Day #	Number of participants					
	Average total daily dose (mg)					

Table 20: Summary of Daily Medications Administered in Standard Procedure by Inpatient Day							
(N=XX)							
**Inpatient Day**		**Antidiarrheal**	**Trazodone**	**Zolpidem**	**Other Sleep Agent**	**Non-Steroidal Anti-Inflammatory Agent**	**Nicotine Replacement Therapy**
Day 1	Number of participants	N (X.x%)	N (X.x%)	N (X.x%)	N (X.x%)	N (X.x%)	N (X.x%)
	Average total daily dose (mg)	-	X.x	X.x	-	-	-
Day 2	Number of participants						
	Average total daily dose (mg)						
Day #	Number of participants						
	Average total daily dose (mg)						

Table 20: Summary of Daily Medications Administered in Standard Procedure by Inpatient Day						
(N=XX)						
**Inpatient Day**		**Alpha 2 Agonists**	**Anxiety/Antihistamine Agents**	**GABA Agents/Muscle Relaxants**	**Antacids**	**Neuroleptics**
Day 1	Number of participants	N (X.x%)	N (X.x%)	N (X.x%)	N (X.x%)	N (X.x%)
	Average total daily dose (mg)	-	-	-	-	-
Day 2	Number of participants					
	Average total daily dose (mg)					
Day #	Number of participants					
	Average total daily dose (mg)					

Table 20: Summary of Daily Medications Administered in Standard Procedure by Inpatient Day						
(N=XX)						
**Inpatient Day**		**Naloxone**	**Benzodiazepines (IM)**	**Clonidine patch**	**Buprenorphine Patch**	**Methadone**
Day 1	Number of participants	N (X.x%)	N (X.x%)	N (X.x%)	N (X.x%)	N (X.x%)
	Average total daily dose (mg)	-	-	-	-	-
Day 2	Number of participants					
	Average total daily dose (mg)					
Day #	Number of participants					
	Average total daily dose (mg)					

Table 21: Summary of Daily Medications Administered in Rapid Procedure by Inpatient Day

(N=XX)

Inpatient Day		Buprenorphine	Oral Naltrexone	Clonidine	Clonazepam
Day 1	Number of participants	N (X.x%)	N (X.x%)	N (X.x%)	N (X.x%)
	Average total daily dose (mg)	X.x	X.x	X.x	X.x
Day 2	Number of participants				
	Average total daily dose (mg)				
Day #	Number of participants				
	Average total daily dose (mg)				

Table 21: Summary of Daily Medications Administered in Rapid Procedure by Inpatient Day

(N=XX)

Inpatient Day		Diazepam	Chlordiazepoxide	Lorazepam	Alprazolam	Temazepam	Oxazepam	Other Benzodiazepine
Day 1	Number of participants	N (X.x%)	N (X.x%)	N (X.x%)	N (X.x%)	N (X.x%)	N (X.x%)	N (X.x%)
	Average total daily dose (mg)	X.x	X.x	X.x	X.x	X.x	X.x	-
Day 2	Number of participants							
	Average total daily dose (mg)							
Day #	Number of participants							
	Average total daily dose (mg)							

Table 21: Summary of Daily Medications Administered in Rapid Procedure by Inpatient Day

(N=XX)

**Inpatient Day**		**Prochlorperazine**	**Promethazine**	**Meclizine**	**Ondansetron**	**Other Antiemetic**
Day 1	Number of participants	N (X.x%)	N (X.x%)	N (X.x%)	N (X.x%)	N (X.x%)
	Average total daily dose (mg)	X.x	X.x	X.x	X.x	-
Day 2	Number of participants					
	Average total daily dose (mg)					
Day #	Number of participants					
	Average total daily dose (mg)					

Table 21: Summary of Daily Medications Administered in Rapid Procedure by Inpatient Day

(N=XX)

**Inpatient Day**		**Antidiarrheal**	**Trazodone**	**Zolpidem**	**Other Sleep Agent**	**Non-Steroidal Anti-Inflammatory Agent**	**Nicotine Replacement Therapy**
Day 1	Number of participants	N (X.x%)	N (X.x%)	N (X.x%)	N (X.x%)	N (X.x%)	N (X.x%)
	Average total daily dose (mg)	-	X.x	X.x	-	-	-
Day 2	Number of participants						
	Average total daily dose (mg)						
Day #	Number of participants						
	Average total daily dose (mg)						

Table 21: Summary of Daily Medications Administered in Rapid Procedure by Inpatient Day						
(N=XX)						
**Inpatient Day**		**Alpha 2 Agonists**	**Anxiety/Antihistamine Agents**	**GABA Agents/Muscle Relaxants**	**Antacids**	**Neuroleptics**
Day 1	Number of participants	N (X.x%)	N (X.x%)	N (X.x%)	N (X.x%)	N (X.x%)
	Average total daily dose (mg)	-	-	-	-	-
Day 2	Number of participants					
	Average total daily dose (mg)					
Day #	Number of participants					
	Average total daily dose (mg)					

Table 21: Summary of Daily Medications Administered in Rapid Procedure by Inpatient Day						
(N=XX)						
**Inpatient Day**		**Naloxone**	**Benzodiazepines (IM)**	**Clonidine patch**	**Buprenorphine Patch**	**Methadone**
Day 1	Number of participants	N (X.x%)	N (X.x%)	N (X.x%)	N (X.x%)	N (X.x%)
	Average total daily dose (mg)	-	-	-	-	-
Day 2	Number of participants					
	Average total daily dose (mg)					
Day #	Number of participants					
	Average total daily dose (mg)					

**Table 19: Summary of Daily Medications Administered to Participants who Initiated Buprenorphine in Standard Procedure by Procedure Phase**				
**(N=XX)**				
**Inpatient Day**		**Buprenorphine**	**Clonidine**	**Clonazepam**
Pre-Buprenorphine Day #	Number of participants	N (X.x%)	N (X.x%)	N (X.x%)
	Average total daily dose (mg)	X.x	X.x	X.x
Buprenorphine Day #	Number of participants			
	Average total daily dose (mg)			
Buprenorphine Washout Day #	Number of participants			
	Average total daily dose (mg)			
Day of XR-NTX Injection Success	Number of participants			
	Average total daily dose (mg)			
Day of XR-NTX Injection Failure	Number of participants			
	Average total daily dose (mg)			

Table 22: Summary of Daily Medications Administered to Participants who Initiated Buprenorphine in Standard Procedure by Procedure Phase

(N=XX)

**Inpatient Day**		**Diazepam**	**Chlordiazepoxide**	**Lorazepam**	**Alprazolam**	**Temazepam**	**Oxazepam**	**Other Benzodiazepine**
Pre-Buprenorphine Day #	Number of participants	N (X.x%)	N (X.x%)	N (X.x%)	N (X.x%)	N (X.x%)	N (X.x%)	N (X.x%)
	Average total daily dose (mg)	X.x	X.x	X.x	X.x	X.x	X.x	-
Buprenorphine Day #	Number of participants							
	Average total daily dose (mg)							
Buprenorphine Washout Day #	Number of participants							
	Average total daily dose (mg)							
Day of XR-NTX Injection Success	Number of participants							
	Average total daily dose (mg)							
Day of XR-NTX Injection Failure	Number of participants							
	Average total daily dose (mg)							

Table 22: Summary of Daily Medications Administered to Participants who Initiated Buprenorphine in Standard Procedure by Procedure Phase

(N=XX)

**Inpatient Day**		**Prochlorperazine**	**Promethazine**	**Meclizine**	**Ondansetron**	**Other Antiemetic**
Pre-Buprenorphine Day #	Number of participants	N (X.x%)	N (X.x%)	N (X.x%)	N (X.x%)	N (X.x%)
	Average total daily dose (mg)	X.x	X.x	X.x	X.x	-
Buprenorphine Day #	Number of participants					
	Average total daily dose (mg)					
Buprenorphine Washout Day #	Number of participants					
	Average total daily dose (mg)					
Day of XR-NTX Injection Success	Number of participants					
	Average total daily dose (mg)					
Day of XR-NTX Injection Failure	Number of participants					
	Average total daily dose (mg)					

**Table 22: Summary of Daily Medications Administered to Participants who Initiated Buprenorphine in Standard Procedure by Procedure Phase**						
**(N=XX)**						
**Inpatient Day**		**Alpha 2 Agonists**	**Anxiety/Antihistamine Agents**	**GABA Agents/Muscle Relaxants**	**Antacids**	**Neuroleptics**
Pre-Buprenorphine Day #	Number of participants	N (X.x%)	N (X.x%)	N (X.x%)	N (X.x%)	N (X.x%)
	Average total daily dose (mg)	-	-	-	-	-
Buprenorphine Day #	Number of participants					
	Average total daily dose (mg)					
Buprenorphine Washout Day #	Number of participants					
	Average total daily dose (mg)					
Day of XR-NTX Injection Success	Number of participants					
	Average total daily dose (mg)					
Day of XR-NTX Injection Failure	Number of participants					
	Average total daily dose (mg)					

Table 22: Summary of Daily Medications Administered to Participants who Initiated Buprenorphine in Standard Procedure by Procedure Phase						
(N=XX)						
**Inpatient Day**		**Naloxone**	**Benzodiazepines (IM)**	**Clonidine patch**	**Buprenorphine Patch**	**Methadone**
Pre-Buprenorphine Day #	Number of participants	N (X.x%)	N (X.x%)	N (X.x%)	N (X.x%)	N (X.x%)
	Average total daily dose (mg)	-	-	-	-	-
Buprenorphine Day #	Number of participants					
	Average total daily dose (mg)					
Buprenorphine Washout Day #	Number of participants					
	Average total daily dose (mg)					
Day of XR-NTX Injection Success	Number of participants					
	Average total daily dose (mg)					
Day of XR-NTX Injection Failure	Number of participants					
	Average total daily dose (mg)					

Table 23: Summary of Daily Medications Administered to Participants who Initiated Buprenorphine in Rapid Procedure by Procedure Phase

**(N=XX)**					
**Inpatient Day**		**Buprenorphine**	**Clonidine**	**Oral Naltrexone**	**Clonazepam**
Pre-Buprenorphine Day #	Number of participants	N (X.x%)	N (X.x%)	N (X.x%)	N (X.x%)
	Average total daily dose (mg)	X.x	X.x	X.x	X.x
Buprenorphine Day #	Number of participants				
	Average total daily dose (mg)				
Buprenorphine Washout Day #	Number of participants				
	Average total daily dose (mg)				
Naltrexone Day #	Number of participants				
	Average total daily dose (mg)				
Day of XR-NTX Injection Success	Number of participants				
	Average total daily dose (mg)				
Day of XR-NTX Injection Failure	Number of participants				
	Average total daily dose (mg)				

Table 23: Summary of Daily Medications Administered to Participants who Initiated Buprenorphine in Rapid Procedure by Procedure Phase

(N=XX)								
**Inpatient Day**		**Diazepam**	**Chlordiazepoxide**	**Lorazepam**	**Alprazolam**	**Temazepam**	**Oxazepam**	**Other Benzodiazepine**
Pre-Buprenorphine Day #	Number of participants	N (X.x%)	N (X.x%)	N (X.x%)	N (X.x%)	N (X.x%)	N (X.x%)	N (X.x%)
	Average total daily dose (mg)	X.x	X.x	X.x	X.x	X.x	X.x	-
Buprenorphine Day #	Number of participants							
	Average total daily dose (mg)							
Buprenorphine Washout Day #	Number of participants							
	Average total daily dose (mg)							
Naltrexone Day #	Number of participants							
	Average total daily dose (mg)							
Day of XR-NTX Injection Success	Number of participants							
	Average total daily dose (mg)							
Day of XR-NTX Injection Failure	Number of participants							
	Average total daily dose (mg)							

Table 23: Summary of Daily Medications Administered to Participants who Initiated Buprenorphine in Rapid Procedure by Procedure Phase						
(N=XX)						
**Inpatient Day**		**Prochlorperazine**	**Promethazine**	**Meclizine**	**Ondansetron**	**Other Antiemetic**
Pre-Buprenorphine Day #	Number of participants	N (X.x%)	N (X.x%)	N (X.x%)	N (X.x%)	N (X.x%)
	Average total daily dose (mg)	X.x	X.x	X.x	X.x	-
Buprenorphine Day #	Number of participants					
	Average total daily dose (mg)					
Buprenorphine Washout Day #	Number of participants					
	Average total daily dose (mg)					
Naltrexone Day #	Number of participants					
	Average total daily dose (mg)					
Day of XR-NTX Injection Success	Number of participants					
	Average total daily dose (mg)					
Day of XR-NTX Injection Failure	Number of participants					
	Average total daily dose (mg)					

Table 23: Summary of Daily Medications Administered to Participants who Initiated Buprenorphine in Rapid Procedure by Procedure Phase							
(N=XX)							
**Inpatient Day**		**Antidiarrheal**	**Trazodone**	**Zolpidem**	**Other Sleep Agent**	**Non-Steroidal Anti-Inflammatory Agent**	**Nicotine Replacement Therapy**
Pre-Buprenorphine Day #	Number of participants	N (X.x%)	N (X.x%)	N (X.x%)	N (X.x%)	N (X.x%)	N (X.x%)
	Average total daily dose (mg)	-	X.x	X.x	-	-	-
Buprenorphine Day #	Number of participants						
	Average total daily dose (mg)						
Buprenorphine Washout Day #	Number of participants						
	Average total daily dose (mg)						
Naltrexone Day #	Number of participants						
	Average total daily dose (mg)						
Day of XR-NTX Injection Success	Number of participants						
	Average total daily dose (mg)						
Day of XR-NTX Injection Failure	Number of participants						
	Average total daily dose (mg)						

Table 23: Summary of Daily Medications Administered to Participants who Initiated Buprenorphine in Rapid Procedure by Procedure Phase						
(N=XX)						
**Inpatient Day**		**Alpha 2 Agonists**	**Anxiety/Antihistamine Agents**	**GABA Agents/Muscle Relaxants**	**Antacids**	**Neuroleptics**
Pre-Buprenorphine Day #	Number of participants	N (X.x%)	N (X.x%)	N (X.x%)	N (X.x%)	N (X.x%)
	Average total daily dose (mg)	-	-	-	-	-
Buprenorphine Day #	Number of participants					
	Average total daily dose (mg)					
Buprenorphine Washout Day #	Number of participants					
	Average total daily dose (mg)					
Naltrexone Day #	Number of participants					
	Average total daily dose (mg)					
Day of XR-NTX Injection Success	Number of participants					
	Average total daily dose (mg)					
Day of XR-NTX Injection Failure	Number of participants					
	Average total daily dose (mg)					

**Table 23: Summary of Daily Medications Administered to Participants who Initiated Buprenorphine in Rapid Procedure by Procedure Phase**						
**(N=XX)**						
**Inpatient Day**		**Naloxone**	**Benzodiazepines (IM)**	**Clonidine patch**	**Buprenorphine Patch**	**Methadone**
Pre-Buprenorphine Day #	Number of participants	N (X.x%)	N (X.x%)	N (X.x%)	N (X.x%)	N (X.x%)
	Average total daily dose (mg)	-	-	-	-	-
Buprenorphine Day #	Number of participants					
	Average total daily dose (mg)					
Buprenorphine Washout Day #	Number of participants					
	Average total daily dose (mg)					
Naltrexone Day #	Number of participants					
	Average total daily dose (mg)					
Day of XR-NTX Injection Success	Number of participants					
	Average total daily dose (mg)					
Day of XR-NTX Injection Failure	Number of participants					
	Average total daily dose (mg)					

19.1.4 Early Induction Termination

Table 24: Summary of Early Induction Terminations by Site

	Gibson Recovery Center (N=XX)	Nexus Recovery Center (N=XX)	Stony Brook Eastern Long Island Hospital (N=XX)	Aspire Health Partners (N=XX)	Avery Road Treatment Center (N=XX)	ADAPT (N=XX)	Total (N=XX)
Number of early induction terminations	N (X.x%)						
Reason for early induction termination[1]							
Prefers other medication (buprenorphine or methadone)	N (X.x%)						
Prefers to not be on medication for opioid use disorder							
Does not want naltrexone since it blocks opioids and prevents high							
Fear of precipitated withdrawal from naltrexone shot							
Withdrawal symptoms were too uncomfortable							
Left detox unit early							
Medical contraindication (including pregnancy, COVID-19 infection)							
Psychiatric contraindication							
Other							
Unknown							

[1] Percentage was based on the number of early induction terminations and may exceed 100% if multiple reasons were selected.

Table 25: Summary of Early Induction Terminations by Induction Procedure			
	**Induction Procedure**		
	**Standard (N=XX)**	**Rapid (N=XX)**	**Total (N=XX)**
Number of early induction terminations	N (X.x%)		
Reason for early induction termination[1]			
Prefers other medication (buprenorphine or methadone)	N (X.x%)		
Prefers to not be on medication for opioid use disorder			
Does not want naltrexone since it blocks opioids and prevents high			
Fear of precipitated withdrawal from naltrexone shot			
Withdrawal symptoms were too uncomfortable			
Left detox unit early			
Medical contraindication (including pregnancy, COVID-19 infection)			
Psychiatric contraindication			
Other			
Unknown			

[1] Percentage was based on the number of early induction terminations and may exceed 100% if multiple reasons were selected.

19.1.5 Treatment Exposure

Table 26: Summary of Treatment Exposure by Site

Site	Number Enrolled	Participants with First Injection Administered While on the Unit[1]	Participants with Second Injection Administered[2]	Participants with Third Injection Administered[3]	Treatment Exposure Percentage[4]
Gibson Recovery Center	N	N (X.x%)	N (X.x%)	N (X.x%)	N (X.x%)
Nexus Recovery Center					
Stony Brook Eastern Long Island Hospital					
Aspire Health Partners					
Avery Road Treatment Center					
ADAPT					
Total					

[1] Percentage was calculated as the number who had first injection administered while on the unit out of the total number enrolled.
[2] Percentage was calculated as the number who had second injections administered out of total number who received the first injection while on the unit.
[3] Percentage was calculated as the number who had third injections administered out of total number who received the second injection.
[4] Percentage was calculated as the number of injections administered out of the expected three injections for participants who received the first injection while on the unit.

Table 27: Summary of Treatment Exposure by Induction Procedure

Induction Procedure	Number Enrolled	Participants with First Injection Administered While on the Unit[1]	Participants with Second Injection Administered[2]	Participants with Third Injection Administered[3]	Treatment Exposure Percentage[4]
Standard	N	N (X.x%)	N (X.x%)	N (X.x%)	N (X.x%)
Rapid					
Total					

[1] Percentage was calculated as the number who had first injection administered while on the unit out of the total number enrolled.
[2] Percentage was calculated as the number who had second injections administered out of total number who received the first injection while on the unit.
[3] Percentage was calculated as the number who had third injections administered out of total number who received the second injection.
[4] Percentage was calculated as the number of injections administered out of the expected three injections for participants who received the first injection while on the unit.

Table 28: Summary of XR-NTX Injections in Induction Failure Participants by Site

Site	Number Enrolled	Number of Induction Failures[1]	Induction Failures Who Had At Least One XR-NTX Injection Administered During Post-Induction Phase[2]
Gibson Recovery Center	N	N (X.x%)	N (X.x%)
Nexus Recovery Center			
Stony Brook Eastern Long Island Hospital			
Aspire Health Partners			
Avery Road Treatment Center			
ADAPT			
Total			

[1] Percentage was calculated as the number who did not have first injection while on the unit administered out of the total number enrolled.
[2] Percentage was calculated as the number who had injections administered out of total number of induction failures.

Table 29: Summary of XR-NTX Injections in Induction Failure Participants by Induction Procedure

Induction Procedure	Number Enrolled	Number of Induction Failures[1]	Induction Failures Who Had at Least One XR-NTX Injection Administered During Post-Induction Phase[2]
Standard	N	N (X.x%)	N (X.x%)
Rapid			
Total			

[1] Percentage was calculated as the number who did not have first injection while on the unit administered out of the total number enrolled.
[2] Percentage was calculated as the number who had injections administered out of total number of induction failures.

19.1.6 Primary Outcome Analyses

Table 30: Summary of Primary Outcome by Site

Site	Number Enrolled	First Injection Administered While on the Unit
Gibson Recovery Center	N	N (X.x%)
Nexus Recovery Center		
Stony Brook Eastern Long Island Hospital		
Aspire Health Partners		
Avery Road Treatment Center		
ADAPT		
Total		

Table 31: Summary of Primary Outcome by Step

Step	Number Enrolled	First Injection Administered While on the Unit
Step 1	N	N (X.x%)
Step 2		
Step 3		
Step 4		
Step 5		
Total		

Table 32: Summary of Primary Outcome and Days to Induction[1] by Site and Step

			Step					Induction Procedure		Total
			Step 1	Step 2	Step 3	Step 4	Step 5	Standard	Rapid	
**Site**	Gibson Recovery Center	Percent inducted	n/N (X.x%)					-		
		Average days to induction (SD)	X.x (X.xx)					-		
	Nexus Recovery Center	Percent inducted								
		Average days to induction (SD)								
	Stony Brook Eastern Long Island Hospital	Percent inducted								
		Average days to induction (SD)								
	Aspire Health Partners	Percent inducted								
		Average days to induction (SD)								
	Avery Road Treatment Center	Percent inducted								
		Average days to induction (SD)								
	ADAPT	Percent inducted							-	
		Average days to induction (SD)							-	
**Induction Procedure**	Standard Total	Percent inducted							-	-
		Average days to induction (SD)							-	-
	Rapid Total	Percent inducted						-		-
		Average days to induction (SD)						-		-
**Total**		Percent inducted						-	-	
		Average days to induction (SD)						-	-	

Table 33: Summary of Primary Outcome by Induction Procedure								
			Results[1]					
Induction Procedure	Number Enrolled	First Injection Administered While on the Unit	Odds Ratio	95% Lower Confidence Limit	95% Upper Confidence Limit	p-value	Number Needed to Treat
Rapid	N	N (X.x%)	X.xx	X.xx	X.xx	0.xxx	X.x
Standard							
Total							

[1] Results are obtained from the generalized linear mixed effects model. To show Rapid Procedure is non-inferior to Standard Procedure, the lower bound of the two-sided 95% CI for the odds ratio for Rapid versus Standard Procedure needs to be higher than 0.67. If the 95% confidence interval for odds ratio lies entirely above 0.67 and also above 1, then there is evidence of superiority in terms of statistical significance at the 5% level ($p < 0.05$).

19.1.7 Supportive Analyses of Primary Outcome

Table 34: Summary of Primary Outcome by Sex and Induction Procedure								
	Standard Procedure		Rapid Procedure		Results[1]			
Subgroup	Number Enrolled	First Injection Administered While on the Unit	Number Enrolled	First Injection Administered While on the Unit	Odds Ratio	95% Lower Confidence Limit	95% Upper Confidence Limit	p-value
Male	N	X (X.x%)	N	X (X.x%)	X.xx	X.xx	X.xx	0.xxx
Female								

[1] Results are obtained from the generalized linear mixed effects model. The p-value for the interaction term between induction procedure and subgroup is shown.

Table 35: Summary of Primary Outcome by Age and induction Procedure

	Standard Procedure		Rapid Procedure		Results[1]			
**Subgroup**	**Number Enrolled**	**First Injection Administered While on the Unit**	**Number Enrolled**	**First Injection Administered While on the Unit**	**Odds Ratio**	**95% Lower Confidence Limit**	**95% Upper Confidence Limit**	**p-value**
≤ 25 years	N	X (X.x%)	N	X (X.x%)	X.xx	X.xx	X.xx	0.xxx
> 25 years								

[1]Results are obtained from the generalized linear mixed effects model. The p-value from the interaction between induction procedure and subgroup is shown.

Table 36: Summary of Primary Outcome by Race and Induction Procedure

	Standard Procedure		Rapid Procedure		Results[1]			
**Subgroup**	**Number Enrolled**	**First Injection Administered While on the Unit**	**Number Enrolled**	**First Injection Administered While on the Unit**	**Odds Ratio**	**95% Lower Confidence Limit**	**95% Upper Confidence Limit**	**p-value**
Black	N	X (X.x%)	N	X (X.x%)	X.xx	X.xx	X.xx	0.xxx
White								
Other								

[1]Results are obtained from the generalized linear mixed effects model. The p-value for the interaction term between induction procedure and subgroup is shown.

Table 37: Summary of Primary Outcome by Ethnicity and Induction Procedure

	Standard Procedure		Rapid Procedure		Results[1]			
**Subgroup**	**Number Enrolled**	**First Injection Administered While on the Unit**	**Number Enrolled**	**First Injection Administered While on the Unit**	**Odds Ratio**	**95% Lower Confidence Limit**	**95% Upper Confidence Limit**	**p-value**
Not Hispanic or Latino	N	X (X.x%)	N	X (X.x%)	X.xx	X.xx	X.xx	0.xxx
Hispanic or Latino								

[1]Results are obtained from the generalized linear mixed effects model. The p-value for the interaction term between induction procedure and subgroup is shown.

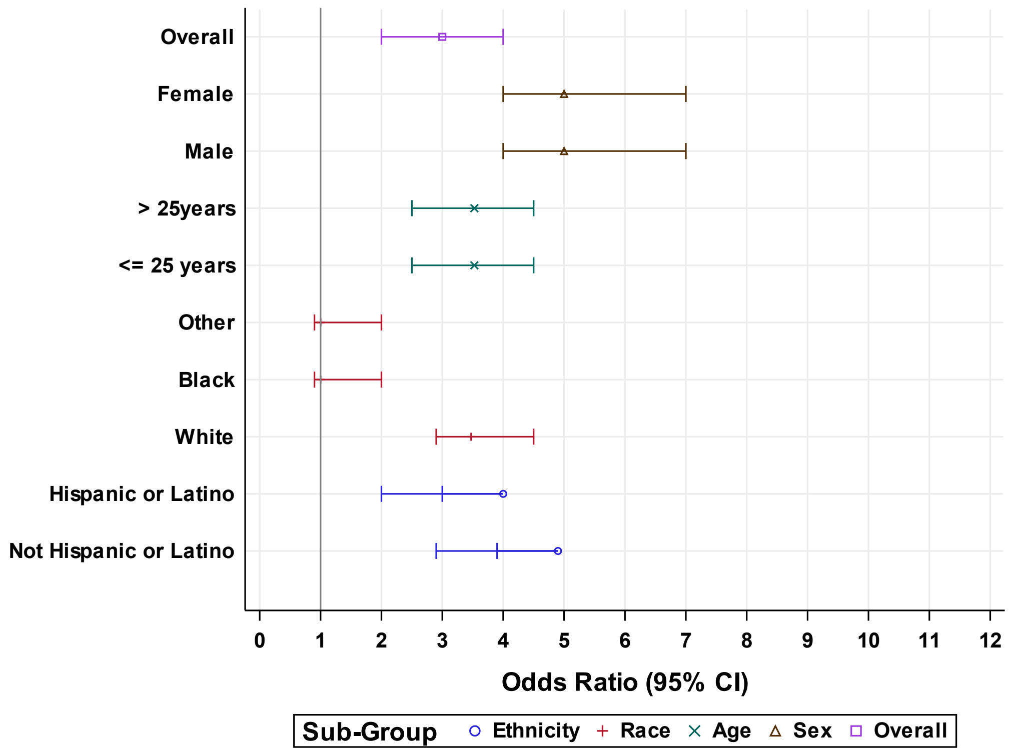

Figure 9: Forest Plot of Odds Ratios by Sub-groups

Example figure provided.

**Table 38: Summary of Primary Outcome Excluding Participants Admitted More than Four Calendar Days Prior to Enrollment by Induction Procedure**						
**Induction Procedure**	**Number Admitted within Four Calendar Days Prior to Enrollment**	**First Injection Administered While on the Unit**	**Odds Ratio**	**95% Lower Confidence Limit**	**95% Upper Confidence Limit**	**p-value**
Rapid	N	N (X.x%)	X.xx	X.xx	X.xx	0.xxx
Standard						
Total						

Participants admitted more than four calendar days prior to enrollment (N=6) were excluded to reflect the updated study exclusion criteria in amended protocol version 3.0 per DSMB recommendation.

Table 39: Summary of Primary Outcome Excluding Participants Enrolled During Pre-Implementation Phase by Induction Procedure

Induction Procedure	Number Admitted Outside of the Pre-implementation Phase	First Injection Administered While on the Unit	Odds Ratio	95% Lower Confidence Limit	95% Upper Confidence Limit	p-value
Rapid	N	N (X.x%)	X.xx	X.xx	X.xx	0.xxx
Standard						
Total						

Participants enrolled in Standard Procedure in the 8 weeks prior to the site crossing over to Rapid Procedure (N=32) were excluded to account for any possible contamination of the SP arm with the RP training.

Table 40: Summary of Primary Outcome by Presence of Baseline Fentanyl Use

Fentanyl Use at Baseline	Number with Baseline Urine Drug Screen	First Injection Administered While on the Unit	Odds Ratio	95% Lower Confidence Limit	95% Upper Confidence Limit	p-value
Positive	N	N (X.x%)	X.xx	X.xx	X.xx	0.xxx
Negative						
Total						

Table 41: Summary of Primary Outcome by Induction Procedure in Participants Who Initiated Buprenorphine

Induction Procedure	Number Enrolled	Participants who Initiated Buprenorphine[1]	Participants who Received First XR-NTX Injection[2]
Rapid	N	N (X.x%)	N (X.x%)
Standard			
Total			

[1] Percentage was calculated with the denominator as number enrolled.
[2] Percentage was calculated with the denominator as the number who initiated buprenorphine.

19.1.8 Key Secondary Outcome Analyses

Table 42: Summary of Days to First XR-NTX Injection by Induction Procedure

Induction Procedure	Days to First Injection While on the Unit		Results		
			Hazard Ratio	95% Lower Confidence Limit	95% Upper Confidence Limit
Rapid	N	N	X.xx	X.xx	X.xx
	Mean	x.x			
	SD	x.xx			
	Minimum	x			
	25th Percentile	x.x			
	Median	x.x			
	75th Percentile	x.x			
	Maximum	x			
Standard	N	N			
	Mean	x.x			
	SD	x.xx			
	Minimum	x			
	25th Percentile	x.x			
	Median	x.x			
	75th Percentile	x.x			
	Maximum	x			
Total	N	N			
	Mean	x.x			
	SD	x.xx			
	Minimum	x			
	25th Percentile	x.x			
	Median	x.x			
	75th Percentile	x.x			
	Maximum	x			

Table 43: Summary of Average Daily Opioid Withdrawal and Craving Scores by Induction Procedure

		Standard Procedure (N=XX)					Rapid Procedure (N=XX)					Total (N=XX)				
**Inpatient Day**		**Daily Maximum COWS[1]**	**Daily COWS[1]**	**Daily SOWS[2]**	**Daily Craving[3]**	**Maximum Craving in the past 24 Hours[4]**	**Daily Maximum COWS[1]**	**Daily COWS[1]**	**Daily SOWS[2]**	**Daily Craving[3]**	**Maximum Craving in the past 24 Hours[4]**	**Daily Maximum COWS[1]**	**Daily COWS[1]**	**Daily SOWS[2]**	**Daily Craving[3]**	**Maximum Craving in the past 24 Hours[4]**
Day 1	N (%)	N (X.x%)														
	Mean (SD)	X.x (X.xx)														
Day 2	N (%)															
	Mean (SD)															
Day 3	N (%)															
	Mean (SD)															
Day 4	N (%)															
	Mean (SD)															
Day #	N (%)															
	Mean (SD)															

[1] Clinical Opiate Withdrawal Scale ranging from 0-48.
[2] Subjective Opioid Withdrawal Scale ranging from 0-64.
[3] Visual Analog Scale for question “Think about your craving for opioids. How intense is it right now?” ranging from 0-100.
[4] Visual Analog Scale for question “Think about your desire to use opioids in the past 24 hours. How intense was your strongest desire to use? ranging from 0-100.

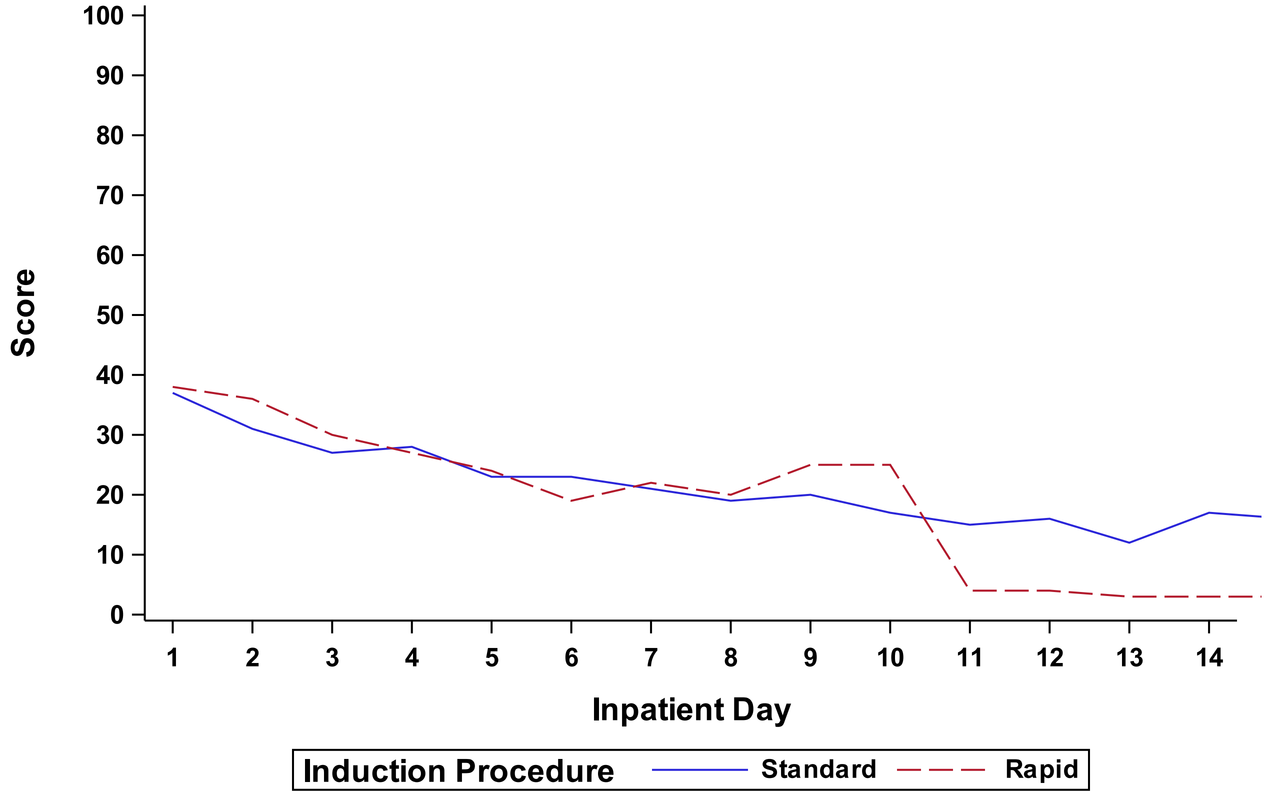

Number of paticipants

**Standard**	7	20	41	61	59	55	54	48	45	39	33	29	27	28
**Rapid**	8	25	56	73	66	58	45	28	11	2	2	1	1	1

Figure 10: Average Daily Maximum COWS Score by Induction Procedure

Example figure provided.

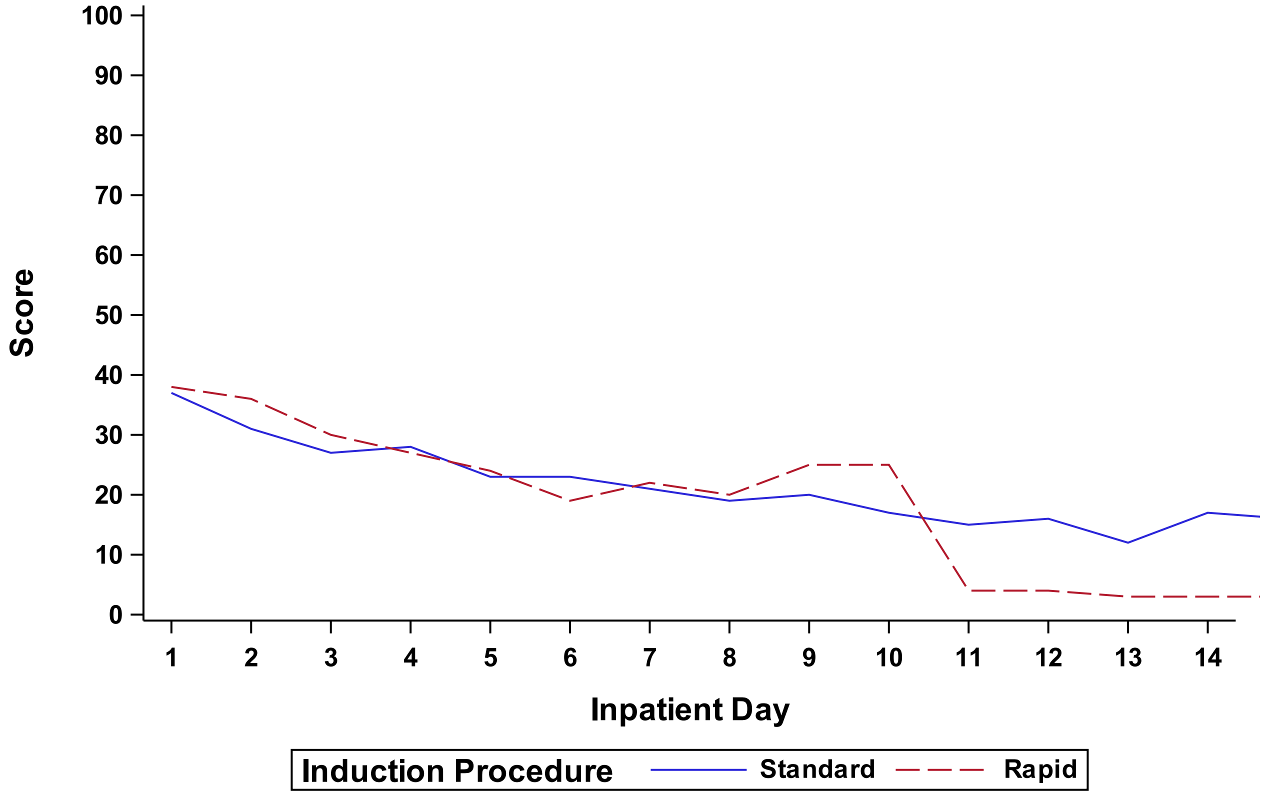

Number of paticipants

**Standard**	7	20	41	61	59	55	54	48	45	39	33	29	27	28
**Rapid**	8	25	56	73	66	58	45	28	11	2	2	1	1	1

Figure 6: Average Daily COWS Score by Induction Procedure

Example figure provided.

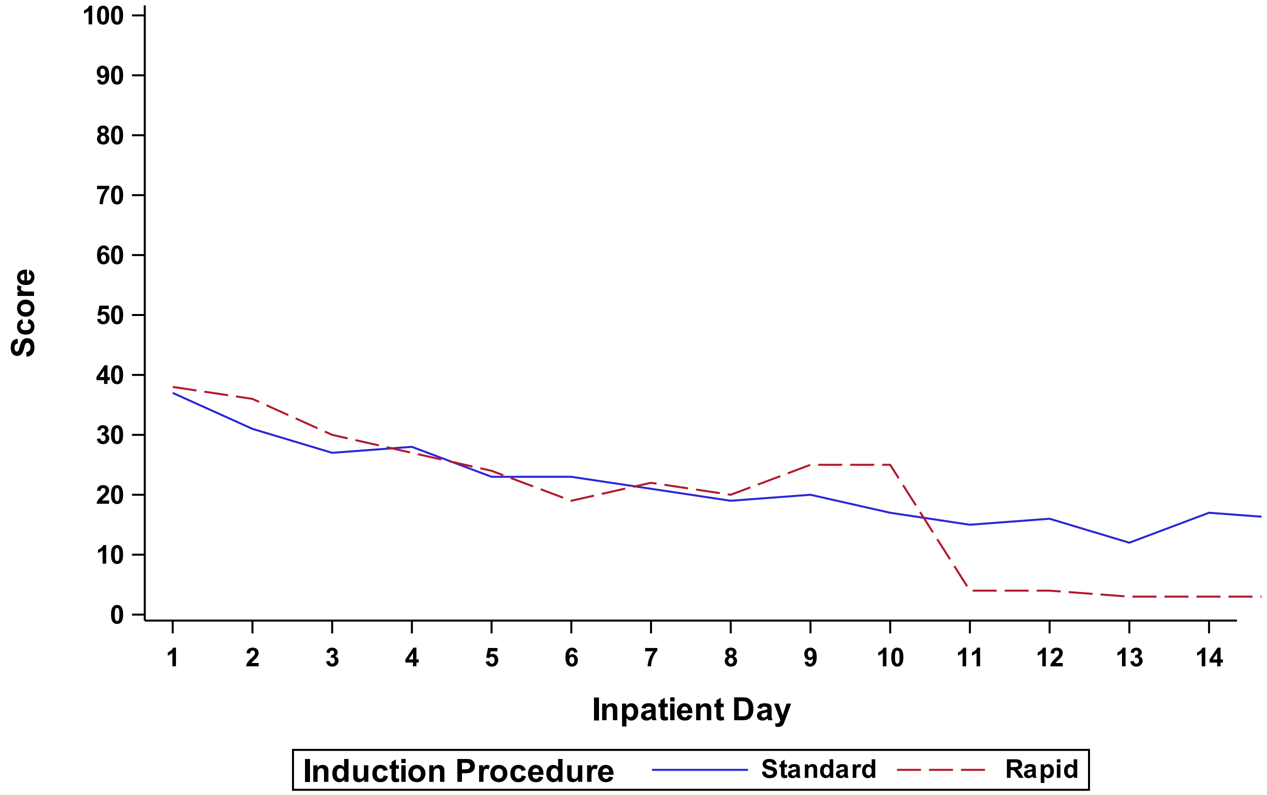

Number of paticipants

**Standard**	7	20	41	61	59	55	54	48	45	39	33	29	27	28
**Rapid**	8	25	56	73	66	58	45	28	11	2	2	1	1	1

Figure 7: Average Daily SOWS Score by Induction Procedure

Example figure provided.

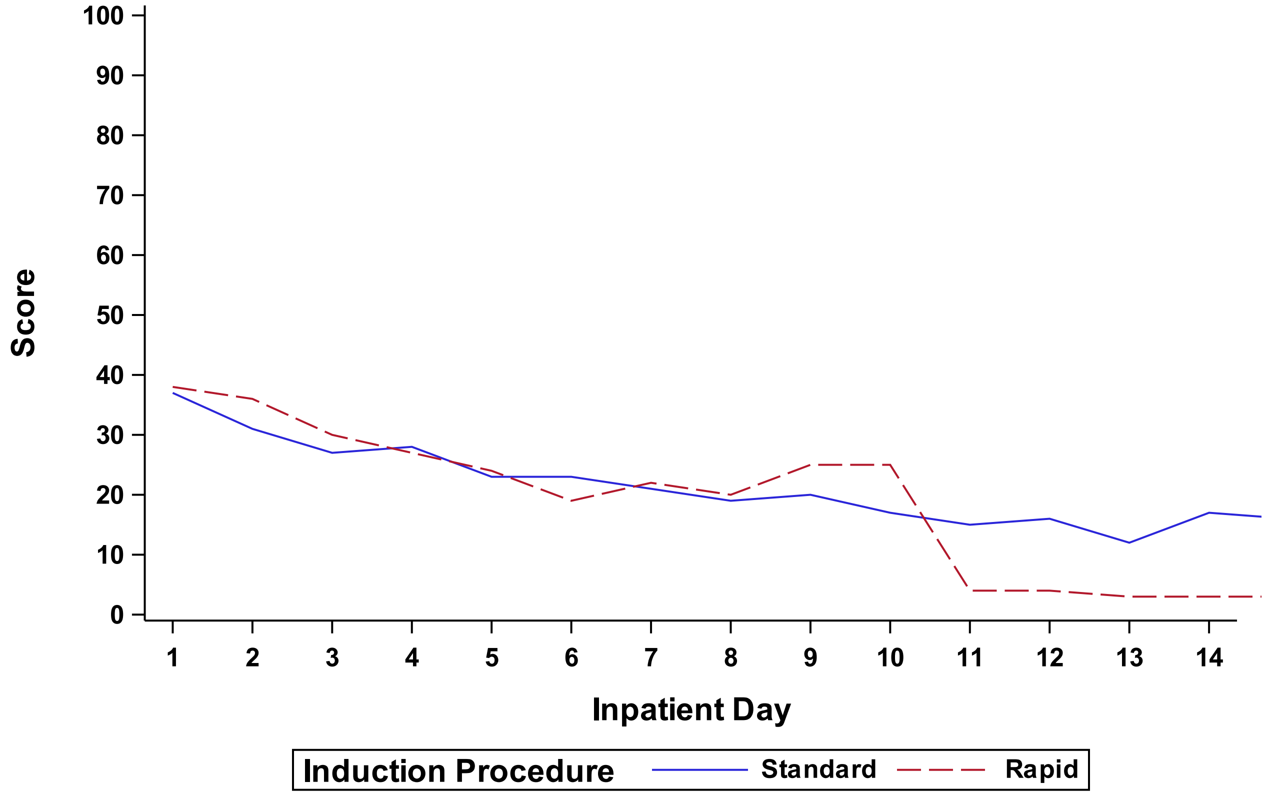

Number of paticipants

	1	2	3	4	5	6	7	8	9	10	11	12	13	14
Standard	7	20	41	61	59	55	54	48	45	39	33	29	27	28
Rapid	8	25	56	73	66	58	45	28	11	2	2	1	1	1

Figure 8: Average Daily VAS Craving Score at Time of Assessment by Induction Procedure

Example figure provided.

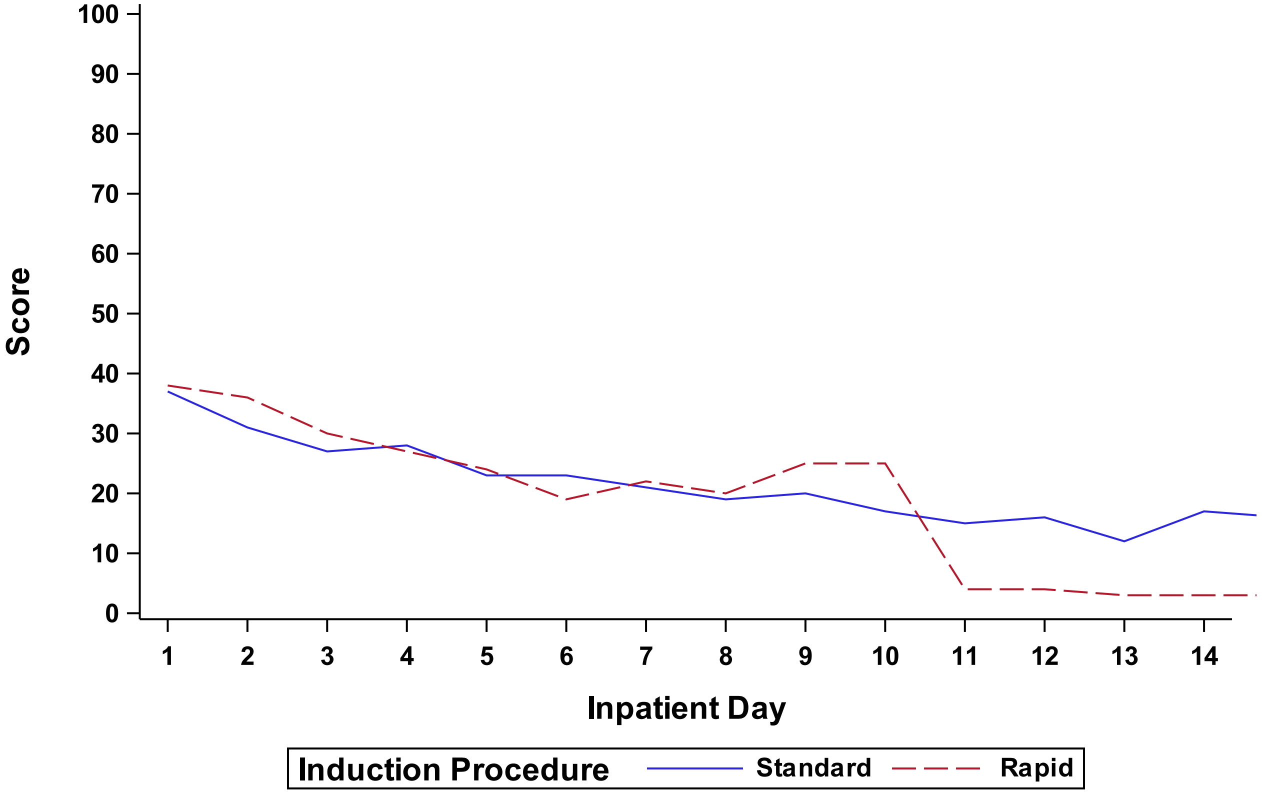

Number of paticipants

	1	2	3	4	5	6	7	8	9	10	11	12	13	14
**Standard**	7	20	41	61	59	55	54	48	45	39	33	29	27	28
**Rapid**	8	25	56	73	66	58	45	28	11	2	2	1	1	1

Figure 9: Average Daily Maximum VAS Craving Score within 24 Hours by Induction Procedure

Example figure provided.

Table 44: Summary of Average Opioid Withdrawal and Craving Scores by Procedure Phase and Induction Procedure

		Standard Procedure (N=XX)					Rapid Procedure (N=XX)					Total (N=XX)				
**Procedure Phase**		**Daily Maximum COWS[1]**	**Daily COWS[1]**	**Daily SOWS[2]**	**Daily Craving[3]**	**Maximum Craving in the past 24 Hours[4]**	**Daily Maximum COWS[1]**	**Daily COWS[1]**	**Daily SOWS[2]**	**Daily Craving[3]**	**Maximum Craving in the past 24 Hours[4]**	**Daily Maximum COWS[1]**	**Daily COWS[1]**	**Daily SOWS[2]**	**Daily Craving[3]**	**Maximum Craving in the past 24 Hours[4]**
Pre-Buprenorphine	Participants with at least one score	N (X.x%)														
	N	N														
	Mean (SD)	X.x (X.xx)														
Buprenorphine	Participants with at least one score	N (X.x%)														
	N	N														
	Mean (SD)	X.x (X.xx)														
Post - Buprenorphine	Participants with at least one score	N (X.x%)														
	N	N														
	Mean (SD)	X.x (X.xx)														
Day of XR-NTX Injection Success	Participants with at least one score	N (X.x%)														
	N	N														
	Mean (SD)	X.x (X.xx)														

Table 44: Summary of Average Opioid Withdrawal and Craving Scores by Procedure Phase and Induction Procedure

		Standard Procedure (N=XX)					Rapid Procedure (N=XX)					Total (N=XX)				
**Procedure Phase**		**Daily Maximum COWS[1]**	**Daily COWS[1]**	**Daily SOWS[2]**	**Daily Craving[3]**	**Maximum Craving in the past 24 Hours[4]**	**Daily Maximum COWS[1]**	**Daily COWS[1]**	**Daily SOWS[2]**	**Daily Craving[3]**	**Maximum Craving in the past 24 Hours[4]**	**Daily Maximum COWS[1]**	**Daily COWS[1]**	**Daily SOWS[2]**	**Daily Craving[3]**	**Maximum Craving in the past 24 Hours[4]**
Pre XR-NTX injection[5]	Participants with at least one score	N (X.x%)														
	N	N														
	Mean (SD)	X.x (X.xx)														
Post XR-NTX Injection[5]	Participants with at least one score	N (X.x%)														
	N	N														
	Mean (SD)	X.x (X.xx)														
Day of XR-NTX Injection Failure	Participants with at least one score	N (X.x%)														
	N	N														
	Mean (SD)	X.x (X.xx)														
Did Not Receive Buprenorphine	Participants with at least one score	N (X.x%)														
	N	N														
	Mean (SD)	X.x (X.xx)														

[1] Clinical Opiate Withdrawal Scale ranging from 0-48.
[2] Subjective Opioid Withdrawal Scale ranging from 0-64.
[3] Visual Analog Scale for question “Think about your craving for opioids. How intense is it right now?” ranging from 0-100.
[4] Visual Analog Scale for question “Think about your desire to use opioids in the past 24 hours. How intense was your strongest desire to use?” ranging from 0-100.
[5] Only included if time of assessment collected on form. Percentage was calculated based on the number of participants who received XR-NTX injection.

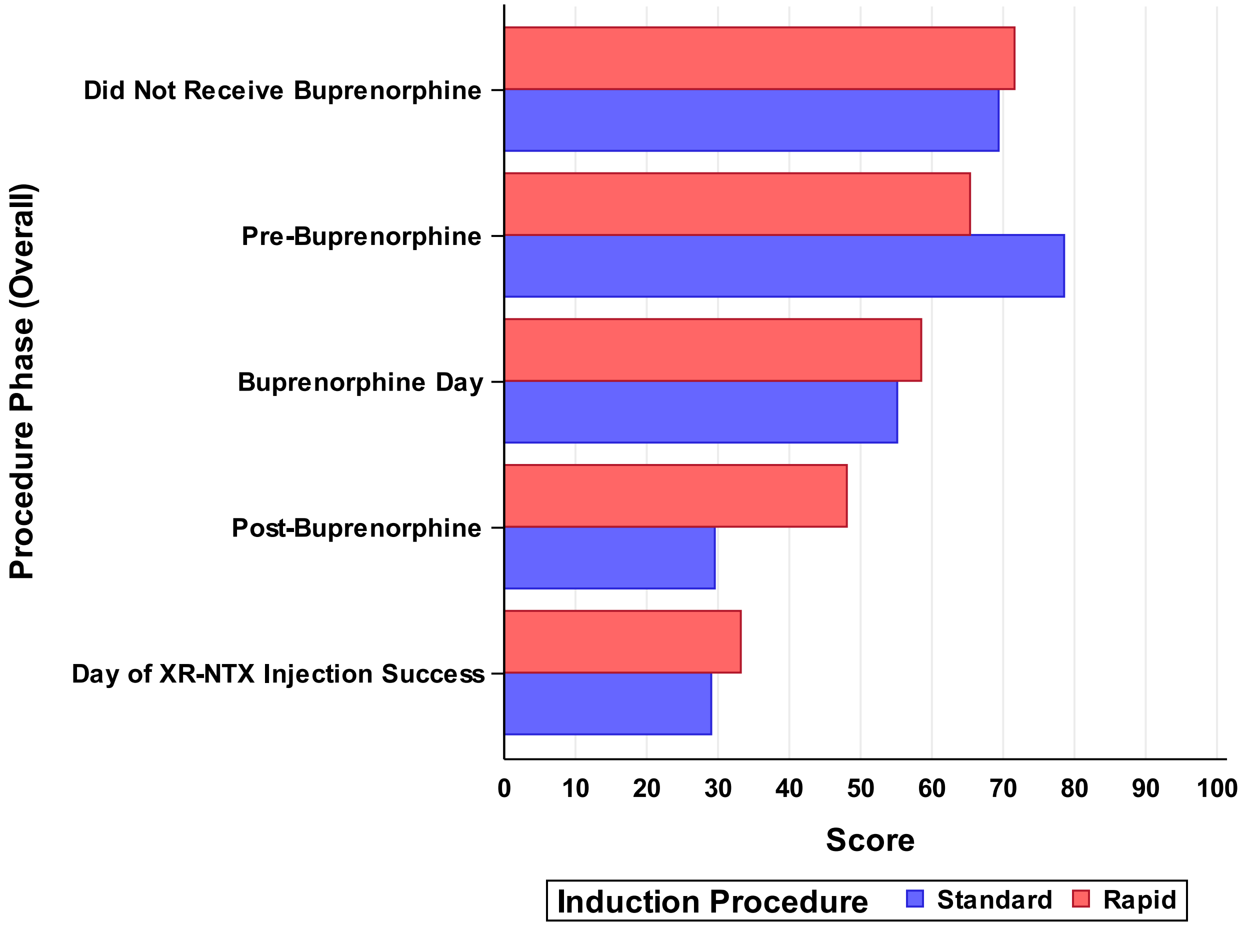

Figure 10: Average Daily Maximum COWS Score by Induction Procedure and Phase

Example figure provided.

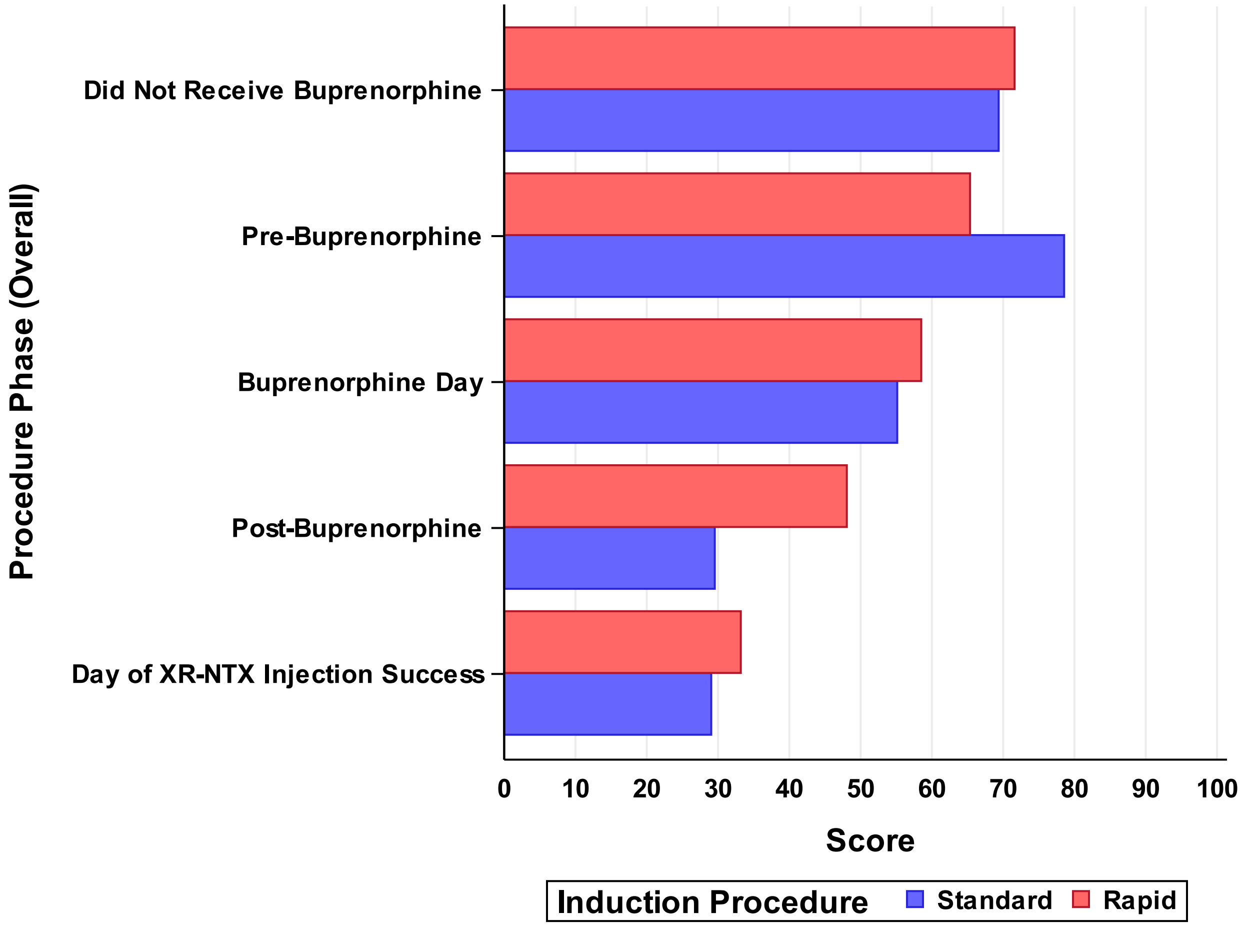

Figure 11: Average Daily COWS Score by Induction Procedure and Phase

Example figure provided.

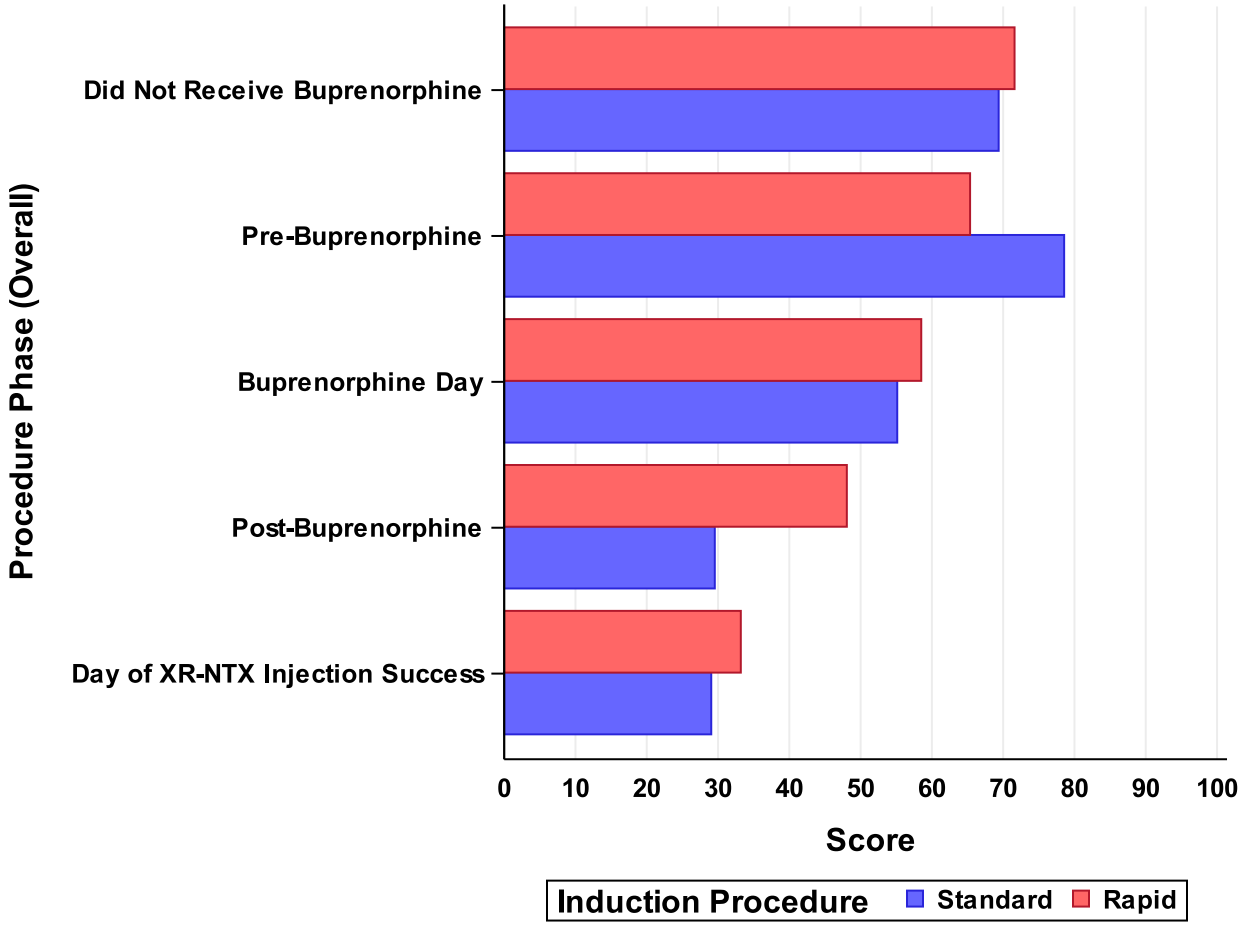

Figure 12: Average Daily SOWS Score by Induction Procedure and Phase

Example figure provided.

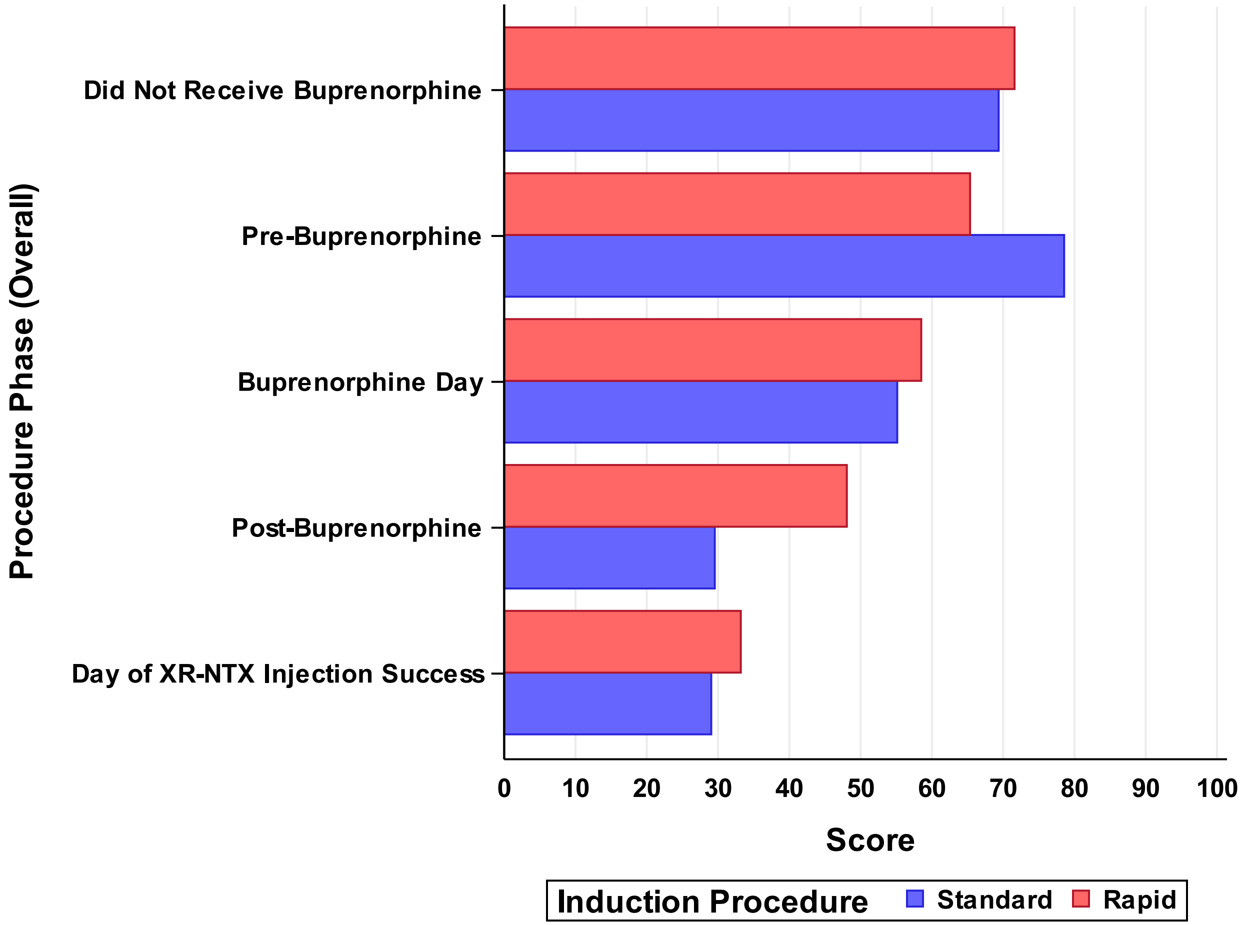

Figure 13: Average VAS Craving Score at Time of Assessment by Induction Procedure and Phase

Example figure provided.

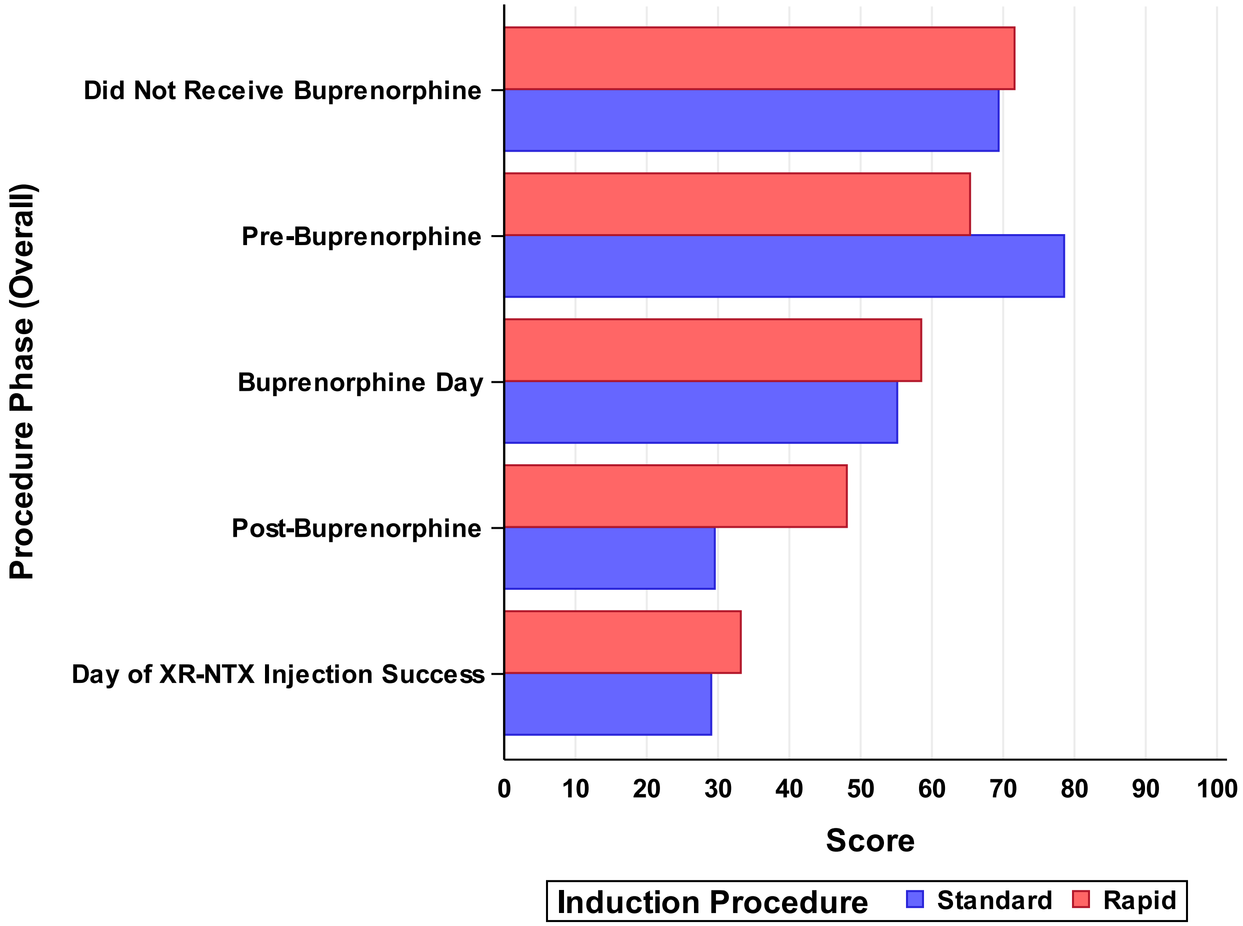

Figure 14: Average Maximum VAS Craving Score within 24 Hours by Induction Procedure and Phase

Example figure provided.

Table 45: Summary of Average Opioid Withdrawal and Craving in Standard Procedure by Procedure Phase						
(N=XX)						
**Procedure Phase**		**Daily Maximum COWS[1]**	**Daily COWS[1]**	**Daily SOWS[2]**	**Daily Craving[3]**	**Maximum Craving in the past 24 Hours[4]**
Pre-Buprenorphine	Participants with at least one score	N (X.x%)				
	N	N				
	Mean (SD)	X.x (X.xx)				
Buprenorphine Taper	Participants with at least one score	N (X.x%)				
	N	N				
	Mean (SD)	X.x (X.xx)				
Buprenorphine Washout	Participants with at least one score	N (X.x%)				
	N	N				
	Mean (SD)	X.x (X.xx)				
Day of XR-NTX Injection Success	Participants with at least one score	N (X.x%)				
	N	N				
	Mean (SD)	X.x (X.xx)				
Pre XR-NTX injection[5]	Participants with at least one score	N (X.x%)				
	N	N				
	Mean (SD)	X.x (X.xx)				
Post XR-NTX Injection[5]	Participants with at least one score	N (X.x%)				
	N	N				
	Mean (SD)	X.x (X.xx)				

Table 45: Summary of Average Opioid Withdrawal and Craving in Standard Procedure by Procedure Phase						
(N=XX)						
**Procedure Phase**		**Daily Maximum COWS[1]**	**Daily COWS[1]**	**Daily SOWS[2]**	**Daily Craving[3]**	**Maximum Craving in the past 24 Hours[4]**
Day of XR-NTX Injection Failure	Participants with at least one score	N (X.x%)				
	N	N				
	Mean (SD)	X.x (X.xx)				

[1] Clinical Opiate Withdrawal Scale ranging from 0-48.
[2] Subjective Opioid Withdrawal Scale ranging from 0-64.
[3] Visual Analog Scale for question “Think about your craving for opioids. How intense is it right now?” ranging from 0-100.
[4] Visual Analog Scale for question “Think about your desire to use opioids in the past 24 hours. How intense was your strongest desire to use?” ranging from 0-100.
[5] Only included if time of assessment collected on form. Percentage was calculated based on the number of participants who received XR-NTX injection.

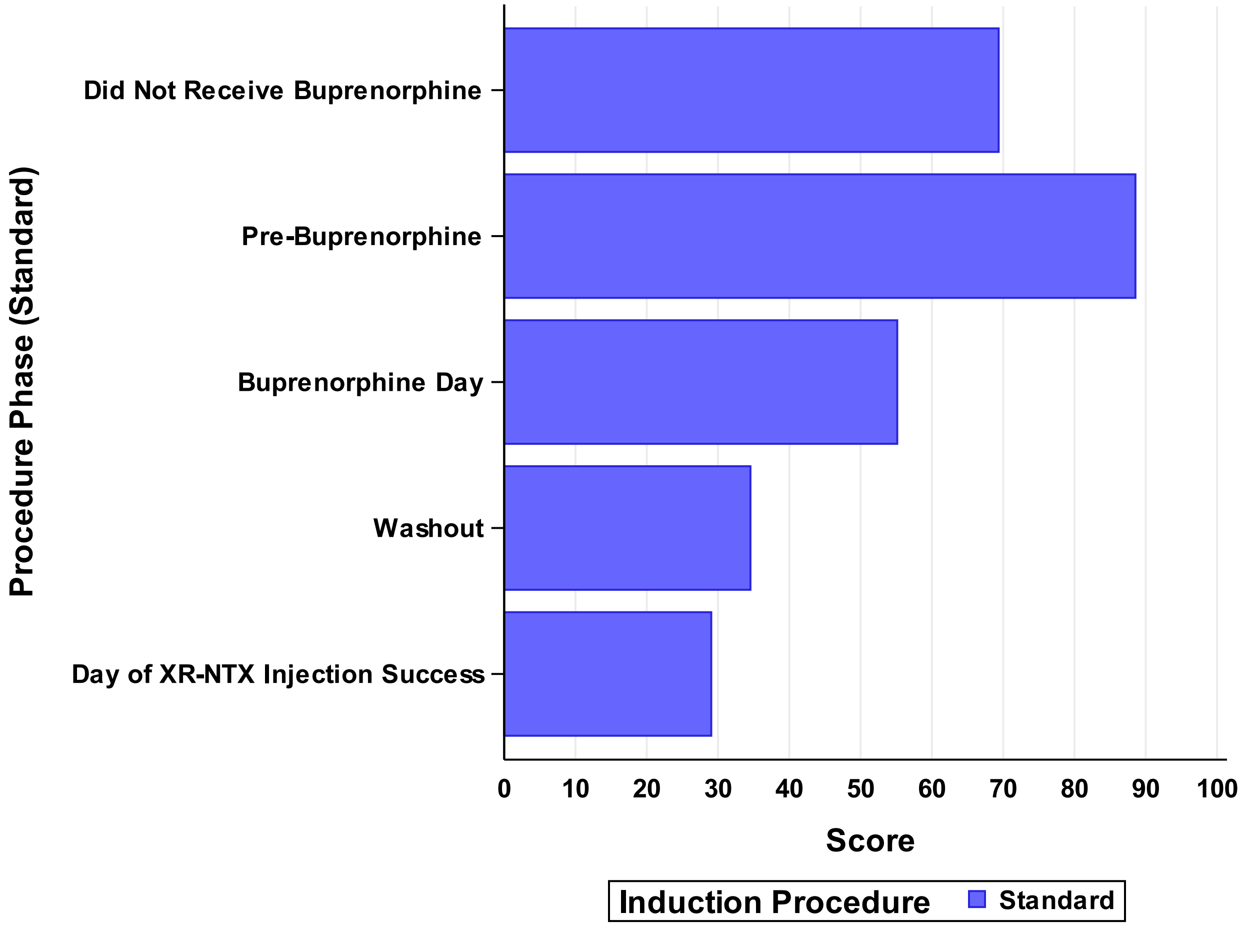

Figure 15: Average Daily Maximum COWS Score in Standard Procedure by Phase

Example figure provided.

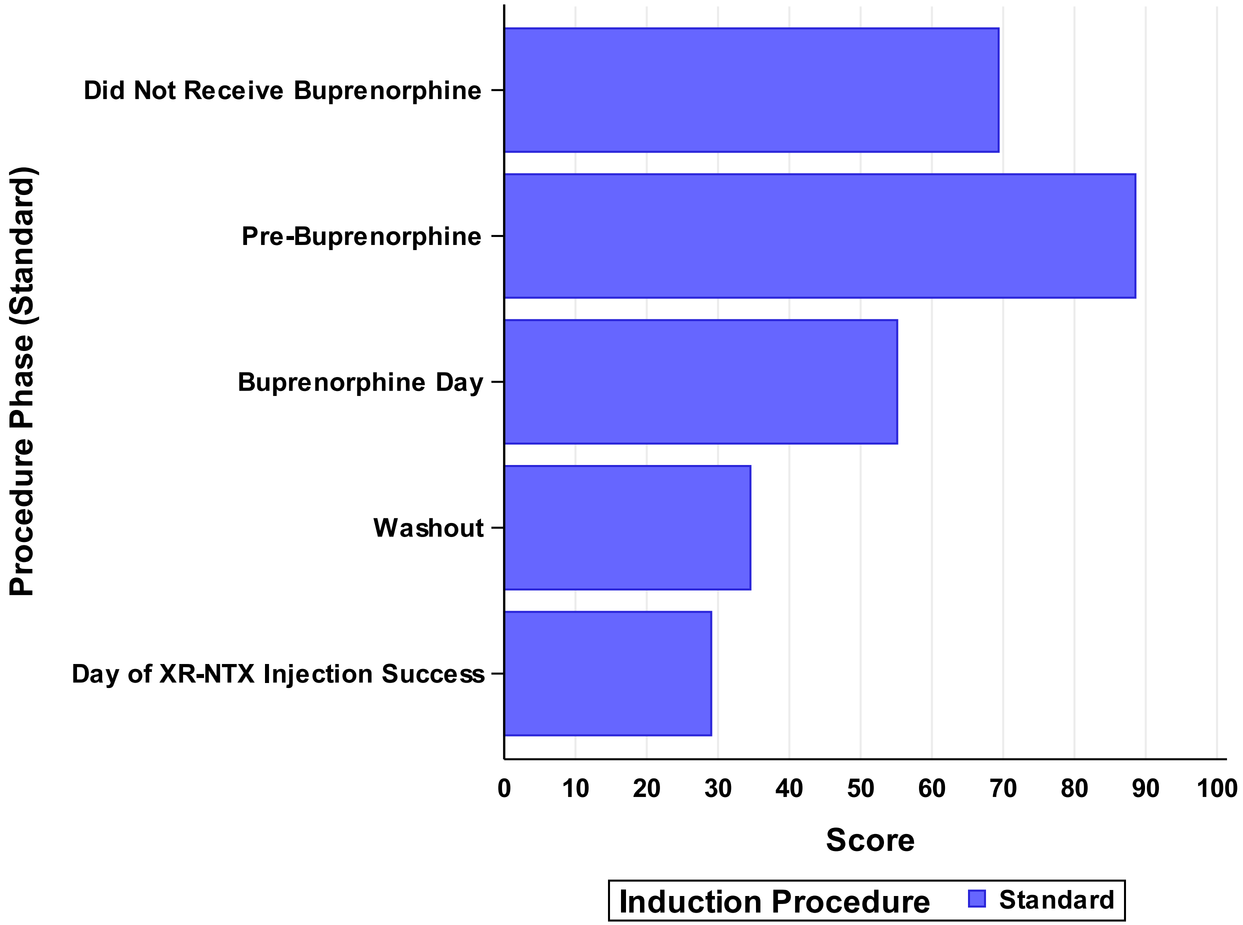

Figure 16: Average Daily COWS Score in Standard Procedure by Phase

Example figure provided.

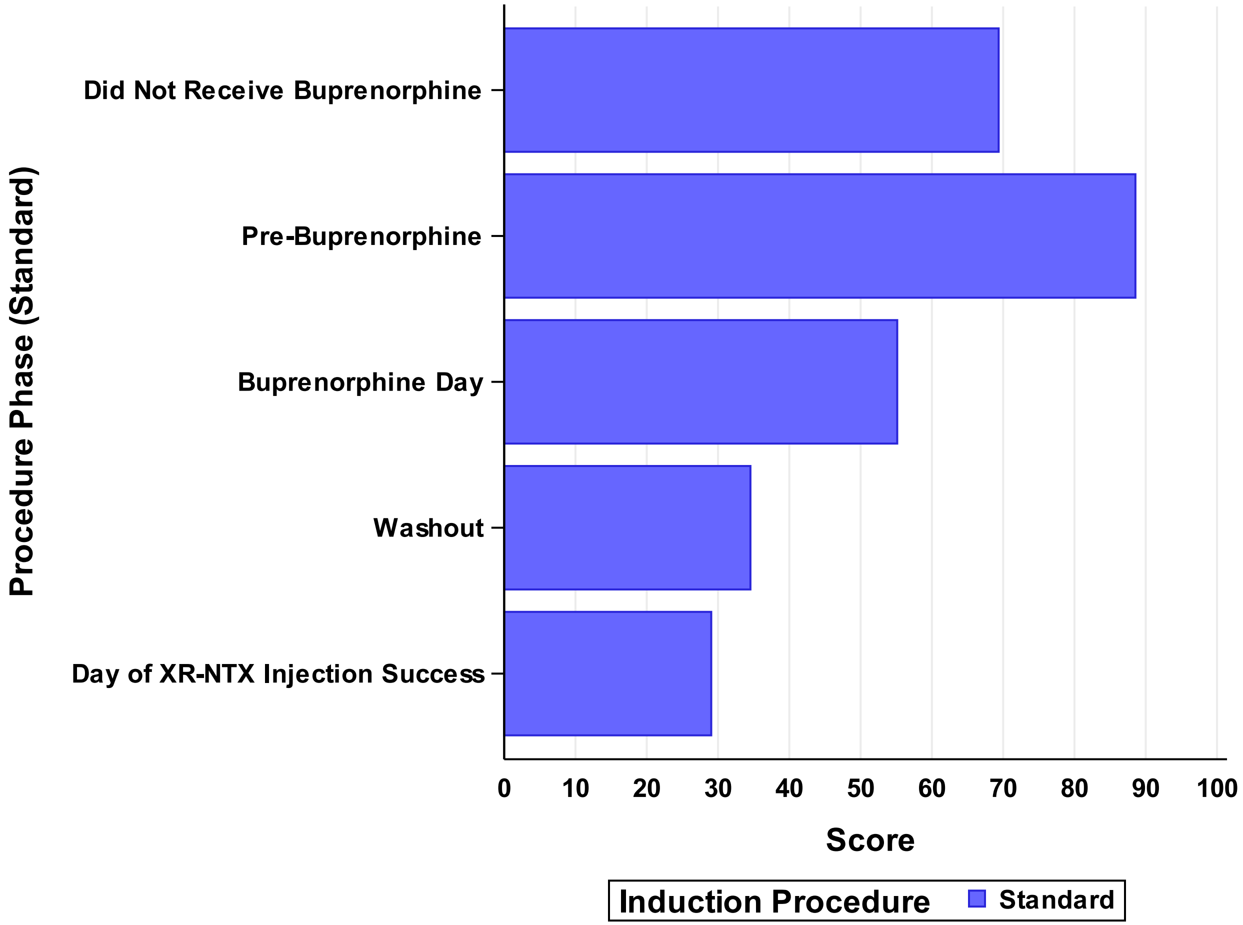

Figure 17: Average Daily SOWS Score in Standard Procedure by Phase

Example figure provided.

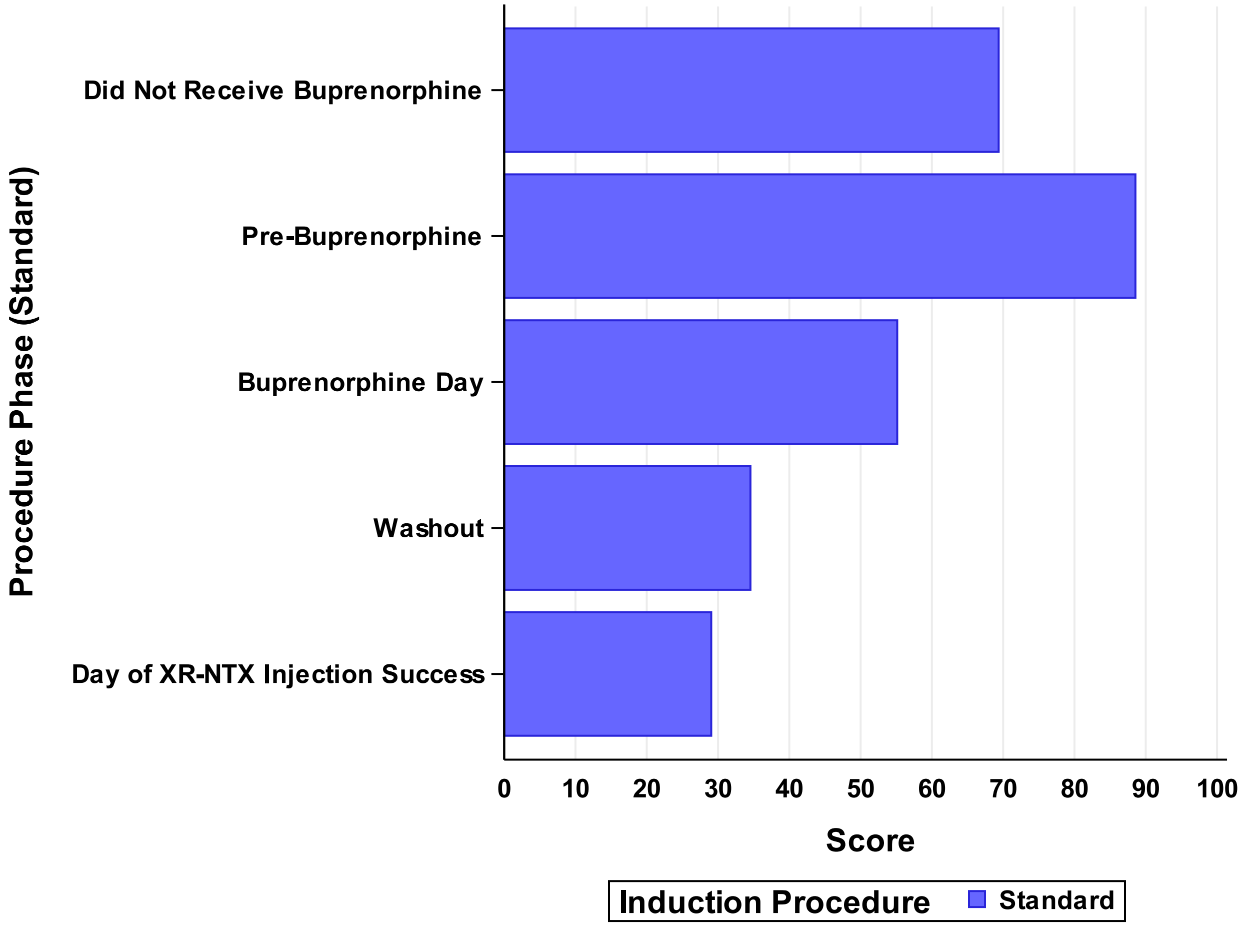

Figure 18: Average VAS Craving Score at Time of Assessment in Standard Procedure by Phase

Example figure provided.

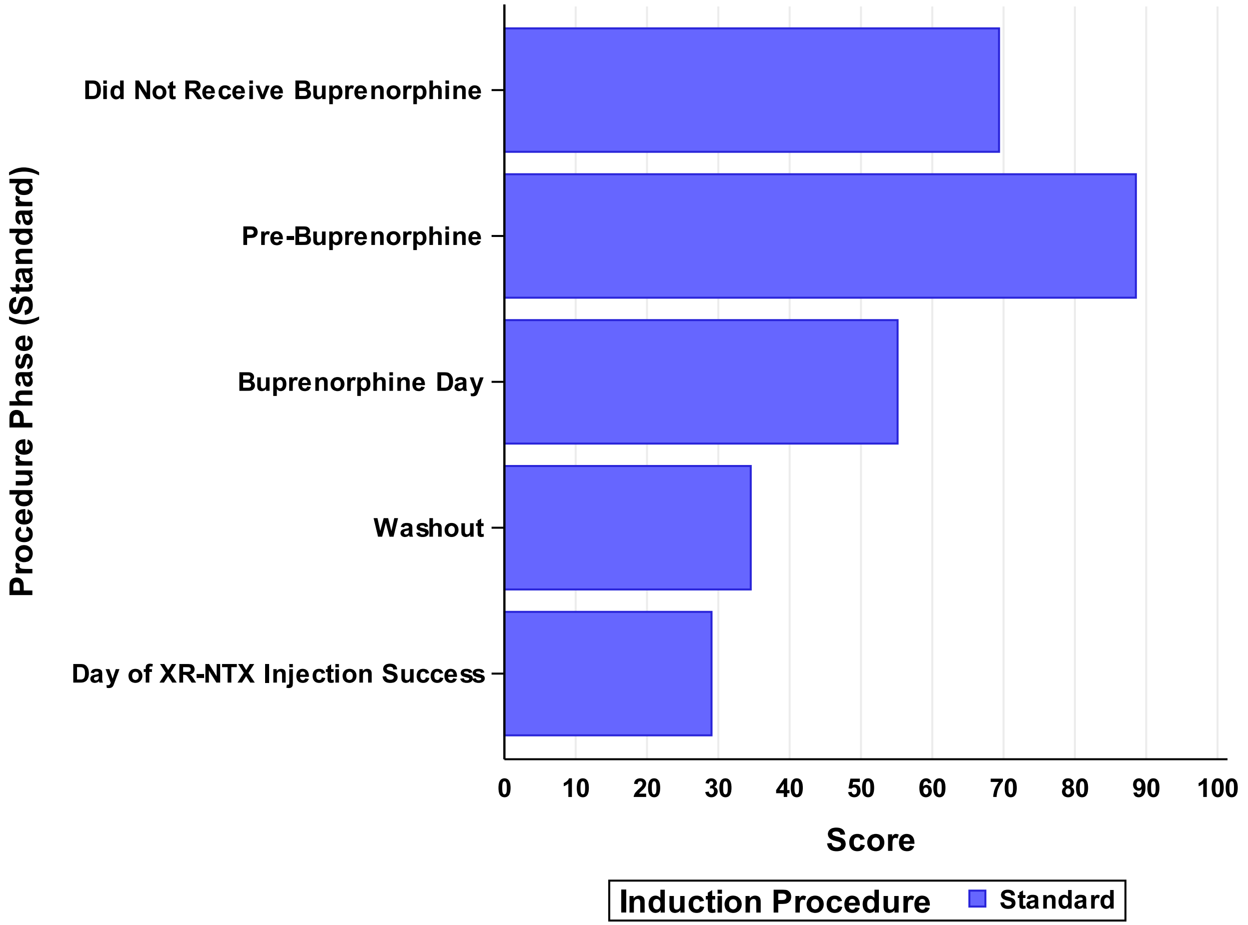

Figure 19: Average Maximum VAS Craving Score within 24 Hours in Standard Procedure by Phase

Example figure provided.

Table 46: Summary of Average Opioid Withdrawal and Craving in Rapid Procedure by Procedure Phase						
(N=XX)						
**Procedure Phase**		**Daily Maximum COWS[1]**	**Daily COWS[1]**	**Daily SOWS[2]**	**Daily Craving[3]**	**Maximum Craving in the past 24 Hours[4]**
Pre-Buprenorphine	Participants with at least one score	N (X.x%)				
	N	N				
	Mean (SD)	X.x (X.xx)				
Buprenorphine Taper	Participants with at least one score	N (X.x%)				
	N	N				
	Mean (SD)	X.x (X.xx)				
Buprenorphine Washout	Participants with at least one score	N (X.x%)				
	N	N				
	Mean (SD)	X.x (X.xx)				
Low Naltrexone Titration	Participants with at least one score	N (X.x%)				
	N	N				
	Mean (SD)	X.x (X.xx)				
Naltrexone Day 1	Participants with at least one score	N (X.x%)				
	N	N				
	Mean (SD)	X.x (X.xx)				
Naltrexone Day #	Participants with at least one score	N (X.x%)				
	N	N				

Table 46: Summary of Average Opioid Withdrawal and Craving in Rapid Procedure by Procedure Phase

(N=XX)

**Procedure Phase**		**Daily Maximum COWS[1]**	**Daily COWS[1]**	**Daily SOWS[2]**	**Daily Craving[3]**	**Maximum Craving in the past 24 Hours[4]**
	Mean (SD)	X.x (X.xx)				
Day of XR-NTX Injection Success	Participants with at least one score	N (X.x%)				
	N	N				
	Mean (SD)	X.x (X.xx)				
Pre XR-NTX injection[5]	Participants with at least one score	N (X.x%)				
	N	N				
	Mean (SD)	X.x (X.xx)				
Post XR-NTX Injection[5]	Participants with at least one score	N (X.x%)				
	N	N				
	Mean (SD)	X.x (X.xx)				
Day of XR-NTX Injection Failure	Participants with at least one score	N (X.x%)				
	N	N				
	Mean (SD)	X.x (X.xx)				

[1] Clinical Opiate Withdrawal Scale.
[2] Subjective Opioid Withdrawal Scale.
[3] Visual Analog Scale for question “Think about your craving for opioids. How intense is it right now?”
[4] Visual Analog Scale for question “Think about your desire to use opioids in the past 24 hours. How intense was your strongest desire to use?”
[5] Only included if time of assessment collected on form. Percentage was calculated based on the number of participants who received XR-NTX injection.

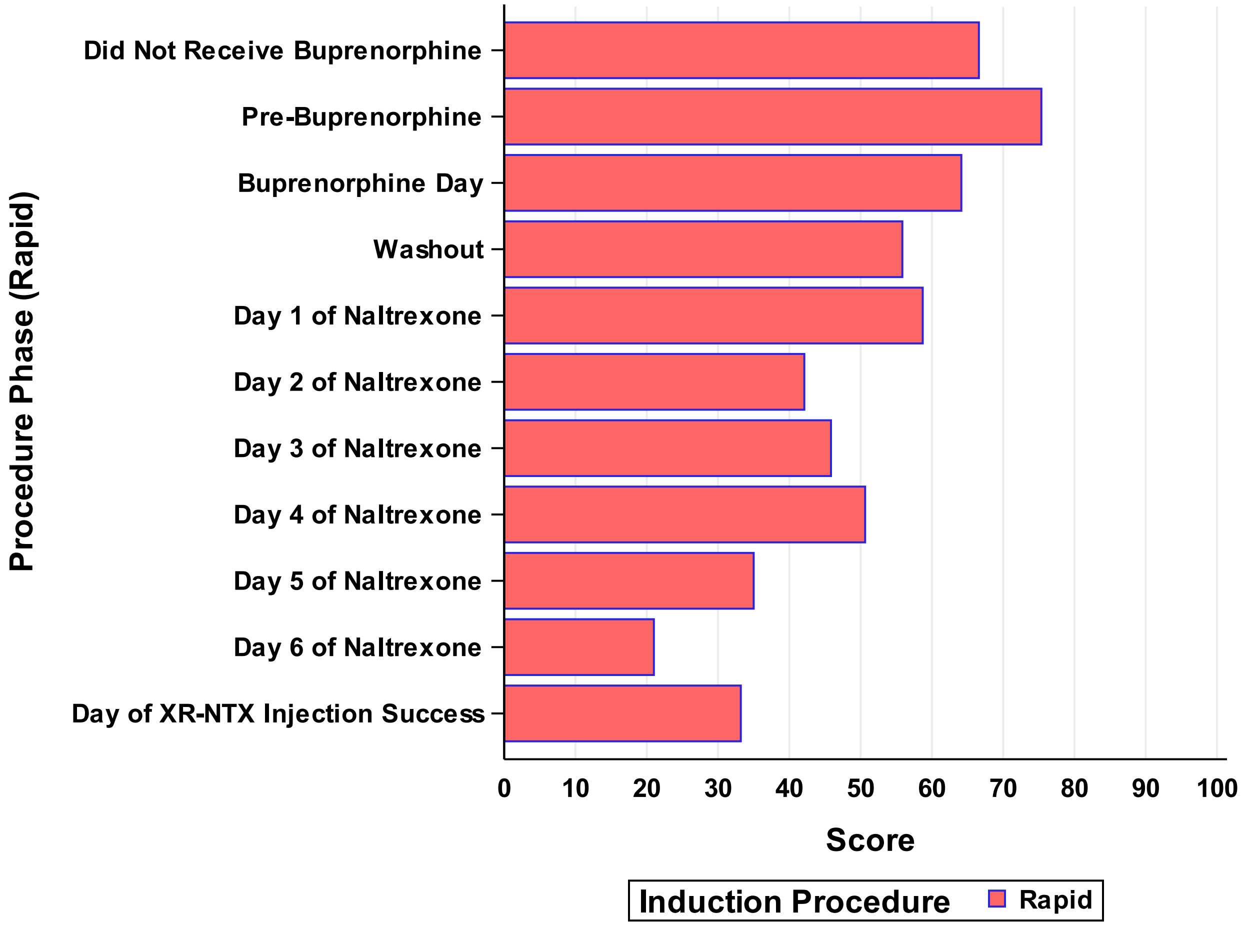

Figure 20: Average Daily Maximum COWS Score in Rapid Procedure by Phase

Example figure provided.

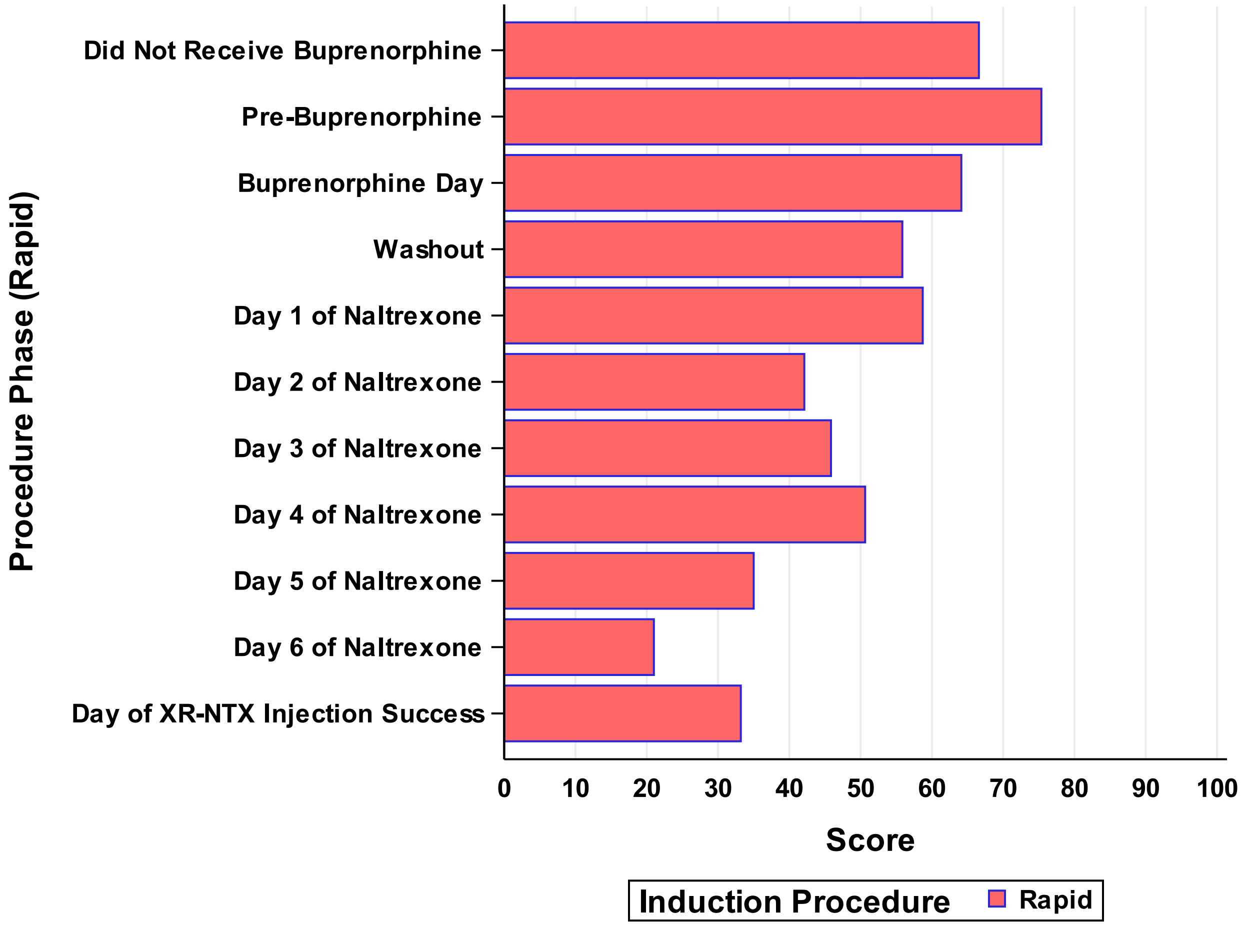

Figure 21: Average Daily COWS Score in Rapid Procedure by Phase

Example figure provided.

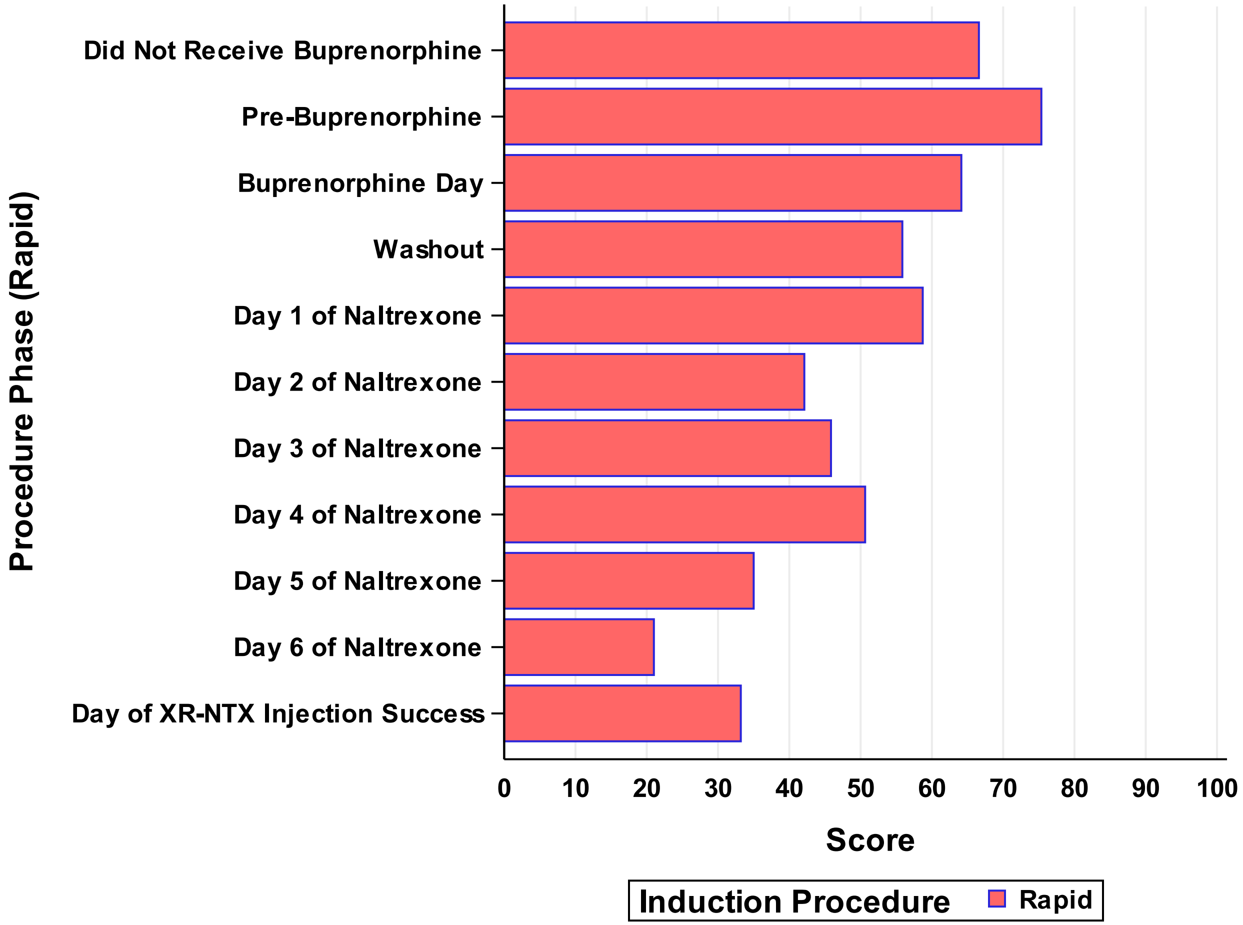

Figure 22: Average Daily SOWS Score in Rapid Procedure by Phase

Example figure provided

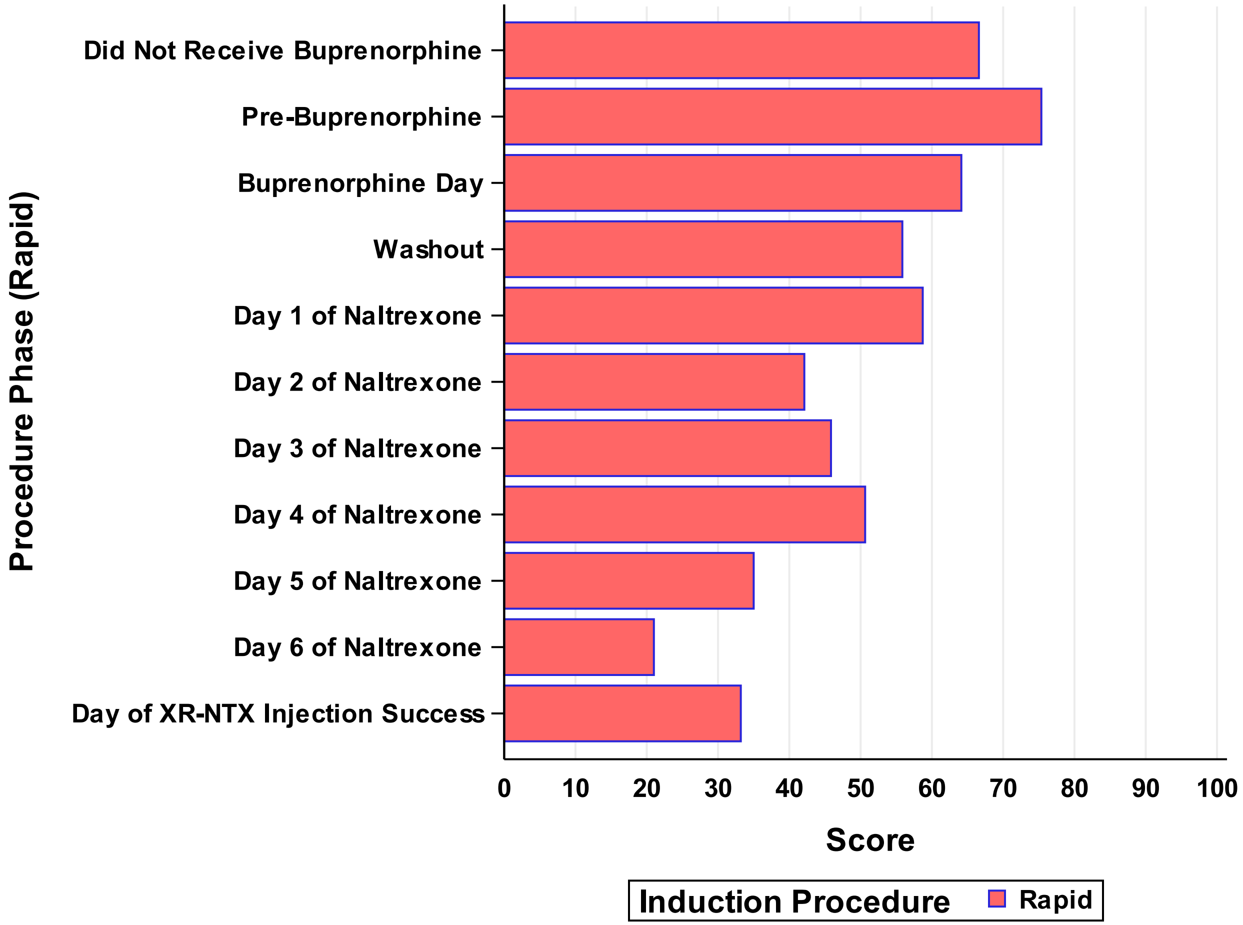

Figure 23: Average VAS Craving Score at Time of Assessment in Rapid Procedure by Phase

Example figure provided.

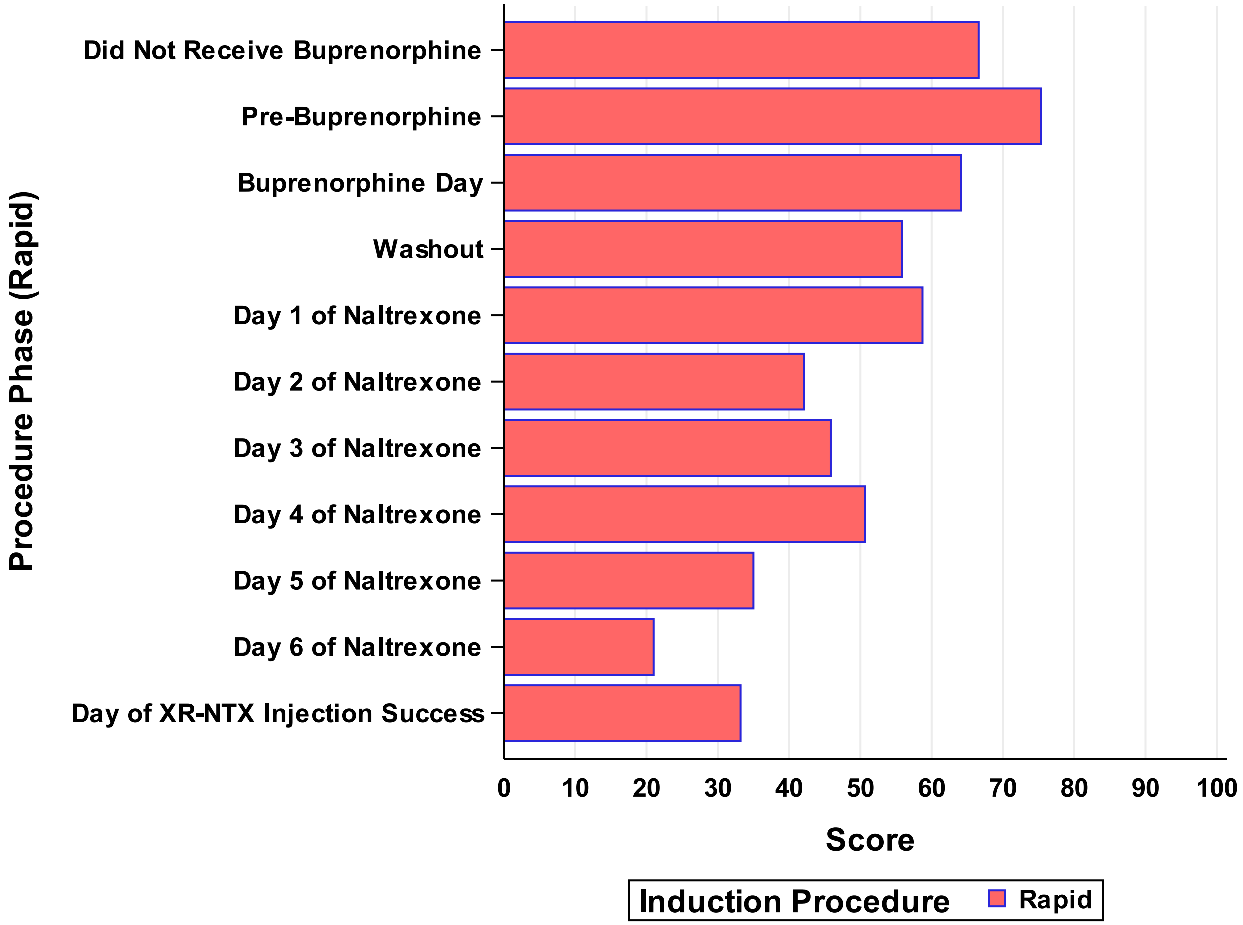

Figure 24: Average Maximum VAS Craving Score within 24 Hours in Rapid Procedure by Phase

Example figure provided.

Table 47: Covariate Adjusted Modeling Results for Opioid Withdrawal as Measured by COWS and SOWS During the Induction Phase

	COWS Daily Maximum				COWS Daily Average				SOWS			
**Effects**	**Odds[1] Ratio**	**95% Lower Confi-dence Limit**	**95% Upper Confi-dence Limit**	**p-value**	**Odds[2] Ratio**	**95% Lower Confi-dence Limit**	**95% Upper Confi-dence Limit**	**p-value**	**Estimate**	**95% Lower Confi-dence Limit**	**95% Upper Confi-dence Limit**	**p-value**
Rapid versus Standard	X.xx	X.xx	X.xx	0.xxx								
Inpatient day												
Nexus Recovery Center versus ADAPT												
Avery Road Treatment Center versus ADAPT												
Aspire Health Partners vs. ADAPT												
Gibson Recovery Center versus ADAPT												
Stony Brook Eastern Long Island Hospital versus ADAPT												

[1] The odds of at least one moderate to severe daily COWS score (maximum score ≥12).
[2] The odds of presence of moderate to severe average daily COWS score (average score ≥12).

Table 48: Covariate Adjusted Modeling Results for Craving for Opioids During the Induction Phase								
	Craving VAS at Time of Assessment				Maximum Craving VAS within 24 Hours			
**Effects**	**Estimate**	**95% Lower Confi-dence Limit**	**95% Upper Confi-dence Limit**	**p-value**	**Estimate**	**95% Lower Confi-dence Limit**	**95% Upper Confi-dence Limit**	**p-value**
Rapid versus Standard	X.xx	X.xx	X.xx	0.xxx				
Inpatient day								
Nexus Recovery Center versus ADAPT								
Avery Road Treatment Center versus ADAPT								
Aspire Health Partners vs. ADAPT								
Gibson Recovery Center versus ADAPT								
Stony Brook Eastern Long Island Hospital versus ADAPT								

Table 49: Retention in the Study to Receive at Least One XR-NTX Injection after Induction Success						
			Results			
**Induction Procedure**	**Number with Induction Success**	**At Least One Additional Injection Administered**	**Odds Ratio**	**95% Lower Confidence Limit**	**95% Upper Confidence Limit**	**p-value**
Rapid	N (X.x%)	N (X.x%)	X.xx	X.xx	X.xx	0.xxx
Standard						
Total						

19.1.9 Safety

Table 50: Summary of Serious Adverse Events by Induction Procedure

	Induction Procedure		
	**Standard (N=XX)**	**Rapid (N=XX)**	**Total (N=XX)**
Number of participants with at least one serious adverse event (SAE)[1]	N (X.x%)		
Maximum severity of SAE for participants with at least one SAE[2]			
Grade 1 - Mild	N (X.x%)		
Grade 2 - Moderate			
Grade 3 - Severe			
Number of participants with at least one SAE related to study medication[1]	N (X.x%)		
Number of participants diagnosed with COVID-19[1]	N (X.x%)		
Number of SAEs	N		
Severity of SAEs[3]			
Grade 1 - Mild	N (X.x%)		
Grade 2 - Moderate			
Grade 3 - Severe			
Relationship of SAE to study medication[3]			
No	N (X.x%)		
Yes			

[1] The percentage was calculated based on the denominator of the number of enrolled participants.
[2] The percentage was calculated with the denominator as the number of enrolled participants with SAEs.
[3] The percentage was calculated with the denominator as the number of SAEs.

Table 51: Summary of MedDRA-coded Serious Adverse Events by Induction Procedure			
	Induction Procedure		
**System Organ Class/Preferred Term (MedDRA V25.0)**	**Standard (N=XX)**	**Rapid (N=XX)**	**Total (N=XX)**
Participants with at least one serious adverse event (SAE)	N (x.x%)		
Injury, poisoning and procedural complications	N (x.x%)		
Overdose	N (x.x%)		
Road traffic accident			
Psychiatric disorders			
Suicidal ideation			
Respiratory, thoracic and mediastinal disorders			
Chronic obstructive pulmonary disease			
Nervous system disorders			
Depressed level of consciousness			
Metabolism and nutrition disorders			
Hypokalaemia			

Percentages were calculated based on the number of participants experiencing the adverse event at least once as the numerator and the number of participants enrolled as the denominator.

Example SOC and preferred terms provided.

Table 52: Summary of Targeted Safety Events by Study Phase and Induction Procedure					
		**Induction Procedure**			
**Study Phase**		**Standard (N=XX)**	**Rapid (N=XX)**	**Total (N=XX)**	**p-Value[8]**
Overall	Number of participants with at least one targeted safety event (TSE)[1]				
	Number of participants with at least one TSE of the following types[2]				
	Fall event[3]				
	Acute change in mental status[4]				
	Acute medical complication likely exacerbated by the stress of withdrawal[5]				
	Acute psychiatric symptoms[6]				
	Number of participants with at least one TSE related to study medication[1]				
	Number of participants with at least one TSE defined as serious adverse event[1]				
	Number of TSEs				
	Type of TSE[7]				
	Fall event[3]				
	Acute change in mental status[4]				
	Acute medical complication likely exacerbated by the stress of withdrawal[5]				
	Acute psychiatric symptoms[6]				
	Relationship of TSE to study medication[7]				
	No				
	Yes				
	TSE defined as serious adverse event[7]				
	No				
	Yes				

Table 52: Summary of Targeted Safety Events by Study Phase and Induction Procedure						
		**Induction Procedure**				
**Study Phase**		**Standard (N=XX)**	**Rapid (N=XX)**	**Total (N=XX)**	**p-Value[8]**
Screening Phase	Number of participants with at least one targeted safety event (TSE)[1]	N (X.x%)			
	Number of participants with at least one TSE of the following types[2]				
	Fall event[3]	N (X.x%)			
	Acute change in mental status[4]				
	Acute medical complication likely exacerbated by the stress of withdrawal[5]				
	Acute psychiatric symptoms[6]				
	Number of participants with at least one TSE related to study medication[1]	N (X.x%)			
	Number of participants with at least one TSE defined as serious adverse event[1]	N (X.x%)			
	Number of TSEs	N			
	Type of TSE[7]				
	Fall event[3]	N (X.x%)			
	Acute change in mental status[4]				
	Acute medical complication likely exacerbated by the stress of withdrawal[5]				
	Acute psychiatric symptoms[6]				
	Relationship of TSE to study medication[7]				
	No	N (X.x%)			
	Yes				
	TSE defined as serious adverse event[7]				
	No	N (X.x%)			
	Yes				

Table 52: Summary of Targeted Safety Events by Study Phase and Induction Procedure					
		**Induction Procedure**			
**Study Phase**		**Standard (N=XX)**	**Rapid (N=XX)**	**Total (N=XX)**	**p-Value[8]**
Induction Phase	Number of participants with at least one targeted safety event (TSE)[1]	N (X.x%)			
	Number of participants with at least one TSE of the following types[2]				
	Fall event[3]	N (X.x%)			
	Acute change in mental status[4]				
	Acute medical complication likely exacerbated by the stress of withdrawal[5]				
	Acute psychiatric symptoms[6]				
	Number of participants with at least one TSE related to study medication[1]	N (X.x%)			
	Number of participants with at least one TSE defined as serious adverse event[1]	N (X.x%)			
	Number of TSEs	N			
	Type of TSE[7]				
	Fall event[3]	N (X.x%)			
	Acute change in mental status[4]				
	Acute medical complication likely exacerbated by the stress of withdrawal[5]				
	Acute psychiatric symptoms[6]				
	Relationship of TSE to study medication[7]				
	No	N (X.x%)			
	Yes				
	TSE defined as serious adverse event[7]				
	No	N (X.x%)			
	Yes				

Table 52: Summary of Targeted Safety Events by Study Phase and Induction Procedure					
		Induction Procedure			
Study Phase		Standard (N=XX)	Rapid (N=XX)	Total (N=XX)	p-Value[8]
Post-Induction Phase	Number of participants with at least one targeted safety event (TSE)[1]	N (X.x%)			
	Number of participants with at least one TSE of the following types[2]				
	Fall event[3]	N (X.x%)			
	Acute change in mental status[4]				
	Acute medical complication likely exacerbated by the stress of withdrawal[5]				
	Acute psychiatric symptoms[6]				
	Number of participants with at least one TSE related to study medication[1]	N (X.x%)			
	Number of participants with at least one TSE defined as serious adverse event[1]	N (X.x%)			
	Number of TSEs	N			
	Type of TSE[7]				
	Fall event[3]	N (X.x%)			
	Acute change in mental status[4]				
	Acute medical complication likely exacerbated by the stress of withdrawal[5]				
	Acute psychiatric symptoms[6]				
	Relationship of TSE to study medication[7]				
	No	N (X.x%)			
	Yes				
	TSE defined as serious adverse event[7]				
	No	N (X.x%)			
	Yes				

[1] The percentage was calculated based on the denominator of the number of enrolled participants.
[2] The percentage was calculated with the denominator as the number of participants with TSEs.
[3] Likely related to medical/psychiatric condition such as dizziness, confusion with head injury.
[4] Disorientation, amnesia, cerebrovascular accident, coma.
[5] Hypertensive crisis, hypotensive event with medical sequelae such as fall and/or requiring urgent fluid resuscitation, severe chest pain, cardiac arrhythmia, acute respiratory decompensation, asthma attack, diabetic ketoacidosis, severe hypoglycemia.

[6] Acute psychiatric symptoms (i.e., psychosis, hypomania, severe agitation, violence).
[7] The percentage was calculated with the denominator as the number of TSEs.
[8] p-value is from Fisher's Exact Test.

Table 53: Summary of Study Injection Site Examinations

	Induction Procedure		
	**Standard (N=XX)**	**Rapid (N=XX)**	**Total (N=XX)**
Number of participants with at least one abnormal injection site[1]	N (X.x%)		
Number of abnormal injection sites	N		
Symptom experienced[2]			
None	N (X.x%)		
Pain			
Tenderness			
Erythema/Redness			
Swelling			
Induration			
Abscess			
Necrosis			
Bruising			
Pruritus			
Nodule			
Hematoma			
Sterile abscess			
Cellulitis			
Warmth			
Other			
Severity[2]			
Grade 1 - Mild	N (X.x%)		
Grade 2 - Moderate			
Grade 3 - Severe			

[1] Percentages were calculated based on number of enrolled participants.
[2] Percentages were calculated based on number of abnormal injection sites.

Table 54: Summary of Non-Fatal Opioid Overdoses by Study Phase and Induction Procedure				
		**Induction Procedure**		
**Study Phase**		**Standard (N=XX)**	**Rapid (N=XX)**	**Total (N=XX)**
Baseline (i.e., before enrollment in the study)	Number of participants with at least one overdose	N (X.x%)		
	Receive NARCAN (naloxone) at home to reverse overdose[1,2]			
	No	N (X.x%)		
	Yes			
	Overdose resulting in hospitalization[1,2]			
	No	N (X.x%)		
	Yes			
	Before the overdose, how likely was it that you would overdose[2,3]			
	N	N		
	Mean	X.X		
	SD	X.XX		
	Minimum	X		
	25th Percentile	X.X		
	Median	X		
	75th Percentile	X.X		
	Maximum	X		
	Before the overdose, how strongly did you want to die[2,3]			
	N	N		
	Mean	X.X		
	SD	X.XX		
	Minimum	X		
	25th Percentile	X.X		
	Median	X		
	75th Percentile	X.X		
	Maximum	X		
	At the time of the overdose, were you trying to kill yourself [2,4]			
	N	N		
	Mean	X.X		
	SD	X.XX		
	Minimum	X		

Table 54: Summary of Non-Fatal Opioid Overdoses by Study Phase and Induction Procedure

Study Phase		Induction Procedure		Total
		Standard (N=XX)	Rapid (N=XX)	(N=XX)
	25th Percentile	X.X		
	Median	X		
	75th Percentile	X.X		
	Maximum	X		
	How likely would you be to seek out certain opioids that resulted in fatal overdoses in your area[5]			
	N	N		
	Mean	X.X		
	SD	X.XX		
	Minimum	X		
	25th Percentile	X.X		
	Median	X		
	75th Percentile	X.X		
	Maximum	X		

Table 54: Summary of Non-Fatal Opioid Overdoses by Study Phase and Induction Procedure				
		**Induction Procedure**		
**Study Phase**		**Standard (N=XX)**	**Rapid (N=XX)**	**Total (N=XX)**
Day 1 to Day 28 Post-Induction Period	Number of participants with at least one overdose	N (X.x%)		
	Receive NARCAN (naloxone) at home to reverse overdose[1,2]			
	No	N (X.x%)		
	Yes			
	Overdose resulting in hospitalization[1,2]			
	No	N (X.x%)		
	Yes			
	Before the overdose, how likely was it that you would overdose[2,3]			
	N	N		
	Mean	X.X		
	SD	X.XX		
	Minimum	X		
	25th Percentile	X.X		
	Median	X		
	75th Percentile	X.X		
	Maximum	X		
	Before the overdose, how strongly did you want to die[2,4]			
	N	N		
	Mean	X.X		
	SD	X.XX		
	Minimum	X		
	25th Percentile	X.X		
	Median	X		
	75th Percentile	X.X		
	Maximum	X		
	At the time of the overdose, were you trying to kill yourself [2,5]			
	N	N		
	Mean	X.X		
	SD	X.XX		
	Minimum	X		

Table 54: Summary of Non-Fatal Opioid Overdoses by Study Phase and Induction Procedure

Study Phase		Induction Procedure		Total
		Standard (N=XX)	Rapid (N=XX)	(N=XX)
	25th Percentile	X.X		
	Median	X		
	75th Percentile	X.X		
	Maximum	X		
	How likely would you be to seek out certain opioids that resulted in fatal overdoses in your area[5]			
	N	N		
	Mean	X.X		
	SD	X.XX		
	Minimum	X		
	25th Percentile	X.X		
	Median	X		
	75th Percentile	X.X		
	Maximum	X		

Table 54: Summary of Non-Fatal Opioid Overdoses by Study Phase and Induction Procedure				
		**Induction Procedure**		
**Study Phase**		**Standard (N=XX)**	**Rapid (N=XX)**	**Total (N=XX)**
After Day 28 to Day 56 Post-Induction period	Number of participants with at least one overdose	N (X.x%)		
	Receive NARCAN (naloxone) at home to reverse overdose[1,2]			
	No	N (X.x%)		
	Yes			
	Overdose resulting in hospitalization[1,2]			
	No	N (X.x%)		
	Yes			
	Before the overdose, how likely was it that you would overdose[2,3]			
	N	N		
	Mean	X.X		
	SD	X.XX		
	Minimum	X		
	25th Percentile	X.X		
	Median	X		
	75th Percentile	X.X		
	Maximum	X		
	Before the overdose, how strongly did you want to die[2,4]			
	N	N		
	Mean	X.X		
	SD	X.XX		
	Minimum	X		
	25th Percentile	X.X		
	Median	X		
	75th Percentile	X.X		
	Maximum	X		
	At the time of the overdose, were you trying to kill yourself [2,5]			
	N	N		
	Mean	X.X		
	SD	X.XX		
	Minimum	X		

Table 54: Summary of Non-Fatal Opioid Overdoses by Study Phase and Induction Procedure

Study Phase		Induction Procedure		Total
		Standard (N=XX)	Rapid (N=XX)	(N=XX)
	25th Percentile	X.X		
	Median	X		
	75th Percentile	X.X		
	Maximum	X		
	How likely would you be to seek out certain opioids that resulted in fatal overdoses in your area[5]			
	N	N		
	Mean	X.X		
	SD	X.XX		
	Minimum	X		
	25th Percentile	X.X		
	Median	X		
	75th Percentile	X.X		
	Maximum	X		

[1] Percentages were calculated based on number of participants with at least one overdose during the timeframe specified in the first column.
[2] For the most recent opioid overdoses per participant during the timeframe specified in the first column.
[3] This question was asked on a scale between 0 (no chance) and 10 (extremely likely).
[4] This question was asked on a scale between 0 (did not want to die) and 10 (definitely wanted to die).
[5] These questions were asked on a scale between 0 (not at all) and 10 (definitely).

Table 55: Summary of Suicide Risk by Induction Procedure

	Induction Procedure		
	Standard (N=190)	Rapid (N=225)	Total (N=415)
Number endorsing suicide risk on PHQ-9[1] at baseline	76 (40.0%)	85 (37.8%)	161 (38.8%)
Number endorsing suicide risk on PHQ-9[1] during induction phase	34 (17.9%)	27 (12.0%)	61 (14.7%)
Number endorsing suicide risk on PHQ-9[1] during post-induction phase	20 (10.5%)	38 (16.9%)	58 (14.0%)
Total[2]	91 (47.9%)	103 (45.8%)	194 (46.7%)

[1] Patient Health Questionnaire-9. Endorsing suicide risk on PHQ-9 is defined as a response of anything other than 'Not at all' on question 9: Over the last 2 weeks, how often have you been bothered by any of the following problems: Thoughts that you would be better off dead, or of hurting yourself in some way.
[2] Total refers to the total number of unique participants who endorsed suicide risk on PHQ-9 at least once at baseline, during the induction phase or during the post-induction phase.

Listing 1: Serious Adverse Events by Induction Procedure

Induction Procedure = Standard

										MedDRA V25.0	
**Site**	**Participant ID**	**Date of Enrollment**	**Onset Date**	**AE Description**	**Severity of AE**	**Relatedness to Study Medication**	**Outcome**	**Date of Resolution/ Death**	**SAE Associated With**	**Preferred Term**	**System Organ Class**
xxxxxxxxxx	xxxxxxxxxxxxxx	mm/dd/yyyy	mm/dd/yyyy	xxxxxxxxxx	xxxxxxxx	xxxxxxxxxx	xxxxxxxxxx	mm/dd/yyyy	xxxxxxxxxx	xxxxxxxxxx	xxxxxxxxxx

Listing 1: Serious Adverse Events by Induction Procedure

Induction Procedure = Rapid

										MedDRA V25.0	
**Site**	**Participant ID**	**Date of Enrollment**	**Onset Date**	**AE Description**	**Severity of AE**	**Relatedness to Study Medication**	**Outcome**	**Date of Resolution/ Death**	**SAE Associated With**	**Preferred Term**	**System Organ Class**
xxxxxxxxxx	xxxxxxxxxxxxxx	mm/dd/yyyy	mm/dd/yyyy	xxxxxxxxxx	xxxxxxxxxx	xxxxxxxxxx	xxxxxxxxxx	mm/dd/yyyy	xxxxxxxxxx	xxxxxxxxxx	xxxxxxxxxx

Listing 2: Deaths by Induction Procedure								
Induction Procedure = Standard								
**Site**	**Participant ID**	**Date of Enrollment**	**Date of Death**	**Source of Death Report**	**Type**	**Cause of Death**	**Contributing Factors**	**Short Narrative**
xxxxxxxxxx	xxxxxxxxxxxxxxx	mm/dd/yyyy	mm/dd/yyyy	Medical chart/ Death certificate/ Autopsy report/ Treating physician/ Other	Primary	xxxxxxxxxx	xxxxxxxxxx	xxxxxxxxxx
					Secondary	xxxxxxxxxx	xxxxxxxxxx	xxxxxxxxxx

Listing 3: Deaths by Induction Procedure								
Induction Procedure = Standard								
**Site**	**Participant ID**	**Date of Enrollment**	**Date of Death**	**Source of Death Report**	**Type**	**Cause of Death**	**Contributing Factors**	**Short Narrative**
xxxxxxxxxx	xxxxxxxxxxxxxxx	mm/dd/yyyy	mm/dd/yyyy	Medical chart/ Death certificate/ Autopsy report/ Treating physician/ Other	Primary	xxxxxxxxxx	xxxxxxxxxx	xxxxxxxxxx
					Secondary	xxxxxxxxxx	xxxxxxxxxx	xxxxxxxxxx

Listing 4: Targeted Safety Events by Induction Procedure										
**Induction Procedure = Standard**										
**Site**	**Participant ID**	**Study Phase**	**Date of Enrollment**	**Date of TSE[1]**	**TSE[1] Description**	**TSE[1] Details**	**Severity of TSE[1]**	**Relatedness to Study Medication**	**Serious Adverse Event**	**Comments**
xxxxxxxxxx	xxxxxxxxxxxxx	Screening/ Induction/ Post-Induction	mm/dd/yyyy	mm/dd/yyyy	xxxxxxxxxx	xxxxxxxxxx	Mild/ Moderate/ Severe	Yes/No	Yes/No	xxxxxxxxx

[1] Targeted Safety Event.

Listing 3: Targeted Safety Events by Induction Procedure										
**Induction Procedure = Rapid**										
**Site**	**Participant ID**	**Study Phase**	**Date of Enrollment**	**Date of TSE[1]**	**TSE[1] Description**	**TSE[1] Details**	**Severity of TSE[1]**	**Relatedness to Study Medication**	**Serious Adverse Event**	**Comments**
xxxxxxxxxx	xxxxxxxxxxxxx	Screening/ Induction/ Post-Induction	mm/dd/yyyy	mm/dd/yyyy	xxxxxxxxxx	xxxxxxxxxx	Mild/ Moderate/ Severe	Yes/No	Yes/No	xxxxxxxxx

[1] Targeted Safety Event.

**Listing 4: Injection Site Abnormalities by Induction Procedure**								
**Induction Procedure = Standard**								
**Site**	**Participant ID**	**Date of Enrollment**	**Injection Number**	**Event Start Date**	**Event Resolution Date**	**Symptom**	**Severity**	**SAE**
xxxxxxxxxx	xxxxxxxxxxxxxxx	mm/dd/yyyy	1, 2 ,3	mm/dd/yyyy	mm/dd/yyyy	xxxxxxxx	Mild/ Moderate/ Severe	Yes/No

**Listing 4: Injection Site Abnormalities by Induction Procedure**								
**Induction Procedure = Rapid**								
**Site**	**Participant ID**	**Date of Enrollment**	**Injection Number**	**Event Start Date**	**Event Resolution Date**	**Symptom**	**Severity**	**SAE**
xxxxxxxxxx	xxxxxxxxxxxxxxx	mm/dd/yyyy	1, 2 ,3	mm/dd/yyyy	mm/dd/yyyy	xxxxxxxx	Mild/ Moderate/ Severe	Yes/No

Listing 5: Non-Fatal Opioid Overdoses by Induction Procedure

Induction Procedure = Standard

Site	Participant ID	Date of Enrollment	Visit	Date of Assessment	Have You Over-dosed[1]?	Most Recent Overdose[1]						Interest in Fatal Opioids[3]	Comments
						Used NARCAN	Hospital Admission	Substances Used	Likelihood to Overdose[2]	Kill Yourself[3]	Want to Die[4]		
xxxxxxxxxx	xxxxxxxxxxxxxxx	mm/dd/yyyy	Baseline/ Day 7 Follow-up/ Day 56 Follow-up	mm/dd/yyyy	Yes/No	Yes/No	Yes/No	[Concatenated all substances used separated by commas]	N	N	N	N	xxxxxxxxxx

[1] For baseline visits, this refers to overdoses up to and including date of assessment; for post-induction visits, this refers to overdoses since last visit.
[2] This question was asked on a scale between 0 (no chance) and 10 (extremely likely).
[3] These questions were asked on a scale between 0 (not at all) and 10 (definitely).
[4] This question was asked on a scale between 0 (did not want to die) and 10 (definitely wanted to die).

Listing 5: Non-Fatal Opioid Overdoses by Induction Procedure

Induction Procedure = Rapid

Site	Participant ID	Date of Enrollment	Visit	Date of Assessment	Have You Over-dosed[1]?	Most Recent Overdose[1]						Interest in Fatal Opioids[2]	Comments
						Used NARCAN	Hospital Admission	Substances Used	Likelihood to Overdose[2]	Kill Yourself[2]	Want to Die[2]		
xxxxxxxx	xxxxxxxxxxxxxxx	mm/dd/yyyy	Baseline/ Day 7 Follow-up/ Day 56 Follow-up	mm/dd/yyyy	Yes/No	Yes/No	Yes/No	[Concatenated all substances used separated by commas]	N	N	N	N	xxxxxxxxxx

[1] For baseline visits, this refers to overdoses up to and including date of assessment; for post-induction visits, this refers to overdoses since last visit.
[2] These questions were asked on a scale between 0 (not at all) and 10 (most likely).

Listing 6: Suicide Risk by Induction Procedure

Induction Procedure = Standard

Site	Participant ID	Date of Enrollment	Visit	Date of Assessment	Over the last 2 weeks, how often have you been bothered by thoughts that you would be better off dead, or of hurting yourself in some way?[1]
xxxxxxxxxx	xxxxxxxxxxxxxxx	mm/dd/yyyy	Baseline/Inpatient Day 1 /…/Day 30<br>Day 7 Follow-up/…/Day 56 Follow-up	mm/dd/yyyy	Not at all/<br>Several days/<br>More than half the days/<br>Nearly every day

Note: All visits are included for participants who answered anything other than 'Not at all' at one visit at least. Responses of 'Several days' are highlighted in yellow, 'More than half the days' are highlighted in orange, and 'Nearly every day' are highlighted in red.

[1] This question was asked as part of the Patient Health Questionnaire (PHQ-9) administered at baseline, induction, and post-induction.

Listing 6: Suicide Risk by Induction Procedure

Induction Procedure = Rapid

Site	Participant ID	Date of Enrollment	Visit	Date of Assessment	Over the last 2 weeks, how often have you been bothered by thoughts that you would be better off dead, or of hurting yourself in some way?[1]
xxxxxxxxxx	xxxxxxxxxxxxxxx	mm/dd/yyyy	Baseline/Inpatient Day 1 /…/Inpatient Day 30<br>Day 7 Follow-up/…/Day 56 Follow-up	mm/dd/yyyy	Not at all/<br>Several days/<br>More than half the days/<br>Nearly every day

Note: All visits are included for participants who answered anything other than 'Not at all' at one visit at least. Responses of 'Several days' are highlighted in yellow, 'More than half the days' are highlighted in orange, and 'Nearly every day' are highlighted in red.

[1] This question is asked as part of the Patient Health Questionnaire (PHQ-9) administered at baseline, induction, and post-induction.

Listing 7: Pregnancies by Induction Procedure							
Induction Procedure = Standard							
Site	Participant ID	Date of Enrollment	Date Staff Aware of Pregnancy	Date Pregnancy Confirmed	Date of Pregnancy Outcome	Pregnancy Outcome	Action Taken with Study Medication
xxxxxxxxxx	xxxxxxxxxxxxxxx	mm/dd/yyyy	mm/dd/yyyy	mm/dd/yyyy	mm/dd/yyyy	xxxxxx	xxxxxxxxxxxxxxx

Listing 7: Pregnancies by Induction Procedure							
Induction Procedure = Rapid							
Site	Participant ID	Date of Enrollment	Date Staff Aware of Pregnancy	Date Pregnancy Confirmed	Date of Pregnancy Outcome	Pregnancy Outcome	Action Taken with Study Medication
xxxxxxxxxx	xxxxxxxxxxxxxxx	mm/dd/yyyy	mm/dd/yyyy	mm/dd/yyyy	mm/dd/yyyy	xxxxxx	xxxxxxxxxxxxxxx

19.1.10 Data Quality

Table 56: Summary of Data Audits by Site

Site	**Date of Audit**	**Total Fields Audited[1]**	**Total Data Discrepancies[2]**	**Error Rate**
Gibson Recovery Center	mm/dd/yyyy	N	N	x.xx%
	Subtotal			
Nexus Recovery Center	mm/dd/yyyy			
	Subtotal			
Stony Brook Eastern Long Island Hospital	mm/dd/yyyy			
	Subtotal			
Aspire Health Partners	mm/dd/yyyy			
	Subtotal			
Avery Road Treatment Center	mm/dd/yyyy			
	Subtotal			
ADAPT	mm/dd/yyyy			
	Subtotal			
Total	-			

[1] Fields reviewed at monitoring visit comparing the databases to source documentation.
[2] Fields discrepant between database and source documentation.

Table 57: Summary of Protocol Deviations by Site							
	Gibson Recovery Center	Nexus Recovery Center	Stony Brook Eastern Long Island Hospital	Aspire Health Partners	Avery Road Treatment Center	ADAPT	Total
Total number of protocol deviations	N						
Number of protocol deviations related to COVID-19	N (x%)						
Number of participants impacted per protocol deviation							
None	N (x%)						
One							
More than one							
Total number of major protocol deviations	N						
Number of major protocol deviations related to COVID-19	N (x%)						
Type of major protocol deviation							
No consent/assent obtained	N (x%)						
Unauthorized assessments and/or procedures conducted prior to obtaining informed consent/assent							
Non-IRB approved/outdated/obsolete informed consent/assent documents used							
Other major informed consent/assent procedures issues							
Other informed consent/assent procedures issues							
Ineligible participant enrolled/inclusion/exclusion criteria not met or eligibility not fully assessed prior to enrollment							
Other inclusion/exclusion criteria issues							
Other laboratory assessment issues							
Study assessment/procedures not followed in accordance with study protocol							
Other study procedures/assessments issues							

Table 57: Summary of Protocol Deviations by Site

	Gibson Recovery Center	Nexus Recovery Center	Stony Brook Eastern Long Island Hospital	Aspire Health Partners	Avery Road Treatment Center	ADAPT	Total
AE not reported							
SAE not reported							
AE/SAE reported out of protocol specified reporting timeframe							
AE/SAE not elicited, observed and/or documented as per protocol							
Safety assessment (e.g., labs, ECG, clinical referral to care) not conducted per protocol							
Other adverse events issues							
Stratification error							
Other randomization procedures issues							
Medication not dispensed/administered in accordance with the study protocol							
Participant use of protocol prohibited medication							
Other study medication management issues							
Destruction of study materials without prior authorization from sponsor							
Breach of Confidentiality							
Other significant deviations issues							
Total number of minor protocol deviations	N						
Number of minor protocol deviations related to COVID-19	N (x%)						
Type of minor protocol deviation							
No consent/assent obtained	N (x%)						
Unauthorized assessments and/or procedures conducted prior to obtaining informed consent/assent							

Table 57: Summary of Protocol Deviations by Site

	Gibson Recovery Center	Nexus Recovery Center	Stony Brook Eastern Long Island Hospital	Aspire Health Partners	Avery Road Treatment Center	ADAPT	Total
Non-IRB approved/outdated/obsolete informed consent/assent documents used							
Other major informed consent/assent procedures issues							
Other informed consent/assent procedures issues							
Ineligible participant enrolled/inclusion/exclusion criteria not met, or eligibility not fully assessed prior to enrollment							
Other inclusion/exclusion criteria issues							
Other laboratory assessment issues							
Study assessment/procedures not followed in accordance with study protocol							
Other study procedures/assessments issues							
AE not reported							
SAE not reported							
AE/SAE reported out of protocol specified reporting timeframe							
AE/SAE not elicited, observed and/or documented as per protocol							
Safety assessment (e.g., labs, ECG, clinical referral to care) not conducted per protocol							
Other adverse events issues							
Stratification error							
Other randomization procedures issues							
Medication not dispensed/administered in accordance with the study protocol							
Participant use of protocol prohibited medication							
Other study medication management issues							

Table 57: Summary of Protocol Deviations by Site

	Gibson Recovery Center	Nexus Recovery Center	Stony Brook Eastern Long Island Hospital	Aspire Health Partners	Avery Road Treatment Center	ADAPT	Total
Destruction of study materials without prior authorization from sponsor							
Breach of Confidentiality							
Other significant deviations issues							

Listing 8: Protocol Deviations

Deviation Category = Informed Consent Procedures

Site	Participant IDs	Date of Protocol Deviation	Date Protocol Deviation Entered in EDC	Deviation Type	Reason for Protocol Deviation	Related to COVID-19?	Deviation Description	Resolution/ Corrective Action	Plan to Prevent Recurrence	IRB Reporting Required?	IRB Notified at Continuing Review?	Planned/ Actual IRB Report Date
xxxxxxxx	xxxxxxxxxxxxxxx	mm/dd/yyyy	mm/dd/yyyy	xxxxxxxx	xxxxxxxx	Yes/No	xxxxxxxx	Yes/No	xxxxxxxx	Yes/No	Yes/No	mm/dd/yyyy

Listing 8: Protocol Deviations (continued)

Deviation Category = Inclusion/Exclusion Criteria

Site	Related Participant IDs	Date of Protocol Deviation	Date Protocol Deviation Entered in EDC	Deviation Type	Reason for Protocol Deviation	Related to COVID-19?	Deviation Description	Resolution/ Corrective Action	Plan to Prevent Recurrence	IRB Reporting Required?	IRB Notified at Continuing Review?	Planned/ Actual IRB Report Date
xxxxxxxx	xxxxxxxxxxxxxxx	mm/dd/yyyy	mm/dd/yyyy	xxxxxxxx	xxxxxxxx	Yes/No	xxxxxxxx	Yes/No	xxxxxxxx	Yes/No	Yes/No	mm/dd/yyyy

**Listing 8: Protocol Deviations (continued)**												
**Deviation Category = Laboratory Assessments**												
**Site**	**Related Participant IDs**	**Date of Protocol Deviation**	**Date Protocol Deviation Entered in EDC**	**Deviation Type**	**Reason for Protocol Deviation**	**Related to COVID-19?**	**Deviation Description**	**Resolution/ Corrective Action**	**Plan to Prevent Recurrence**	**IRB Reporting Required?**	**IRB Notified at Continuing Review?**	**Planned/ Actual IRB Report Date**
xxxxxxxx	xxxxxxxxxxxxxxx	mm/dd/yyyy	mm/dd/yyyy	xxxxxxxx	xxxxxxxx	Yes/No	xxxxxxxx	Yes/No	xxxxxxxx	Yes/No	Yes/No	mm/dd/yyyy

**Listing 8: Protocol Deviations (continued)**												
**Deviation Category = Study Procedures/Assessments**												
**Site**	**Related Participant IDs**	**Date of Protocol Deviation**	**Date Protocol Deviation Entered in EDC**	**Deviation Type**	**Reason for Protocol Deviation**	**Related to COVID-19?**	**Deviation Description**	**Resolution/ Corrective Action**	**Plan to Prevent Recurrence**	**IRB Reporting Required?**	**IRB Notified at Continuing Review?**	**Planned/ Actual IRB Report Date**
xxxxxxxx	xxxxxxxxxxxxxxx	mm/dd/yyyy	mm/dd/yyyy	xxxxxxxx	xxxxxxxx	Yes/No	xxxxxxxx	Yes/No	xxxxxxxx	Yes/No	Yes/No	mm/dd/yyyy

Listing 8: Protocol Deviations (continued)												
Deviation Category = Adverse Event												
**Site**	**Related Participant IDs**	**Date of Protocol Deviation**	**Date Protocol Deviation Entered in EDC**	**Deviation Type**	**Reason for Protocol Deviation**	**Related to COVID-19?**	**Deviation Description**	**Resolution/ Corrective Action**	**Plan to Prevent Recurrence**	**IRB Reporting Required?**	**IRB Notified at Continuing Review?**	**Planned/ Actual IRB Report Date**
xxxxxxxx	xxxxxxxxxxxxxxx	mm/dd/yyyy	mm/dd/yyyy	xxxxxxxx	xxxxxxxx	Yes/No	xxxxxxxx	Yes/No	xxxxxxxx	Yes/No	Yes/No	mm/dd/yyyy

Listing 8: Protocol Deviations (continued)												
Deviation Category = Enrollment Procedures												
**Site**	**Related Participant IDs**	**Date of Protocol Deviation**	**Date Protocol Deviation Entered in EDC**	**Deviation Type**	**Reason for Protocol Deviation**	**Related to COVID-19?**	**Deviation Description**	**Resolution/ Corrective Action**	**Plan to Prevent Recurrence**	**IRB Reporting Required?**	**IRB Notified at Continuing Review?**	**Planned/ Actual IRB Report Date**
xxxxxxxx	xxxxxxxxxxxxxxx	mm/dd/yyyy	mm/dd/yyyy	xxxxxxxx	xxxxxxxx	Yes/No	xxxxxxxx	Yes/No	xxxxxxxx	Yes/No	Yes/No	mm/dd/yyyy

Major PDs are highlighted in grey.

Listing 8: Protocol Deviations (continued)

Deviation Category = Study Medication Management

Site	Related Participant IDs	Date of Protocol Deviation	Date Protocol Deviation Entered in EDC	Deviation Type	Reason for Protocol Deviation	Related to COVID-19?	Deviation Description	Resolution/ Corrective Action	Plan to Prevent Recurrence	IRB Reporting Required?	IRB Notified at Continuing Review?	Planned/ Actual IRB Report Date
xxxxxxxx	xxxxxxxxxxxxxxx	mm/dd/yyyy	mm/dd/yyyy	xxxxxxxx	xxxxxxxx	Yes/No	xxxxxxxx	Yes/No	xxxxxxxx	Yes/No	Yes/No	mm/dd/yyyy

Listing 8: Protocol Deviations (continued)

Deviation Category = Safety Event

Site	Related Participant IDs	Date of Protocol Deviation	Date Protocol Deviation Entered in EDC	Deviation Type	Reason for Protocol Deviation	Related to COVID-19?	Deviation Description	Resolution/ Corrective Action	Plan to Prevent Recurrence	IRB Reporting Required?	IRB Notified at Continuing Review?	Planned/ Actual IRB Report Date
xxxxxxxx	xxxxxxxxxxxxxxx	mm/dd/yyyy	mm/dd/yyyy	xxxxxxxx	xxxxxxxx	Yes/No	xxxxxxxx	Yes/No	xxxxxxxx	Yes/No	Yes/No	mm/dd/yyyy

Major PDs are highlighted in grey.

Listing 8: Protocol Deviations (continued)

Deviation Category = Other Significant Deviations

Site	Related Participant IDs	Date of Protocol Deviation	Date Protocol Deviation Entered in EDC	Deviation Type	Reason for Protocol Deviation	Related to COVID-19?	Deviation Description	Resolution/ Corrective Action	Plan to Prevent Recurrence	IRB Reporting Required?	IRB Notified at Continuing Review?	Planned/ Actual IRB Report Date
xxxxxxxx	xxxxxxxxxxxxxxx	mm/dd/yyyy	mm/dd/yyyy	xxxxxxxx	xxxxxxxx	Yes/No	xxxxxxxx	Yes/No	xxxxxxxx	Yes/No	Yes/No	mm/dd/yyyy
