# Supplementary material for: Rapid Initiation of Injection Naltrexone for Opioid Use Disorder: A Stepped-Wedge Cluster Randomized Clinical Trial
Source: JAMA Netw Open. 2024 May 8;7(5):e249744. doi: 10.1001/jamanetworkopen.2024.9744 (PMC11079685; doi:10.1001/jamanetworkopen.2024.9744)
Supplement: Supplement 4. — Data Sharing Statement [file jamanetwopen-e249744-s004.pdf]

Data Sharing Statement

Shulman. Rapid Initiation of Injection Naltrexone for Opioid Use Disorder. *JAMA Netw Open*. Published May 08, 2024. doi:10.1001/jamanetworkopen.2024.9744

Data
Data available: Yes
Data types: Deidentified participant data
How to access data: This data will be available through the NIDA data share website along with the publicly available dataset on https://datashare.nida.nih.gov/divisions/ctn
When available: beginning date: 07-01-2024

Supporting Documents
Document types: Statistical/analytic code, Informed consent form
How to access documents: THese will be made available by request via email.
When available: With publication

Additional Information
Who can access the data: Anyone requesting the data, researchers whose proposed use of the data has been approved
Types of analyses: This will be made available for any research purpose.
Mechanisms of data availability: This is will be made available with investigator support with a signed data access agreement.
